# Atroposelective Synthesis of Azobenzenes by Palladium-Catalyzed Cross-Coupling of Racemic Biaryl Triflates and Diazenyl Pronucleophiles

**DOI:** 10.1021/jacs.5c09097

**Published:** 2025-08-25

**Authors:** Kezhuo Zhang, Martin Oestreich

**Affiliations:** Institut für Chemie, 26524 Technische Universität Berlin, Straße des 17. Juni 115, 10623 Berlin, Germany

## Abstract

A kinetic resolution of racemic biaryl monotriflates by an atroposelective palladium-catalyzed diazenylation enables the synthesis of azobenzene derivatives decorated with an axially chiral substituent. The C­(sp^2^)–N­(sp^2^) cross-coupling reaction makes use of silylated diazenes as diazenyl anion equivalents, and chiral ferrocene-based bisphosphine ligands act as effective supporting ligands. The resulting chiral azobenzenes bearing binaphthyl and naphthyl/phenyl backbones undergo reversible *trans*–*cis* isomerization under irradiation with LEDs of different wavelengths, highlighting their potential use in photoresponsive chiroptical materials.

## Graphical abstract

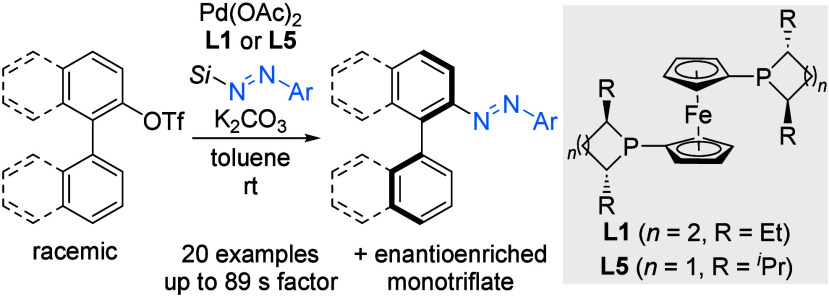

Compared to axially chiral C- and O-functionalized biaryls, the synthesis of N-functionalized biaryls is considerably less explored.^1^ To date, five main strategies have been established for the construction of axially chiral biaryl amines and their derivatives. Aside from asymmetric cross-coupling to forge aryl–aryl bonds^2^ and annulation approaches,^3^ modification of existing biaryl amine structures as part of a kinetic resolution (KR) provides an alternative access.^4^ Of note, Fernández, Lassaletta, and co-workers introduced an enantioselective Buchwald–Hartwig cross-coupling in the form of a kinetic asymmetric transformation (DYKAT) (Scheme 1A, left).^5^ The fifth approach is based on desymmetrization.^4d,6^ Notably, Gu and co-workers reported a highly atroposelective ring-opening amination of achiral cyclic diaryliodonium salts (Scheme 1A, middle).^7^ Another typical example of enantioselective C­(sp^2^)–N­(sp^3^) bond formation is a desymmetrization of 1,1′-biaryl-2,6-dibromides by Cong and co-workers (Scheme 1A, right).^8^

**Scheme 1:**
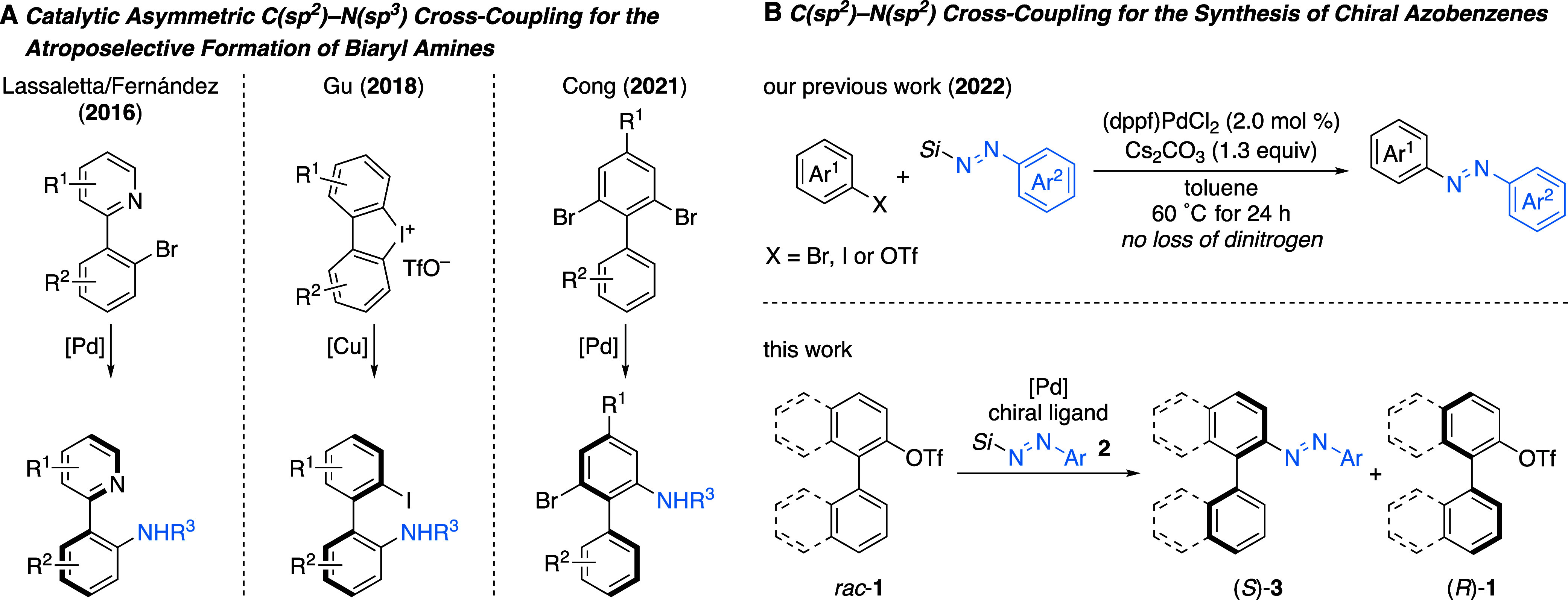
Catalytic Asymmetric C­(sp^2^)–N­(sp^3^) Cross-Coupling for the Atroposelective Formation of Biaryl Amines and Planned C­(sp^2^)–N­(sp^2^) Cross-Coupling for the Synthesis of Chiral Azobenzenes^a^

a dppf = 1,1′-bis­(diphenylphosphino)­ferrocene; *Si* = triorganosilyl.

A few years ago, our laboratory had begun to investigate diverse reactivity of silicon-masked aryl-substituted diazenes.^9^ In 2022, we introduced an unprecedented palladium catalysis where silylated diazenes serve as precursors of diazenyl anions (Scheme 1B, top),^10a^ otherwise fleeting intermediates prone to rapid loss of dinitrogen.^10b^ This rare palladium-catalyzed C­(sp^2^)–N­(sp^2^) cross-coupling^7c^ allowed for the direct installation of an azo unit at a prefunctionalized arene, thereby enabling the synthesis of nonsymmetric azobenzenes. An asymmetric variant of this reaction is not known, but it would be appealing because it could grant access to axially chiral azobenzenes. This class of photoresponsive molecules with their unique physicochemical properties such as reversible *trans*–*cis* isomerization have been shown to be particularly attractive for the design of chiroptical materials.^11^ Their synthesis typically starts from enantioenriched BINAM^12a^ and BINOL^12b^ (for axially chiral systems) or is done by chromatographic separation on chiral stationary phases (for planarly chiral systems).^12c^ To the best of our knowledge, there have been no reports on the asymmetric synthesis of these axially chiral azobenzenes starting from racemic or prochiral substrates.^12^ We hypothesized that racemic biaryl monotriflates could engage in a KR process in the presence of a chiral palladium catalyst (Scheme 1B, bottom).

Our study commenced with 2-(trifluoromethanesulfonyloxy)-1,1′-binaphthyl (*rac*-**1a**) and *N*-phenyl-*N*′-trimethylsilyldiazene (**2a**) as model substrates (Table 1; for variation of the substitution pattern at the silicon atom, see Table S2). Guided by our previous work, where dppf had been found to be crucial for suppressing dinitrogen extrusion,^10c^ we decided to begin with chiral ferrocene-type bisphosphine ligands. We optimized the reaction conditions employing Pd­(OAc)_2_ as the precatalyst and CsOAc as the base in toluene as the solvent. Initially, a series of ferrocene-based ligands **L1**–**L5** was examined. Using **L1**, we achieved the formation of the desired axially chiral azobenzene product (*S*)-**3aa** in a good yield of 53% with a promising selectivity factor (*s*) of 20 (entry 1). In contrast, the 2,5-diisopropyl-substituted ligand **L2** resulted in both a low yield and low selectivity (entry 2). Next, we investigated structurally similar 2,4-disubstituted FerroTANE ligands **L3**–**L5**.^13^ For ligands bearing small R groups (Me and Et), the yield and selectivity remained low (entries 3 and 4). The 2,4-diisopropyl-substituted ligand **L5** provided a substantially higher selectivity but lower yield (entry 5). The reaction also proceeded in the absence of a base, albeit with a decreased yield (entries 6 and 7). Additional ferrocene-based Josiphos-type ligands did not lead to any improvement (see the Supporting Information for details). Conducting the reaction at room temperature led to a lower yield of (*S*)-**3aa** but an improved *s* factor (entry 8). K_2_CO_3_ instead of CsOAc did improve the yield of **3aa** and the selectivity, making it the base of choice (entry 9). Given that **L5** exhibited a high *s* factor, we also probed various bases using **L5** at room temperature over prolonged reaction times (entries 10–13). To our delight, the *s* factors of this reaction were close to 40 when CsOAc, KOAc, or K_2_CO_3_ was used as the base. Considering the high hygroscopicity of CsOAc and KOAc, which may lead to reproducibility issues, we opted for K_2_CO_3_ as the base. Increasing the amount of K_2_CO_3_ from 0.3 to 1.0 equiv resulted in a 42% NMR yield of (*S*)-**3aa** with *s* = 40 (entry 14). The absolute configuration was assigned as *S* for **3aa** by comparison of the HPLC traces of the recovered binaphthyl triflate **1a** with independently prepared samples of (*R*)-**1a** and (*S*)-**1a** (see the Supporting Information for details).

**Table 1:**
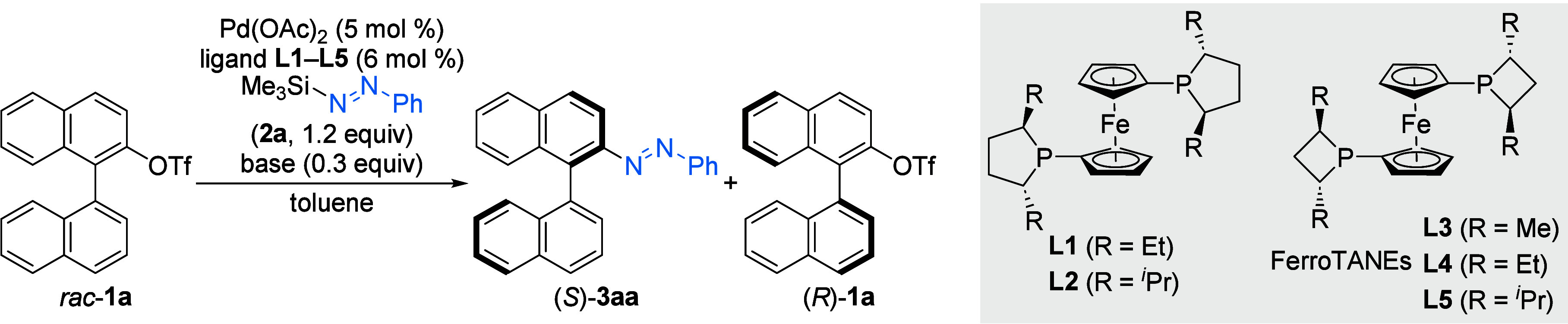
Optimization of the Reaction Conditions^a^

entry	ligand	*T* (°C)	*t* (h)	base	yield of **3aa** (%)^b^	ee of **3aa** (%)^c^	ee of **1a** (%)^c^	conv. (%)^d^	*s* ^e^
1​	**L1**	60	4	CsOAc	53	72	92	56	20
2​	**L2**	60	24	CsOAc	8	47	19	29	3
3​	**L3**	60	24	CsOAc	15	54	14	21	4
4​	**L4**	60	24	CsOAc	26	78	34	30	11
5​	**L5**	60	24	CsOAc	34	82	53	39	17
6​	**L1**	60	4	none	41	80	63	44	17
7​	**L5**	60	4	none	19	90	32	26	26
8​	**L1**	25	60	CsOAc	28	78	88	53	23
9​	**L1**	25	48	K_2_CO_3_	39	85	74	47	27
10​	**L5**	25	60	CsOAc	33	89	63	41	33
11​	**L5**	25	72	KOAc	31	92	50	35	39
12​	**L5**	25	72	Cs_2_CO_3_	trace				
13​	**L5**	25	72	K_2_CO_3_	21	93	34	26	38
14​^f^	**L5**	25	72	K_2_CO_3_	42^g^	89	77	46	40

a Reactions were performed on a 0.10 mmol scale at a concentration of 0.50 M under an argon atmosphere.

b Yields were determined by ^1^H NMR spectroscopy using CH_2_Br_2_ as an internal standard.

c The ee values were determined by HPLC analysis on a chiral stationary phase.

d Calculated conversion: *C* = ee_SM_/(ee_SM_ + ee_PR_), where ee_SM_ is the ee of recovered **1a** and ee_PR_ is the ee of **3aa**.

e *s* = ln­[(1 – *C*)­(1 – ee_SM_)]/ln­[(1 – *C*)­(1 + ee_SM_)].

f 1.0 equiv of K_2_CO_3_.

g The isolated yield was 37% after flash chromatography on silica gel.

With the optimized reaction conditions in hand, we explored the substrate scope of this atroposelective cross-coupling (Scheme 2). The coupling reactions of silyldiazenes substituted with halogen atoms provided the corresponding axially chiral azobenzenes (*S*)-**3ab**, (*S*)-**3ac**, and (*S*)-**3ad** in good to excellent yields with excellent enantioselectivity. Electron-donating substituents at the *para* position were also compatible, and the desired products (*S*)-**3ae** with a methyl and (*S*)-**3af** with a methoxy substituent were obtained in good yields with good *s* factors. A lower reaction rate was observed for the silyldiazene bearing an electron-withdrawing ester group, and even after extending the reaction time to 7 days, no increase in yield of (*S*)-**3ag** was observed. Next, we gauged the substitution pattern of the biaryl triflate electrophile. Both 6-methoxy- and 6-trimethylsilyl-substituted binaphthyl triflates were well-tolerated using **L5** as the ligand, which led to higher reactivity than **L1**, affording products with *s* factors of 63 for (*S*)-**3bb** and 38 for (*S*)-**3ca**, respectively. Substitution at the *ortho* position with a methyl group resulted in good to excellent enantioselectivity and *s* factors for products (*S*)-**3da** and (*S*)-**3db** at an elevated reaction temperature of 60 °C. We attribute this loss of reactivity to steric hindrance. Although the yield was lower, substitution at the *ortho* position proved to be beneficial for enantioselectivity, and hence, an additional *o*-fluoro substituent was tested. The yield remained moderate when the reaction time was extended to 7 days, presumably due to the electron deficiency, but the *s* factor was still good for (*S*)-**3ea**. We continued to expand the substrate scope by modifying the other aryl moiety of the electrophile. Replacing the naphthyl group with a phenanthryl substituent as in (*S*)-**3fe** resulted in a good yield and a good *s* factor. The naphthyl group was also successfully replaced by *ortho*-substituted phenyl groups, which coupled effectively with silyldiazenes. Substitution at the *ortho* position with methoxy as in (*S*)-**3ga** and (*S*)-**3gb**, ethyl as in (*S*)-**3ha**, or methyl as in (*S*)-**3ia** and (*S*)-**3ja** gave good yields and selectivities. A halogen substituent was also well-tolerated, affording (*S*)-**3ka** in good yield with a moderate *s* factor. An *o*-phenyl substituent led to a moderate yield and selectivity for (*S*)-**3la**. Finally, a significant decrease in enantioselectivity was observed when the triflyloxy-substituted naphthyl moiety was exchanged for a phenyl group as in **3ma** and **3na** (gray box). Typically, the reaction predominantly yields the *trans* isomer, with most *trans*:*cis* ratios exceeding 92:8 (deviating *trans*:*cis* ratios: 89:11 for **3ae**, 88:12 for **3ca**, and 85:15 for **3ea**). A minor amount of the *cis* isomer can be observed in the NMR spectrum.

**Scheme 2:**
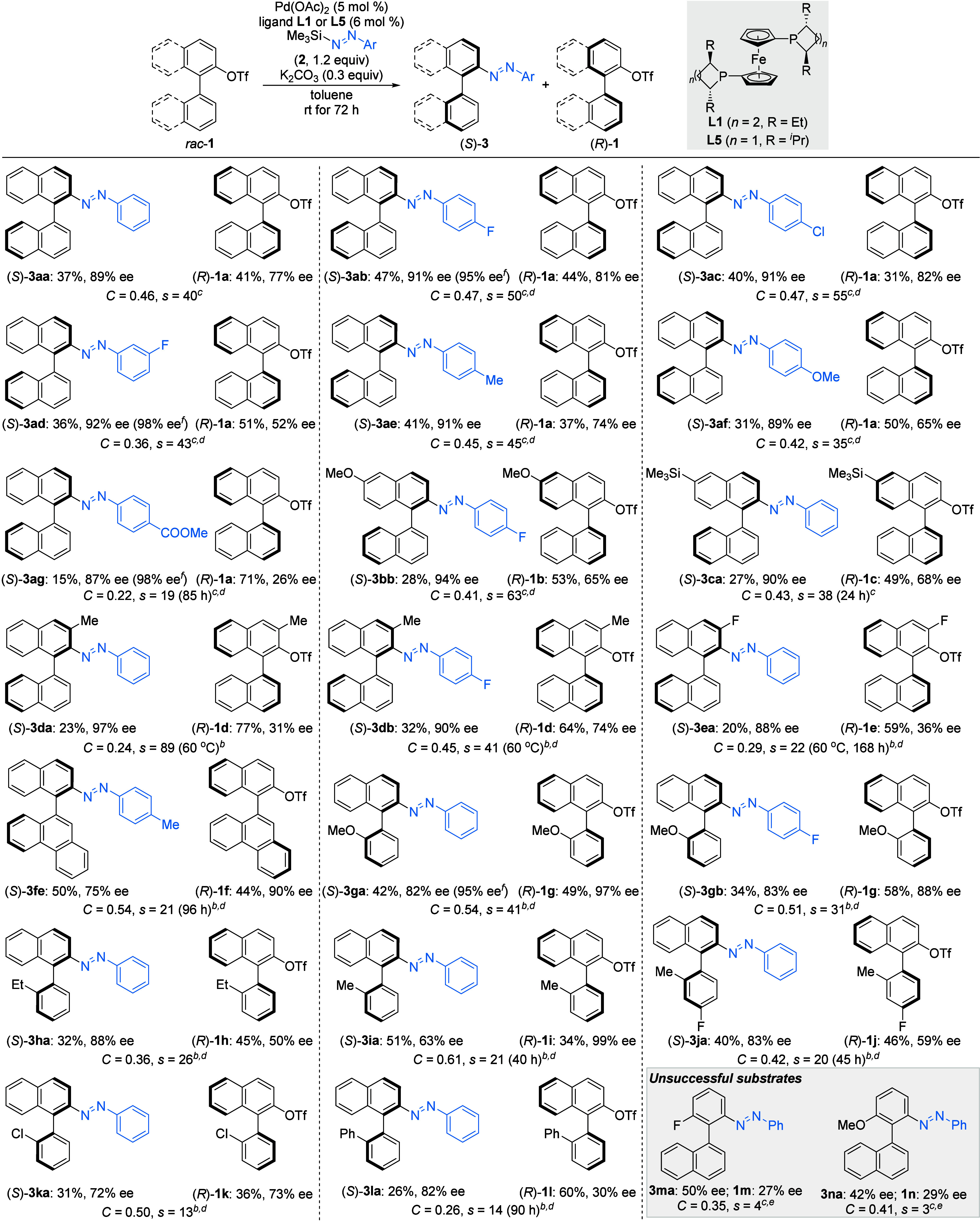
Substrate Scope of the Atroposelective C­(sp^2^)–N­(sp^2^) Cross-Coupling^a^

a All reactions were performed on a 0.10 mmol scale at a concentration of 0.50 M under an argon atmosphere. Isolated yields are reported; ee values were determined by HPLC analysis on a chiral stationary phase. Calculated conversion: *C* = ee_SM_/(ee_SM_ + ee_PR_), where ee_SM_ is the ee of recovered **1** and ee_PR_ is the ee of **3**. *s* = ln­[(1 – *C*)­(1 – ee_SM_)]/ln­[(1 – *C*)­(1 + ee_SM_)].
b
**L1** as the ligand.
c
**L5** as the ligand.
d A 10 mol % loading of Pd­(OAc)_2_ and a 12 mol % loading of the indicated ligand were used.
e Not isolated.
f The ee value after recrystallization from *n*-hexane.

To investigate the kinetic resolution process, enantiopure (*S*)-**1a** and (*R*)-**1a** were individually subjected to the standard conditions using **L5** as a ligand (Scheme 3). When (*S*)-**1a** was used, the desired product (*S*)-**3aa** was obtained in 47% yield (eq 1). In contrast, only a very low yield of (*R*)-**3aa** was observed with (*R*)-**1a** (eq 2). In both cases, no racemization of the recovered triflate **1a** was detected. These results suggest that ligand **L5** matches with the *R* configuration of the substrate, thereby facilitating efficient enantiomer discrimination in the KR.

**Scheme 3:**
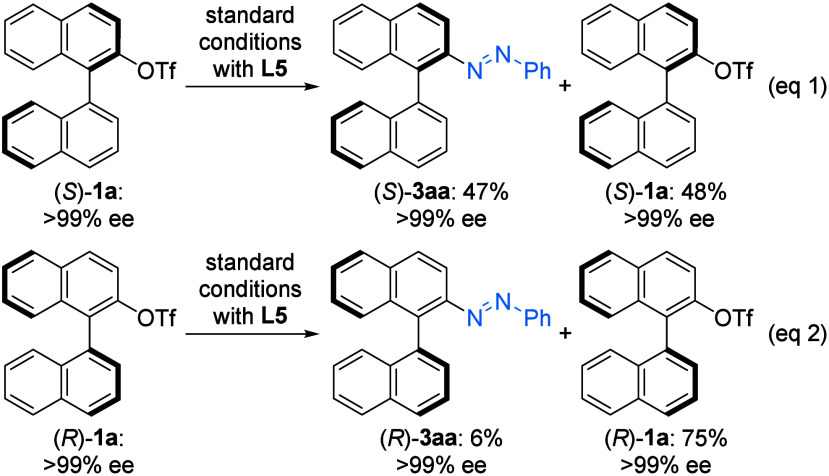
Match/Mismatch Control Experiments^a^

a Reactions were performed on a 0.10 mmol scale under the standard conditions. Isolated yields after flash chromatography on silica gel are reported.

The products *trans*-**3** obtained from our atroposelective cross-coupling reaction are both chemically and thermally stable and exhibit the expected reversible photoswitchable behavior in organic solvents (Scheme 4A, left). To evaluate the photoresponsiveness, we irradiated the *trans*-configured model compound *trans*-(*S*)-**3aa** (*trans*:*cis* = 91:9) with 390 nm light for 30 min in C_6_D_6_, successfully obtaining the corresponding *cis* isomer *cis*-(*S*)-**3aa** (*trans*:*cis* = 18:82); the reversible, photoinduced *trans*-to-*cis* interconversion was clearly seen by NMR spectroscopy (see the Supporting Information for details). Unexpectedly, when the same experiment with *trans*-(*S*)-**3aa** (*trans*:*cis* = 95:5) was repeated in CDCl_3_ for 40 min, *N*-phenyl-7*H*-dibenzo­[*c*,*g*]­carbazol-7-amine (**4aa**) was obtained quantitatively (Scheme 4A, right).^14^ NMR monitoring suggests that the reaction proceeds via an initial *trans*-to-*cis* isomerization followed by a proton-initiated intramolecular electrophilic aromatic substitution. We favor this ionic mechanism over a radical pathway^15^ also because the addition of K_2_CO_3_ suppresses the cyclization event. Moreover, we recorded the UV–vis absorption spectra of several chiral azobenzenes: *trans*-(*S*)-**3ab**, (*S*)-**3ae**, (*S*)-**3fe**, and (*S*)-**3ia** (Scheme 4B). These compounds exhibit similar spectral profiles, and the absorption maxima are at around 337, 343, 341, and 334 nm, respectively. Irradiation at 390 nm induced a rapid photoisomerization of *trans*-(*S*)-**3aa** to *cis*-(*S*)-**3aa** in toluene, reaching a photostationary state within approximately 5 s (Scheme 4C). Compared with *trans*-(*S*)-**3aa**, *cis*-(*S*)-**3aa** shows decreased absorbance at 333 nm and increased absorbance at 453 nm. The presence of an isosbestic point at 422 nm, where all absorption curves intersect, indicates a clean interconversion in toluene between two species without the accumulation of other intermediates. These results show that these axially chiral benzenes possess good photoresponsiveness. Additionally, most of them exhibit very high specific rotation values (maximum around 600; see the Supporting Information for details), suggesting their potential applications in chiral sensing and optoelectronic materials.^16^

**Scheme 4:**
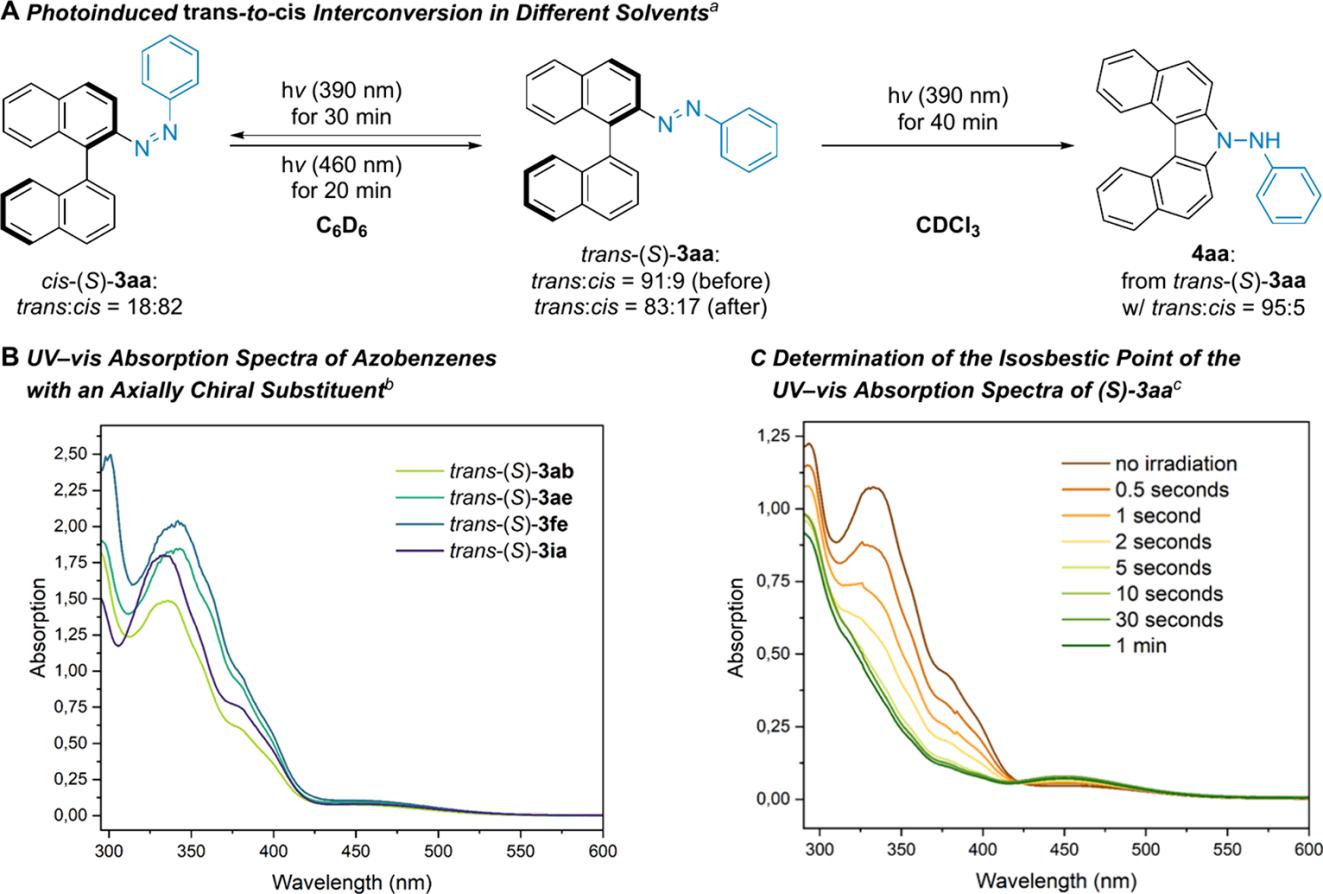
Photophysical Properties

a The reactions were performed in an NMR tube at a concentration of 0.056 M.
b UV–vis absorption spectra of various products (*S*)-**3** (in toluene, 100 μmol).
c UV–vis absorption spectra of **3aa** (in toluene, 100 μmol) after UV irradiation at 390 nm for 0 s, 0.5 s, 1 s, 2 s, 5 s, 10 s, 30 s, and 1 min.

In summary, we developed an atroposelective cross-coupling between silylated diazenes and biaryl monotriflates for the direct formation of C­(sp^2^)–N­(sp^2^) bonds. This kinetic resolution exhibits a synthetically useful selectivity factors. The approach enables the asymmetric synthesis of axially chiral azobenzenes from racemic substrates using palladium with a chiral bisphosphine ligand as a catalytic system. The reaction displays broad scope with good functional group tolerance, and reversible *trans*-to-*cis* isomerization of these chiral azobenzene derivatives indicates potential applications in materials science.

## Supplementary Material



## Data Availability

The data underlying this study are available in the published article and its Supporting Information.

## Notes

Experimental procedures, including the synthesis of starting materials and characterization data, and NMR spectral data for all new compounds (PDF)
