# Supplementary material for: Atroposelective Synthesis of Azobenzenes by Palladium-Catalyzed Cross-Coupling of Racemic Biaryl Triflates and Diazenyl Pronucleophiles
Source: J Am Chem Soc. 2025 Aug 25;147(36):32329–34. doi: 10.1021/jacs.5c09097 (PMC12426921; doi:10.1021/jacs.5c09097)
Supplement: Supplementary file 1 [file ja5c09097_si_001.pdf]

Atroposelective Synthesis of Azobenzenes by Palladium-Catalyzed Cross-Coupling of Racemic Biaryl Triflates and Diazenyl Pronucleophiles

Kezhuo Zhang and Martin Oestreich*

Institut für Chemie, Technische Universität Berlin

Straße des 17. Juni 115, 10623 Berlin, Germany

martin.oestreich@tu-berlin.de

Supporting Information

Table of Contents

1 General Information 3

2 Optimization Study 4

3 General Procedures 6

3.1 General Procedure for the Preparation of Biaryl monotriflates *rac*-**1** (GP 1)... 6

3.2 Synthesis of *N*-Aryl-*N*'-silyldiazenes **2** 6

3.3 Typical Procedure for the Synthesis of Enantioenriched Products **1** & **3** (GP 2) 7

4 Characterization Data 8

4.1 Characterization Data of Biaryl monotriflates *rac*-**1** 8

4.2 Characterization Data of Enantioenriched Products **1** and **3** 166

5 Match/Mismatch Control Experiments 37

5.1 Synthesis of (*S*) and (*R*)-**1a** from (*R*) and (*S*)-BINOL 37

5.2 Match/Mismatch Control Experiments 38

6 Photophysical Properties 39

6.1 Light Control Experiments 39

6.2 UV-VIS Absorption Spectra 42

7 Determination of the Absolute Configuration 45

8 HPLC Traces 47

9 NMR Spectra 92

10 References 184

1 General Information

All reactions were performed in flame-dried glassware using conventional Schlenk techniques under a static pressure of nitrogen unless stated otherwise. Liquids and solutions were transferred with syringes. Technical grade solvents for extraction and chromatography were distilled prior to use. Toluene dried over CaH_2, distilled, degassed by three freeze-pump-thaw cycles, and stored in a glovebox over thermally activated 4 Å molecular sieves. Dichloromethane (CH_2Cl_2) were dried over CaH_2, freshly distilled prior to use. Analytical thin layer chromatography (TLC) was performed on ALUGRAM® Xtra SIL G/UV_{254} TLC-Sheets by Macherey-Nagel. Flash column chromatography was performed on silica gel 60 (40-63 *μ*m, 230-400 mesh, ASTM) by Grace using the indicated solvents. ^{1}H, ^{13}C, and ^{19}F spectra were recorded in $CDCl_3$ or C_6D_6 on Bruker AV400 or AV500 instruments. Chemical shifts were reported in parts per million (ppm) and were referenced to the residual solvent resonance as the internal standard ($CHCl_3$: δ = 7.26 ppm for ^{1}H NMR and $CDCl_3$: δ = 77.16 ppm for ^{13}C NMR; C_6D_6: δ = 7.16 ppm for ^{1}H NMR). ^{19}F NMR spectra are referenced in compliance with the unified scale for NMR chemical shifts as recommended by the IUPAC stating the chemical shift relative to CCl_3F. Data were reported as follows: chemical shift, multiplicity (s = singlet, d = doublet, t = triplet, q = quartet, m = multiplet), coupling constants (Hz), and integration. Infrared (IR) spectra were recorded on an *Agilent Technologies Cary* 630 FT-IR spectrometer equipped with an ATR unit and the signals were reported in wave-numbers (cm^{-1}). Melting points (m.p.) were determined with a Stuart Scientific SMP20 melting point apparatus and were not corrected. Enantiomeric excesses were determined by analytical high performance liquid chromatography (HPLC) analysis on an *Agilent Technologies* 1290 Infinity instrument with a chiral stationary phase using a *Daicel* Chiralcel OD-H column, *Daicel* Chiralcel AD-H column, *Daicel* Chiralcel OJ-H column, *Daicel* Chiralcel IB, or a *Daicel* Chiralcel IA column (^{n}heptane/*iso*propanol mixtures as solvent). High resolution mass spectra (HRMS) were obtained from the Center for Mass Spectrometry at the *Institut für Chemie, Technische Universität Berlin* on a Thermo Fisher Scientific LTQ Orbitrap XL apparatus using APCI or ESI technique with a linear ion trap analyzer. Optical rotations were measured on a *Schmidt & Haensch Polartronic* H532 Polarimeter. The enantioenriched compound was dissolved in the specified solvent and transferred to a cuvette. The following equation was used to calculate the specific rotation:

$$[\alpha]_D^T = \frac{[\alpha] * 100}{c * d}$$

The measured rotation is described by [α]. All measurements were carried out using the sodium D-line (λ = 589 nm) as the light source, indicated by the index "D" at room temperature (T). The cuvette length is d = 1 dm and the concentration is reported in c = g/100 mL.

2 Optimization Study

Table S1. Screening of Reaction Conditions[a]

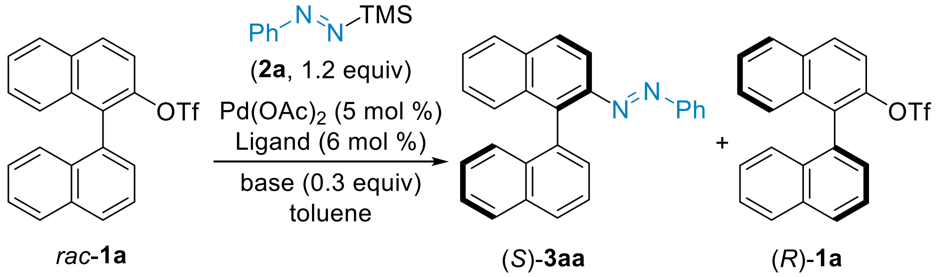

entry	ligand	T (°C)	t (h)	base	yield of **3aa** (%)[b]	ee of **3aa** (%)[c]	ee of **1a** (%)[c]	Conv (%)[d]	S[e]
1	**L6**	60	24	Cs_2CO_3	n.d.[f]	9	n.d.[f]	--	--
2	**L7**	60	24	Cs_2CO_3	trace				
3	**L8**	60	48	CsOAc	trace	--	--	--	--
4	**L9**	60	48	CsOAc	trace	--	--	--	--
5	**L10**	60	4	w/o	18	40	8	17	7
6	**L11**	60	4	w/o	15	6	5	45	1
7	**L12**	rt	72	K_2CO_3	42	24	10	30	2
8[g]	**L1**	60	4	CsOAc	49	67	92	58	16
9[h]	**L1**	60	4	CsOAc	49	80	76	49	23

[a]Reactions were performed on a 0.10 mmol scale under argon atmosphere. [b]Yields were determined by ^{1}H NMR spectroscopy using CH_2Br_2 as an internal standard. [c]Ee was determined by HPLC analysis on a chiral stationary phase. [d]Calculated conversion, $C = ee_{SM}/(ee_{SM} + ee_{PR})$, ee_{SM} is the enantiomeric excess of **1a** and ee_{PR} is the enantiomeric excess of **3aa**. [e]Selectivity $s = \ln(1 - C)(1 - ee_{SM}) / \ln(1 - C)(1 + ee_{SM})$; [f]Not tested. [g]$PhCF_3$ as solvent. [h]1,2-Difluorobenzene as solvent.

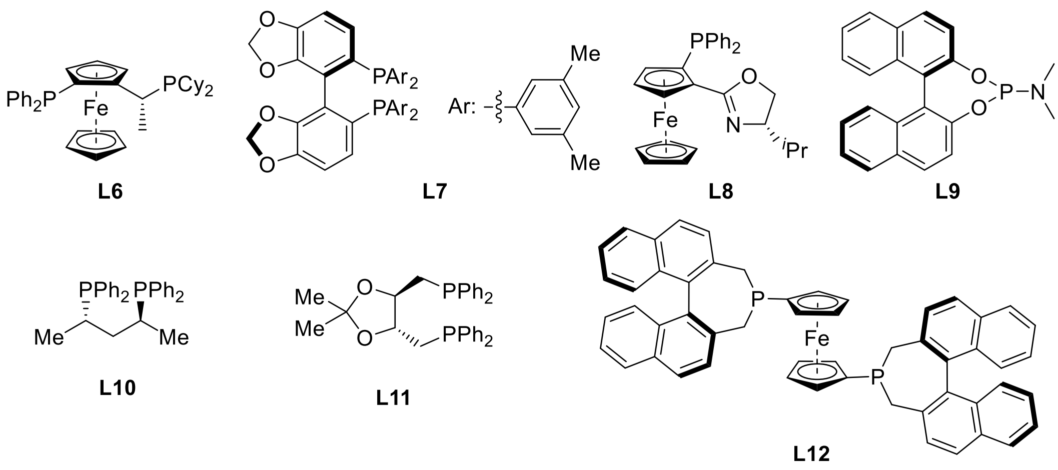

Table S2. Screening of Different Silyl Groups

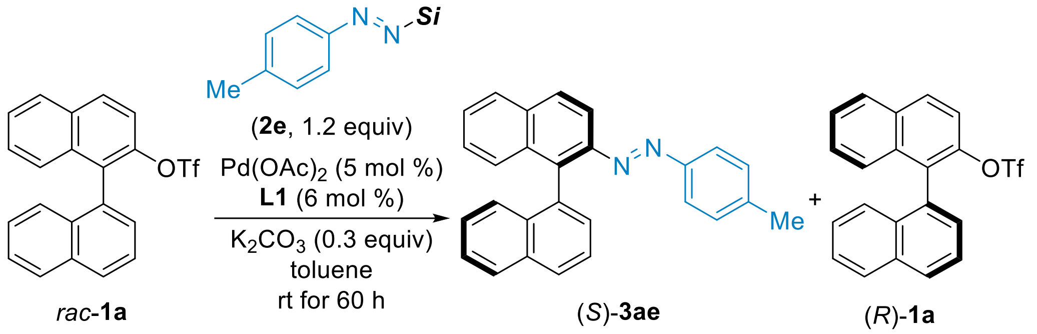

entry	*Si*	ee of **3ae** (%)[b]	ee of **1a** (%)[b]	Conv (%)[c]	s[d]
1	Me_3Si	88	50	36	25
2	Me_2PhSi	92	11	11	27
3	$MePh_2Si$	87	26	23	19
4	$(^iPr)_3Si$	--	--	trace	--
5	$^tBuMe_2Si$	--	--	trace	--

[a]Reactions were performed on a 0.10 mmol scale under argon atmosphere. [b]Ee was determined by HPLC analysis on a chiral stationary phase. [c]Calculated conversion, $C = ee_{SM}/(ee_{SM} + ee_{PR})$, ee_{SM} is the enantiomeric excess of **1a** and ee_{PR} is the enantiomeric excess of **3ae**. [d]Selectivity $s = \ln(1 - C)(1 - ee_{SM}) / \ln(1 - C)(1 + ee_{SM})$.

3 General Procedures

3.1 General Procedure for the Preparation of Biaryl monotriflates *rac*-1 (GP 1)

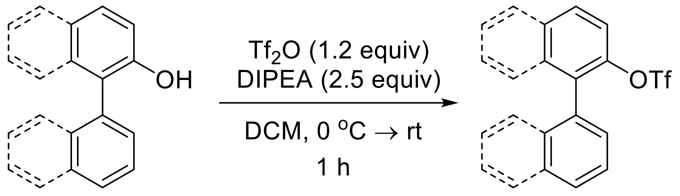

S1 *rac*-**1**

Tf_2O (Trifluoromethanesulfonic anhydride, 1.2 equiv) is added dropwise to a solution of biaryl alcohol **S1** (1.0 equiv) and DIPEA (2.5 equiv) in CH_2Cl_2 at 0 °C. The reaction mixture is stirred at room temperature for 1 h. Upon completion (monitored by TLC), the reaction is quenched by the addition of water, and the resulting mixture is extracted with CH_2Cl_2 (3 × 10 mL). The combined organic phases are dried over Na_2SO_4 and concentrated under reduced pressure. Purification of the residue by flash column chromatography on silica gel using *n*-pentane and CH_2Cl_2 as the eluent affords biaryl trifluoromethanesulfonate *rac*-**1**. (**S1a**–**j**, **S1l**–**n** were prepared according to reported procedure.[S1] **S1k** was prepared according to reported procedure.[S2])

3.2 Synthesis of *N*-Aryl-*N'*-silyldiazenes 2

All *N*-aryl-*N'*-silyldiazenes were prepared according to our previously reported procedures.[S3]

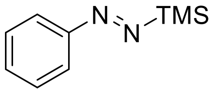

2a

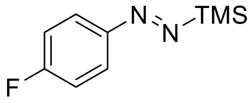

2b

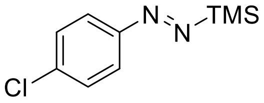

2c

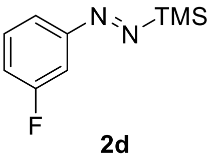

2d

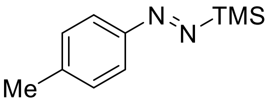

2e

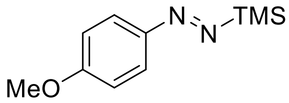

2f

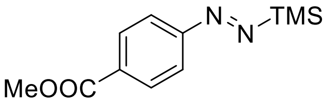

2g

(*E*)-1-phenyl-2-(trimethylsilyl)diazene (**2a**)
(*E*)-1-(4-fluorophenyl)-2-(trimethylsilyl)diazene (**2b**)
(*E*)-1-(4-chlorophenyl)-2-(trimethylsilyl)diazene (**2c**)
(*E*)-1-(3-fluorophenyl)-2-(trimethylsilyl)diazene (**2d**)
(*E*)-1-(4-methyphenyl)-2-(trimethylsilyl)diazene (**2e**)
(*E*)-1-(4-methoxyphenyl)-2-(trimethylsilyl)diazene (**2f**)
methyl (E)-4-((trimethylsilyl)diazenyl)benzoate (**2g**)

3.3 Typical Procedure for the Synthesis of Enantioenriched Products 1 & 3 (GP 2)

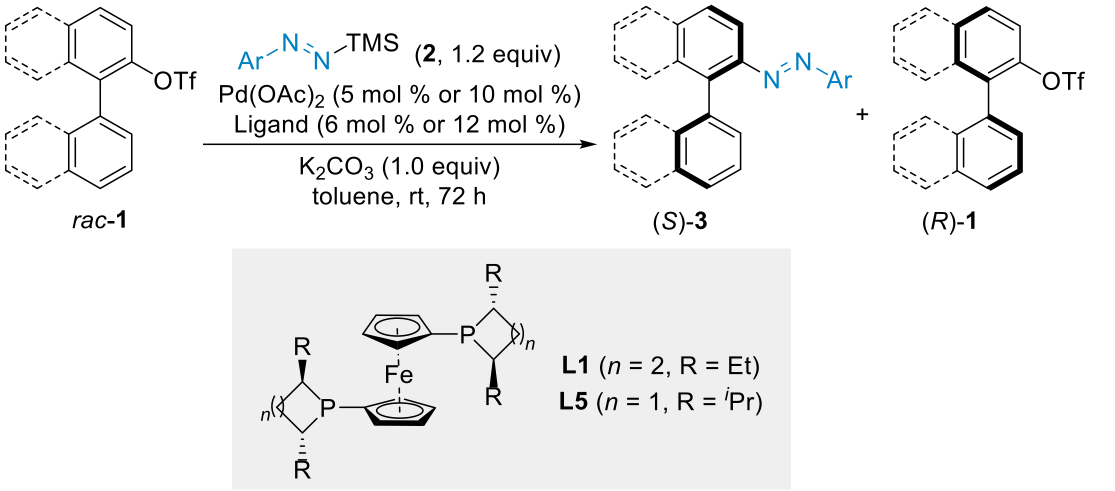

In an argon-filled glovebox, an oven-dried 1.5 mL vial was charged with palladium acetate (5 mol % or 10 mol %), **L1** or **L5** (6 mol % or 12 mol %) and toluene (0.1 mL). The resulting suspension was stirred for 5 min at room temperature, at which time potassium carbonate (0.100 mmol, 1.00 equiv), biaryl trifluoromethanesulfonate **1** (0.10 mmol, 1.0 equiv), *N*-aryl-*N*'-silyldiazene **2** (0.12 mmol, 1.2 equiv) and toluene (0.1 mL) were added. The reaction stirred for 72 h at 25 °C. The reaction was quenched by filtration over silica, and flash column chromatography on silica gel.

4 Characterization Data

4.1 Characterization Data of Biaryl monotriflates *rac*-1

[1,1'-binaphthalen]-2-yl trifluoromethanesulfonate (*rac*-1a)

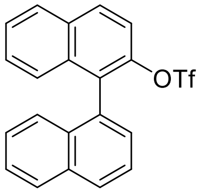

rac-**1a**
$C_{21}H_{13}F_3O_3S$
M = 402.38 g/mol

Prepared according to **GP 1** from [1,1'-binaphthalen]-2-ol (**S1a**, 1.04 g, 3.85 mmol, 1.00 equiv), DIPEA (1.68 mL, 9.63 mmol, 2.50 equiv) and Tf_2O (0.78 mL, 4.6 mmol, 1.2 equiv) in CH_2Cl_2 (10 mL). Flash column chromatography on silica gel using *n*-pentane and CH_2Cl_2 as the eluent (40:1→20:1) afforded substrate *rac*-**1a** as a pale yellow solid (1.21 g, 78% yield).

R*f* = 0.40 (*n*-hexane/CH_2Cl_2=10:1). **^{1}H NMR** (500 MHz, $CDCl_3$, 298 K): δ = 8.10–7.93 (m, 4H), 7.71–7.63 (m, 1H), 7.62–7.47 (m, 4H), 7.42–7.30 (m, 3H), 7.29–7.21 (m, 1H). **^{13}C{^{1}H} NMR** (126 MHz, $CDCl_3$, 298 K): δ = 145.1, 134.0, 133.8, 132.6, 132.6, 131.0, 130.7, 130.6, 129.4, 129.3, 128.5, 128.3, 127.7, 127.20, 127.16, 126.6, 126.2, 125.8, 125.3, 119.6, 118.4 (q, *J* = 319.2 Hz). **^{19}F NMR** (471 MHz, $CDCl_3$, 298 K) δ = −74.4. For **HRMS** and **IR** data, see those reported for the enantioriched compound (*R*)-**1a**.

6-methoxy-[1,1'-binaphthalen]-2-yl trifluoromethanesulfonate (*rac*-1b)

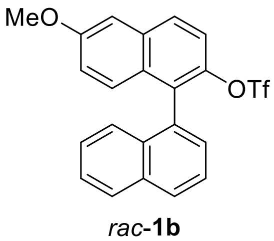

rac-**1b**
$C_{22}H_{15}F_3O_4S$
M = 432.41 g/mol

Prepared according to **GP 1** from 6-methoxy-[1,1'-binaphthalen]-2-ol (**S1b**, 563 mg, 1.87 mmol, 1.00 equiv), DIPEA (0.83 mL, 4.8 mmol, 2.5 equiv) and Tf_2O (0.38 mL, 2.2 mmol, 1.2 equiv) in CH_2Cl_2 (10 mL). Flash column chromatography on silica gel using *n*-pentane and CH_2Cl_2 as the eluent (20:1→5:1) afforded substrate *rac*-**1b** as a white solid (499 mg, 61% yield).

R*f* = 0.35 (*n*-hexane/CH_2Cl_2=5:1). **^{1}H NMR** (500 MHz, $CDCl_3$, 298 K): δ = 8.02 (d, *J* = 8.3

Hz, 1H), 7.99–7.89 (m, 2H), 7.67–7.61 (m, 1H), 7.57–7.47 (m, 3H), 7.36–7.30 (m, 1H), 7.29–7.22 (m, 3H), 7.03 (dd, *J* = 9.2, 2.6 Hz, 1H), 3.93 (s, 3H). **$^{13}C\{^1H\}$ NMR** (126 MHz, $CDCl_3$, 298 K): *δ* = 158.6, 143.6, 134.1, 133.7, 132.6, 130.9, 130.9, 129.3, 129.2, 129.0, 128.7, 128.5, 126.6, 126.2, 125.8, 125.3, 120.5, 120.1, 118.3 (q, *J* = 283.2 Hz), 106.2, 55.6. **^{19}F NMR** (471 MHz, $CDCl_3$, 298 K) *δ* = −74.5. For **HRMS** and **IR** data, see those reported for the enantioriched compound (*R*)-**1b**.

6-(trimethylsilyl)-[1,1'-binaphthalen]-2-yl trifluoromethanesulfonate (*rac*-1c)

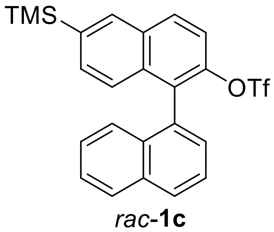

rac-**1c**
$C_{24}H_{21}F_3O_3SSi$
M = 474.57 g/mol

Prepared according to **GP 1** from 6-(trimethylsilyl)-[1,1'-binaphthalen]-2-ol (**S1c**, 1.17 g, 3.42 mmol, 1.00 equiv), DIPEA (1.48 mL, 8.55 mmol, 2.50 equiv) and Tf_2O (0.69 mL, 1.2 mmol, 1.2 equiv) in CH_2Cl_2 (16 mL). Flash column chromatography on silica gel using *n*-pentane and CH_2Cl_2 as the eluent (40:1→20:1) afforded substrate *rac*-**1c** as a pale yellow oil (1.42 g, 88% yield).

R_f = 0.45 (*n*-hexane/CH_2Cl_2=10:1). **^1H NMR** (400 MHz, $CDCl_3$, 298 K): *δ* = 8.15 (s, 1H), 8.10 – 8.00 (m, 2H), 7.97 (d, *J* = 8.3 Hz, 1H), 7.64 (t, *J* = 7.7 Hz, 1H), 7.58 (d, *J* = 9.1 Hz, 1H), 7.55 – 7.46 (m, 3H), 7.39 – 7.30 (m, 2H), 7.25 (d, *J* = 8.4 Hz, 1H), 0.35 (s, 9H). **$^{13}C\{^1H\}$ NMR** (101 MHz, $CDCl_3$, 298 K): *δ* = 145.3, 139.9, 134.1, 134.0, 133.7, 132.6, 132.0, 131.7, 130.8, 130.7, 129.4, 129.2, 128.5, 127.0, 126.6, 126.2, 126.1, 125.8, 125.3, 119.6, 118.3 (q, *J* = 321.2 Hz), −1.1. **^{19}F NMR** (471 MHz, $CDCl_3$, 298 K) *δ* = −74.5. For **HRMS** and **IR** data, see those reported for the enantioriched compound (*R*)-**1c**.

3-methyl-[1,1'-binaphthalen]-2-yl trifluoromethanesulfonate (*rac*-1d)

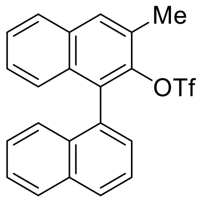

rac-**1d**
$C_{22}H_{15}F_3O_3S$
M = 416.41 g/mol

Prepared according to **GP 1** from 3-methyl-[1,1'-binaphthalen]-2-ol (**S1d**, 1.16 g, 4.07 mmol, 1.00 equiv), DIPEA (1.77 mL, 10.2 mmol, 2.50 equiv) and Tf_2O (0.82 mL, 4.9 mmol, 1.2

equiv) in CH_2Cl_2 (20 mL). Flash column chromatography on silica gel using *n*-pentane and CH_2Cl_2 as the eluent (40:1→20:1) afforded substrate *rac*-**1d** as a white solid (972 mg, 57% yield).

R*f* = 0.45 (*n*-hexane/CH_2Cl_2=10:1). **^{1}H NMR** (500 MHz, $CDCl_3$, 298 K): *δ* = 8.02–7.88 (m, 2H), 7.88–7.77 (m, 2H), 7.62–7.55 (m, 1H), 7.54–7.39 (m, 3H), 7.33–7.17 (m, 4H), 2.65 (s, 3H). **^{13}C{^{1}H} NMR** (126 MHz, $CDCl_3$, 298 K): *δ* = 145.1, 133.8, 132.8, 131.5, 131.2, 131.0, 129.7, 129.4, 129.3, 128.4, 127.4, 127.2, 127.1, 126.7, 126.5, 126.1, 125.9, 125.3, 118.1 (q, *J* = 321.2 Hz), 18.3. **^{19}F NMR** (471 MHz, $CDCl_3$, 298 K) *δ* = −73.9. For **HRMS** and **IR** data, see those reported for the enantioriched compound (*R*)-**1d**.

3-fluoro-[1,1'-binaphthalen]-2-yl trifluoromethanesulfonate (*rac*-1e)

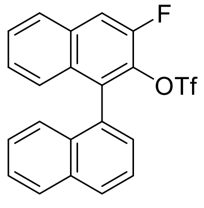

rac-**1e**
$C_{21}H_{12}F_4O_3S$
M = 420.38 g/mol

Prepared according to **GP 1** from 3-fluoro-[1,1'-binaphthalen]-2-ol (**S1e**, 971 mg, 3.37 mmol, 1.00 equiv), DIPEA (1.46 mL, 8.43 mmol, 2.50 equiv) and Tf_2O (0.68 mL, 4.0 mmol, 1.2 equiv) in CH_2Cl_2 (20 mL). Flash column chromatography on silica gel using *n*-pentane and CH_2Cl_2 as the eluent (40:1→20:1) afforded substrate *rac*-**1e** as a pale yellow oil (1.16 g, 82% yield).

R*f* = 0.40 (*n*-hexane/CH_2Cl_2=10:1). **^{1}H NMR** (500 MHz, $CDCl_3$, 298 K): *δ* = 8.09–8.04 (m, 1H), 8.01–7.96 (m, 1H), 7.94–7.89 (m, 1H), 7.84–7.77 (m, 1H), 7.69–7.63 (m, 1H), 7.61–7.49 (m, 3H), 7.39–7.30 (m, 3H), 7.28–7.22 (m, 1H). **^{13}C{^{1}H} NMR** (126 MHz, $CDCl_3$, 298 K): *δ* = 151.7 (d, *J* = 252.8 Hz), 134.5 (d, *J* = 16.7 Hz), 134.3, 133.8, 132.7 (d, *J* = 8.4 Hz), 132.4, 130.5, 129.8, 129,7, 129.4, 128.6, 128.2, 127.7 (d, *J* = 5.4 Hz), 127.4, 126.9, 126.4, 125.6, 125.3, 118.3 (q, *J* = 321.5 Hz), 113.5 (d, *J* = 18.1 Hz). **^{19}F NMR** (471 MHz, $CDCl_3$, 298 K) *δ* = −73.6 (d, *J* = 11.3 Hz, 3F), −127.6–−127.8 (m, 1F). For **HRMS** and **IR** data, see those reported for the enantioriched compound (*R*)-**1e**.

1-(phenanthren-9-yl)naphthalen-2-yl trifluoromethanesulfonate (*rac*-1f)

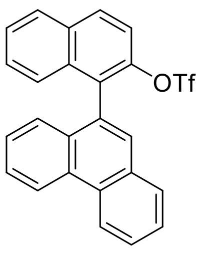

rac-**1f**
$C_{25}H_{15}F_3O_3S$
M = 452.45 g/mol

Prepared according to **GP 1** from 1-(phenanthren-9-yl)naphthalen-2-ol (**S1f**, 800 mg, 2.50 mmol, 1.00 equiv), DIPEA (1.09 mL, 6.25 mmol, 2.50 equiv) and Tf_2O (0.50 mL, 3.0 mmol, 1.2 equiv) in CH_2Cl_2 (15 mL). Flash column chromatography on silica gel using *n*-pentane and CH_2Cl_2 as the eluent (20:1→5:1) afforded substrate *rac*-**1f** as a white solid (858 mg, 76% yield).

$\mathbf{R_f}$ = 0.30 (*n*-hexane/CH_2Cl_2=10:1). **^{1}H NMR** (500 MHz, $CDCl_3$, 298 K): δ = 8.89–8.79 (m, 2H), 8.09 (d, *J* = 9.1 Hz, 1H), 8.01 (d, *J* = 8.2 Hz, 1H), 7.98–7.92 (m, 1H), 7.85 (s, 1H), 7.81–7.74 (m, 1H), 7.72–7.65 (m, 2H), 7.65–7.60 (m, 1H), 7.60–7.54 (m, 1H), 7.49–7.40 (m, 2H), 7.40–7.34 (m, 1H), 7.32–7.27 (m, 1H). **^{13}C{^{1}H} NMR** (126 MHz, $CDCl_3$, 298 K): δ = 145.3, 134.1, 132.7, 131.4, 131.3, 130.84, 130.81, 130.7, 130.6, 130.4, 129.5, 129.1, 128.3, 127.8, 127.5, 127.24, 127.22, 127.16, 126.98, 126.7, 123.1, 122.9, 119.7, 118.4 (q, *J* = 323.8 Hz). **^{19}F NMR** (471 MHz, $CDCl_3$, 298 K) δ = −74.4. For **HRMS** and **IR** data, see those reported for the enantioriched compound (*R*)-**1f**.

1-(2-methoxyphenyl)naphthalen-2-yl trifluoromethanesulfonate (*rac*-1g)

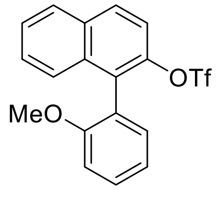

rac-**1g**
$C_{18}H_{13}F_3O_4S$
M = 382.35 g/mol

Prepared according to **GP 1** from 1-(2-methoxyphenyl)naphthalen-2-ol (**S1g**, 470 mg, 1.88 mmol, 1.00 equiv), DIPEA (0.82 mL, 4.7 mmol, 2.5 equiv) and Tf_2O (0.38 mL, 2.3 mmol, 1.2 equiv) in CH_2Cl_2 (7 mL). Flash column chromatography on silica gel using *n*-pentane and CH_2Cl_2 as the eluent (10:1→5:1) afforded substrate *rac*-**1g** as a white solid (702 mg, 98% yield).

$\mathbf{R_f}$ = 0.30 (*n*-hexane/CH_2Cl_2=5:1). **^{1}H NMR** (500 MHz, $CDCl_3$, 298 K): δ = 7.97–7.89 (m, 2H), 7.63–7.58 (m, 1H), 7.58–7.43 (m, 4H), 7.31–7.24 (m, 1H), 7.16–7.06 (m, 2H), 3.72 (s,

3H). **$^{13}C\{^{1}H\}$ NMR** (126 MHz, $CDCl_3$, 298 K): δ = 157.6, 145.0, 133.5, 132.7, 132.5, 130.5, 130.0, 129.3, 128.2, 127.3, 126.9, 126.8, 121.9, 120.6, 119.4, 118.6 (q, J = 320.7 Hz), 111.3, 55.6. **^{19}F NMR** (471 MHz, $CDCl_3$, 298 K) δ = −74.6. For **HRMS** and **IR** data, see those reported for the enantioriched compound (*R*)-**1g**.

1-(2-ethylphenyl)naphthalen-2-yl trifluoromethanesulfonate (*rac*-1h)

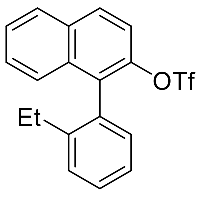

rac-**1h**
$C_{19}H_{15}F_3O_3S$
M = 380.38 g/mol

Prepared according to **GP 1** from 1-(2-ethylphenyl)naphthalen-2-ol (**S1h**, 1.11 g, 4.48 mmol, 1.00 equiv), DIPEA (1.95 mL, 11.2 mmol, 2.50 equiv) and Tf_2O (0.90 mL, 5.4 mmol, 1.2 equiv) in CH_2Cl_2 (20 mL). Flash column chromatography on silica gel using *n*-pentane and CH_2Cl_2 as the eluent (40:1→20:1) afforded substrate *rac*-**1h** as a pale yellow oil (1.37 g, 80% yield).

R_f = 0.40 (*n*-hexane/CH_2Cl_2=10:1). **^{1}H NMR** (500 MHz, $CDCl_3$, 298 K): δ = 8.05–7.92 (m, 2H), 7.64–7.58 (m, 1H), 7.58–7.45 (m, 5H), 7.43–7.36 (m, 1H), 7.33–7.25 (m, 1H), 2.47–2.26 (m, 2H), 1.06 (t, J = 7.5 Hz, 3H). **$^{13}C\{^{1}H\}$ NMR** (126 MHz, $CDCl_3$, 298 K): δ = 144.3, 143.3, 133.7, 132.6, 132.1, 132.0, 131.2, 130.1, 129.2, 128.4, 128.3, 127.6, 127.1, 127.0, 125.8, 119.4, 118.5 (q, J = 320.7 Hz), 26.2, 14.6. **^{19}F NMR** (471 MHz, $CDCl_3$, 298 K) δ = −74.5. For **HRMS** and **IR** data, see those reported for the enantioriched compound (*R*)-**1h**.

1-(o-tolyl)naphthalen-2-yl trifluoromethanesulfonate (*rac*-1i)

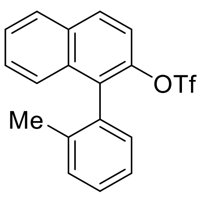

rac-**1i**
$C_{18}H_{13}F_3O_3S$
M = 366.35 g/mol

Prepared according to **GP 1** from 1-(o-tolyl)naphthalen-2-ol (**S1i**, 936 mg, 4.00 mmol, 1.00 equiv), DIPEA (1.74 mL, 10.0 mmol, 2.50 equiv) and Tf_2O (0.81 mL, 4.8 mmol, 1.2 equiv) in CH_2Cl_2 (20 mL). Flash column chromatography on silica gel using *n*-pentane and CH_2Cl_2 as the eluent (40:1→20:1) afforded substrate *rac*-**1i** as a pale yellow oil (1.19 g, 81% yield).

R_f = 0.40 (*n*-hexane/CH_2Cl_2=10:1). **^{1}H NMR** (500 MHz, $CDCl_3$, 298 K): δ = 8.02–7.93 (m,

2H), 7.61–7.55 (m, 1H), 7.53–7.45 (m, 3H), 7.45–7.32 (m, 3H), 7.30–7.25 (m, 1H), 2.03 (s, 3H). **^{13}C{^{1}H} NMR** (126 MHz, $CDCl_3$, 298 K): *δ* = 144.3, 137.6, 133.2, 132.7, 132.6, 132.2, 131.1, 130.4, 130.1, 129.0, 128.3, 127.7, 127.1, 126.8, 125.9, 119.6, 118.5 (q, *J* = 321.0 Hz), 19.8. **^{19}F NMR** (471 MHz, $CDCl_3$, 298 K) *δ* = −74.5. For **HRMS** and **IR** data, see those reported for the enantioriched compound (*R*)-**1i**.

1-(4-fluoro-2-methylphenyl)naphthalen-2-yl trifluoromethanesulfonate (*rac*-1j)

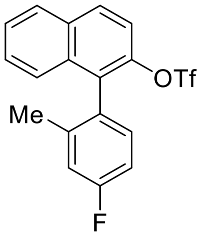

rac-**1j**
$C_{18}H_{12}F_4O_3S$
M = 384.34 g/mol

Prepared according to **GP 1** from 1-(4-fluoro-2-methylphenyl)naphthalen-2-ol (**S1j**, 750 mg, 2.97 mmol, 1.00 equiv), DIPEA (1.29 mL, 7.43 mmol, 2.50 equiv) and Tf_2O (0.60 mL, 3.6 mmol, 1.2 equiv) in CH_2Cl_2 (15 mL). Flash column chromatography on silica gel using *n*-pentane and CH_2Cl_2 as the eluent (40:1→20:1) afforded substrate *rac*-**1j** as a pale yellow solid (950 mg, 83% yield).

R_f = 0.40 (*n*-hexane/CH_2Cl_2=10:1). **^{1}H NMR** (500 MHz, $CDCl_3$, 298 K): *δ* = 8.02–7.91 (m, 2H), 7.62–7.54 (m, 1H), 7.53–7.46 (m, 2H), 7.46–7.41 (m, 1H), 7.26–7.20 (m, 1H), 7.12 (dd, *J* = 9.6, 2.7 Hz, 1H), 7.06 (td, *J* = 8.4, 2.7 Hz, 1H), 2.01 (s, 3H). **^{13}C{^{1}H} NMR** (126 MHz, $CDCl_3$, 298 K): *δ* = 163.2 (d, *J* = 247.8 Hz), 144.5, 140.4 (d, *J* = 8.1 Hz), 133.3, 132.8, 132.7, 131.2, 130.4, 128.5, 128.5, 127.9, 127.2, 126.5, 119.6, 118.5 (q, *J* = 320.6 Hz), 117.2 (d, *J* = 21.3 Hz), 113.0 (d, *J* = 21.6 Hz), 20.0. **^{19}F NMR** (471 MHz, $CDCl_3$, 298 K) *δ* = −74.4, −113.6–−113.8 (m). For **HRMS** and **IR** data, see those reported for the enantioriched compound (*R*)-**1j**.

1-(2-chlorophenyl)naphthalen-2-yl trifluoromethanesulfonate (*rac*-1k)

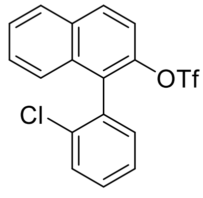

rac-**1k**
$C_{17}H_{10}ClF_3O_3S$
M = 386.77 g/mol

Prepared according to **GP 1** from 1-(2-chlorophenyl)naphthalen-2-ol (**S1k**, 724 mg, 2.85 mmol, 1.00 equiv), DIPEA (1.24 mL, 7.13 mmol, 2.50 equiv) and Tf_2O (0.59 mL, 3.5 mmol, 1.2 equiv) in CH_2Cl_2 (15 mL). Flash column chromatography on silica gel using *n*-pentane and CH_2Cl_2 as the eluent (40:1→20:1) afforded substrate *rac*-**1k** as a pale yellow oil (816 mg, 74% yield).

R*f* = 0.30 (*n*-hexane/CH_2Cl_2=10:1). **^{1}H NMR** (500 MHz, $CDCl_3$, 298 K): δ = 8.01 (d, *J* = 9.1 Hz, 1H), 7.96 (d, *J* = 8.2 Hz, 1H), 7.63–7.55 (m, 2H), 7.55–7.36 (m, 6H). **^{13}C{^{1}H} NMR** (126 MHz, $CDCl_3$, 298 K): δ = 144.4, 134.8, 132.9, 132.8, 132.6, 132.3, 130.9, 130.4, 130.0, 129.8, 128.4, 127.9, 127.2, 126.9, 126.4, 119.4, 118.5 (q, *J* = 320.6 Hz). **^{19}F NMR** (471 MHz, $CDCl_3$, 298 K) δ = −74.3. For **HRMS** and **IR** data, see those reported for the enantioriched compound (*R*)-**1k**.

1-([1,1'-biphenyl]-2-yl)naphthalen-2-yl trifluoromethanesulfonate (*rac*-1l)

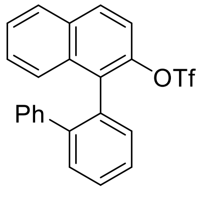

rac-**1l**
$C_{23}H_{15}F_3O_3S$
M = 428.43 g/mol

Prepared according to **GP 1** from 1-([1,1'-biphenyl]-2-yl)naphthalen-2-ol (**S1l**, 1.79 g, 6.05 mmol, 1.00 equiv), DIPEA (2.63 mL, 15.1 mmol, 2.50 equiv) and Tf_2O (1.22 mL, 7.26 mmol, 1.20 equiv) in CH_2Cl_2 (30 mL). Flash column chromatography on silica gel using *n*-pentane and CH_2Cl_2 as the eluent (40:1→20:1) afforded substrate *rac*-**1l** as a pale yellow solid (1.69 g, 66% yield).

R*f* = 0.40 (*n*-hexane/CH_2Cl_2=10:1). **^{1}H NMR** (500 MHz, $CDCl_3$, 298 K): δ = 7.91 (d, *J* = 7.9 Hz, 1H), 7.88–7.78 (m, 2H), 7.68–7.50 (m, 5H), 7.50–7.40 (m, 1H), 7.29–7.21 (m, 1H), 7.17–6.95 (m, 5H). **^{13}C{^{1}H} NMR** (126 MHz, $CDCl_3$, 298 K): δ = 144.0, 143.1, 140.9, 133.9, 132.5, 132.4, 132.0, 131.4, 130.5, 130.0, 129.3, 128.8, 128.3, 127.8, 127.7, 127.2, 127.0, 126.9, 126.9, 119.3, 118.4 (q, *J* = 320.8 Hz). **^{19}F NMR** (471 MHz, $CDCl_3$, 298 K) δ = −74.6. For **HRMS** and **IR** data, see those reported for the enantioriched compound (*R*)-**1l**.

3-fluoro-2-(naphthalen-1-yl)phenyl trifluoromethanesulfonate (*rac*-1m)

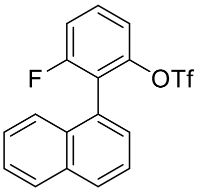

rac-**1m**
$C_{17}H_{10}F_4O_3S$
M = 370.32 g/mol

Prepared according to **GP 1** from 3-fluoro-2-(naphthalen-1-yl)phenol (**S1m**, 1.41 g, 5.91 mmol, 1.00 equiv), DIPEA (2.57 mL, 14.8 mmol, 2.50 equiv) and Tf_2O (1.19 mL, 7.08 mmol, 1.20 equiv) in CH_2Cl_2 (30 mL). Flash column chromatography on silica gel using *n*-pentane and CH_2Cl_2 as the eluent (40:1→20:1) afforded substrate *rac*-**1m** as a colorless oil (1.92 g, 88% yield).

R*f* = 0.35 (*n*-hexane/CH_2Cl_2=10:1). **^{1}H NMR** (500 MHz, $CDCl_3$, 298 K): δ = 8.01 (d, *J* = 8.3 Hz, 1H), 7.96 (d, *J* = 8.2 Hz, 1H), 7.65–7.58 (m, 1H), 7.58–7.44 (m, 5H), 7.37–7.28 (m, 2H). **^{13}C{^{1}H} NMR** (126 MHz, $CDCl_3$, 298 K): *δ* = 161.0 (d, *J* = 250.9 Hz), 148.1 (d, *J* = 6.3 Hz), 133.7, 131.9, 130.1(d, *J* = 9.4 Hz), 129.9, 129.1, 128.6, 126.8, 126.4, 126.3, 125.2, 125.1, 123.3 (d, *J* = 21.4 Hz), 118.3 (q, *J* = 321.1 Hz), 117.8 (d, *J* = 3.1 Hz), 116.0 (d, *J* = 22.8 Hz). **^{19}F NMR** (471 MHz, $CDCl_3$, 298 K) *δ* = −74.2, −108.0–−108.2 (m). **IR** (ATR): ṽ = 3060, 1619, 1577, 1458, 1422, 1199, 1137, 982, 849, 799, 737 cm^{-1}. **HRMS** (APCI): calculated for $C_{17}H_{10}F_4O_3S^+$ $[M]^+$: 370.0281; Found 370.0286.

3-methoxy-2-(naphthalen-1-yl)phenyl trifluoromethanesulfonate (*rac*-1n)

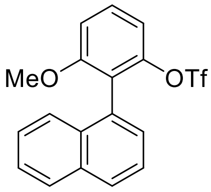

rac-**1n**
$C_{18}H_{13}F_3O_4S$
M = 382.35 g/mol

Prepared according to **GP 1** from 3-methoxy-2-(naphthalen-1-yl)phenol (**S1v**, 1.22 g, 4.88 mmol, 1.00 equiv), DIPEA (2.12 mL, 12.2 mmol, 2.50 equiv) and Tf_2O (0.99 mL, 5.9 mmol, 1.2 equiv) in CH_2Cl_2 (25 mL). Flash column chromatography on silica gel using *n*-pentane and CH_2Cl_2 as the eluent (20:1→10:1) afforded substrate *rac*-**1v** as a white solid (943 mg, 51% yield).

R*f* = 0.35 (*n*-hexane/CH_2Cl_2=5:1). **M.p.**: 52–56 °C **^{1}H NMR** (500 MHz, $CDCl_3$, 298 K): *δ* = 8.01–7.84 (m, 2H), 7.63–7.56 (m, 1H), 7.54–7.35 (m, 5H), 7.17–7.04 (m, 2H), 3.70 (s, 3H). **^{13}C{^{1}H} NMR** (126 MHz, $CDCl_3$, 298 K): *δ* = 159.2, 148.4, 133.6, 132.2, 129.9, 129.2, 129.0,

128.8, 128.4, 126.2, 125.9, 125.5, 125.2, 123.5, 118.3 (q, *J* = 321.0 Hz), 113.7, 110.9, 56.3. **^{19}F NMR** (471 MHz, $CDCl_3$, 298 K) *δ* = −74.5. **IR** (ATR): ṽ = 3058, 1609, 1464, 1267, 1198, 1138, 1057, 935, 777, 738 cm^{-1}. **HRMS** (APCI): calculated for $C_{18}H_{13}F_3O_4S^+$ $[M]^+$: 382.0481; Found 382.0486.

4.2 Characterization Data of Enantioenriched Products 1 and 3

(*S*)-1-([1,1'-binaphthalen]-2-yl)-2-phenyldiazene [(*S*)-3aa] and (*R*)-[1,1'-binaphthalen]-2-yl trifluoromethanesulfonate [(*R*)-1a]

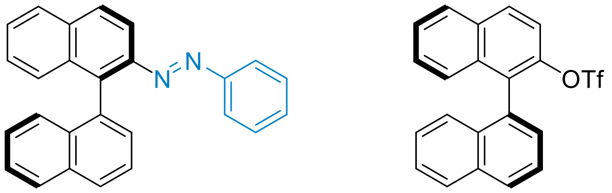

(*S*)-**3aa**
$C_{26}H_{18}N_2$
M = 358.44 g/mol

(*R*)-**1a**
$C_{21}H_{13}F_3O_3S$
M = 402.39 g/mol

Prepared according to **GP 2** from *rac*-**1a** (40.2 mg, 0.100 mmol, 1.00 equiv) and (*E*)-1-phenyl-2-(trimethylsilyl)diazene **2a** (21.4 mg, 0.120 mmol, 1.20 equiv), $Pd(OAc)_2$ (1.1 mg, 5 mol %), **L5** (3.0 mg, 6 mol %), K_2CO_3 (13.8 mg, 0.100 mmol, 1.00 equiv). The reaction was stirred for 72 h at 25 °C. Flash column chromatography on silica gel using *n*-pentane and CH_2Cl_2 as the eluent (100:1→50:1→20:1) afforded (*S*)-**3aa** (13.2 mg, 37% yield, 89% ee) as an orange oil and (*R*)-**1a** (16.3 mg, 41% yield, 77% ee) as a white solid.

Analytical data for (*S*)-**3aa**

R*f* = 0.30 (*n*-hexane/CH_2Cl_2 = 10/1). **^{1}H NMR** (500 MHz, $CDCl_3$, 298 K) δ = 8.15 (d, *J* = 8.9 Hz, 1H), 8.06–7.94 (m, 4H), 7.66–7.60 (m, 1H), 7.59–7.50 (m, 3H), 7.48–7.42 (m, 1H), 7.40–7.31 (m, 4H), 7.32–7.22 (m, 4H). **^{13}C{^{1}H} NMR** (126 MHz, $CDCl_3$, 298 K) δ = 153.0, 148.0, 139.9, 135.2, 135.0, 134.0, 133.8, 133.4, 130.7, 129.2, 129.0, 128.3, 128.2, 128.0, 127.8, 127.6, 127.0, 126.8, 126.1, 125.7, 125.1, 123.0, 114.7. **HRMS** (ESI): calculated for $C_{26}H_{19}N_2^+$ $[(M+H)^+]$: 359.1543; found: 359.1543. **IR** (ATR): ṽ = 3057, 2924, 2852, 1505, 1218, 1150, 1018, 823, 778, 773, 691 cm^{-1}. **Optical Rotation**: $[\alpha]_D^{20}$ = 357.3 (*c* 0.4, $CHCl_3$, 89% ee). The enantiomeric excess of **3aa** was determined by HPLC analysis on a chiral stationary phase (Daicel Chiralcel OD-H column, column temperature 20 °C, solvent *n*-heptane:*i*PrOH = 99:1, flow rate 0.6 mL/min, λ = 280 nm): t_R = 10.5 min (minor), t_S = 11.7 min (major).

Analytical data for (*R*)-**1a**

R*f* = 0.40 (*n*-hexane/CH_2Cl_2 = 10/1). **M.p.**: 72–75 °C. **HRMS** (APCI): calculated for

$C_{21}H_{14}F_3O_3S^+$ [(M+H)$^+$]: 403.0610; found: 403.0606. **IR** (ATR): ṽ = 3059, 2925, 1971, 1507, 1420, 1211, 1140, 956, 833, 773 cm^{-1}. **Optical Rotation**: $[\alpha]_D^{20}$ = 24.4 (*c* 1.0, $CHCl_3$, 73% ee). The enantiomeric excess of **1a** was determined by HPLC analysis on a chiral stationary phase (Daicel Chiralcel IB column, column temperature 20 °C, solvent *n*-heptane:*i*PrOH = 99.8:0.2, flow rate 0.6 mL/min, λ = 280 nm): t_R = 10.5 min (major), t_S = 12.1 min (minor). The NMR data are in accordance with those reported for the racemic compound *rac*-**1a**.

(*S*)-1-([1,1'-binaphthalen]-2-yl)-2-(4-fluorophenyl)diazene [(*S*)-3ab] and (*R*)-[1,1'-binaphthalen]-2-yl trifluoromethanesulfonate [(*R*)-1a]

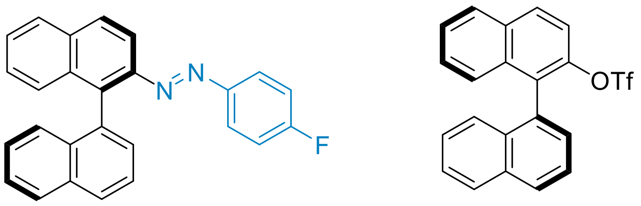

(*S*)-**3ab**
$C_{26}H_{17}FN_2$
M = 376.43 g/mol

(*R*)-**1a**
$C_{21}H_{13}F_3O_3S$
M = 402.39 g/mol

Prepared according to **GP 2** from *rac*-**1a** (40.2 mg, 0.100 mmol, 1.00 equiv) and (*E*)-1-(4-fluorophenyl)-2-(trimethylsilyl)diazene **2b** (23.5 mg, 0.120 mmol, 1.20 equiv), $Pd(OAc)_2$ (2.2 mg, 10 mol %), **L5** (6.0 mg, 12 mol %), K_2CO_3 (13.8 mg, 0.100 mmol, 1.00 equiv). The reaction was stirred for 72 h at 25 °C. Flash column chromatography on silica gel using *n*-pentane and CH_2Cl_2 as the eluent (100:1→50:1→20:1) afforded (*S*)-**3ab** (17.7 mg, 47% yield, 91% ee; 48% recrystallization yield, 95% ee) as an orange solid and (*R*)-**1a** (17.5 mg, 44% yield, 81% ee) as a white solid.

Analytical data for (*S*)-**3ab**

R_f = 0.30 (*n*-hexane/CH_2Cl_2 = 10/1). **M.p.**: 102–105 °C. **^{1}H NMR** (500 MHz, $CDCl_3$, 298 K) δ = 8.13 (d, *J* = 8.7 Hz, 1H), 8.06–7.94 (m, 4H), 7.67–7.59 (m, 1H), 7.58–7.49 (m, 3H), 7.48–7.42 (m, 1H), 7.40–7.30 (m, 4H), 7.26–7.21 (m, 1H), 6.99–6.90 (m, 2H). **^{13}C{^{1}H} NMR** (126 MHz, $CDCl_3$, 298 K) *δ* = 164.2 (d, *J* = 253 Hz), 149.6 (d, *J* = 3.2 Hz), 147.8, 139.9, 135.2, 135.0, 134.0, 133.8, 133.4, 129.2 (d, *J* = 5.7 Hz), 128.3, 128.2, 128.0, 127.8, 127.6, 127.0, 126.9, 126.1, 125.8, 125.1, 125.0, 124.9, 115.9 (d, *J* = 23.0 Hz), 114.6. **^{19}F NMR** (471 MHz, $CDCl_3$, 298 K) δ = −109.9. **HRMS** (ESI): calculated for $C_{26}H_{18}FN_2^+$ [(M+H)$^+$]: 377.1449; found: 377.1450. **IR** (ATR): ṽ = 3058, 1592, 1496, 1228, 1137, 843, 801, 779 cm^{-1}. **Optical Rotation**: $[\alpha]_D^{20}$ = 542.5 (*c* 1.0, $CHCl_3$, 91% ee). The enantiomeric excess of **3ab** was determined by HPLC analysis on a chiral stationary phase (Daicel Chiralcel OD-H column, column temperature 20 °C, solvent *n*-heptane:*i*PrOH = 99:1, flow rate 0.6 mL/min): t_R = 9.6 min (minor), t_S = 10.4 min (major).

Analytical data for (*R*)-**1a**

The enantiomeric excess of **1a** (81% ee) was determined by HPLC analysis on a chiral stationary phase (Daicel Chiralcel IB column, column temperature 20 °C, solvent *n*-heptane:*i*PrOH = 99.8:0.2, flow rate 0.6 mL/min, λ = 280 nm): t_R = 12.2 min (major), t_S = 13.9 min (minor). The NMR data are in accordance with those reported for the racemic compound *rac*-**1a**.

(*S*)-1-([1,1'-binaphthalen]-2-yl)-2-(4-chlorophenyl)diazene [(*S*)-3ac] and (*R*)-[1,1'-binaphthalen]-2-yl trifluoromethanesulfonate [(*R*)-1a]

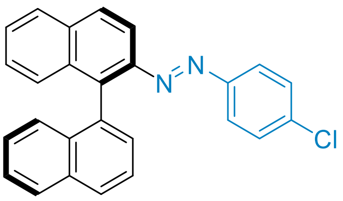

(*S*)-**3ac**
$C_{26}H_{17}ClN_2$
M = 392.89 g/mol

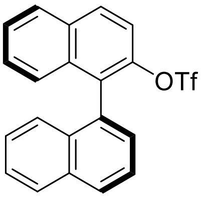

(*R*)-**1a**
$C_{21}H_{13}F_3O_3S$
M = 402.39 g/mol

Prepared according to **GP 2** from *rac*-**1a** (40.2 mg, 0.100 mmol, 1.00 equiv) and (*E*)-1-(4-chlorophenyl)-2-(trimethylsilyl)diazene **2c** (25.4 mg, 0.12 mmol, 1.2 equiv), $Pd(OAc)_2$ (2.2 mg, 10 mol %), **L5** (6.0 mg, 12 mol %), K_2CO_3 (13.8 mg, 0.100 mmol, 1.00 equiv). The reaction was stirred for 72 h at 25 °C. Flash column chromatography on silica gel using *n*-pentane and CH_2Cl_2 as the eluent (100:1→50:1→20:1) afforded (*S*)-**3ac** (15.6 mg, 40% yield, 91% ee) as an orange oil and (*R*)-**1a** (12.7 mg, 31% yield, 82% ee) as a white solid.

Analytical data for (*S*)-**3ac**

R_f = 0.25 (*n*-hexane/CH_2Cl_2 = 10/1) **^{1}H NMR** (500 MHz, $CDCl_3$, 298 K) δ = 8.14 (d, J = 9.0 Hz, 1H), 8.06–7.96 (m, 4H), 7.67–7.62 (m, 1H), 7.61–7.52 (m, 3H), 7.49–7.44 (m, 1H), 7.40–7.21 (m, 7H). **^{13}C{^{1}H} NMR** (126 MHz, $CDCl_3$, 298 K) δ = 151.4, 147.8, 140.3, 136.6, 135.11, 135.07, 134.0, 133.8, 133.4, 129.23, 129.15, 128.3, 128.2, 128.1, 127.9, 127.8, 126.9, 126.2, 125.8, 125.1, 124.2, 114.5. **HRMS** (ESI): calculated for $C_{26}H_{18}ClN_2^+$ [(M+H)$^+$]: 393.1153; found: 393.1151. **IR** (ATR): ṽ = 3056, 2923, 1588, 1478, 1420, 1213, 1144, 1087, 1009, 835, 777, 773 cm^{-1}. **Optical Rotation**: $[\alpha]_D^{20}$ = 615.6 (*c* 1.0, $CHCl_3$, 91% ee). The enantiomeric excess of **3c** was determined by HPLC analysis on a chiral stationary phase (Daicel Chiralcel OD-H column, column temperature 20 °C, solvent *n*-heptane:*i*PrOH = 99.8:0.2, flow rate 0.6 mL/min, λ = 280 nm): t_R = 21.4 min (minor), t_S = 25.2 min (major).

Analytical data for (*R*)-**1a**

The enantiomeric excess of **1a** (82% ee) was determined by HPLC analysis on a chiral stationary phase (Daicel Chiralcel IB column, column temperature 20 °C, solvent *n*-

heptane:*i*PrOH = 99.8:0.2, flow rate 0.6 mL/min, λ = 280 nm): t_R = 12.2 min (major), t_S = 14.4 min (minor). The NMR data are in accordance with those reported for the racemic compound *rac*-**1a**.

(*S*)-1-([1,1'-binaphthalen]-2-yl)-2-(3-fluorophenyl)diazene [(*S*)-3ad] and (*R*)-[1,1'-binaphthalen]-2-yl trifluoromethanesulfonate [(*R*)-1a]

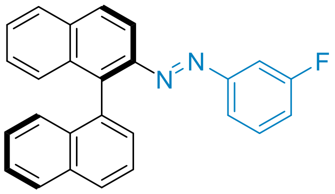

(*S*)-**3ad**
$C_{26}H_{17}FN_2$
M = 376.43 g/mol

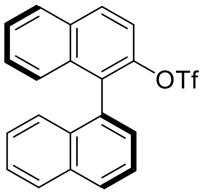

(*R*)-**1a**
$C_{21}H_{13}F_3O_3S$
M = 402.39 g/mol

Prepared according to **GP 2** from *rac*-**1a** (40.2 mg, 0.100 mmol, 1.00 equiv) and (*E*)-1-(3-fluorophenyl)-2-(trimethylsilyl)diazene **2d** (23.5 mg, 0.120 mmol, 1.20 equiv), $Pd(OAc)_2$ (2.2 mg, 10 mol %), **L5** (6.0 mg, 12 mol %), K_2CO_3 (13.8 mg, 0.100 mmol, 1.00 equiv). The reaction was stirred for 72 h at 25 °C. Flash column chromatography on silica gel using *n*-pentane and CH_2Cl_2 as the eluent (100:1→50:1→20:1) afforded (*S*)-**3ad** (13.4 mg, 36% yield, 92% ee; 49% recrystallization yield, 98% ee) as an orange solid and (*R*)-**1a** (20.6 mg, 51% yield, 52% ee) as a white solid.

Analytical data for (*S*)-**3ad**

R*f* = 0.30 (*n*-hexane/CH_2Cl_2 = 10/1). **M.p.**: 109–111 °C. **^{1}H NMR** (500 MHz, $CDCl_3$, 298 K) δ = 8.16–8.09 (m, 1H), 8.05–7.95 (m, 4H), 7.67–7.60 (m, 1H), 7.60–7.49 (m, 3H), 7.49–7.42 (m, 1H), 7.39–7.20 (m, 6H), 7.04–6.95 (m, 1H), 6.89 (d, *J* = 10.1 Hz, 1H). **^{13}C{^{1}H} NMR** (101 MHz, $CDCl_3$, 298 K) δ = 163.1 (d, *J* = 246.3 Hz), 154.5 (d, *J* = 8.2 Hz), 147.7, 140.6, 135.2, 134.9, 134.0, 133.7, 133.4, 130.1 (d, *J* = 8.9 Hz), 129.2, 129.1, 128.3, 128,23, 127.9, 127.9, 126.9, 126.9, 126.2, 125.8, 125.1, 120.5 (d, *J* = 2.9 Hz), 117.4 (d, *J* = 21.9 Hz), 114.5, 108.3 (d, *J* = 22.8 Hz) ppm. **^{19}F NMR** (471 MHz, $CDCl_3$, 298 K) δ = −112.4. **HRMS** (ESI): calculated for $C_{26}H_{18}FN_2^+$ [(M+H)$^+$]: 377.1449; found: 377.1449. **IR** (ATR): ṽ = 3062, 2922, 2159, 1978, 1592, 1476, 1239, 780, 691 cm^{-1}. **Optical Rotation**: $[\alpha]_D^{20}$ = 652.8 (*c* 0.5, $CHCl_3$, 92% ee). The enantiomeric excess of **3ad** was determined by HPLC analysis on a chiral stationary phase (Daicel Chiralcel OD-H column, column temperature 20 °C, solvent *n*-heptane:*i*PrOH = 99:1, flow rate 0.6 mL/min, λ = 280 nm): t_R = 10.3 min (minor), t_S = 11.6 min (major).

Analytical data for (*R*)-**1a**

The enantiomeric excess of **1a** (52% ee) was determined by HPLC analysis on a chiral

stationary phase (Daicel Chiralcel IB column, column temperature 20 °C, solvent *n*-heptane:*i*PrOH = 99.8:0.2, flow rate 0.6 mL/min, λ = 280 nm): t_R = 12.5 min (major), t_S = 14.5 min (minor). The NMR data are in accordance with those reported for the racemic compound *rac*-**1a**.

(*S*)-1-([1,1'-binaphthalen]-2-yl)-2-(p-tolyl)diazene [(*S*)-3ae] and (*R*)-[1,1'-binaphthalen]-2-yl trifluoromethanesulfonate [(*R*)-1a]

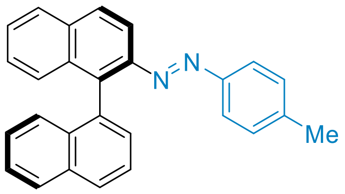

(*S*)-**3ae**
$C_{27}H_{20}N_2$
M = 372.47 g/mol

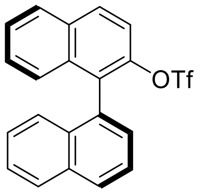

(*R*)-**1a**
$C_{21}H_{13}F_3O_3S$
M = 402.39 g/mol

Prepared according to **GP 2** from *rac*-**1a** (40.2 mg, 0.100 mmol, 1.00 equiv) and (*E*)-1-(p-tolyl)-2-(trimethylsilyl)diazene **2e** (23.0 mg, 0.120 mmol, 1.20 equiv), $Pd(OAc)_2$ (2.2 mg, 10 mol %), **L5** (6.0 mg, 12 mol %), K_2CO_3 (13.8 mg, 0.100 mmol, 1.00 equiv). The reaction was stirred for 72 h at 25 °C. Flash column chromatography on silica gel using *n*-pentane and CH_2Cl_2 as the eluent (100:1→50:1→20:1) afforded (*S*)-**3ae** (15.3 mg, 41% yield, 91% ee) as an orange oil and (*R*)-**1a** (14.7 mg, 37% yield, 74% ee) as a white solid.

Analytical data for (*S*)-**3ae**

R_f = 0.30 (*n*-hexane/CH_2Cl_2 = 10/1) **^{1}H NMR** (500 MHz, $CDCl_3$, 298 K) δ = 8.17–8.08 (m, 1H), 8.05–7.91 (m, 4H), 7.66–7.58 (m, 1H), 7.58–7.48 (m, 3H), 7.48–7.40 (m, 1H), 7.37–7.30 (m, 2H), 7.30–7.19 (m, 3H), 7.11–7.00 (m, 2H), 2.30 (s, 3H). **^{13}C{^{1}H} NMR** (126 MHz, $CDCl_3$, 298 K) δ = 151.2, 148.0, 141.2, 139.4, 135.3, 134.9, 134.0, 133.8, 133.4, 129.6, 129.2, 129.1, 128.3, 128.2, 127.9, 127.7, 127.4, 127.0, 126.8, 126.1, 125.7, 125.1, 123.0, 114.8, 21.5. **HRMS** (ESI): calculated for $C_{27}H_{21}N_2^+$ [(M+H)$^+$]: 373.1699; found: 373.1701. **IR** (ATR): ṽ = 3055, 2920, 2062, 2501, 1151, 828, 800, 779, 750 cm^{-1}. **Optical Rotation**: $[\alpha]_D^{20}$ = 560.8 (*c* 1.0, $CHCl_3$, 91% ee). The enantiomeric excess of **3ae** was determined by HPLC analysis on a chiral stationary phase (Daicel Chiralcel OD-H column, column temperature 20 °C, solvent *n*-heptane:*i*PrOH = 99.8:0.2, flow rate 0.6 mL/min, λ = 280 nm): t_R = 19.6 min (minor), t_S = 24.3 min (major).

Analytical data for (*R*)-**1a**

The enantiomeric excess of **1a** (74% ee) was determined by HPLC analysis on a chiral stationary phase (Daicel Chiralcel IB column, column temperature 20 °C, solvent *n*-heptane:*i*PrOH = 99.8:0.2, flow rate 0.6 mL/min, λ = 280 nm): t_R = 11.7 min (major), t_S =

14.3 min (minor). The NMR data are in accordance with those reported for the racemic compound *rac*-**1a**.

(*S*)-1-([1,1'-binaphthalen]-2-yl)-2-(4-methoxyphenyl)diazene [(*S*)-3af] and (*R*)-[1,1'-binaphthalen]-2-yl trifluoromethanesulfonate [(*R*)-1a]

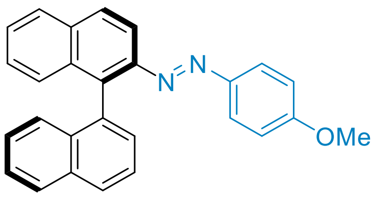

(*S*)-**3af**
$C_{27}H_{20}N_2O$
M = 388.47 g/mol

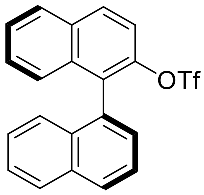

(*R*)-**1a**
$C_{21}H_{13}F_3O_3S$
M = 402.39 g/mol

Prepared according to **GP 2** from *rac*-**1a** (40.2 mg, 0.100 mmol, 1.00 equiv) and (*E*)-1-(4-methoxyphenyl)-2-(trimethylsilyl)diazene **2f** (25.0 mg, 0.120 mmol, 1.20 equiv), $Pd(OAc)_2$ (2.2 mg, 10 mol %), **L5** (6.0 mg, 12 mol %), K_2CO_3 (13.8 mg, 0.100 mmol, 1.00 equiv). The reaction was stirred for 72 h at 25 °C. Flash column chromatography on silica gel using *n*-pentane and CH_2Cl_2 as the eluent (50:1→20:1→10:1) afforded (*S*)-**3af** (12.2 mg, 31% yield, 89% ee) as an orange oil and (*R*)-**1a** (20.1 mg, 50% yield, 65% ee) as a white solid.

Analytical data for (*S*)-**3af**

R_f = 0.25 (*n*-hexane/CH_2Cl_2 = 3/1) **^{1}H NMR** (500 MHz, $CDCl_3$, 298 K) δ = 8.13 (d, *J* = 9.0 Hz, 1H), 8.05–7.93 (m, 4H), 7.65–7.59 (m, 1H), 7.56–7.47 (m, 3H), 7.47–7.41 (m, 1H), 7.40–7.30 (m, 4H), 7.25–7.19 (m, 1H), 6.80–6.74 (m, 2H), 3.78 (s, 3H). **^{13}C{^{1}H} NMR** (126 MHz, $CDCl_3$, 298 K) δ = 161.9, 148.0, 147.5, 139.0, 135.4, 134.7, 134.0, 133.8, 133.4, 129.2, 129.1, 128.3, 128.1, 127.9, 127.7, 127.2, 127.1, 126.7, 126.0, 125.7, 125.1, 124.9, 114.9, 114.1, 55.6. **HRMS** (ESI): calculated for $C_{27}H_{21}N_2O^+$ [(M+H)$^+$]: 389.1648; found: 389.1646. **IR** (ATR): ṽ = 3056, 1599, 1500, 1254, 1144, 1029, 839 cm^{-1}. **Optical Rotation**: $[\alpha]_D^{20}$ = 217.9 (*c* 0.8, $CHCl_3$, 89% ee). The enantiomeric excess of **3af** was determined by HPLC analysis on a chiral stationary phase (Daicel Chiralcel OD-H column, column temperature 20 °C, solvent *n*-heptane:*i*PrOH = 98:2, flow rate 0.6 mL/min, λ = 230 nm): t_R = 12.5 min (minor), t_S = 14.3 min (major).

Analytical data for (*R*)-**1a**

The enantiomeric excess of **1a** (65% ee) was determined by HPLC analysis on a chiral stationary phase (Daicel Chiralcel IB column, column temperature 20 °C, solvent *n*-heptane:*i*PrOH = 99.8:0.2, flow rate 0.6 mL/min, λ = 280 nm): t_R = 10.0 min (major), t_S = 11.3 min (minor). The NMR data are in accordance with those reported for the racemic compound *rac*-**1a**.

Methyl-(*S*)-4-([1,1'-binaphthalen]-2-yldiazenyl)benzoate [(*S*)-3ag] and (*R*)-[1,1'-binaphthalen]-2-yl trifluoromethanesulfonate [(*R*)-1a]

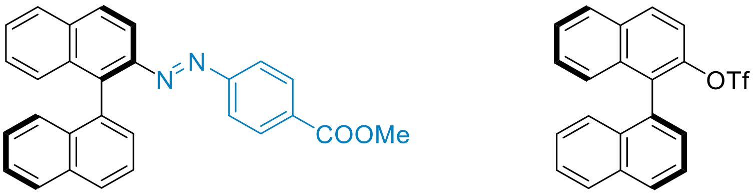

(*S*)-**3ag**
$C_{28}H_{20}N_2O_2$
M = 416.48 g/mol

(*R*)-**1a**
$C_{21}H_{13}F_3O_3S$
M = 402.39 g/mol

Prepared according to **GP 2** from *rac*-**1a** (40.2 mg, 0.100 mmol, 1.00 equiv) and methyl (*E*)-4-((trimethylsilyl)diazenyl)benzoate **2g** (28.3 mg, 0.120 mmol, 1.20 equiv), $Pd(OAc)_2$ (2.2 mg, 10 mol %), **L5** (6.0 mg, 12 mol %), K_2CO_3 (13.8 mg, 0.100 mmol, 1.00 equiv). The reaction was stirred for 85 h at 25 °C. Flash column chromatography on silica gel using *n*-pentane and CH_2Cl_2 as the eluent (50:1→20:1→10:1) afforded (*S*)-**3ag** (6.4 mg, 15% yield, 87% ee; 61% recrystallization yield, 98% ee) as an orange solid and (*R*)-**1a** (28.6 mg, 71% yield, 26% ee) as a pale yellow oil.

Analytical data for (*S*)-**3ag**

R_f = 0.30 (*n*-hexane/CH_2Cl_2 = 5/1). **M.p.**: 122–125 °C. **^{1}H NMR** (500 MHz, $CDCl_3$, 298 K) δ = 8.13 (d, *J* = 9.0 Hz, 1H), 8.04–7.91 (m, 6H), 7.66–7.60 (m, 1H), 7.60–7.51 (m, 3H), 7.48–7.43 (m, 1H), 7.39–7.29 (m, 4H), 7.28–7.22 (m, 1H), 3.88 (s, 3H). **^{13}C{^{1}H} NMR** (126 MHz, $CDCl_3$, 298 K) δ = 166.6, 155.5, 147.9, 141.0, 135.3, 134.9, 133.8, 133.4, 131.5, 130.5, 129.3, 129.2, 128.4, 128.3, 128.3, 128.2, 128.0, 128.0, 127.0, 126.9, 126.2, 125.8, 125.1, 122.8, 114.4, 52.3. **HRMS** (ESI): calculated for $C_{28}H_{21}N_2O_2^+$ [$(M+H)^+$]: 417.1598; found: 417.1595. **IR** (ATR): $\tilde{\nu}$ = 3053, 1721, 1593, 1277, 1111, 822, 775, 699 cm^{-1}. **Optical Rotation**: $[\alpha]_D^{20}$ = 689.8 (*c* 0.5, $CHCl_3$, 87% ee). The enantiomeric excess of **3ag** was determined by HPLC analysis on a chiral stationary phase (Daicel Chiralcel OD-H column, column temperature 20 °C, solvent *n*-heptane:*i*PrOH = 99:1, flow rate 0.6 mL/min, λ = 230 nm): t_R = 18.7 min (minor), t_S = 21.4 min (major).

Analytical data for (*R*)-**1a**

The enantiomeric excess of **1a** (26% ee) was determined by HPLC analysis on a chiral stationary phase (Daicel Chiralcel IB column, column temperature 20 °C, solvent *n*-heptane:*i*PrOH = 99.8:0.2, flow rate 0.6 mL/min, λ = 280 nm): t_R = 12.5 min (major), t_S = 14.8 min (minor). The NMR data are in accordance with those reported for the racemic compound *rac*-**1a**.

(*S*)-1-(4-fluorophenyl)-2-(6-methoxy-[1,1'-binaphthalen]-2-yl)diazene [(*S*)-3bb] and (*R*)-6-methoxy-[1,1'-binaphthalen]-2-yl trifluoromethanesulfonate [(*R*)-1b]

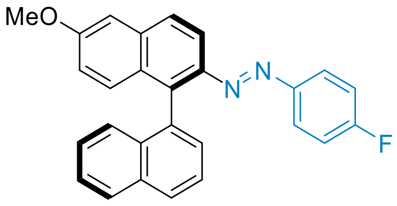

(*S*)-**3bb**
$C_{27}H_{19}FN_2O$
M = 406.46 g/mol

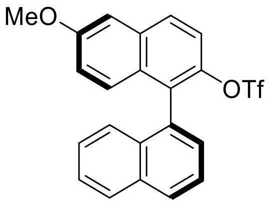

(*R*)-**1b**
$C_{22}H_{15}F_3O_4S$
M = 432.41 g/mol

Prepared according to **GP 2** from *rac*-**1b** (43.2 mg, 0.10 mmol, 1.0 equiv) and (*E*)-1-(4-fluorophenyl)-2-(trimethylsilyl)diazene **2b** (23.5 mg, 0.12 mmol, 1.2 equiv), $Pd(OAc)_2$ (2.2 mg, 10 mol %), **L5** (6.0 mg, 12 mol %), K_2CO_3 (13.8 mg, 0.100 mmol, 1.00 equiv). The reaction was stirred for 72 h at 25 °C. Flash column chromatography on silica gel using *n*-pentane and CH_2Cl_2 as the eluent (50:1→20:1→10:1) afforded (*S*)-**3bb** (11.5 mg, 28% yield, 94% ee) as an orange oil and (*R*)-**1b** (22.8 mg, 53% yield, 65% ee) as a pale yellow solid.

Analytical data for (*S*)-**3bb**

$\mathbf{R}_f$ = 0.25 (*n*-hexane/CH_2Cl_2 = 3/1) **^{1}H NMR** (500 MHz, $CDCl_3$, 298 K) δ = 8.14–8.08 (m, 1H), 8.02–7.94 (m, 2H), 7.92–7.86 (m, 1H), 7.64–7.59 (m, 1H), 7.53–7.48 (m, 1H), 7.48–7.38 (m, 2H), 7.37–7.30 (m, 3H), 7.30–7.21 (m, 2H), 7.03–6.97 (m, 1H), 6.97–6.90 (m, 2H), 3.96 (s, 3H). **^{13}C{^{1}H} NMR** (126 MHz, $CDCl_3$, 298 K) δ = 159.2, 149.6 (d, J = 2.7 Hz), 146.5, 140.2, 136.6, 135.3, 133.7, 133.4, 129.5, 129.11, 129.05, 128.2, 128.0, 127.9, 127.0, 126.1, 125.8, 125.1, 124.8 (d, J = 8.5 Hz), 119.2, 115.9 (d, J = 22.8 Hz), 115.0, 106.7, 55.6. (The C–F carbon was not detected due to low concentration.) **^{19}F NMR** (471 MHz, $CDCl_3$, 298 K) δ = −110.4. **HRMS** (ESI): calculated for $C_{27}H_{20}FN_2O^+$ [(M+H)$^+$]: 407.1554; found: 407.1551. **IR** (ATR): ṽ = 3060, 2931, 1618, 1495, 1418, 1227, 1165, 1031, 843, 798, 688 cm^{-1}. **Optical Rotation**: $[\alpha]_D^{20}$ = 503.5 (*c* 0.8, $CHCl_3$, 94% ee). The enantiomeric excess of **3bb** was determined by HPLC analysis on a chiral stationary phase (Daicel Chiralcel IA column, column temperature 20 °C, solvent *n*-heptane:*i*PrOH = 99:1, flow rate 0.6 mL/min, λ = 230 nm): t_R = 12.4 min (minor), t_S = 13.8 min (major).

Analytical data for (*R*)-**1b**

$\mathbf{R}_f$ = 0.40 (*n*-hexane/CH_2Cl_2 = 3/1). **M.p.**: 70–73 °C. **HRMS** (APCI): calculated for $C_{22}H_{16}F_3O_4S^+$ [(M+H)$^+$]: 433.0716; found: 433.0708. **IR** (ATR): ṽ = 3058, 2934, 1624, 1509, 1419, 1208, 1138, 946, 849, 809, 777, 746, 689 cm^{-1}. **Optical Rotation**: $[\alpha]_D^{20}$ = 33,4 (*c* 1.0, $CHCl_3$, 65% ee). The enantiomeric excess of **1b** was determined by HPLC analysis

on a chiral stationary phase (Daicel Chiralcel IA column, column temperature 20 °C, solvent *n*-heptane:*i*PrOH = 99:1, flow rate 0.6 mL/min, λ = 230 nm): t_R = 12.5 min (minor), t_S = 13.6 min (major). The NMR data are in accordance with those reported for the racemic compound *rac*-**1b**.

(*S*,*E*)-1-phenyl-2-(6-(trimethylsilyl)-[1,1'-binaphthalen]-2-yl)diazene [(*S*)-3ca] and (*R*)-6-(trimethylsilyl)-[1,1'-binaphthalen]-2-yl trifluoromethanesulfonate [(*R*)-1c]

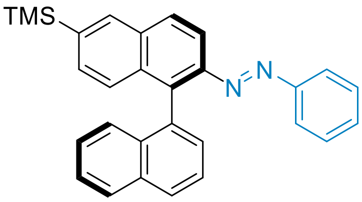

(*S*)-**3ca**
$C_{29}H_{26}N_2Si$
M = 430.63 g/mol

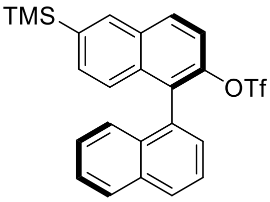

(*R*)-**1c**
$C_{24}H_{21}F_3O_3SSi$
M = 474.57 g/mol

Prepared according to **GP 2** from *rac*-**1c** (47.4 mg, 0.100 mmol, 1.00 equiv) and (*E*)-1-phenyl-2-(trimethylsilyl)diazene **2a** (21.4 mg, 0.120 mmol, 1.20 equiv), $Pd(OAc)_2$ (1.1 mg, 5 mol %), **L5** (3.0 mg, 6 mol %), K_2CO_3 (13.8 mg, 0.100 mmol, 1.00 equiv). The reaction was stirred for 72 h at 25 °C. Flash column chromatography on silica gel using *n*-pentane and CH_2Cl_2 as the eluent (100:1→50:1→20:1) afforded (*S*)-**3ca** (11.4 mg, 27% yield, 90% ee) as an orange oil and (*R*)-**1c** (23.0 mg, 49% yield, 68% ee) as a pale yellow oil.

Analytical data for (*S*)-**3ca**

R_f = 0.30 (*n*-hexane/CH_2Cl_2 = 10/1) **^{1}H NMR** (500 MHz, $CDCl_3$, 298 K) δ = 8.09–8.02 (m, 2H), 7.98–7.86 (m, 3H), 7.57–7.51 (m, 1H), 7.47–7.32 (m, 4H), 7.32–7.24 (m, 2H), 7.24–7.12 (m, 5H), 0.27 (s, 9H). **^{13}C{^{1}H} NMR** (126 MHz, $CDCl_3$, 298 K) δ = 153.0, 148.1, 140.4, 139.8, 135.2, 134.3, 134.2, 134.1, 133.8, 133.4, 130.9, 130.7, 129.3, 129.1, 129.0, 128.2, 128.0, 127.0, 126.6, 126.1, 125.7, 125.1, 123.0, 114.7, −1.0. **HRMS** (ESI): calculated for $C_{29}H_{27}N_2Si^+$ [(M+H)$^+$]: 431.1938; found: 431.1934. **IR** (ATR): ṽ = 3056, 2952, 1589, 1336, 1248, 1100, 1070, 832, 775, 690 cm^{-1}. **Optical Rotation**: $[\alpha]_D^{20}$ = 460.6 (*c* 1.0, $CHCl_3$, 90% ee). The enantiomeric excess of **3ca** was determined by HPLC analysis on a chiral stationary phase (Daicel Chiralcel IB column, column temperature 20 °C, solvent *n*-heptane:*i*PrOH = 99.8:0.2, flow rate 0.6 mL/min, λ = 230 nm): t_R = 9.3 min (minor), t_S = 9.9 min (major).

Analytical data for (*R*)-**1c**

R_f = 0.30 (*n*-hexane/CH_2Cl_2 = 10/1). **HRMS** (APCI): calculated for $C_{24}H_{21}F_3O_3SSi^+$ [M$^+$]: 474.0927; found: 474.0924. **IR** (ATR): ṽ = 3059, 2955, 1590, 1421, 1209, 1138, 958, 886, 845, 828, 775, 689 cm^{-1}. **Optical Rotation**: $[\alpha]_D^{20}$ = 45.6 (*c* 1.0, $CHCl_3$, 68% ee). The

enantiomeric excess of **1c** was determined by HPLC analysis on a chiral stationary phase (Daicel Chiralcel OD-H column, column temperature 20 °C, solvent *n*-heptane:*i*PrOH = 99.9:0.1, flow rate 0.6 mL/min, λ = 280 nm): t_R = 20.9 min (major), t_S = 22.2 min (minor). The NMR data are in accordance with those reported for the racemic compound *rac*-**1c**.

(*S*)-1-(3-methyl-[1,1'-binaphthalen]-2-yl)-2-phenyldiazene [(*S*)-3da] and (*R*)-3-methyl-[1,1'-binaphthalen]-2-yl trifluoromethanesulfonate [(*R*)-1d]

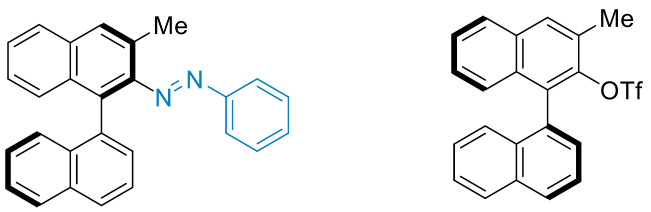

(*S*)-**3da**
$C_{27}H_{20}N_2$
M = 372.47 g/mol

(*R*)-**1d**
$C_{22}H_{15}F_3O_3S$
M = 416.41 g/mol

Prepared according to **GP 2** from *rac*-**1d** (41.6 mg, 0.100 mmol, 1.00 equiv) and (*E*)-1-phenyl-2-(trimethylsilyl)diazene **2a** (21.4 mg, 0.120 mmol, 1.20 equiv), $Pd(OAc)_2$ (1.1 mg, 5 mol %), **L1** (2.8 mg, 6 mol %), K_2CO_3 (13.8 mg, 0.100 mmol, 1.00 equiv). The reaction was stirred for 72 h at 60 °C. Flash column chromatography on silica gel using *n*-pentane and CH_2Cl_2 as the eluent (100:1→50:1→20:1) afforded (*S*)-**3da** (8.5 mg, 23% yield, 97% ee) as an orange oil and (*R*)-**1d** (32.1 mg, 77% yield, 31% ee) as a pale yellow oil.

Analytical data for (*S*)-**3da**

R_f = 0.30 (*n*-hexane/CH_2Cl_2 = 10/1) **^{1}H NMR** (500 MHz, $CDCl_3$, 298 K) δ = 7.92–7.84 (m, 4H), 7.51–7.44 (m, 2H), 7.44–7.39 (m, 1H), 7.39–7.35 (m, 2H), 7.33–7.29 (m, 1H), 7.29–7.21 (m, 5H), 7.15–7.09 (m, 2H), 2.60 (s, 3H). **^{13}C{^{1}H} NMR** (101 MHz, $CDCl_3$, 298 K) δ = 152.9, 150.1, 135.4, 133.6, 133.4, 133.3, 132.7, 130.8, 130.1, 129.9, 129.1, 128.9, 128.9, 128.1, 127.7, 127.4, 127.3, 127.0, 126.5, 126.1, 125.9, 125.7, 125.2, 122.1, 19.8. **HRMS** (ESI): calculated for $C_{27}H_{21}N_2^+$ [(M+H)$^+$]: 373.1699; found: 373.1700. **IR** (ATR): ṽ = 3057, 2923, 2852, 1591, 1495, 1146, 1018, 883, 798, 775, 751, 687 cm^{-1}. **Optical Rotation**: $[\alpha]_D^{20}$ = 489.7 (*c* 0.3, $CHCl_3$, 97% ee). The enantiomeric excess of **3da** was determined by HPLC analysis on a chiral stationary phase (Daicel Chiralcel OD-H column, column temperature 20 °C, solvent *n*-heptane:*i*PrOH = 99:1, flow rate 0.6 mL/min, λ = 280 nm): t_R = 8.6 min (minor), t_S = 9.8 min (major).

Analytical data for (*R*)-**1d**

The enantiomeric excess of **1d** (31% ee) was determined by HPLC analysis on a chiral stationary phase (Daicel Chiralcel OD-H column, column temperature 20 °C, solvent *n*-heptane:*i*PrOH = 99:1, flow rate 0.6 mL/min, λ = 280 nm): t_R = 8.2 min (major), t_S = 9.3 min

(minor). The NMR data are in accordance with those reported for the racemic compound *rac*-**1c**.

(*S*)-1-(3-methyl-[1,1'-binaphthalen]-2-yl)-2-phenyldiazene [(*S*)-3db] and (*R*)-3-methyl-[1,1'-binaphthalen]-2-yl trifluoromethanesulfonate [(*R*)-1d]

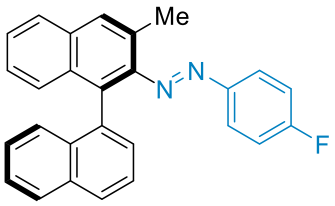

(*S*)-**3db**
$C_{27}H_{19}FN_2$
M = 390.46 g/mol

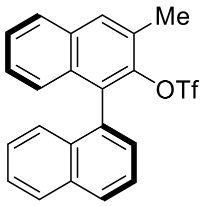

(*R*)-**1d**
$C_{22}H_{15}F_3O_3S$
M = 416.41 g/mol

Prepared according to **GP 2** from *rac*-**1d** (41.6 mg, 0.100 mmol, 1.00 equiv) and (*E*)-1-(4-fluorophenyl)-2-(trimethylsilyl)diazene **2b** (24.0 mg, 0.120 mmol, 1.20 equiv), $Pd(OAc)_2$ (1.1 mg, 5 mol %), **L1** (2.8 mg, 6 mol %), K_2CO_3 (13.8 mg, 0.100 mmol, 1.00 equiv). The reaction was stirred for 72 h at 60 °C. Flash column chromatography on silica gel using *n*-pentane and CH_2Cl_2 as the eluent (100:1→50:1→20:1) afforded (*S*)-**3db** (12.3 mg, 32% yield, 90% ee) as an orange oil and (*R*)-**1d** (26.5 mg, 64% yield, 74% ee) as a pale yellow solid.

Analytical data for (*S*)-**3db**

$\mathbf{R_f}$ = 0.30 (*n*-hexane/CH_2Cl_2 = 10/1) **^{1}H NMR** (400 MHz, $CDCl_3$, 298 K) δ = 7.92–7.83 (m, 4H), 7.52–7.45 (m, 2H), 7.45–7.39 (m, 1H), 7.39–7.29 (m, 3H), 7.29–7.21 (m, 2H), 7.17–7.08 (m, 2H), 6.98–6.86 (m, 2H), 2.59 (s, 3H). **^{13}C{^{1}H} NMR** (101 MHz, $CDCl_3$, 298 K) δ = 164.2 (d, *J* = 258.4 Hz), 149.7, 149.3 (d, *J* = 2.8 Hz), 135.4, 133.5, 133.3, 133.3, 132.6, 130.3, 129.9, 129.0, 128.8, 128.1, 127.7, 127.4, 127.3, 126.9, 126.6, 126.1, 126.0, 125.7, 125.1, 124.1 (d, *J* = 8.9 Hz), 115.8 (d, *J* = 23.0 Hz), 19.9. **^{19}F NMR** (471 MHz, $CDCl_3$, 298 K) δ = −109.8–−109.9 (m). **HRMS** (ESI): calculated for $C_{27}H_{20}FN_2^+$ [$(M+H)^+$]: 391.1605; found: 391.1605. **IR** (ATR): ṽ = 3028, 2923, 1593, 1500, 1228, 1136, 842, 798, 776, 750 cm^{-1}. **Optical Rotation**: $[\alpha]_D^{20}$ = 517.3 (*c* 0.3, $CHCl_3$, 90% ee). The enantiomeric excess of **3db** was determined by HPLC analysis on a chiral stationary phase (Daicel Chiralcel OD-H column, column temperature 20 °C, solvent *n*-heptane:*i*PrOH = 99:1, flow rate 0.6 mL/min, λ = 280 nm): t_R = 8.9 min (minor), t_S = 10.2 min (major).

Analytical data for (*R*)-**1d**

$\mathbf{R_f}$ = 0.35 (*n*-hexane/CH_2Cl_2 = 10/1). **HRMS** (APCI): calculated for $C_{22}H_{15}F_3O_3S^+$ [M^+]: 416.0689; found: 416.0685. **IR** (ATR): ṽ = 3055, 2929, 1593, 1502, 1405, 1201, 1132, 1058,

937, 886, 818, 775, 751, 670 cm^{-1}. **Optical Rotation**: $[\alpha]_D^{20}$ = 2.33 (*c* 1.5, $CHCl_3$, 74% ee). The enantiomeric excess of **1d** was determined by HPLC analysis on a chiral stationary phase (Daicel Chiralcel OD-H column, column temperature 20 °C, solvent *n*-heptane:*i*-PrOH = 99:1, flow rate 0.6 mL/min, λ = 280 nm): t_R = 8.4 min (major), t_S = 9.8 min (minor). The NMR data are in accordance with those reported for the racemic compound *rac*-**1d**.

(*S*)-1-(3-fluoro-[1,1'-binaphthalen]-2-yl)-2-phenyldiazene [(*S*)-3ea] and (*R*)-3-fluoro-[1,1'-binaphthalen]-2-yl trifluoromethanesulfonate [(*R*)-1e]

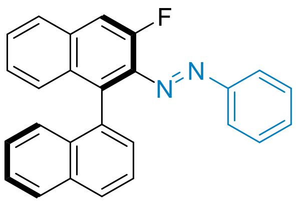

(*S*)-**3ea**
$C_{26}H_{17}FN_2$
M = 376.43 g/mol

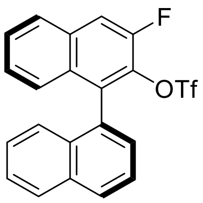

(*R*)-**1e**
$C_{21}H_{12}F_4O_3S$
M = 420.38 g/mol

Prepared according to **GP 2** from *rac*-**1e** (42.0 mg, 0.100 mmol, 1.00 equiv) and (*E*)-1-phenyl-2-(trimethylsilyl)diazene **2a** (21.4 mg, 0.120 mmol, 1.20 equiv), $Pd(OAc)_2$ (2.2 mg, 10 mol %), **L1** (5.6 mg, 12 mol %), K_2CO_3 (13.8 mg, 0.100 mmol, 1.00 equiv). The reaction was stirred for 168 h at 60 °C. Flash column chromatography on silica gel using *n*-pentane and CH_2Cl_2 as the eluent (100:1→50:1→20:1) afforded (*S*)-**3ea** (7.9 mg, 20% yield, 88% ee) as an orange oil and (*R*)-**1e** (24.8 mg, 59% yield, 36% ee) as a pale yellow oil.

Analytical data for (*S*)-**3ea**

R_f = 0.30 (*n*-hexane/CH_2Cl_2 = 10/1) **^{1}H NMR** (400 MHz, $CDCl_3$, 298 K) δ = 7.97–7.88 (m, 3H), 7.74 (d, *J* = 11.9 Hz, 1H), 7.58–7.50 (m, 2H), 7.47–7.23 (m, 11H). **^{13}C{^{1}H} NMR** (101 MHz, $CDCl_3$, 298 K) *δ* = 153.2, 152.2 (d, *J* = 258.5 Hz), 140.3 (d, *J* = 10.6 Hz), 136.2 (d, *J* = 1.3 Hz), 134.0 (d, *J* = 1.7 Hz), 133.6 (d, *J* = 9.3 Hz), 133.4, 133.2, 131.3, 130.7, 129.0, 128.9, 128.3, 128.2, 127.8, 127.6 (d, *J* = 2.2 Hz), 127.5 (d, *J* = 5.4 Hz), 126.6, 126.4, 126.1 (d, *J* = 2.2 Hz), 125.9, 125.1, 122.6, 113.0 (d, *J* = 19.6 Hz). **^{19}F NMR** (471 MHz, $CDCl_3$, 298 K) *δ* = −127.4 (d, *J* = 12.1 Hz). **HRMS** (ESI): calculated for $C_{26}H_{18}FN_2^+$ [$(M+H)^+$]: 377.1449; found: 377.1450. **IR** (ATR): ṽ = 3058, 2924, 1598, 1501, 1334, 1148, 799, 776, 750, 689 cm^{-1}. **Optical Rotation**: $[\alpha]_D^{20}$ = 471.7 (*c* 0.3, $CHCl_3$, 88% ee). The enantiomeric excess of **3ea** was determined by HPLC analysis on a chiral stationary phase (Daicel Chiralcel OD-H column, column temperature 20 °C, solvent *n*-heptane:*i*PrOH = 99:1, flow rate 0.6 mL/min, λ = 280 nm): t_R = 13.4 min (minor), t_S = 19.3 min (major).

Analytical data for (*R*)-**1e**

R_f = 0.35 (*n*-hexane/CH_2Cl_2 = 10/1). **HRMS** (APCI): calculated for $C_{21}H_{12}F_4O_3S^+$ [M^+]:

420.0438; found: 420.0436. **IR** (ATR): ṽ = 3057, 1604, 1505, 1419, 1208, 1132, 1090, 944, 901, 813, 775, 751, 699 cm^{-1}. **Optical Rotation**: $[\alpha]_D^{20}$ = 51.5 (*c* 2.0, $CHCl_3$, 36% ee). The enantiomeric excess of **1e** was determined by HPLC analysis on a chiral stationary phase (Daicel Chiralcel OD-H column, column temperature 20 °C, solvent *n*-heptane:*i*PrOH = 99:1, flow rate 0.6 mL/min, λ = 280 nm): t_R = 12.3 min (major), t_S = 13.6 min (minor). The NMR data are in accordance with those reported for the racemic compound *rac*-**1e**.

(*S*)-1-(1-(phenanthren-9-yl)naphthalen-2-yl)-2-(p-tolyl)diazene [(*S*)-3fe] and (*R*)-1-(phenanthren-9-yl)naphthalen-2-yl trifluoromethanesulfonate [(*R*)-1f]

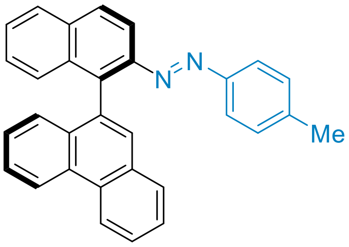

(*S*)-**3fe**
$C_{31}H_{22}N_2$
M = 422.53 g/mol

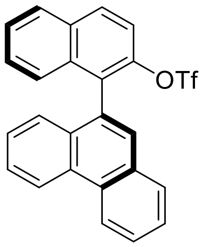

(*R*)-**1f**
$C_{25}H_{15}F_3O_3S$
M = 452.45 g/mol

Prepared according to **GP 2** from *rac*-**1f** (45.2 mg, 0.100 mmol, 1.00 equiv) and (*E*)-1-(p-tolyl)-2-(trimethylsilyl)diazene **2e** (23.0 mg, 0.120 mmol, 1.20 equiv), $Pd(OAc)_2$ (2.2 mg, 10 mol %), **L1** (5.6 mg, 12 mol %), K_2CO_3 (13.8 mg, 0.100 mmol, 1.00 equiv). The reaction was stirred for 96 h at 25 °C. Flash column chromatography on silica gel using *n*-pentane and CH_2Cl_2 as the eluent (100:1→50:1→20:1) afforded (*S*)-**3fe** (20.9 mg, 50% yield, 75% ee) as an orange solid and (*R*)-**1f** (19.8 mg, 44% yield, 90% ee) as a white solid.

Analytical data for (*S*)-**3fe**

R_f = 0.30 (*n*-hexane/CH_2Cl_2 = 10/1). **M.p.**: 127–135 °C. **^{1}H NMR** (500 MHz, $CDCl_3$, 298 K) δ = 8.84 (dd, *J* = 8.5, 2.9 Hz, 2H), 8.21–8.14 (m, 1H), 8.05 (d, *J* = 8.9 Hz, 1H), 7.99 (d, *J* = 8.2 Hz, 1H), 7.90 (d, *J* = 7.9 Hz, 1H), 7.82 (s, 1H), 7.76–7.72 (m, 1H), 7.68–7.59 (m, 3H), 7.57–7.52 (m, 1H), 7.43 (d, *J* = 8.2 Hz, 1H), 7.37–7.30 (m, 2H), 7.30–7.22 (m, 2H), 7.01 (d, *J* = 7.9 Hz, 2H), 2.26 (s, 3H). **^{13}C{^{1}H} NMR** (126 MHz, $CDCl_3$, 298 K) δ = 151.1, 148.2, 141.3, 139.3, 134.9, 134.09, 134.06, 133.1, 131.6, 130.5, 130.2, 129.7, 129.6, 129.2, 128.9, 128.3, 127.9, 127.7, 127.5, 126.9, 126.8, 126.7, 126.4, 123.0, 122.78, 122.75, 114.8, 21.5. **HRMS** (ESI): calculated for $C_{31}H_{23}N_2^+$ [(M+H)$^+$]: 423.1856; found: 423.1850. **IR** (ATR): ṽ = 3054, 2920, 1920, 1720, 1601, 1494, 1447, 1262, 1149, 827, 738, 735 cm^{-1}. **Optical Rotation**: $[\alpha]_D^{20}$ = 280.5 (*c* 1.5, $CHCl_3$, 75% ee). The enantiomeric excess of **3fe** was determined by HPLC analysis on a chiral stationary phase (Daicel Chiralcel OD-H column, column temperature 20 °C, solvent *n*-heptane:*i*PrOH = 99:1, flow rate 0.6 mL/min, λ = 280 nm): t_R = 13.4 min (minor), t_S = 15.5 min (major).

Analytical data for (*R*)-**1f**

R*f* = 0.35 (*n*-hexane/CH_2Cl_2 = 10/1). **M.p.**: 95–100 °C. **HRMS** (APCI): calculated for $C_{25}H_{15}F_3O_3S^+$ [M$^+$]: 452.0689; found: 452.0684. **IR** (ATR): ṽ = 3064, 2925, 1420, 1212, 1138, 951, 833, 748, 675 cm^{-1}. **Optical Rotation**: $[\alpha]_D^{20}$ = −35.0 (*c* 1.0, $CHCl_3$, 90% ee). The enantiomeric excess of **1f** was determined by HPLC analysis on a chiral stationary phase (Daicel Chiralcel OD-H column, column temperature 20 °C, solvent *n*-heptane:*i*PrOH = 99:1, flow rate 0.6 mL/min, λ = 280 nm): t_S = 15.0 min (minor), t_R = 17.8 min (major). The NMR data are in accordance with those reported for the racemic compound *rac*-**1f**.

(*S*)-1-(1-(2-methoxyphenyl)naphthalen-2-yl)-2-phenyldiazene [(*S*)-3ga] and (*R*)-1-(2-methoxyphenyl)naphthalen-2-yl trifluoromethanesulfonate [(*R*)-1g]

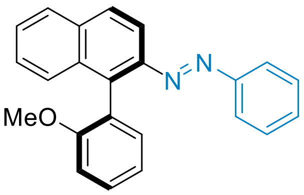

(*S*)-**3ga**
$C_{23}H_{18}N_2O$
M = 338.41 g/mol

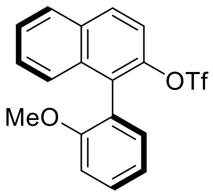

(*R*)-**1g**
$C_{18}H_{13}F_3O_4S$
M = 382.35 g/mol

Prepared according to **GP 2** from *rac*-**1g** (38.2 mg, 0.100 mmol, 1.00 equiv) and (*E*)-1-phenyl-2-(trimethylsilyl)diazene **2a** (21.4 mg, 0.120 mmol, 1.20 equiv), $Pd(OAc)_2$ (2.2 mg, 10 mol %), **L1** (5.6 mg, 12 mol %), K_2CO_3 (13.8 mg, 0.100 mmol, 1.00 equiv). The reaction was stirred for 72 h at 25 °C. Flash column chromatography on silica gel using *n*-pentane and CH_2Cl_2 as the eluent (50:1→20:1→10:1) afforded (*S*)-**3ga** (14.3 mg, 42% yield, 82% ee; 46% recrystallization yield, 95% ee) as an orange solid and (*R*)-**1g** (18.8 mg, 49% yield, 97% ee) as a pale yellow oil.

Analytical data for (*S*)-**3ga**

R*f* = 0.25 (*n*-hexane/CH_2Cl_2 = 5/1). **M.p.**: 77–90 °C. **^{1}H NMR** (500 MHz, $CDCl_3$, 298 K) δ = 8.06 (d, *J* = 9.0 Hz, 1H), 7.92 (d, *J* = 8.9 Hz, 2H), 7.75 (d, *J* = 8.5 Hz, 1H), 7.68–7.63 (m, 2H), 7.57–7.53 (m, 1H), 7.51–7.36 (m, 5H), 7.32 (dd, *J* = 7.4, 1.8 Hz, 1H), 7.14–7.05 (m, 2H), 3.61 (s, 3H). **^{13}C{^{1}H} NMR** (126 MHz, $CDCl_3$, 298 K) δ = 158.1, 153.2, 147.4, 138.4, 135.1, 133.4, 132.9, 130.7, 129.2, 129.1, 128.8, 128.3, 127.5, 127.3, 126.6, 126.2, 123.1, 120.1, 114.7, 111.0, 55.8. **HRMS** (ESI): calculated for $C_{23}H_{19}N_2O^+$ [(M+H)$^+$]: 339.1492; found: 339.1493. **IR** (ATR): ṽ = 3058, 2929, 1598, 1596, 1491, 1459, 1433, 1245, 1025, 822, 749, 691 cm^{-1}. **Optical Rotation**: $[\alpha]_D^{20}$ = 33.5 (*c* 1.0, $CHCl_3$, 82% ee). The enantiomeric excess of **3ga** was determined by HPLC analysis on a chiral stationary phase (Daicel Chiralcel AD-H column, column temperature 20 °C, solvent *n*-heptane:*i*PrOH =

99.8:0.2, flow rate 0.6 mL/min, λ = 280 nm): t_S = 23.4 min (major), t_R = 29.7 min (major).

Analytical data for (*R*)-**1g**

R*f* = 0.30 (*n*-hexane/CH_2Cl_2 = 5/1). **HRMS** (APCI): calculated for $C_{18}H_{13}F_3O_4S^+$ [M^+]: 382.0481; found: 382.0483. **IR** (ATR): ṽ = 3059, 2925, 1580, 1494, 1461, 1416, 1247, 1202, 1172, 1138, 1104, 1025, 947, 833, 810, 750, 670 cm^{-1}. **Optical Rotation**: $[\alpha]_D^{20}$ = −31.6 (*c* 1.0, $CHCl_3$, 97% ee). The enantiomeric excess of **1g** was determined by HPLC analysis on a chiral stationary phase (Daicel Chiralcel IB column, column temperature 20 °C, solvent *n*-heptane:*i*PrOH = 99.8:0.2, flow rate 0.6 mL/min, λ = 280 nm): t_R = 12.3 min (major), t_S = 13.2 min (minor). The NMR data are in accordance with those reported for the racemic compound *rac*-**1g**.

(*S*)-1-(4-fluorophenyl)-2-(1-(2-methoxyphenyl)naphthalen-2-yl)diazene [(*S*)-3gb] and (*R*)-1-(2-methoxyphenyl)naphthalen-2-yl trifluoromethanesulfonate [(*R*)-1g]

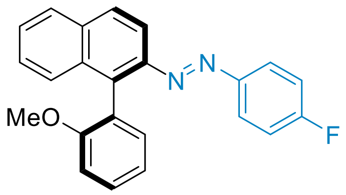

(*S*)-**3gb**
$C_{23}H_{17}FN_2O$
M = 356.40 g/mol

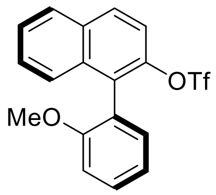

(*R*)-**1g**
$C_{18}H_{13}F_3O_4S$
M = 382.35 g/mol

Prepared according to **GP 2** from *rac*-**1g** (38.2 mg, 0.100 mmol, 1.00 equiv) and (*E*)-1-(4-fluorophenyl)-2-(trimethylsilyl)diazene **2b** (24.0 mg, 0.120 mmol, 1.20 equiv), $Pd(OAc)_2$ (2.2 mg, 10 mol %), **L1** (5.6 mg, 12 mol %), K_2CO_3 (13.8 mg, 0.100 mmol, 1.00 equiv). The reaction was stirred for 72 h at 25 °C. Flash column chromatography on silica gel using *n*-pentane and CH_2Cl_2 as the eluent (50:1→20:1→10:1) afforded (*S*)-**3gb** (12.2 mg, 34% yield, 83% ee) as an orange oil and (*R*)-**1g** (22.0 mg, 58% yield, 88% ee) as a pale yellow oil.

Analytical data for (*S*)-**3gb**

R*f* = 0.25 (*n*-hexane/CH_2Cl_2 = 5/1). **M.p.**: 90–110 °C. **^{1}H NMR** (500 MHz, $CDCl_3$, 298 K) *δ* = 8.04 (dd, *J* = 9.1, 2.2 Hz, 1H), 7.92 (d, *J* = 9.1 Hz, 2H), 7.74 (d, *J* = 8.4 Hz, 1H), 7.68–7.60 (m, 2H), 7.59–7.52 (m, 1H), 7.52–7.42 (m, 2H), 7.31 (dd, *J* = 7.6, 2.1 Hz, 1H), 7.14–7.03 (m, 4H), 3.61 (s, 3H). **^{13}C{^{1}H} NMR** (126 MHz, $CDCl_3$, 298 K) *δ* = 164.2 (d, *J* = 252.0 Hz), 158.1, 150.0 (d, *J* = 2.0 Hz), 147.2, 138.5, 135.1, 133.4, 132.9, 129.3, 128.8, 128.3, 127.5, 127.4, 126.6, 126.1, 125.0 (d, *J* = 8.7 Hz), 120.1, 116.0 (d, *J* = 22.9 Hz), 114.6, 110.9, 55.8. **^{19}F NMR** (471 MHz, $CDCl_3$, 298 K) *δ* = −110.01–−110.25 (m). **HRMS** (ESI): calculated for $C_{23}H_{18}FN_2O^+$ [$(M+H)^+$]: 357.1398; found: 357.1397. **IR** (ATR): ṽ = 3061, 2930,

1592, 1494, 1460, 1434, 1228, 1137, 1026, 843, 751, 679 cm^{-1}. **Optical Rotation**: $[\alpha]_D^{20}$ = −234.3 (*c* 0.8, $CHCl_3$, 83% ee). The enantiomeric excess of **3gb** was determined by HPLC analysis on a chiral stationary phase (Daicel Chiralcel IB column, column temperature 20 °C, solvent *n*-heptane:*i*PrOH = 99.8:0.2, flow rate 0.6 mL/min, λ = 230 nm): t_R = 15.8 min (minor), t_S = 17.0 min (major).

Analytical data for (*R*)-**1g**

The enantiomeric excess of **1g** (88% ee) was determined by HPLC analysis on a chiral stationary phase (Daicel Chiralcel IB column, column temperature 20 °C, solvent *n*-heptane:*i*PrOH = 99.8:0.2, flow rate 0.6 mL/min, λ = 280 nm): t_R = 11.7 min (major), t_S = 12.6 min (minor). The NMR data are in accordance with those reported for the racemic compound *rac*-**1g**.

(*S*)-1-(1-(2-ethylphenyl)naphthalen-2-yl)-2-phenyldiazene [(*S*)-3ha] and (*R*)-1-(2-ethylphenyl)naphthalen-2-yl trifluoromethanesulfonate [(*R*)-1h]

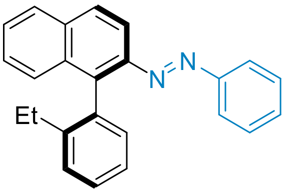

(*S*)-**3ha**
$C_{24}H_{20}N_2$
M = 336.44 g/mol

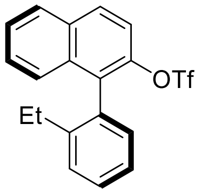

(*R*)-**1h**
$C_{19}H_{15}F_3O_3S$
M = 380.38 g/mol

Prepared according to **GP 2** from *rac*-**1h** (38.0 mg, 0.100 mmol, 1.00 equiv) and (*E*)-1-phenyl-2-(trimethylsilyl)diazene **2a** (21.4 mg, 0.120 mmol, 1.20 equiv), $Pd(OAc)_2$ (2.2 mg, 10 mol %), **L1** (5.6 mg, 12 mol %), K_2CO_3 (13.8 mg, 0.100 mmol, 1.00 equiv). The reaction was stirred for 72 h at 25 °C. Flash column chromatography on silica gel using *n*-pentane and CH_2Cl_2 as the eluent (100:1→50:1→20:1) afforded (*S*)-**3ha** (10.8 mg, 32% yield, 88% ee) as an orange solid and (*R*)-**1h** (17.0 mg, 45% yield, 50% ee) as a pale yellow oil.

Analytical data for (*S*)-**3ha**

R_f = 0.35 (*n*-hexane/CH_2Cl_2 = 10/1). **M.p.**: 77–98 °C. **^{1}H NMR** (500 MHz, $CDCl_3$, 298 K) δ = 8.07 (d, *J* = 9.0 Hz, 1H), 7.96–7.90 (m, 2H), 7.65–7.58 (m, 3H), 7.58–7.53 (m, 1H), 7.47–7.37 (m, 7H), 7.31 (td, *J* = 7.2, 1.8 Hz, 1H), 7.27–7.25 (m, 1H), 2.38–2.26 (m, *J* = 7.4 Hz, 2H), 0.91 (t, *J* = 7.6 Hz, 3H). **^{13}C{^{1}H} NMR** (126 MHz, $CDCl_3$, 298 K) δ = 153.1, 147.2, 143.4, 141.2, 136.4, 135.0, 133.6, 131.3, 130.8, 129.1, 128.7, 128.3, 127.9, 127.8, 127.6, 127.5, 126.7, 125.1, 123.1, 114.6, 26.8, 14.9. **HRMS** (ESI): calculated for $C_{24}H_{21}N_2^+$ [(M+H)$^+$]: 337.1699; found: 337.1700. **IR** (ATR): ṽ = 3057, 2964, 2928, 1486, 1220, 1149, 822, 750, 690 cm^{-1}. **Optical Rotation**: $[\alpha]_D^{20}$ = 355.3 (*c* 0.8, $CHCl_3$, 88% ee). The

enantiomeric excess of **3ha** was determined by HPLC analysis on a chiral stationary phase (Daicel Chiralcel OD-H column, column temperature 20 °C, solvent *n*-heptane:*i*PrOH = 99.8:0.2, flow rate 0.6 mL/min, λ = 280 nm): t_S = 12.4 min (major), t_R = 14.6 min (minor).

Analytical data for (*R*)-**1h**

R*f* = 0.30 (*n*-hexane/CH_2Cl_2 = 10/1). **HRMS** (APCI): calculated for $C_{19}H_{15}F_3O_3S^+$ [M$^+$]: 380.0689; found: 380.0691. **IR** (ATR): ṽ = 3062, 2968, 1421, 1209, 1171, 1140, 938, 836, 754, 691 cm^{-1}. **Optical Rotation**: $[\alpha]_D^{20}$ = −1.30 (*c* 1.0, $CHCl_3$, 50% ee). The enantiomeric excess of **1h** was determined by HPLC analysis on a chiral stationary phase (Daicel Chiralcel OD-H column, column temperature 20 °C, solvent *n*-heptane:*i*PrOH = 99.8:0.2, flow rate 0.6 mL/min, λ = 280 nm): t_S = 10.2 min (minor), t_R = 10.8 min (major). The NMR data are in accordance with those reported for the racemic compound *rac*-**1h**.

(*S*)-1-phenyl-2-(1-(o-tolyl)naphthalen-2-yl)diazene [(*S*)-3ia] and (*R*)-1-(o-tolyl)naphthalen-2-yl trifluoromethanesulfonate [(*R*)-1i]

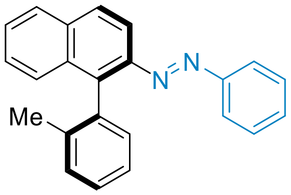

(*S*)-**3ia**
$C_{23}H_{18}N_2$
M = 322.41 g/mol

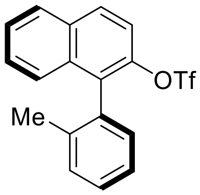

(*R*)-**1i**
$C_{18}H_{13}F_3O_3S$
M = 366.35 g/mol

Prepared according to **GP 2** from *rac*-**1i** (36.6 mg, 0.100 mmol, 1.00 equiv) and (*E*)-1-phenyl-2-(trimethylsilyl)diazene **2a** (21.4 mg, 0.120 mmol, 1.20 equiv), $Pd(OAc)_2$ (2.2 mg, 10 mol %), **L1** (5.6 mg, 12 mol %), K_2CO_3 (13.8 mg, 0.100 mmol, 1.00 equiv). The reaction was stirred for 40 h at 25 °C. Flash column chromatography on silica gel using *n*-pentane and CH_2Cl_2 as the eluent (100:1→50:1→20:1) afforded (*S*)-**3ia** (16.4 mg, 51% yield, 63% ee) as an orange oil and (*R*)-**1i** (12.3 mg, 34% yield, 99% ee) as a colorless oil.

Analytical data for (*S*)-**3ia**

R*f* = 0.30 (*n*-hexane/CH_2Cl_2 = 10/1). **M.p.**: 53–59 °C. **^{1}H NMR** (500 MHz, $CDCl_3$, 298 K) δ = 8.09–8.05 (m, 1H), 7.96–7.92 (m, 2H), 7.66–7.59 (m, 3H), 7.58–7.54 (m, 1H), 7.46–7.35 (m, 6H), 7.34–7.28 (m, 2H), 1.98 (s, 3H). **^{13}C{^{1}H} NMR** (126 MHz, $CDCl_3$, 298 K) δ = 153.1, 147.1, 141.2, 137.5, 137.0, 135.1, 133.3, 131.2, 130.8, 129.6, 129.1, 128.7, 128.3, 127.7, 127.5, 127.4, 126.8, 125.1, 123.1, 114.7, 20.5. **HRMS** (ESI): calculated for $C_{23}H_{19}N_2^+$ [(M+H)$^+$]: 323.1543; found: 323.1544. **IR** (ATR): ṽ = 3058, 2922, 1589, 1486, 1150, 822, 751, 690 cm^{-1}. **Optical Rotation**: $[\alpha]_D^{20}$ = 184.4 (*c* 1.0, $CHCl_3$, 63% ee). The enantiomeric excess of **3ia** was determined by HPLC analysis on a chiral stationary phase (Daicel

Chiralcel OD-H column, column temperature 20 °C, solvent *n*-heptane:*i*PrOH = 99.8:0.2, flow rate 0.6 mL/min, λ = 280 nm): t_S = 12.8 min (major), t_R = 15.5 min (minor).

Analytical data for (*R*)-**1i**

R*f* = 0.35 (*n*-hexane/CH_2Cl_2 = 10/1). **HRMS** (APCI): calculated for $C_{18}H_{13}F_3O_3S^+$ [M^+]: 366.0532; found: 366.0531. **IR** (ATR): ṽ = 3061, 2925, 1420, 1208, 1140, 936, 833, 754, 670 cm^{-1}. **Optical Rotation**: $[\alpha]_D^{20}$ = 4.50 (*c* 0.8, $CHCl_3$, 99% ee). The enantiomeric excess of **1i** was determined by HPLC analysis on a chiral stationary phase (Daicel Chiralcel OJ-H column, column temperature 20 °C, solvent *n*-heptane:*i*PrOH = 99:1, flow rate 0.6 mL/min, λ = 280 nm): t_R = 10.5 min (major), t_S = 14.6 min (minor). The NMR data are in accordance with those reported for the racemic compound *rac*-**1i**.

(*S*)-1-(1-(4-fluoro-2-methylphenyl)naphthalen-2-yl)-2-phenyldiazene [(*S*)-3ja] and (*R*)-1-(4-fluoro-2-methylphenyl)naphthalen-2-yl trifluoromethanesulfonate [(*R*)-1j]

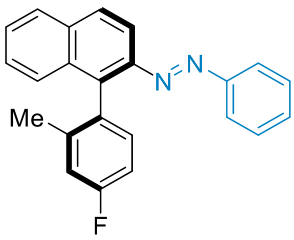

(*S*)-**3ja**
$C_{23}H_{17}FN_2$
M = 340.40 g/mol

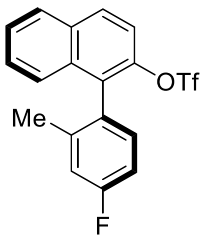

(*R*)-**1j**
$C_{18}H_{12}F_4O_3S$
M = 384.34 g/mol

Prepared according to **GP 2** from *rac*-**1j** (38.4 mg, 0.100 mmol, 1.00 equiv) and (*E*)-1-phenyl-2-(trimethylsilyl)diazene **2a** (21.4 mg, 0.120 mmol, 1.20 equiv), $Pd(OAc)_2$ (2.2 mg, 10 mol %), **L1** (5.6 mg, 12 mol %), K_2CO_3 (13.8 mg, 0.100 mmol, 1.00 equiv). The reaction was stirred for 40 h at 25 °C. Flash column chromatography on silica gel using *n*-pentane and CH_2Cl_2 as the eluent (100:1→50:1→20:1) afforded (*S*)-**3ja** (13.7 mg, 40% yield, 83% ee) as an orange oil and (*R*)-**1j** (17.7 mg, 46% yield, 59% ee) as a pale yellow solid.

Analytical data for (*S*)-**3ja**

R*f* = 0.30 (*n*-hexane/CH_2Cl_2 = 10/1) **^{1}H NMR** (500 MHz, $CDCl_3$, 298 K) *δ* = 8.06 (d, *J* = 9.0 Hz, 1H), 7.94 (d, *J* = 8.6 Hz, 2H), 7.66–7.61 (m, 2H), 7.60–7.55 (m, 2H), 7.49–7.38 (m, 4H), 7.27–7.21 (m, 1H), 7.09 (dd, *J* = 9.9, 2.7 Hz, 1H), 7.06–7.00 (m, 1H), 1.97 (s, 3H). **^{13}C{^{1}H} NMR** (101 MHz, $CDCl_3$, 298 K) *δ* = 162.4 (d, *J* = 245.6 Hz), 153.0, 147.2, 140.1, 140.0 (d, *J* = 7.7 Hz), 135.0, 133.3, 132.8 (d, *J* = 3.0 Hz), 132.5 (d, *J* = 8.3 Hz), 131.0, 129.2, 129.0, 128.4, 127.6, 127.1, 126.9, 123.1, 116.2 (d, *J* = 21.0 Hz), 114.7, 112.1 (d, *J* = 21.1 Hz), 20.7. **^{19}F NMR** (471 MHz, $CDCl_3$, 298 K) *δ* = −115.93–−116.12 (m). **HRMS** (ESI): calculated for $C_{23}H_{18}FN_2^+$ [$(M+H)^+$]: 341.1449; found: 341.1451. **IR** (ATR): ṽ = 3058, 2922,

1588, 1494, 1269, 1151, 823, 750, 691 cm^{-1}. **Optical Rotation**: $[\alpha]_D^{20}$ = 244.4 (*c* 0.8, $CHCl_3$, 83% ee). The enantiomeric excess of **3ja** was determined by HPLC analysis on a chiral stationary phase (Daicel Chiralcel OD-H column, column temperature 20 °C, solvent *n*-heptane:*i*PrOH = 99.8:0.2, flow rate 0.6 mL/min, λ = 280 nm): t_S = 12.5 min (major), t_R = 13.4 min (minor).

Analytical data for (*R*)-**1j**

R*f* = 0.35 (*n*-hexane/CH_2Cl_2 = 10/1). **M.p.**: 48–56 °C. **HRMS** (APCI): calculated for $C_{18}H_{12}F_4O_3S^+$ [M$^+$]: 384.0438; found: 384.0441. **IR** (ATR): ṽ = 3063, 2926, 1585, 1421, 1209, 1140, 952, 836, 680 cm^{-1}. **Optical Rotation**: $[\alpha]_D^{20}$ = 3.50 (*c* 1.0, $CHCl_3$, 59% ee). The enantiomeric excess of **1j** was determined by HPLC analysis on a chiral stationary phase (Daicel Chiralcel AD-H column, column temperature 20 °C, solvent *n*-heptane:*i*PrOH = 99:1, flow rate 0.6 mL/min, λ = 280 nm): t_S = 8.2 min (minor), t_R = 9.1 min (major). The NMR data are in accordance with those reported for the racemic compound *rac*-**1j**.

(*S*)-1-(1-(2-chlorophenyl)naphthalen-2-yl)-2-phenyldiazene [(*S*)-3ka] and (*R*)-1-(2-chlorophenyl)naphthalen-2-yl trifluoromethanesulfonate [(*R*)-1k]

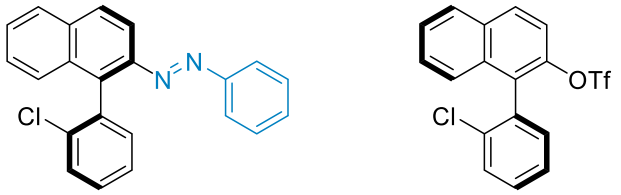

(*S*)-**3ka**
$C_{22}H_{15}ClN_2$
M = 342.83 g/mol

(*R*)-**1k**
$C_{17}H_{10}ClF_3O_3S$
M = 386.77 g/mol

Prepared according to **GP 2** from *rac*-**1k** (38.5 mg, 0.100 mmol, 1.00 equiv) and (*E*)-1-phenyl-2-(trimethylsilyl)diazene **2a** (21.4 mg, 0.120 mmol, 1.20 equiv), $Pd(OAc)_2$ (2.2 mg, 10 mol %), **L1** (5.6 mg, 12 mol %), K_2CO_3 (13.8 mg, 0.100 mmol, 1.00 equiv). The reaction was stirred for 72 h at 25 °C. Flash column chromatography on silica gel using *n*-pentane and CH_2Cl_2 as the eluent (100:1→50:1→20:1) afforded (*S*)-**3ka** (10.6 mg, 31% yield, 72% ee) as an orange solid and (*S*)-**1k** (13.8 mg, 36% yield, 73% ee) as a pale yellow oil.

Analytical data for (*S*)-**3ka**

R*f* = 0.40 (*n*-hexane/CH_2Cl_2 = 5/1). **M.p.**: 59–77 °C. **^{1}H NMR** (500 MHz, $CDCl_3$, 298 K) δ = 8.10 (d, *J* = 8.9 Hz, 1H), 8.01–7.92 (m, 2H), 7.68–7.55 (m, 5H), 7.50–7.38 (m, 7H). **^{13}C{^{1}H} NMR** (126 MHz, $CDCl_3$, 298 K) δ = 153.1, 147.1, 138.9, 136.5, 135.1, 135.0, 132.9, 132.9, 130.9, 129.5, 129.3, 129.12, 129.09, 128.4, 127.6, 127.0, 126.1, 123.2, 114.6. **HRMS** (ESI): calculated for $C_{22}H_{16}ClN_2^+$ [(M+H)$^+$]: 343.0997; found: 343.0997. **IR** (ATR): ṽ = 3058, 2923, 1591, 1475, 1058, 822, 750, 690 cm^{-1}. **Optical Rotation**: $[\alpha]_D^{20}$ = 264.4 (*c* 0.8, $CHCl_3$, 72%

ee). The enantiomeric excess of **3ka** was determined by HPLC analysis on a chiral stationary phase (Daicel Chiralcel OD-H column, column temperature 20 °C, solvent *n*-heptane:*i*PrOH = 99.8:0.2, flow rate 0.6 mL/min, λ = 280 nm): t_S = 21.2 min (major), t_R = 24.0 min (minor).

Analytical data for (*R*)-**1k**

R*f* = 0.50 (*n*-hexane/CH_2Cl_2 = 5/1). **HRMS** (APCI): calculated for $C_{17}H_{10}ClF_3O_3S^+$ [M$^+$]: 385.9986; found: 385.9988. **IR** (ATR): ṽ = 3062, 2925, 1509, 1420, 1209, 1174, 1140, 940, 835, 754, 676 cm^{-1}. **Optical Rotation**: $[\alpha]_D^{20}$ = −7.25 (*c* 0.8, $CHCl_3$, 73% ee). The enantiomeric excess of **1k** was determined by HPLC analysis on a chiral stationary phase (Daicel Chiralcel AD-H column, column temperature 20 °C, solvent *n*-heptane:*i*PrOH = 99.8:0.2, flow rate 0.6 mL/min, λ = 280 nm): t_S = 13.4 min (minor), t_R = 14.3 min (major). The NMR data are in accordance with those reported for the racemic compound *rac*-**1k**.

(*S*)-1-(1-([1,1'-biphenyl]-2-yl)naphthalen-2-yl)-2-phenyldiazene [(*S*)-3la] and (*R*)-1-([1,1'-biphenyl]-2-yl)naphthalen-2-yl trifluoromethanesulfonate [(*R*)-1l]

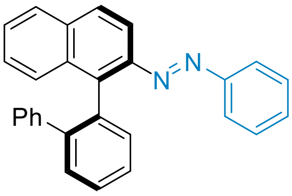

(*S*)-**3la**
$C_{28}H_{20}N_2$
M = 384.48 g/mol

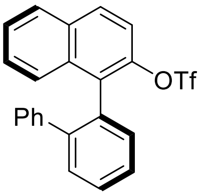

(*R*)-**1l**
$C_{23}H_{15}F_3O_3S$
M = 428.43 g/mol

Prepared according to **GP 2** from *rac*-**1l** (42.8 mg, 0.100 mmol, 1.00 equiv) and (*E*)-1-phenyl-2-(trimethylsilyl)diazene **2a** (21.4 mg, 0.120 mmol, 1.20 equiv), $Pd(OAc)_2$ (2.2 mg, 10 mol %), **L5** (6.0 mg, 12 mol %), K_2CO_3 (13.8 mg, 0.100 mmol, 1.00 equiv). The reaction was stirred for 72 h at 25 °C. Flash column chromatography on silica gel using *n*-pentane and CH_2Cl_2 as the eluent (100:1→50:1→20:1) afforded (*S*)-**3la** (9.9 mg, 26% yield, 82% ee) as an orange solid and (*R*)-**1l** (25.5 mg, 60% yield, 30% ee) as a pale yellow oil.

Analytical data for (*S*)-**3la**

R*f* = 0.25 (*n*-hexane/CH_2Cl_2 = 10/1) **^{1}H NMR** (500 MHz, $CDCl_3$, 298 K) δ = 7.88–7.77 (m, 4H), 7.66–7.61 (m, 2H), 7.59–7.39 (m, 9H), 6.98–6.91 (m, 3H), 6.90–6.85 (m, 2H). **^{13}C{^{1}H} NMR** (126 MHz, $CDCl_3$, 298 K) δ = 153.0, 147.1, 143.4, 141.7, 140.9, 135.7, 134.8, 133.6, 132.2, 130.8, 129.6, 129.0, 128.9, 128.7, 128.3, 128.0, 127.6, 127.5, 127.3, 126.7, 126.50, 126.47, 123.2, 114.5. **HRMS** (ESI) m/z: [M+H]$^+$ calcd for $C_{28}H_{21}N_2^+$ 385.1699; found 385.1700. **IR** (ATR): ṽ = 3056, 2923, 1593, 1478, 1221, 1149, 821, 746, 691 cm^{-1}. **Optical Rotation**: $[\alpha]_D^{20}$ = 149.0 (*c* 0.8, $CHCl_3$, 82% ee). The enantiomeric excess of **3la** was

determined by HPLC analysis on a chiral stationary phase (Daicel Chiralcel OD-H column, column temperature 20 °C, solvent *n*-heptane:*i*PrOH = 99:1, flow rate 0.6 mL/min, λ = 230 nm): t_S = 9.4 min (major), t_R = 12.3 min (minor).

Analytical data for (*R*)-**1l**

R*f* = 0.30 (*n*-hexane/CH_2Cl_2 = 10/1). **M.p.**: 63–72 °C. **HRMS** (APCI): calculated for $C_{23}H_{15}F_3O_3S^+$ [M^+]: 428.0689; found: 428.0689. **IR** (ATR): ṽ = 3058, 1418, 1205, 1138, 971, 939, 834, 746, 701, 669 cm^{-1}. **Optical Rotation**: $[\alpha]_D^{20}$ = 13.0 (*c* 2.0, $CHCl_3$, 30% ee). The enantiomeric excess of **1l** was determined by HPLC analysis on a chiral stationary phase (Daicel Chiralcel OD-H column, column temperature 20 °C, solvent *n*-heptane:*i*PrOH = 99:1, flow rate 0.6 mL/min, λ = 230 nm): t_S = 8.6 min (minor), t_R = 10.4 min (major). The NMR data are in accordance with those reported for the racemic compound *rac*-**1l**.

5 Match/Mismatch Control Experiments

5.1 Synthesis of (*S*) and (*R*)-1a from (*R*) and (*S*)-BINOL

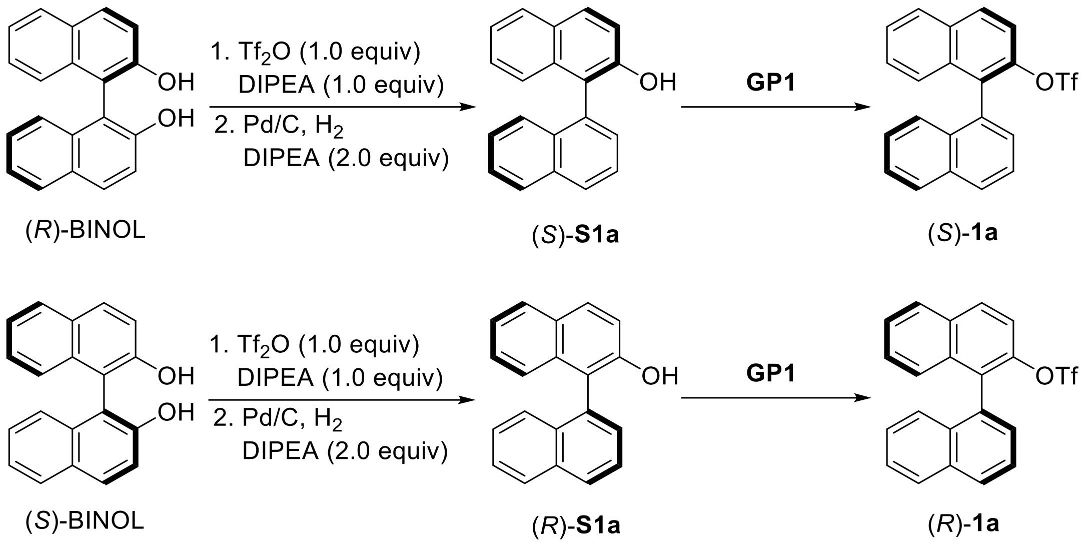

According to a modified literature procedure[S4], Tf_2O (5.0 mmol, 0.84 mL, 1.0 equiv) is added dropwise to a solution of (*R*)-BINOL (5.0 mmol, 1.4 g, 1.0 equiv) and DIPEA (5.0 mmol, 0.87 mL, 1.0 equiv) in CH_2Cl_2 at 0 °C. The reaction mixture is stirred at room temperature for 1 h. Upon completion (monitored by TLC), the reaction is quenched by the addition of water, and the resulting mixture is extracted with CH_2Cl_2 (3 × 10 mL). The combined organic phases are dried over Na_2SO_4 and concentrated under reduced pressure. Purification of the residue by flash column chromatography on silica gel using *n*-pentane and CH_2Cl_2 as the eluent (10:1→5:1) affords (*R*)-2'-hydroxy-[1,1'-binaphthalen]-2-yl trifluoromethanesulfonate (1.93 g, 93%). To a suspension of 10% palladium on charcoal (500 mg) in 50 mL of ethanol was added DIPEA (9.2 mmol, 1.6 mL, 2.0 equiv) and (*R*)-2'-hydroxy-[1,1'-binaphthalen]-2-yl trifluoromethanesulfonate (1.93 g, 4.60 mmol) at room temperature. The mixture was stirred under hydrogen gas (1 atm) for 6 h. The mixture was filtered through Celite pad and washed with EtOAc. The combined organic layer was concentrated under reduced pressure. Purification of the residue by flash column chromatography on silica gel using *n*-pentane and CH_2Cl_2 as the eluent (10:1→5:1) affords (*S*)-**S1a** (1.18 g, 95%) as a white solid. (*S*)-**1a** was prepared as a pale yellow oil (1.57 g, 90%) according to **GP 1** from (*S*)-**S1a**.

The same procedure afforded (*R*)-**1a** (1.62 g, 81%, 3 steps) as a white solid from (*S*)-BINOL.

5.2 Match/Mismatch Control Experiments

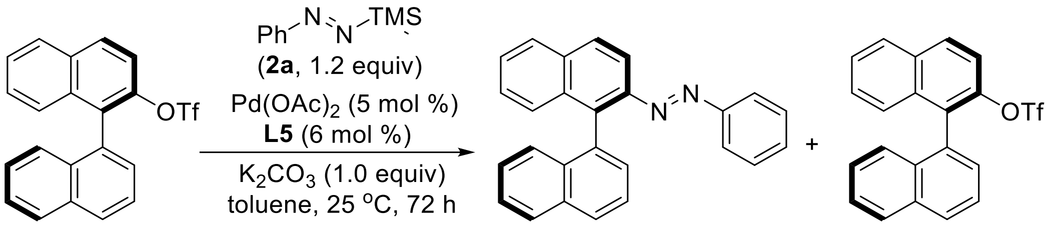

(*S*)-**1a**, 0.1 mmol (*S*)-**3a** 47%, >99% ee (*S*)-**1a** 48%, >99% ee

According to GP 2: in an argon-filled glovebox, an oven-dried 1.5 mL vial was charged with palladium acetate 5 mol %, **L5** 6 mol % and toluene (0.1 mL). The resulting suspension was stirred for 5 min at room temperature, at which time potassium carbonate (13.4 mg, 0.100 mmol, 1.00 equiv), biaryl trifluoromethanesulfonate (*S*)-**1a** (0.10 mmol, 40.2 mg, 1.0 equiv), (*E*)-1-phenyl-2-(trimethylsilyl)diazene **2a** (0.120 mmol, 21.4 mg, 1.20 equiv) and toluene (0.1 mL) were added. The reaction stirred for 72 h at 25 °C. The reaction was quenched by filtration over silica, and flash column chromatography on silica gel using *n*-pentane and CH_2Cl_2 as the eluent (100:1→50:1→20:1) afforded enantiopure (*S*)-**3a** (16.8 mg, 47% yield) as an orange oil and enantiopure (*S*)-**1a** (19.3 mg, 48% yield) as a pale yellow oil.

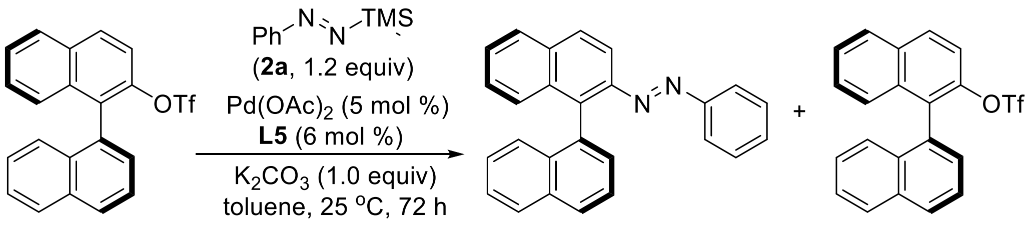

(*R*)-**1a**, 0.1 mmol (*R*)-**3a** 6%, >99% ee (*R*)-**1a** 75%, >99% ee

According to GP 2: in an argon-filled glovebox, an oven-dried 1.5 mL vial was charged with palladium acetate 5 mol %, **L5** 6 mol % and toluene (0.1 mL). The resulting suspension was stirred for 5 min at room temperature, at which time potassium carbonate (13.4 mg, 0.100 mmol, 1.00 equiv), biaryl trifluoromethanesulfonate (*R*)-**1a** (0.100 mmol, 40.2 mg, 1.00 equiv), (*E*)-1-phenyl-2-(trimethylsilyl)diazene **2a** (0.120 mmol, 21.4 mg, 1.20 equiv) and toluene (0.1 mL) were added. The reaction stirred for 72 h at 25 °C. The reaction was quenched by filtration over silica, and flash column chromatography on silica gel using *n*-pentane and CH_2Cl_2 as the eluent (100:1→50:1→20:1) afforded enantiopure (*R*)-**3a** (2.2 mg, 6% yield) as an orange oil and enantiopure (*R*)-**1a** (30.1 mg, 75% yield) as a white solid.

6 Photophysical Properties

6.1 Photoinduced *trans*-to-*cis* interconversion in different solvents

(1) Photoisomerization experiment studies of 3aa by ^{1}H NMR analysis (in C_6D_6)

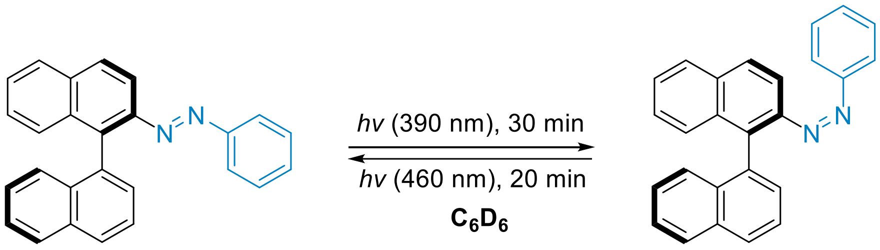

trans-(*S*)-**3aa**, *trans*/*cis* = 91/9, 90% ee
trans/*cis* = 83/17(after 460 nm irridiation)

cis-(*S*)-**3aa,** *trans*/*cis* = 18/82, 89% ee

3aa (*trans*/*cis* = 91/9) was dissolved in deuterated benzene (C_6D_6, 0.06 M) and the solution was transferred into an NMR tube. The tube was then irradiated with 390 nm light for 30 minutes. Analysis by ^{1}H NMR spectroscopy revealed a *trans*/*cis* ratio of 18/82. Subsequently, the same NMR tube was irradiated with 460 nm light for an additional 20 minutes. ^{1}H NMR analysis after this second irradiation indicated a *trans*/*cis* ratio of 83/17.

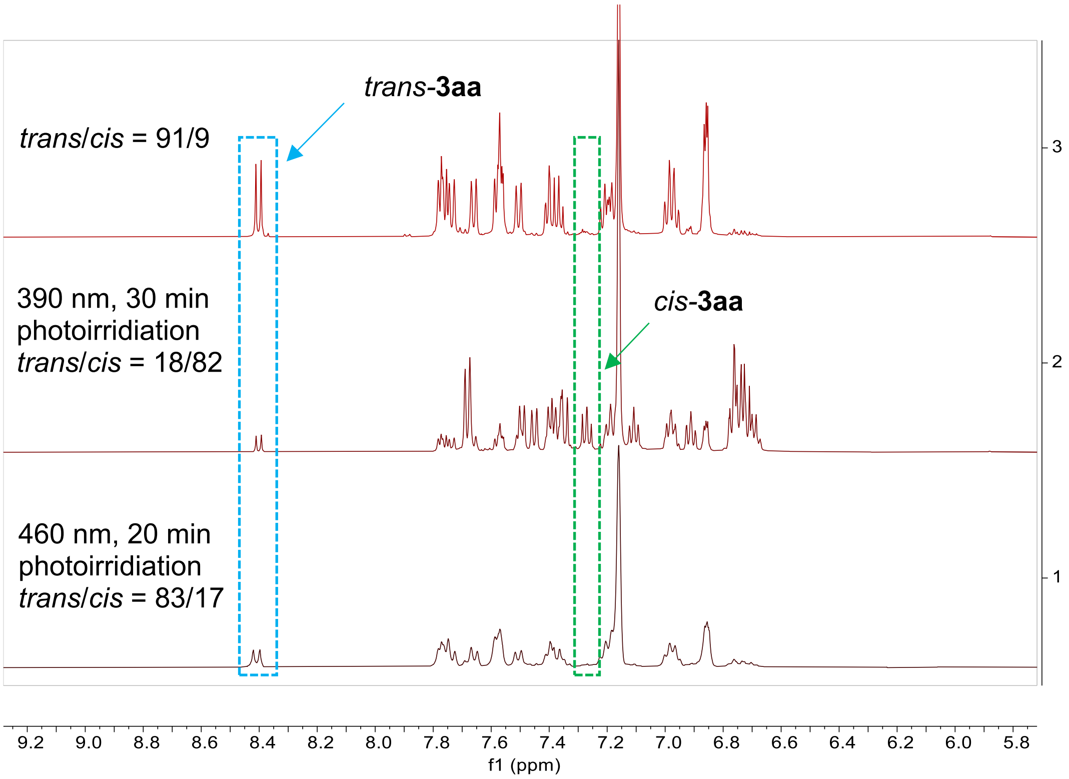

Figure S1. ^{1}H NMR spectra **3aa**: trans/cis = 91/9 (top), *trans*/*cis* = 18/82 (middle) and *trans*/*cis* = 83/7 (bottom).

Analytical data for *cis*-(*S*)-**3aa**

R*f* = 0.35 (CH_2Cl_2) **^{1}H NMR** (400 MHz, Benzene-d_6, 298 K) δ = 7.70–7.66 (m, 2H), 7.52–7.43 (m, 2H), 7.42–7.32 (m, 3H), 7.30–7.24 (m, 1H), 7.22–7.17 (m, 2H), 7.13–7.08 (m, 1H), 7.01–6.95 (m, 1H), 6.94–6.88 (m, 1H), 6.80–6.66 (m, 5H). **^{13}C{^{1}H} NMR** (101 MHz, $CDCl_3$, 298 K) δ = 153.3, 150.1, 133.9, 133.7, 133.6, 132.3, 132.1, 128.9, 128.6, 128.4, 128.2, 128.0, 127.0, 126.7, 126.5, 126.4, 126.3, 126.0, 125.5, 121.1, 118.1. **HRMS** (APCI) m/z: $[M+H]^+$ calcd for $C_{26}H_{19}N_2^+$ 359.1543; found 359.1538. **IR** (ATR): ṽ = 3056, 2924, 1505, 802, 754, 691 cm^{-1}. **Optical Rotation**: $[\alpha]_D^{20}$ = 1684.8 (*c* 0.5, CH_2Cl_2, 89% ee). The enantiomeric excess of (*cis*)-**3aa** was determined by HPLC analysis on a chiral stationary phase (Daicel Chiralcel OD-H column, column temperature 20 °C, solvent *n*-heptane:*i*PrOH = 97:3, flow rate 0.6 mL/min, λ = 280 nm): t_S = 24.9 min (major), t_S = 27.2 min (minor).

(2) Photoisomerization experiment studies of 3aa by ^{1}H NMR analysis (in $CDCl_3$)

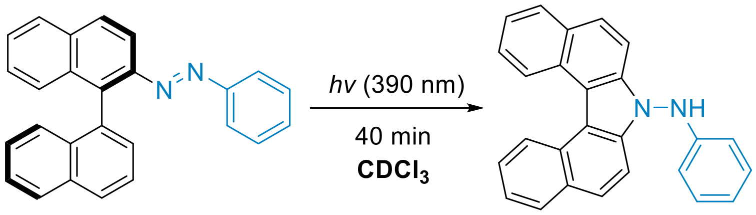

3aa, *trans*/*cis* = 95/5 **4aa**

3aa (trans/cis = 95/5) was dissolved in deuterated chloroform ($CDCl_3$, 0.056 M) and the solution was transferred into an NMR tube. The tube was then irradiated with 390 nm light for 40 minutes. Analysis by ^{1}H NMR spectroscopy revealed that *N*-phenyl-7*H*-dibenzo[*c*,*g*]carbazol-7-amine **4aa** had formed quantitatively.

Analytical data for **4aa**

R*f* = 0.40 (*n*-hexane/CH_2Cl_2 = 1/1) **M.p.**: 172–179 °C **^{1}H NMR** (400 MHz, $CDCl_3$, 298 K) δ = 9.26 (d, *J* = 8.5 Hz, 2H), 8.04 (dd, *J* = 8.1, 1.5 Hz, 2H), 7.83 (d, *J* = 8.7 Hz, 2H), 7.78–7.70 (m, 2H), 7.63 (d, *J* = 8.7 Hz, 2H), 7.61–7.53 (m, 2H), 7.20–7.13 (m, 2H), 6.94–6.86 (m, 1H), 6.72–6.69 (br, 1H), 6.55–6.48 (m, 2H). **^{13}C{^{1}H} NMR** (101 MHz, $CDCl_3$, 298 K) δ = 146.8, 137.2, 130.5, 129.7, 129.5, 129.2, 127.3, 125.8, 125.4, 123.6, 121.5, 115.9, 112.7, 110.8. **HRMS** (APCI) m/z: $[M+H]^+$ calcd for $C_{26}H_{29}N_2^+$ 359.1543; found 359.1546. **IR** (ATR): ṽ = 3336, 1586, 1492, 1375, 1308, 1244, 791, 752, 690 cm^{-1}.

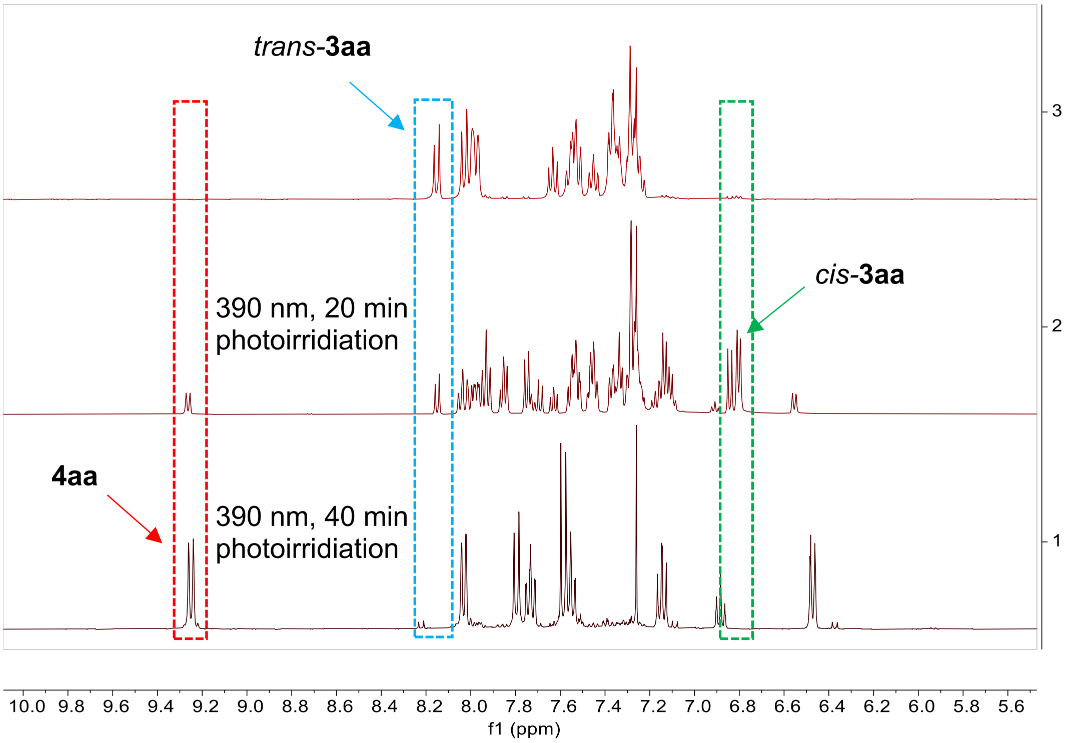

Figure S2. ^{1}H NMR spectra: *trans*-**3aa** (top), mixture of *trans*-**3aa**/*cis*-**3aa**/**4aa** (middle) and **4aa** (bottom).

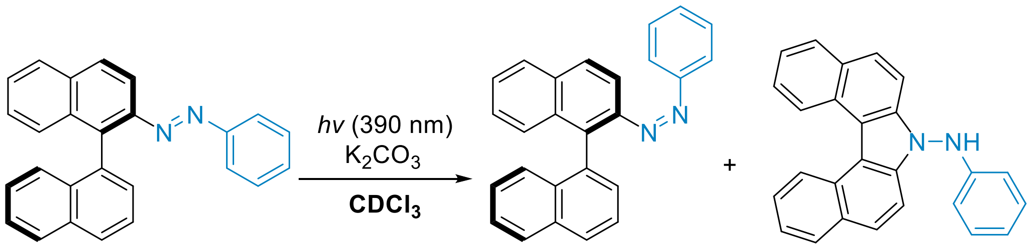

3aa (*trans*/*cis* = 88/12, 1.0 equiv, 10 mg) was dissolved in deuterated chloroform ($CDCl_3$). K_2CO_3 (10.0 equiv, 38.5 mg) was added to the solution. The suspension was stirred and irradiated with 390 nm light for 40 minutes. The crude mixture was filtered through cotton. Analysis by ^{1}H NMR spectroscopy revealed a **3aa** *trans*/*cis* ratio of 26/74, and no **4aa** was observed. This result indicates a proton-initiated intramolecular electrophilic aromatic substitution process.

6.2 UV-VIS Absorption Spectra

UV-vis spectra were measured on PerkinElmer spectrophotometer Lambada 25 employing a scan rate of 480 nm/min. All measurements were made at 25 °C

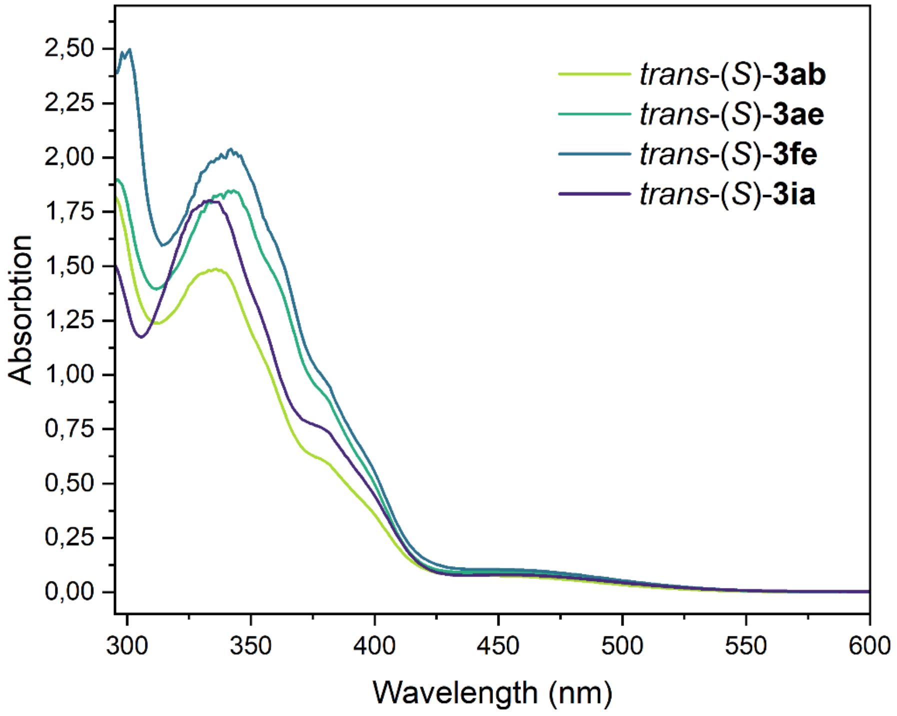

Figure S3. UV-vis absorption spectra of products (in toluene, 100 μmol).

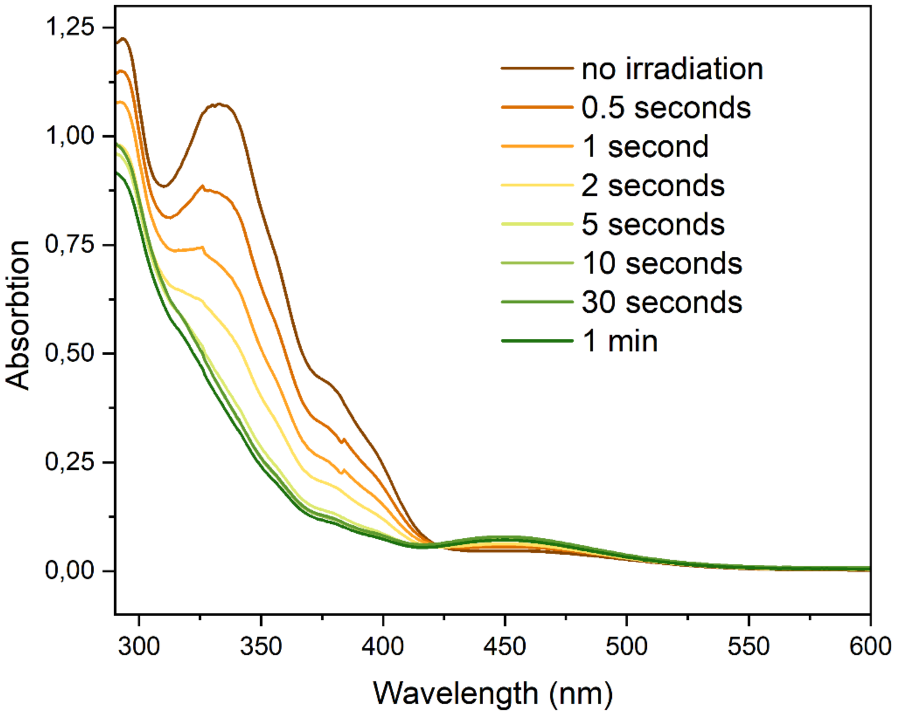

Figure S4. UV-vis absorption spectra of **3aa** (in toluene, 100 μmol) under UV irradiation (390 nm at 0 s, 0.5 s, 1 s, 2 s, 5 s, 10 s, 30 s, and 1 min).

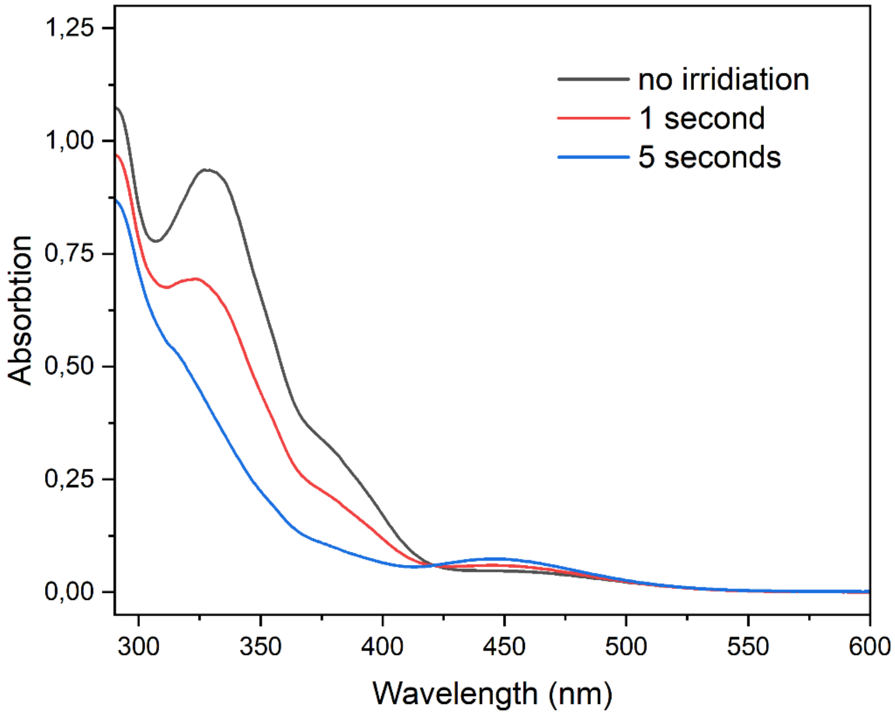

Figure S5. UV-vis absorption spectra of **3aa** (in MeCN, 100 *μ*mol) under UV irradiation (390 nm at 0 s, 1 s, and 5 s).

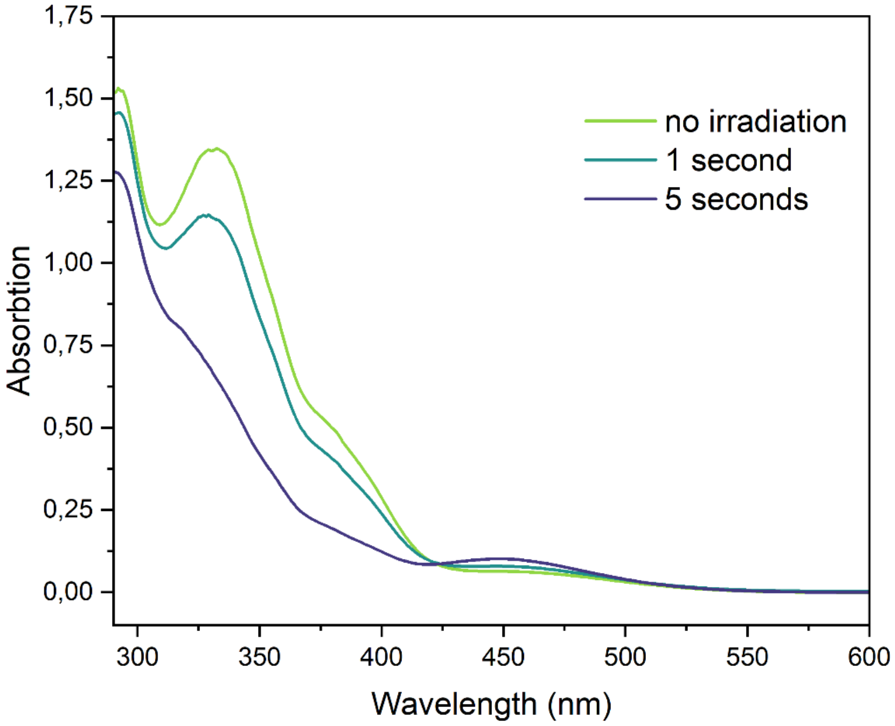

Figure S6. UV-vis absorption spectra of **3aa** (in CH_2Cl_2, 100 *μ*mol) under UV irradiation (390 nm at 0 s, 1 s, and 5 s).

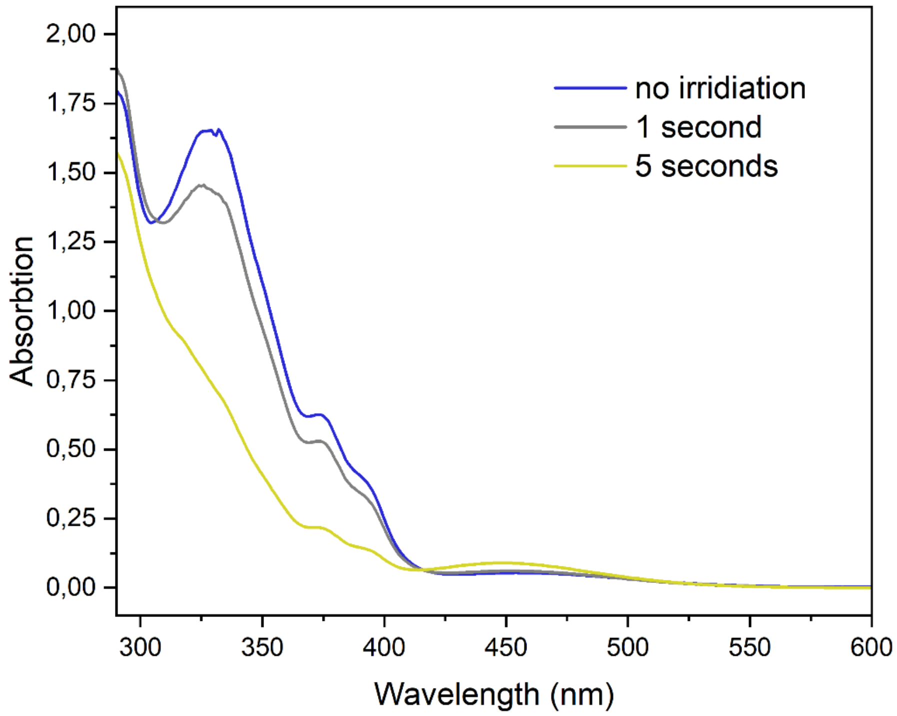

Figure S7. UV-vis absorption spectra of **3aa** (in *n*-hexane, 100 μmol) under UV irradiation (390 nm at 0 s, 1 s, and 5 s).

7 Determination of the Absolute Configuration

According to **5.1**, (*S*)-**1a** was synthesized from (*R*)-BINOL, and (*R*)-**1a** was synthesized from (*S*)-BINOL, respectively. The absolute configuration was assigned as *S* for **3aa**, *R* for **1a** by comparison of the HPLC traces of the recovered binaphthyl triflate **1a** with independently prepared samples of (*R*)-**1a** and (*S*)-**1a**.

rac-**1a**

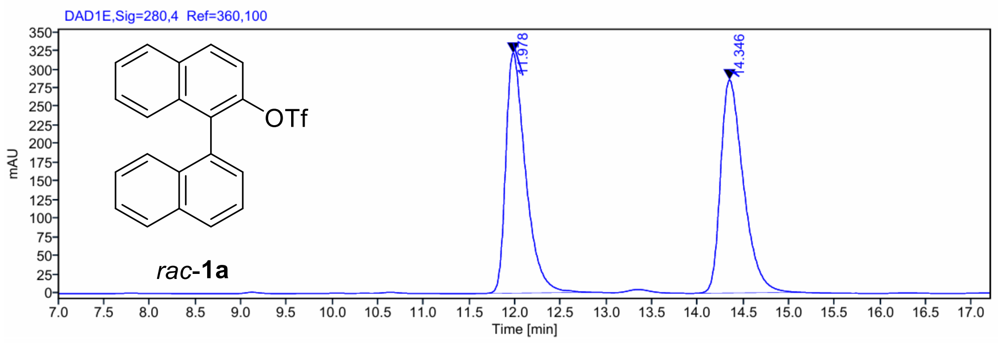

Signal:	DAD1E,Sig=280,4 Ref=360,100			
**RT [min]**	**Width [min]**	**Area**	**Height**	**Area%**
11.978	1.26	4855.94	321.28	49.46
14.346	1.22	4961.76	285.40	50.54
	**Sum**	**9817.70**		

(*S*)-**1a**

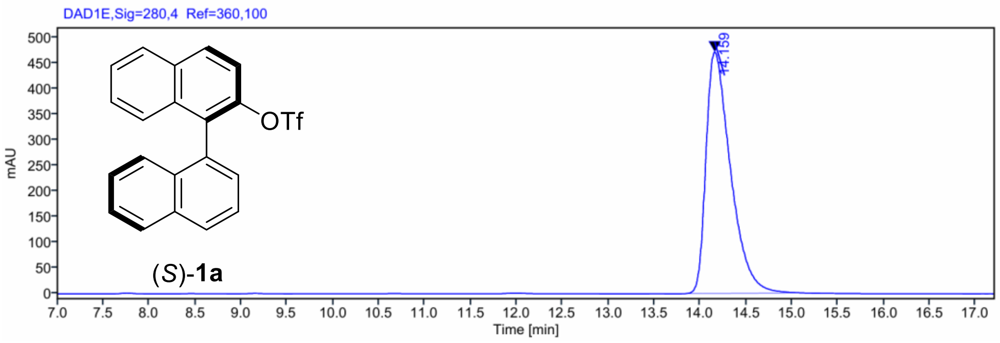

Signal:	DAD1E,Sig=280,4 Ref=360,100			
**RT [min]**	**Width [min]**	**Area**	**Height**	**Area%**
14.159	1.29	8776.14	471.95	100.00
	**Sum**	**8776.14**		

(*R*)-**1a**

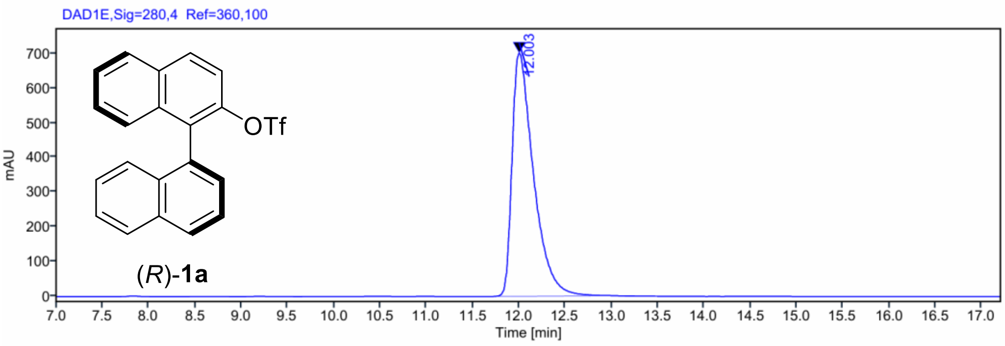

Signal:	DAD1E,Sig=280,4 Ref=360,100			
**RT [min]**	**Width [min]**	**Area**	**Height**	**Area%**
12.003	1.32	11288.89	700.31	100.00
	**Sum**	**11288.89**		

8 HPLC Traces

(*S*)-1-([1,1'-binaphthalen]-2-yl)-2-phenyldiazene [(*S*)-3aa] and (*R*)-[1,1'-binaphthalen]-2-yl trifluoromethanesulfonate [(*R*)-1a]

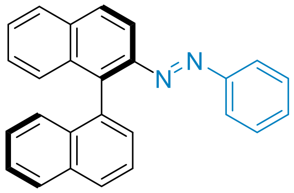

(*S*)-**3aa**

rac-**3aa**

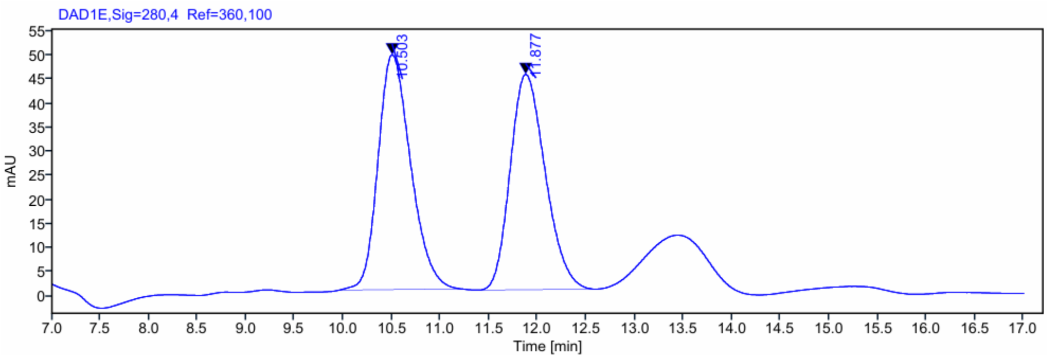

Signal:	DAD1E,Sig=280,4 Ref=360,100			
RT [min]	Width [min]	Area	Height	Area%
10.503	1.27	1139.46	48.74	50.42
11.877	1.18	1120.28	44.70	49.58
	**Sum**	**2259.74**		

(*S*)-**3aa**

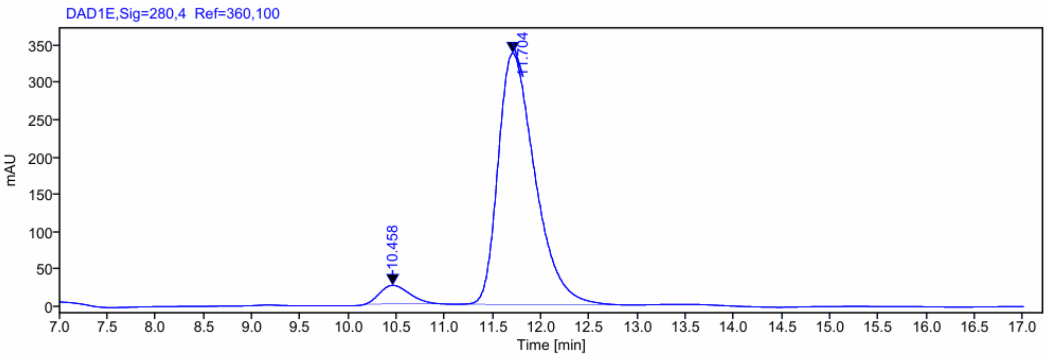

Signal:	DAD1E,Sig=280,4 Ref=360,100			
RT [min]	Width [min]	Area	Height	Area%
10.458	0.74	525.12	24.92	5.55
11.704	1.86	8945.00	336.97	94.45
	**Sum**	**9470.12**		

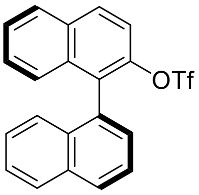

(*R*)-**1a**

rac-**1a**

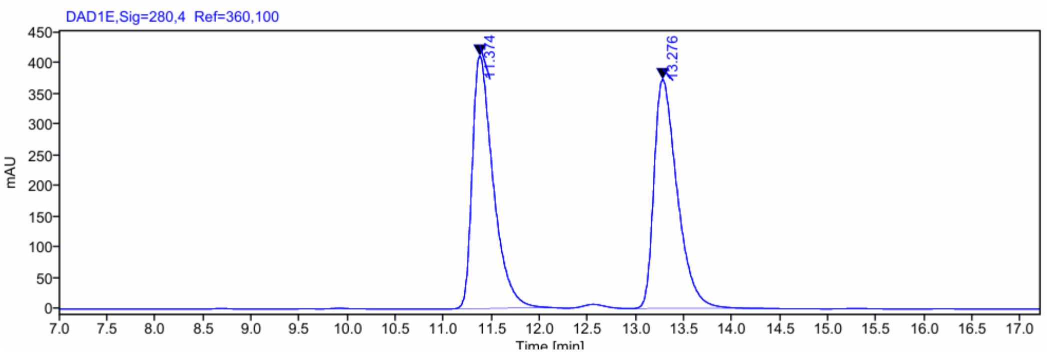

**Signal:**	DAD1E,Sig=280,4 Ref=360,100			
**RT [min]**	**Width [min]**	**Area**	**Height**	**Area%**
11.374	1.03	6205.26	410.78	49.90
13.276	1.26	6230.14	372.25	50.10
	**Sum**	**12435.40**		

(*R*)-**1a**

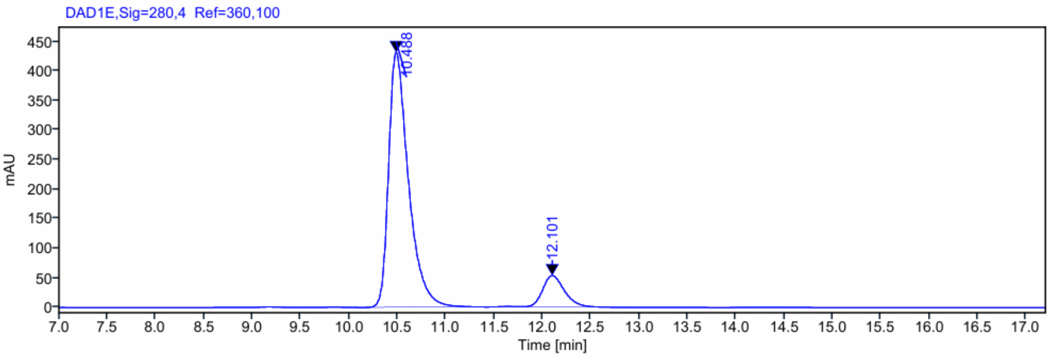

**Signal:**	DAD1E,Sig=280,4 Ref=360,100			
**RT [min]**	**Width [min]**	**Area**	**Height**	**Area%**
10.488	1.14	6127.27	430.85	88.28
12.101	0.77	813.60	52.77	11.72
	**Sum**	**6940.86**		

(*S*)-1-([1,1'-binaphthalen]-2-yl)-2-(4-fluorophenyl)diazene [(*S*)-3ab] and (*R*)-[1,1'-binaphthalen]-2-yl trifluoromethanesulfonate [(*R*)-1a]

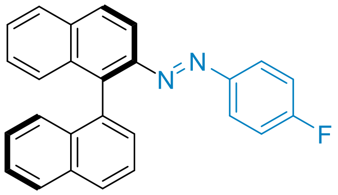

(*S*)-**3ab**

rac-**3ab**

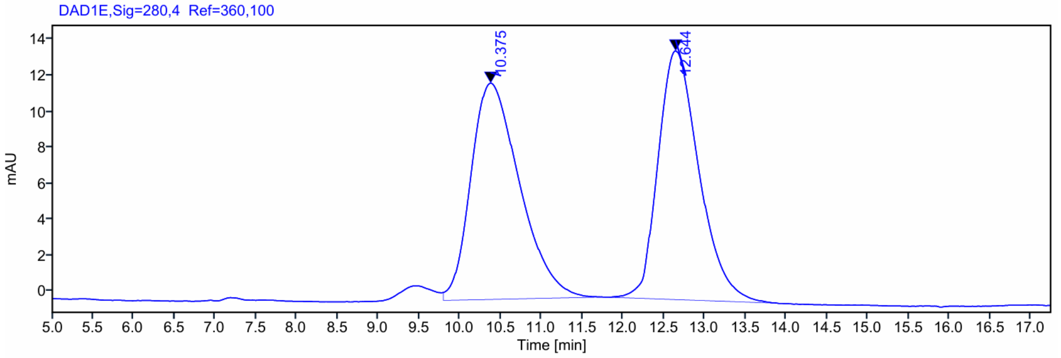

Signal:	DAD1E,Sig=280,4 Ref=360,100			
**RT [min]**	**Width [min]**	**Area**	**Height**	**Area%**
10.375	1.97	500.74	12.04	50.88
12.644	2.13	483.44	13.84	49.12
	**Sum**	**984.18**		

(*S*)-**3ab**

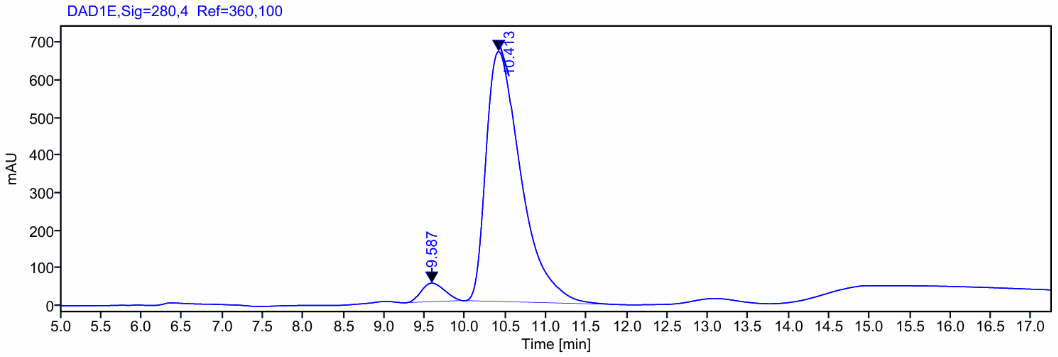

Signal:	DAD1E,Sig=280,4 Ref=360,100			
**RT [min]**	**Width [min]**	**Area**	**Height**	**Area%**
9.587	0.73	991.43	49.38	4.72
10.413	2.04	20018.75	666.25	95.28
	**Sum**	**21010.19**		

(*S*)-**3ab** (after recrystallization)

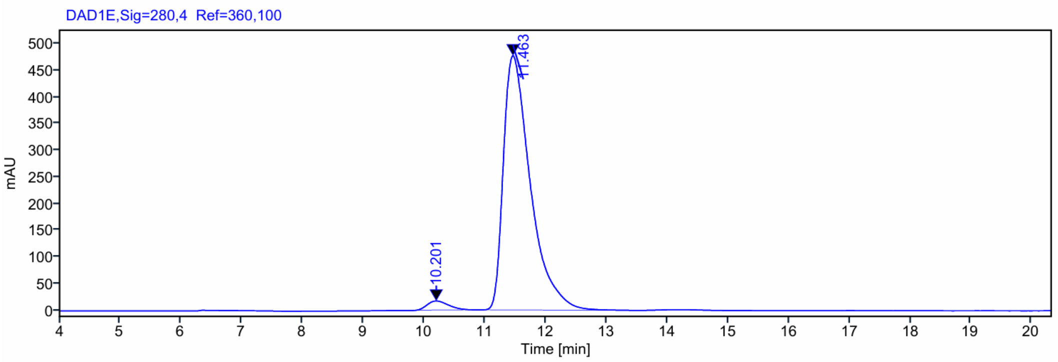

Signal:	DAD1E,Sig=280,4 Ref=360,100			
**RT [min]**	**Width [min]**	**Area**	**Height**	**Area%**
10.201	0.92	407.01	17.00	2.68
11.463	2.72	14759.65	477.00	97.32
	**Sum**	**15166.66**		

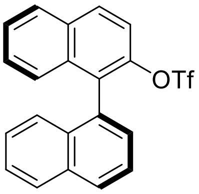

(*R*)-**1a**

rac-**1a**

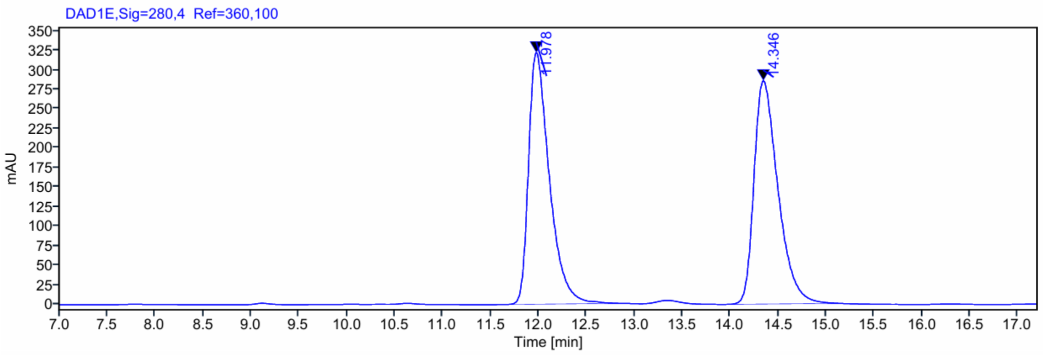

Signal:	DAD1E,Sig=280,4 Ref=360,100			
**RT [min]**	**Width [min]**	**Area**	**Height**	**Area%**
11.978	1.26	4855.94	321.28	49.46
14.346	1.22	4961.76	285.40	50.54
	**Sum**	**9817.70**		

(*R*)-**1a**

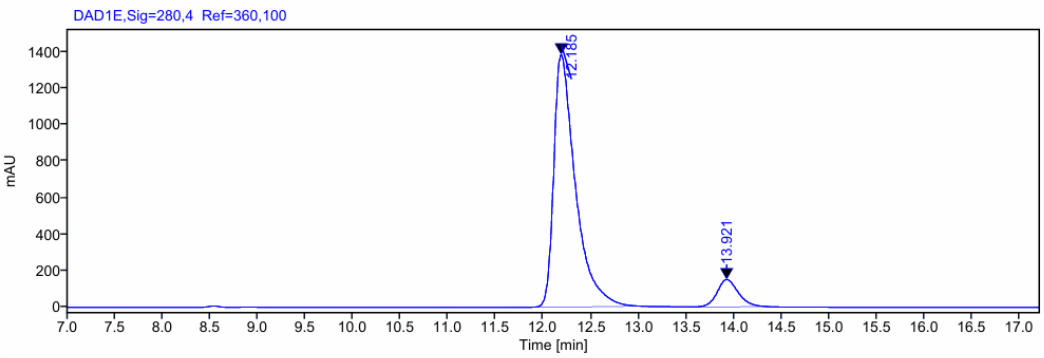

Signal: DAD1E,Sig=280,4 Ref=360,100

RT [min]	Width [min]	Area	Height	Area%
12.185	1.39	21795.42	1380.54	90.51
13.921	0.78	2285.81	148.81	9.49
	**Sum**	**24081.23**		

(*S*)-1-([1,1'-binaphthalen]-2-yl)-2-(4-chlorophenyl)diazene [(*S*)-3ac] and (*R*)-[1,1'-binaphthalen]-2-yl trifluoromethanesulfonate [(*R*)-1a]

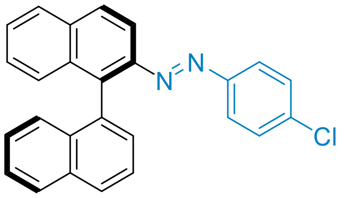

(*S*)-**3ac**

rac-**3ac**

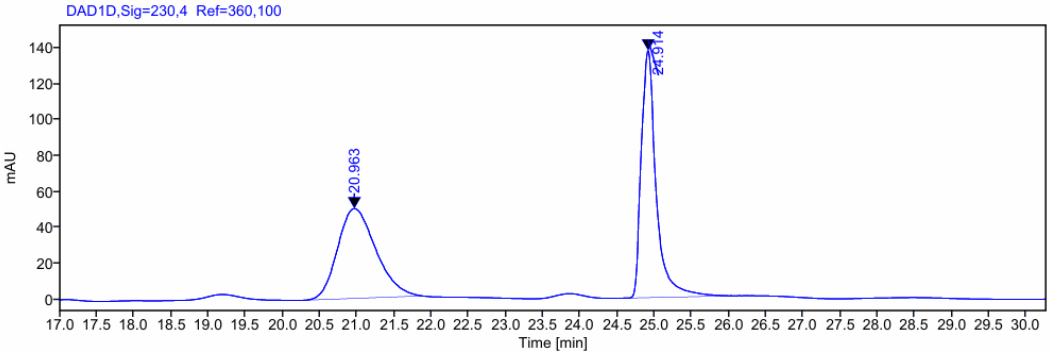

Signal:	DAD1D,Sig=230,4 Ref=360,100			
**RT [min]**	**Width [min]**	**Area**	**Height**	**Area%**
20.963	1.58	1782.45	49.92	48.19
24.914	1.61	1916.46	137.24	51.81
	**Sum**	**3698.91**		

(*S*)-**3ac**

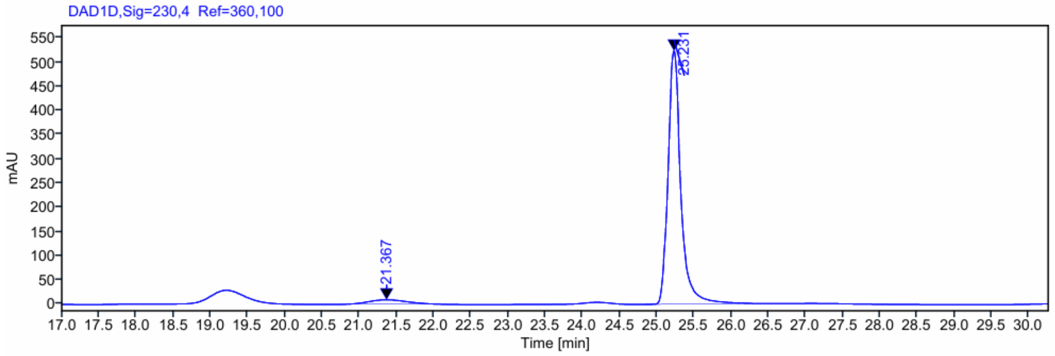

Signal:	DAD1D,Sig=230,4 Ref=360,100			
**RT [min]**	**Width [min]**	**Area**	**Height**	**Area%**
21.367	1.15	291.56	8.60	4.46
25.231	1.40	6239.76	522.54	95.54
	**Sum**	**6531.32**		

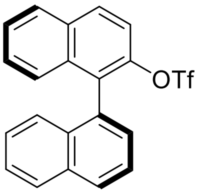

(*R*)-**1a**

rac-**1a**

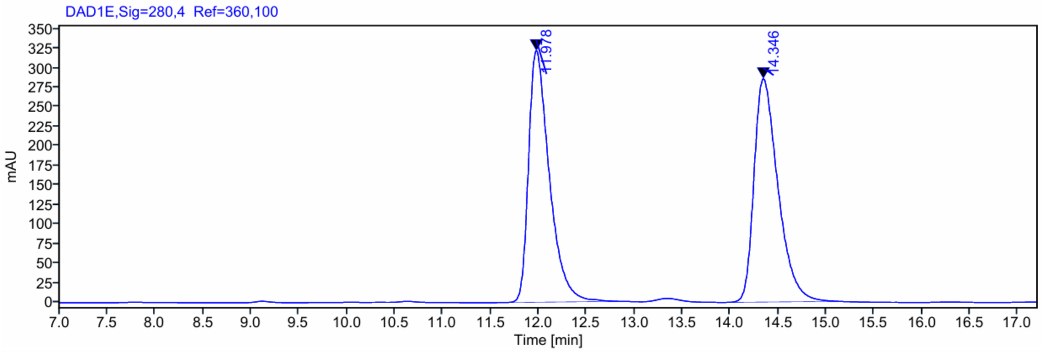

Signal:	DAD1E,Sig=280,4 Ref=360,100			
**RT [min]**	**Width [min]**	**Area**	**Height**	**Area%**
11.978	1.26	4855.94	321.28	49.46
14.346	1.22	4961.76	285.40	50.54
	**Sum**	**9817.70**		

(*R*)-**1a**

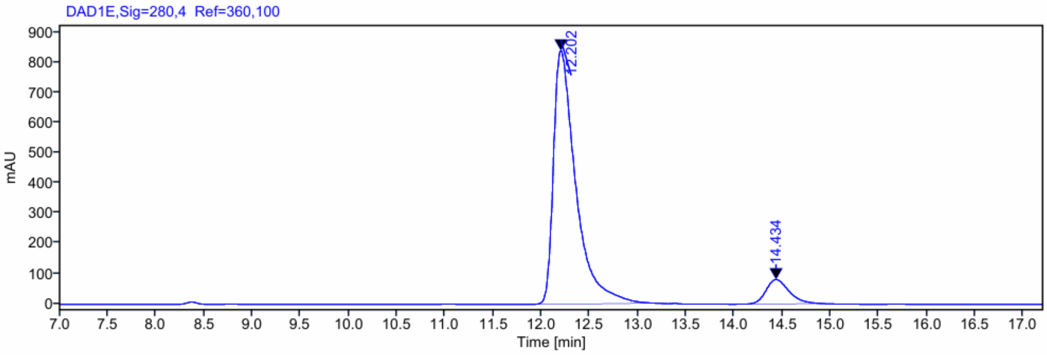

Signal:	DAD1E,Sig=280,4 Ref=360,100			
**RT [min]**	**Width [min]**	**Area**	**Height**	**Area%**
12.202	1.39	13555.82	840.59	91.02
14.434	0.83	1337.33	80.73	8.98
	**Sum**	**14893.15**		

(*S*)-1-([1,1'-binaphthalen]-2-yl)-2-(3-fluorophenyl)diazene [(*S*)-3ad] and (*R*)-[1,1'-binaphthalen]-2-yl trifluoromethanesulfonate [(*R*)-1a]

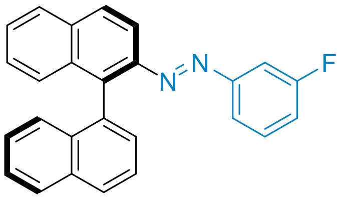

(*S*)-**3ad**

rac-**3ad**

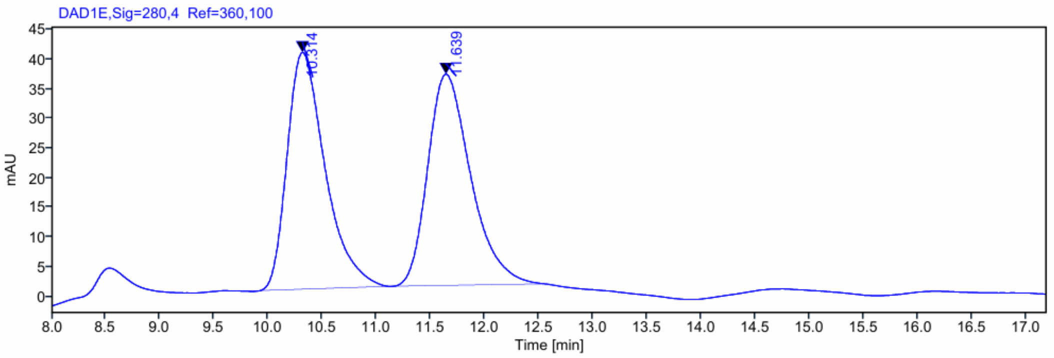

Signal:	DAD1E,Sig=280,4 Ref=360,100			
**RT [min]**	**Width [min]**	**Area**	**Height**	**Area%**
10.314	1.34	973.33	39.88	49.75
11.639	1.44	983.28	35.60	50.25
	**Sum**	**1956.60**		

(*S*)-**3ad**

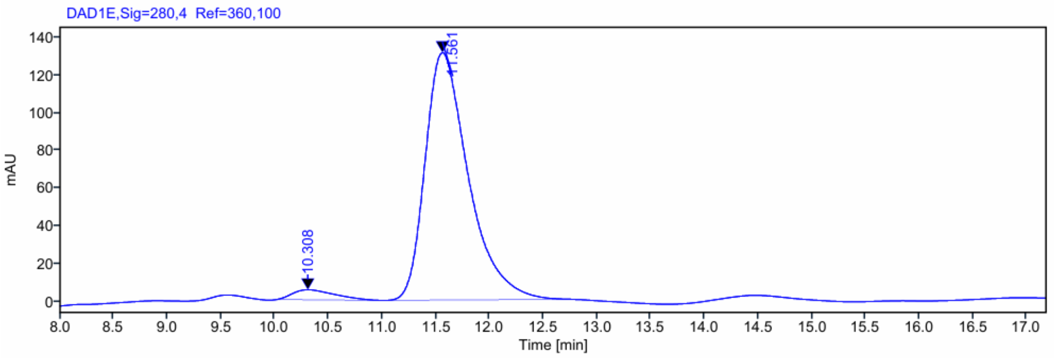

Signal:	DAD1E,Sig=280,4 Ref=360,100			
**RT [min]**	**Width [min]**	**Area**	**Height**	**Area%**
10.308	0.94	144.36	5.31	3.79
11.561	1.84	3669.69	130.94	96.21
	**Sum**	**3814.05**		

(*S*)-**3ad** (after recrystallization)

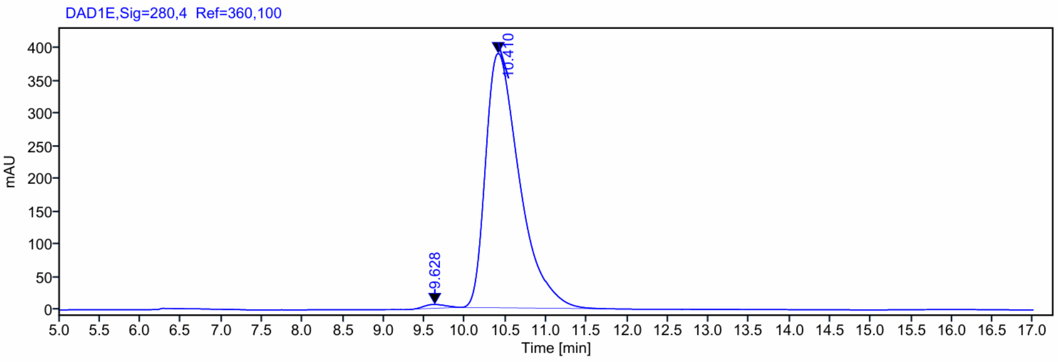

Signal:	DAD1E,Sig=280,4 Ref=360,100			
RT [min]	Width [min]	Area	Height	Area%
9.628	0.57	109.68	5.87	0.96
10.410	2.15	11263.76	388.56	99.04
	Sum	11373.44		

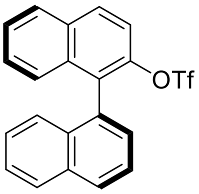

(*R*)-**1a**

rac-**1a**

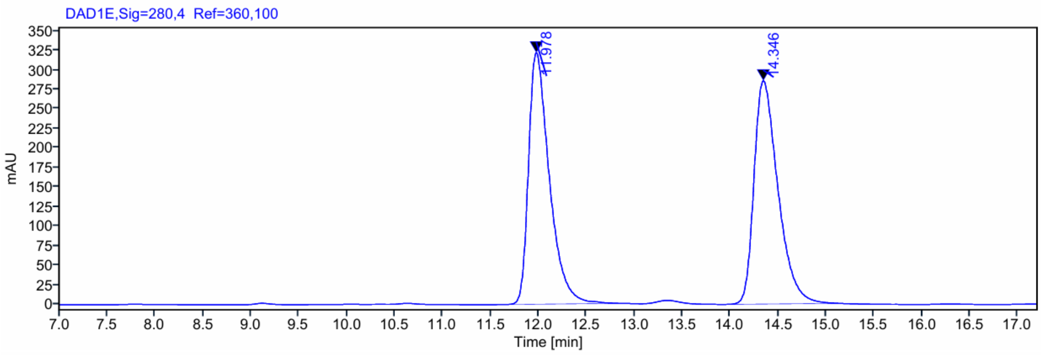

Signal:	DAD1E,Sig=280,4 Ref=360,100			
RT [min]	Width [min]	Area	Height	Area%
11.978	1.26	4855.94	321.28	49.46
14.346	1.22	4961.76	285.40	50.54
	Sum	9817.70		

(*R*)-**1a**

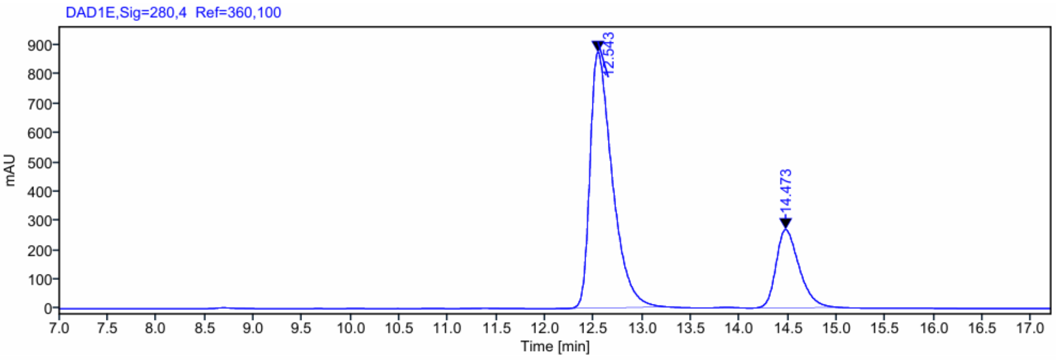

Signal: DAD1E,Sig=280,4 Ref=360,100

RT [min]	Width [min]	Area	Height	Area%
12.543	1.11	13915.64	873.65	75.95
14.473	0.87	4406.52	267.40	24.05
	**Sum**	**18322.16**		

(*S*)-1-([1,1'-binaphthalen]-2-yl)-2-(p-tolyl)diazene [(*S*)-3ae] and (*R*)-[1,1'-binaphthalen]-2-yl trifluoromethanesulfonate [(*R*)-1a]

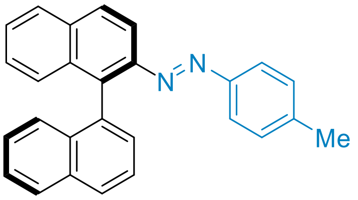

(*S*)-**3ae**

rac-**3ae**

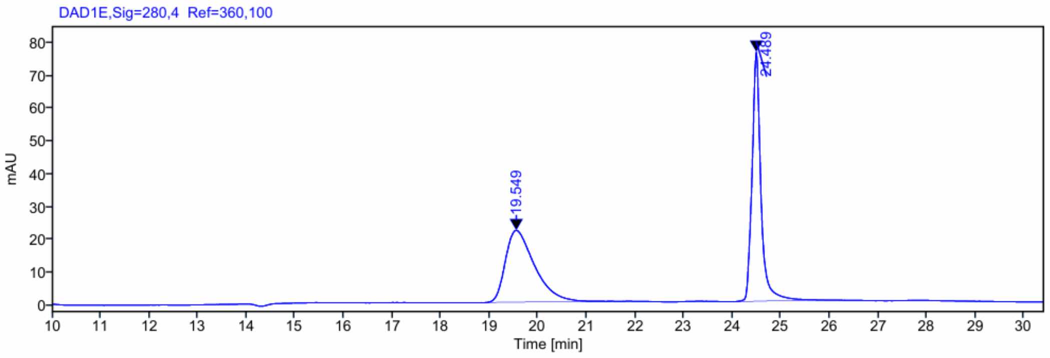

Signal:	DAD1E,Sig=280,4 Ref=360,100			
**RT [min]**	**Width [min]**	**Area**	**Height**	**Area%**
19.549	3.10	909.86	21.88	48.47
24.489	1.82	967.29	76.03	51.53
	**Sum**	**1877.15**		

(*S*)-**3ae**

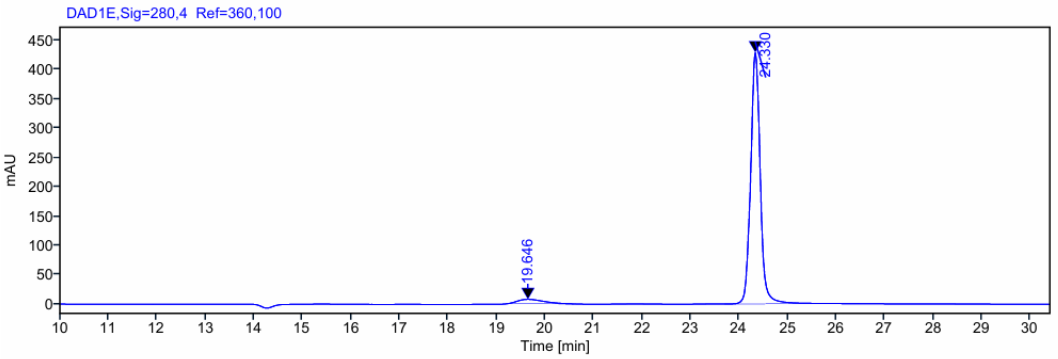

Signal:	DAD1E,Sig=280,4 Ref=360,100			
**RT [min]**	**Width [min]**	**Area**	**Height**	**Area%**
19.646	1.36	288.37	7.64	4.64
24.330	2.77	5922.99	428.36	95.36
	**Sum**	**6211.36**		

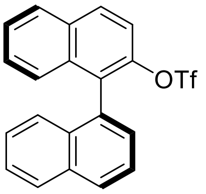

(*R*)-**1a**

rac-**1a**

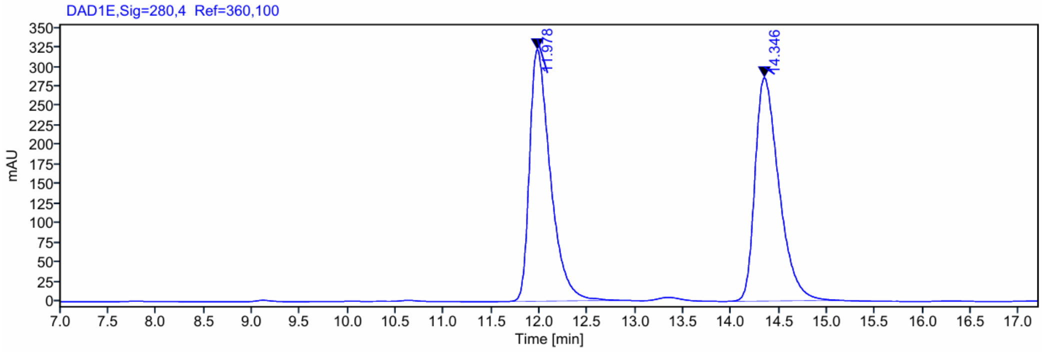

Signal:	DAD1E,Sig=280,4 Ref=360,100			
**RT [min]**	**Width [min]**	**Area**	**Height**	**Area%**
11.978	1.26	4855.94	321.28	49.46
14.346	1.22	4961.76	285.40	50.54
	**Sum**	**9817.70**		

(*R*)-**1a**

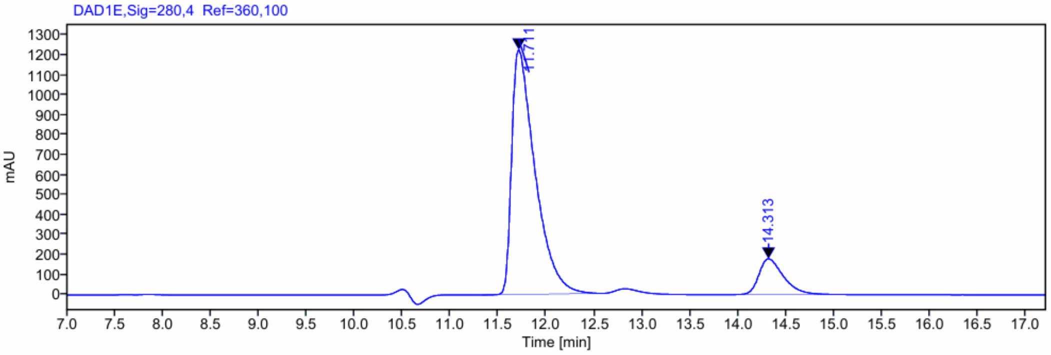

Signal:	DAD1E,Sig=280,4 Ref=360,100			
**RT [min]**	**Width [min]**	**Area**	**Height**	**Area%**
11.711	1.28	20744.28	1226.87	86.75
14.313	0.98	3167.70	178.86	13.25
	**Sum**	**23911.98**		

(*S*)-1-([1,1'-binaphthalen]-2-yl)-2-(4-methoxyphenyl)diazene [(*S*)-3af] and (*R*)-[1,1'-binaphthalen]-2-yl trifluoromethanesulfonate [(*R*)-1a]

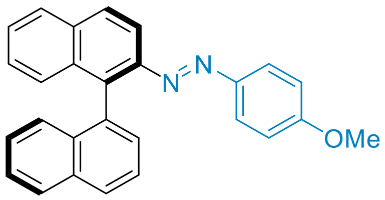

(*S*)-**3af**

rac-**3af**

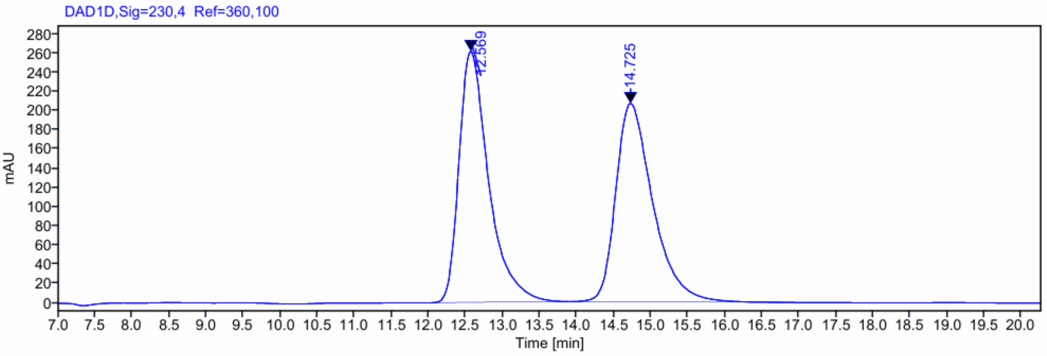

Signal: DAD1D,Sig=230,4 Ref=360,100

RT [min]	Width [min]	Area	Height	Area%
12.569	2.00	7254.14	261.43	50.33
14.725	2.98	7158.56	206.37	49.67
	**Sum**	**14412.71**		

(*S*)-**3af**

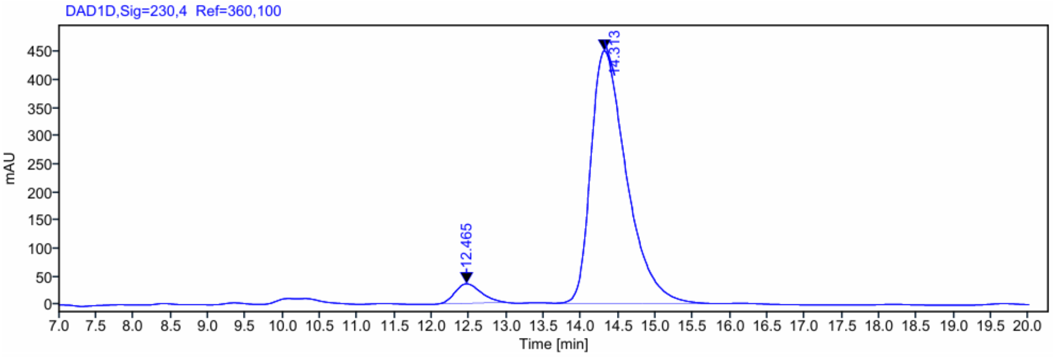

Signal: DAD1D,Sig=230,4 Ref=360,100

RT [min]	Width [min]	Area	Height	Area%
12.465	0.92	843.78	34.79	5.35
14.313	2.24	14938.03	449.21	94.65
	**Sum**	**15781.80**		

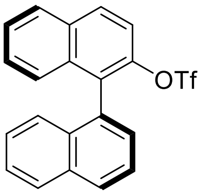

(*R*)-**1a**

rac-**1a**

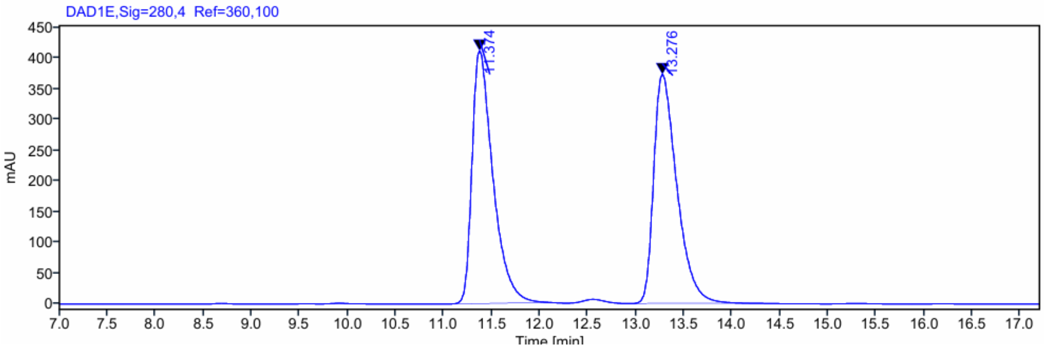

**Signal:**	DAD1E,Sig=280,4 Ref=360,100			
**RT [min]**	**Width [min]**	**Area**	**Height**	**Area%**
11.374	1.03	6205.26	410.78	49.90
13.276	1.26	6230.14	372.25	50.10
	**Sum**	**12435.40**		

(*R*)-**1a**

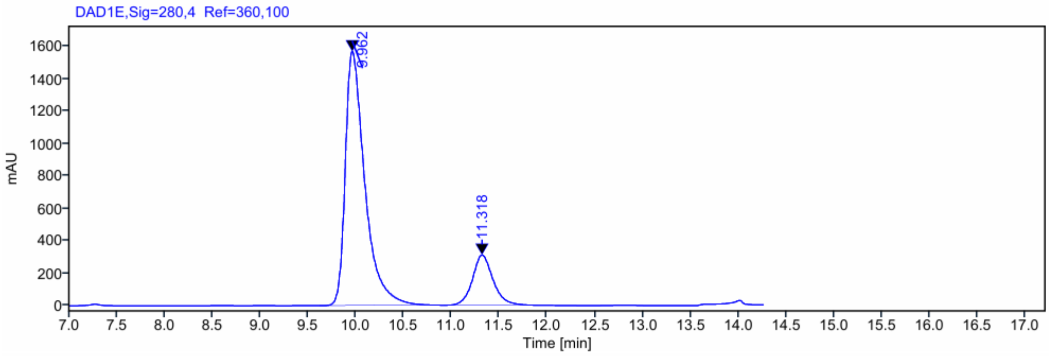

**Signal:**	DAD1E,Sig=280,4 Ref=360,100			
**RT [min]**	**Width [min]**	**Area**	**Height**	**Area%**
9.962	1.10	22176.35	1567.69	82.70
11.318	0.98	4640.26	310.65	17.30
	**Sum**	**26816.61**		

Methyl-(*S*)-4-([1,1'-binaphthalen]-2-yldiazenyl)benzoate [(*S*)-3ag] and (*R*)-[1,1'-binaphthalen]-2-yl trifluoromethanesulfonate [(*R*)-1a]

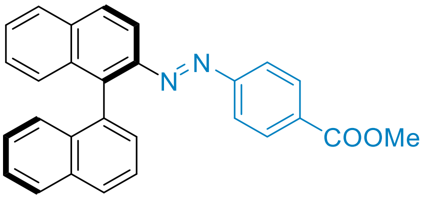

(*S*)-**3ag**

rac-**3ag**

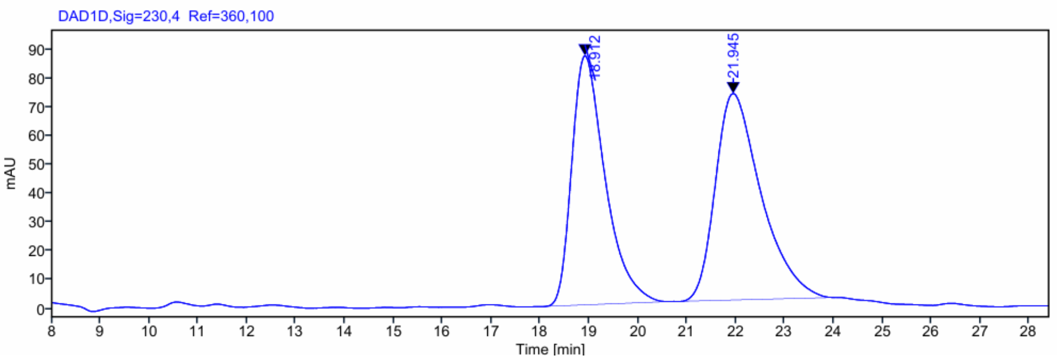

Signal:	DAD1D,Sig=230,4 Ref=360,100			
**RT [min]**	**Width [min]**	**Area**	**Height**	**Area%**
18.912	2.68	4112.81	86.67	46.80
21.945	3.10	4675.44	71.81	53.20
	**Sum**	**8788.25**		

(*S*)-**3ag**

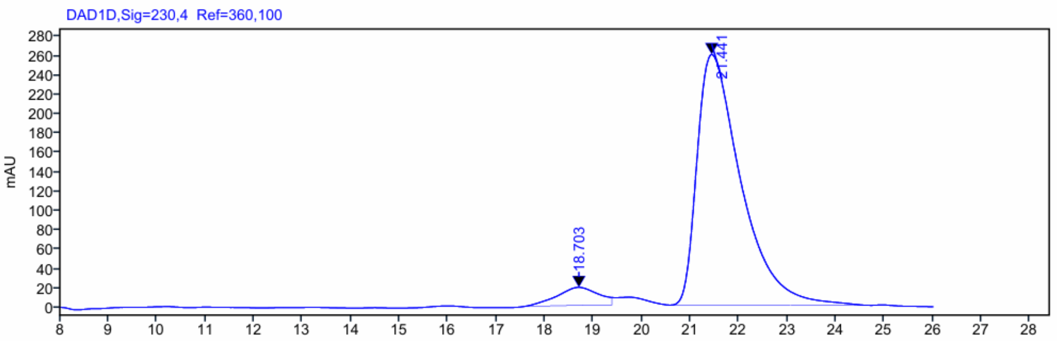

Signal:	DAD1D,Sig=230,4 Ref=360,100			
**RT [min]**	**Width [min]**	**Area**	**Height**	**Area%**
18.703	1.79	1112.95	18.67	6.40
21.441	3.97	16277.21	258.76	93.60
	**Sum**	**17390.16**		

(*S*)-**3ag** (after recrystallization)

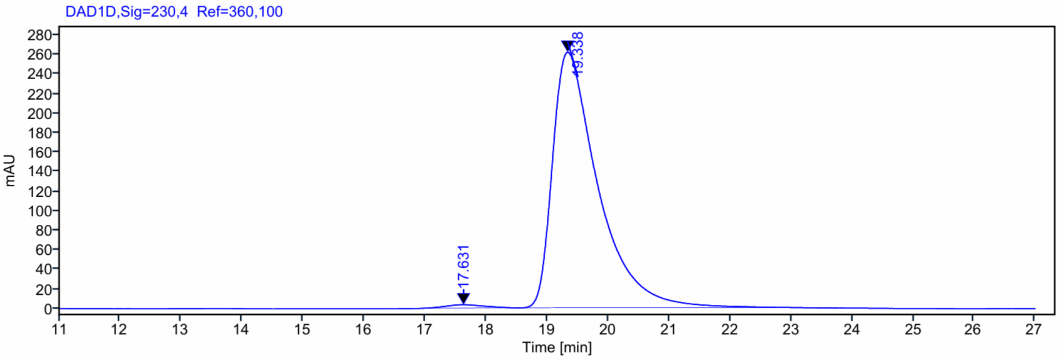

Signal:	DAD1D,Sig=230,4 Ref=360,100			
RT [min]	Width [min]	Area	Height	Area%
17.631	1.45	143.94	3.35	1.05
19.338	4.62	13573.04	262.01	98.95
	Sum	13716.98		

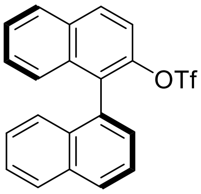

(*R*)-**1a**

rac-**1a**

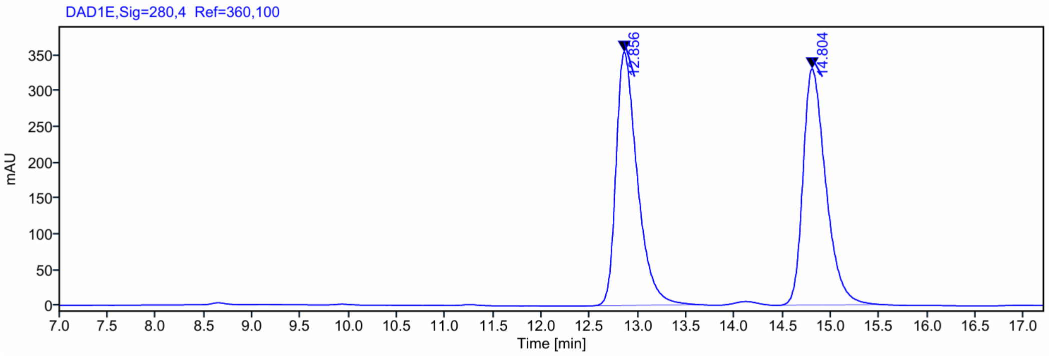

Signal:	DAD1E,Sig=280,4 Ref=360,100			
RT [min]	Width [min]	Area	Height	Area%
12.856	1.22	5449.78	354.39	50.13
14.804	1.06	5422.00	330.58	49.87
	Sum	10871.78		

(*R*)-**1a**

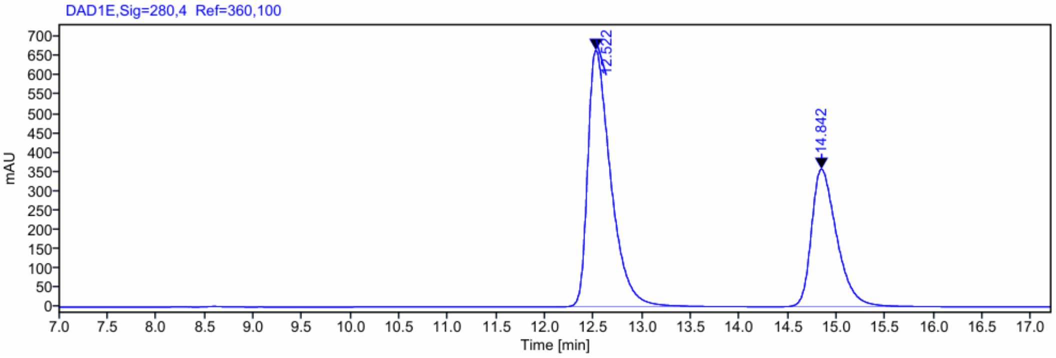

Signal: DAD1E,Sig=280,4 Ref=360,100

RT [min]	Width [min]	Area	Height	Area%
12.522	1.33	10879.49	664.51	62.82
14.842	1.45	6438.36	356.48	37.18
	**Sum**	**17317.85**		

(*S*)-1-(4-fluorophenyl)-2-(6-methoxy-[1,1'-binaphthalen]-2-yl)diazene [(*S*)-3bb] and (*R*)-6-methoxy-[1,1'-binaphthalen]-2-yl trifluoromethanesulfonate [(*R*)-1b]

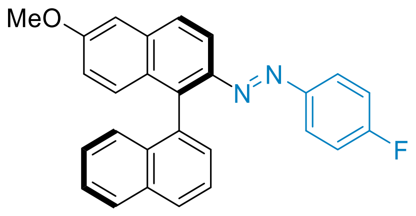

(*S*)-**3bb**

rac-**3bb**

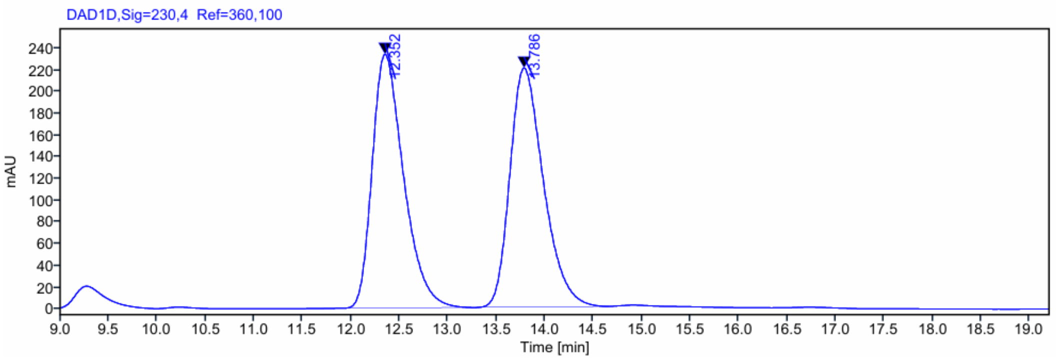

Signal:	DAD1D,Sig=230,4 Ref=360,100			
RT [min]	Width [min]	Area	Height	Area%
12.352	1.37	5255.98	233.55	49.85
13.786	1.46	5287.04	220.00	50.15
	**Sum**	**10543.02**		

(*S*)-**3bb**

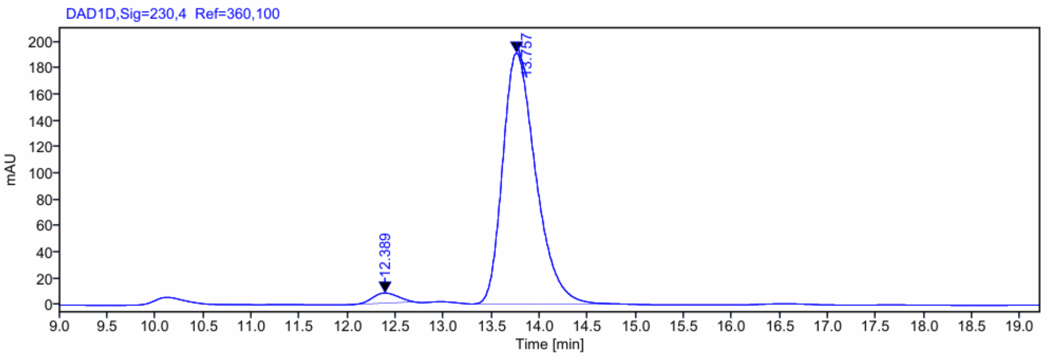

Signal:	DAD1D,Sig=230,4 Ref=360,100			
RT [min]	Width [min]	Area	Height	Area%
12.389	0.65	141.68	7.70	3.01
13.757	1.67	4562.80	190.82	96.99
	**Sum**	**4704.48**		

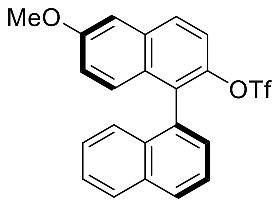

(*R*)-**1b**

rac-**1b**

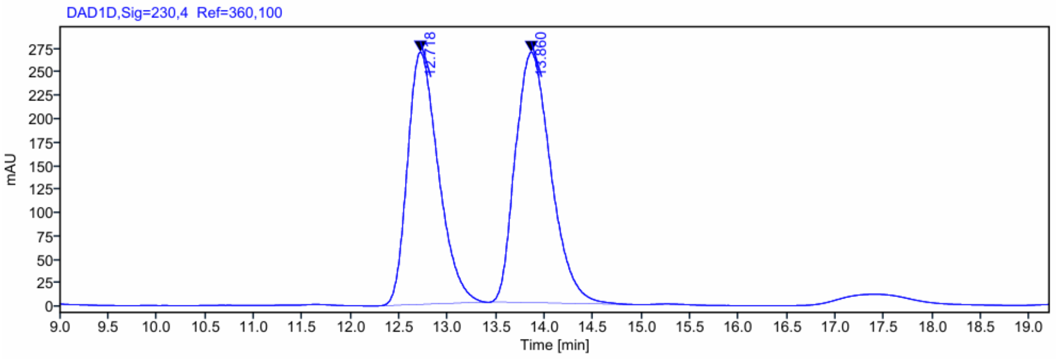

Signal:	DAD1D,Sig=230,4 Ref=360,100			
**RT [min]**	**Width [min]**	**Area**	**Height**	**Area%**
12.718	1.16	5950.49	268.89	46.63
13.860	1.47	6811.46	266.91	53.37
	**Sum**	**12761.95**		

(*R*)-**1b**

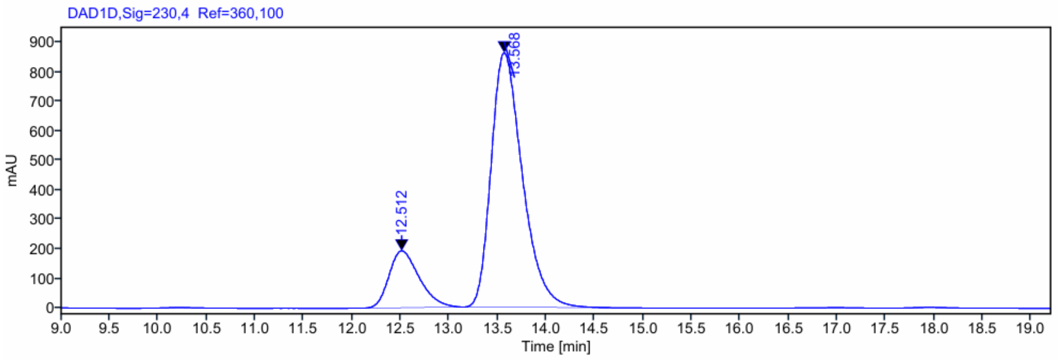

Signal:	DAD1D,Sig=230,4 Ref=360,100			
**RT [min]**	**Width [min]**	**Area**	**Height**	**Area%**
12.512	1.09	4167.59	192.79	17.73
13.568	1.51	19333.30	862.00	82.27
	**Sum**	**23500.89**		

(*S*)-1-phenyl-2-(6-(trimethylsilyl)-[1,1'-binaphthalen]-2-yl)diazene [(*S*)-3ca] and (*R*)-6-(trimethylsilyl)-[1,1'-binaphthalen]-2-yl trifluoromethanesulfonate [(*R*)-1c]

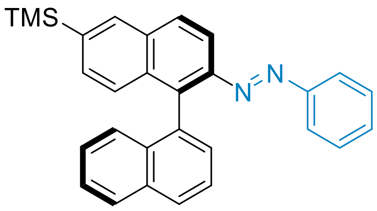

(*S*)-**3ca**

rac-**3ca**

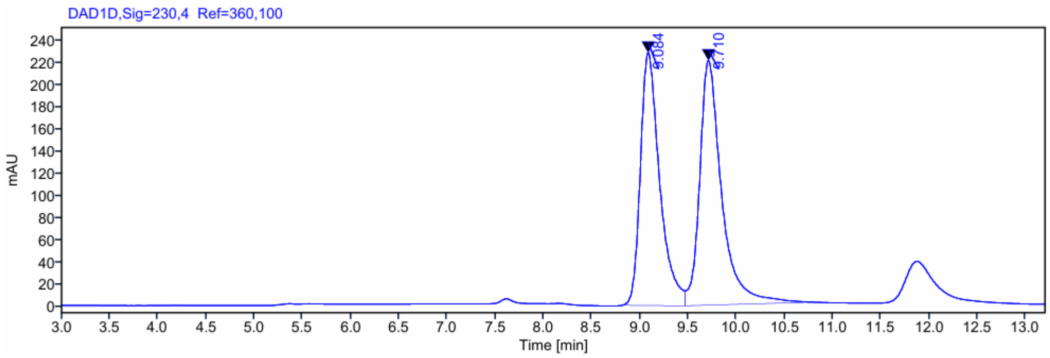

Signal:	DAD1D,Sig=230,4 Ref=360,100			
**RT [min]**	**Width [min]**	**Area**	**Height**	**Area%**
9.084	0.65	3172.43	228.05	47.47
9.710	1.32	3510.78	220.71	52.53
	**Sum**	**6683.21**		

(*S*)-**3ca**

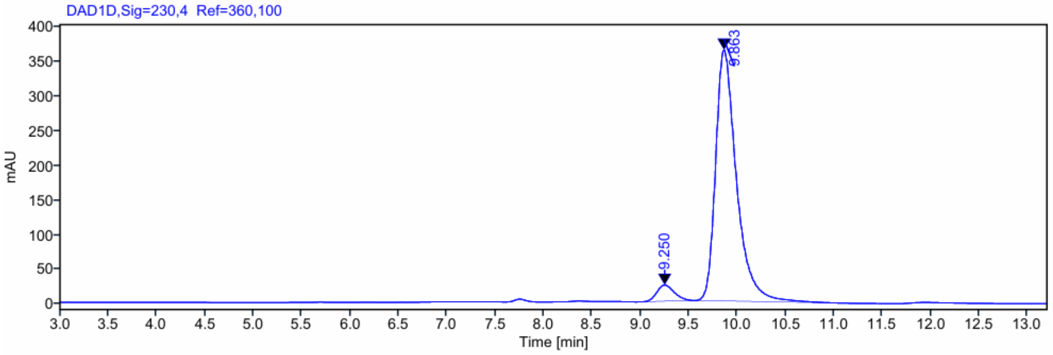

Signal:	DAD1D,Sig=230,4 Ref=360,100			
**RT [min]**	**Width [min]**	**Area**	**Height**	**Area%**
9.250	0.52	287.30	23.19	5.02
9.863	1.46	5437.14	362.28	94.98
	**Sum**	**5724.44**		

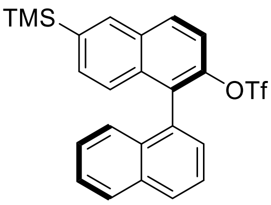

(*R*)-**1c**

rac-**1c**

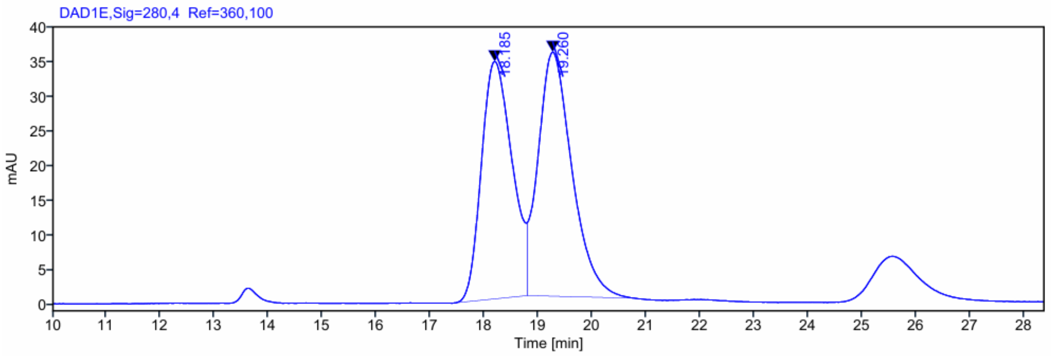

Signal: DAD1E,Sig=280,4 Ref=360,100

RT [min]	Width [min]	Area	Height	Area%
18.185	1.41	1353.82	34.11	46.85
19.260	1.98	1535.92	35.08	53.15
	**Sum**	**2889.75**		

(*R*)-**1c**

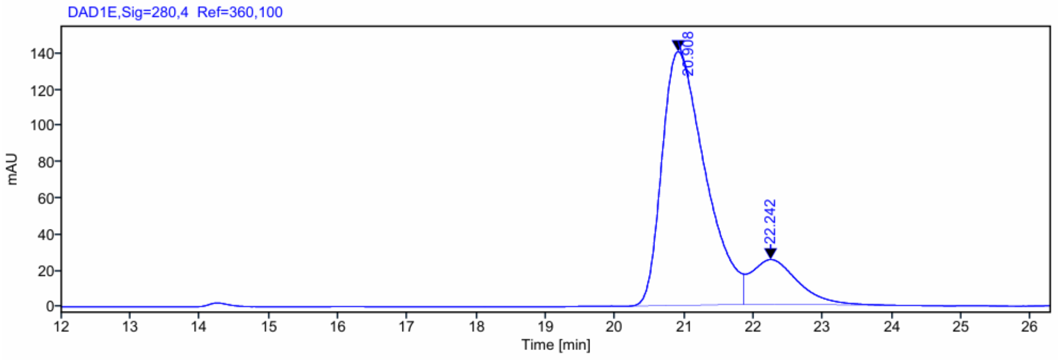

Signal: DAD1E,Sig=280,4 Ref=360,100

RT [min]	Width [min]	Area	Height	Area%
20.908	1.69	6095.18	140.23	83.93
22.242	1.98	1167.20	24.82	16.07
	**Sum**	**7262.38**		

(*S*)-1-(3-methyl-[1,1'-binaphthalen]-2-yl)-2-phenyldiazene [(*S*)-3da] and (*R*)-3-methyl-[1,1'-binaphthalen]-2-yl trifluoromethanesulfonate [(*R*)-1d]

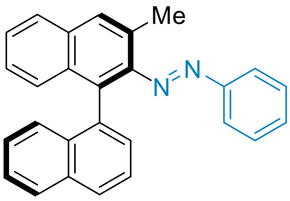

(*S*)-**3da**

rac-**3da**

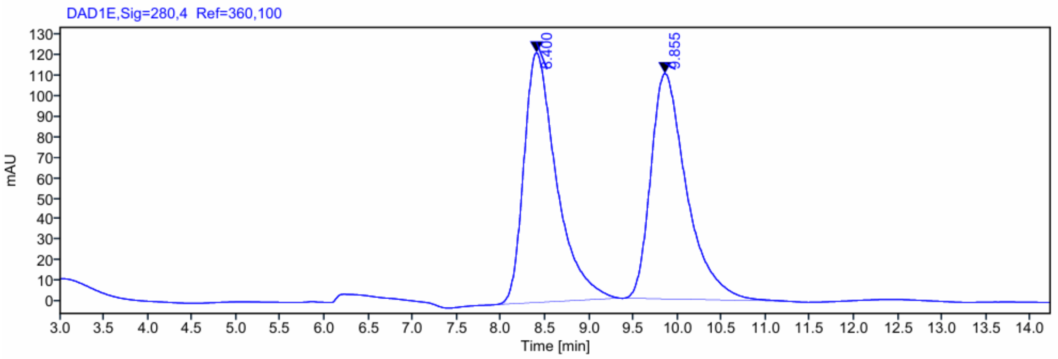

Signal:	DAD1E,Sig=280,4 Ref=360,100			
**RT [min]**	**Width [min]**	**Area**	**Height**	**Area%**
8.400	1.40	3130.14	121.82	50.20
9.855	1.88	3104.99	109.98	49.80
	**Sum**	**6235.13**		

(*S*)-**3da**

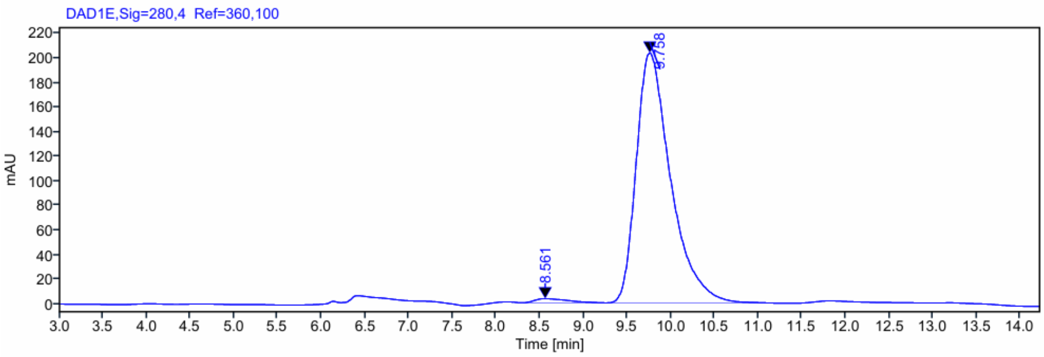

Signal:	DAD1E,Sig=280,4 Ref=360,100			
**RT [min]**	**Width [min]**	**Area**	**Height**	**Area%**
8.561	0.89	84.79	3.35	1.50
9.758	1.87	5556.19	203.24	98.50
	**Sum**	**5640.98**		

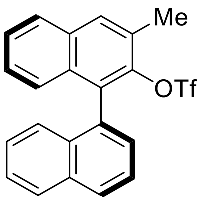

(*R*)-**1d**

rac-**1d**

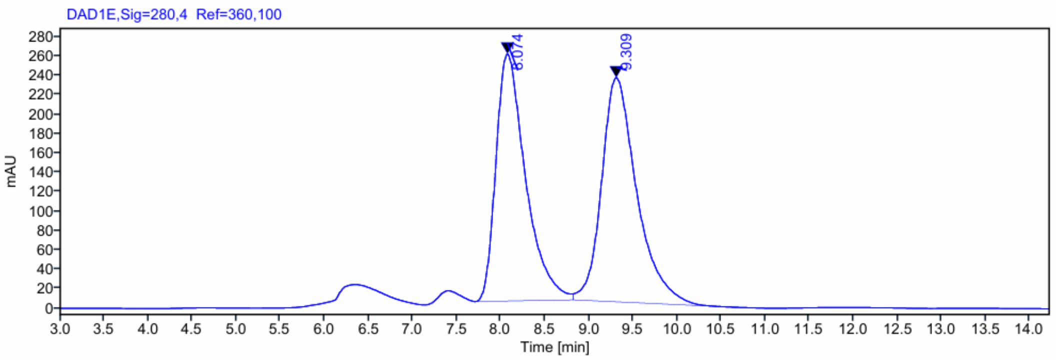

Signal:	DAD1E,Sig=280,4 Ref=360,100			
**RT [min]**	**Width [min]**	**Area**	**Height**	**Area%**
8.074	1.12	5954.45	255.00	48.09
9.309	1.86	6427.96	231.31	51.91
	**Sum**	**12382.41**		

(*R*)-**1d**

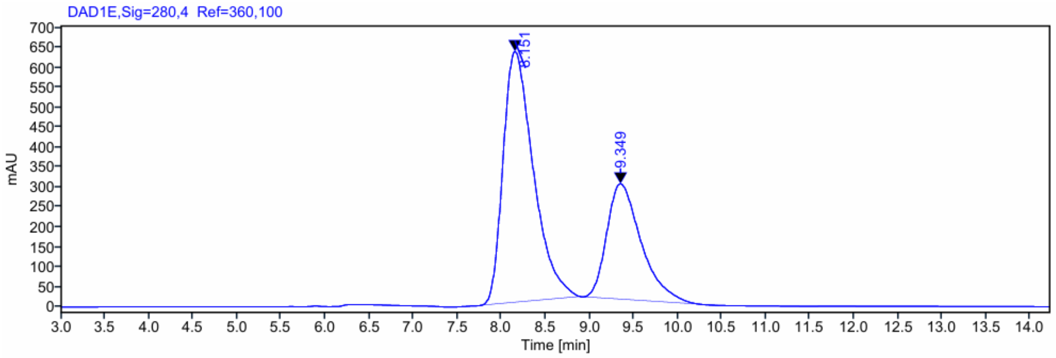

Signal:	DAD1E,Sig=280,4 Ref=360,100			
**RT [min]**	**Width [min]**	**Area**	**Height**	**Area%**
8.151	1.23	15022.25	627.84	65.73
9.349	1.59	7832.45	288.81	34.27
	**Sum**	**22854.69**		

(*S*)-1-(3-methyl-[1,1'-binaphthalen]-2-yl)-2-phenyldiazene [(*S*)-3db] and (*R*)-3-methyl-[1,1'-binaphthalen]-2-yl trifluoromethanesulfonate [(*R*)-1d]

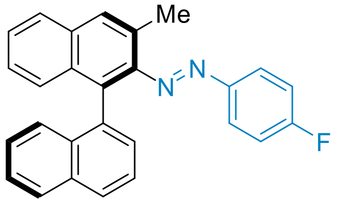

(*S*)-**3db**

rac-**3db**

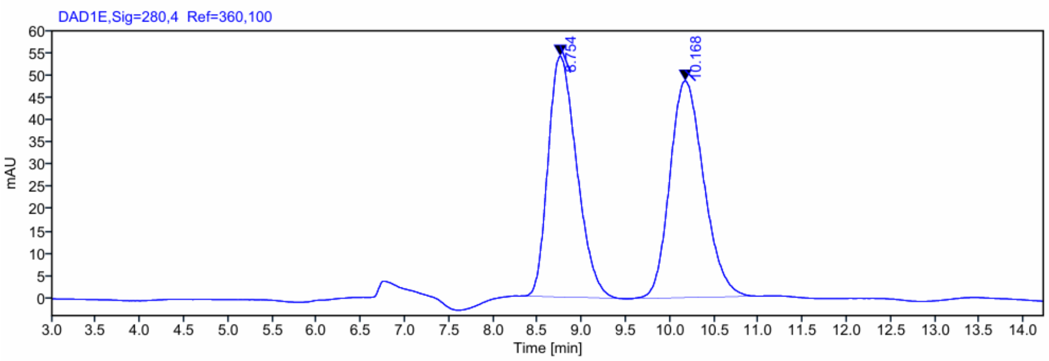

Signal:	DAD1E,Sig=280,4 Ref=360,100			
RT [min]	Width [min]	Area	Height	Area%
8.754	1.27	1225.39	54.25	48.45
10.168	1.63	1303.74	48.81	51.55
	Sum	2529.14		

(*S*)-**3db**

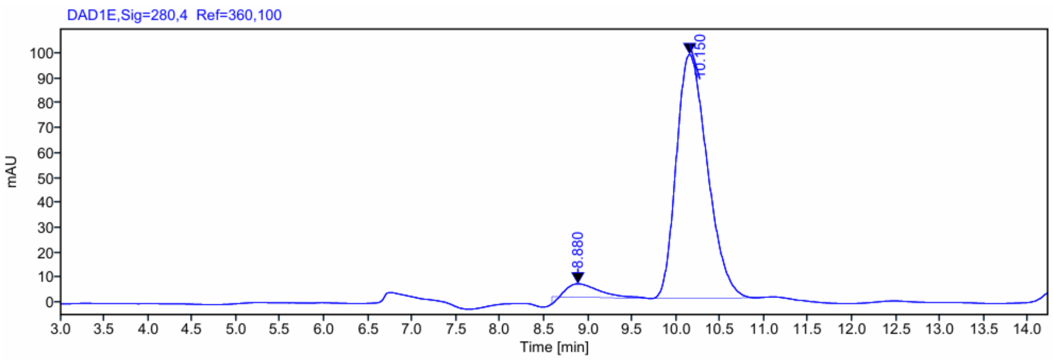

Signal:	DAD1E,Sig=280,4 Ref=360,100			
RT [min]	Width [min]	Area	Height	Area%
8.880	1.13	131.97	5.35	5.21
10.150	1.23	2401.03	97.90	94.79
	Sum	2533.01		

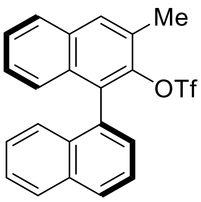

(*R*)-**1d**

rac-**1d**

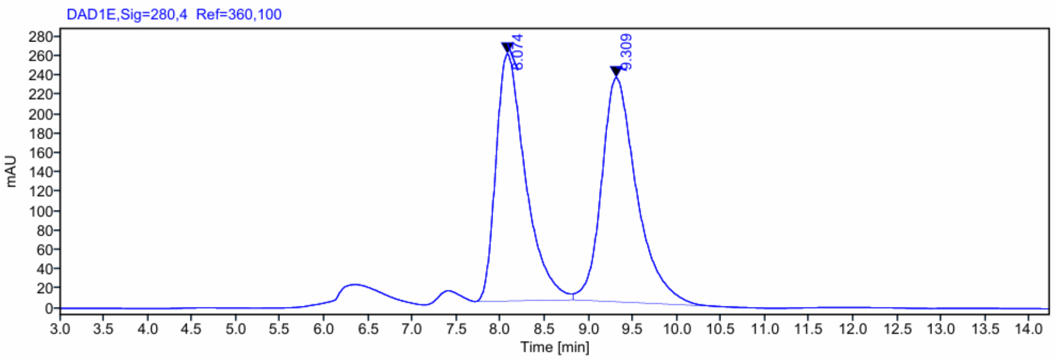

**Signal:**	DAD1E,Sig=280,4 Ref=360,100			
**RT [min]**	**Width [min]**	**Area**	**Height**	**Area%**
8.074	1.12	5954.45	255.00	48.09
9.309	1.86	6427.96	231.31	51.91
	**Sum**	**12382.41**		

(*R*)-**1d**

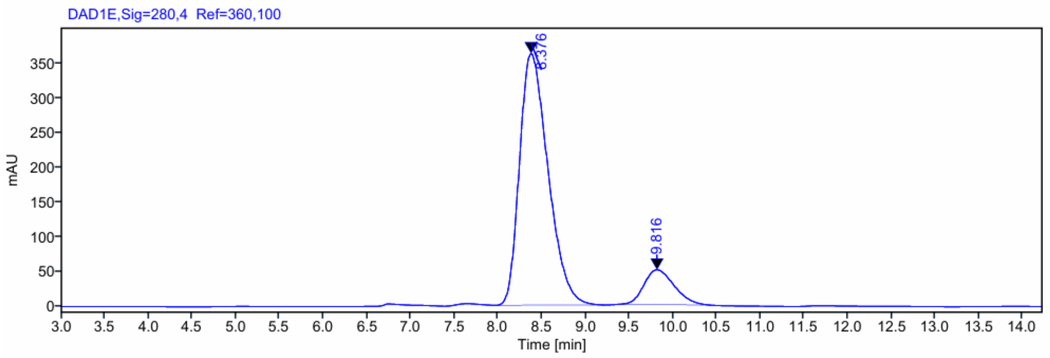

**Signal:**	DAD1E,Sig=280,4 Ref=360,100			
**RT [min]**	**Width [min]**	**Area**	**Height**	**Area%**
8.376	1.22	8190.74	361.30	87.24
9.816	1.03	1197.96	49.88	12.76
	**Sum**	**9388.70**		

(*S*)-1-(3-fluoro-[1,1'-binaphthalen]-2-yl)-2-phenyldiazene [(*S*)-3ea] and (*R*)-3-fluoro-[1,1'-binaphthalen]-2-yl trifluoromethanesulfonate [(*R*)-1e]

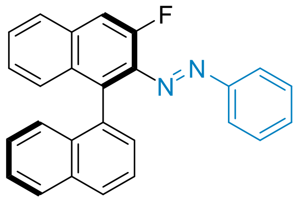

(*S*)-**3ea**

rac-**3ea**

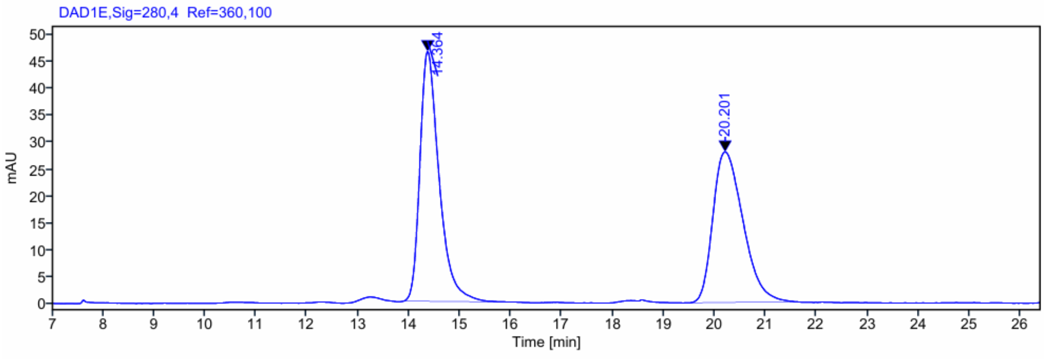

Signal:	DAD1E,Sig=280,4 Ref=360,100			
RT [min]	Width [min]	Area	Height	Area%
14.364	1.96	1214.04	46.40	51.36
20.201	2.13	1149.96	27.96	48.64
	**Sum**	**2364.00**		

(*S*)-**3ea**

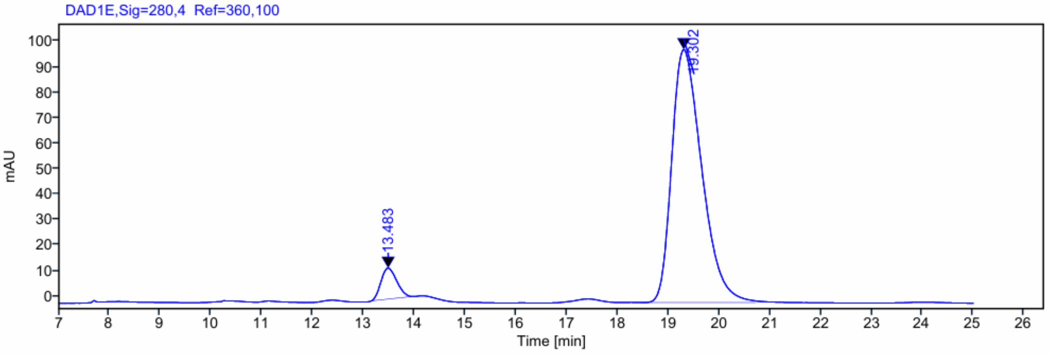

Signal:	DAD1E,Sig=280,4 Ref=360,100			
RT [min]	Width [min]	Area	Height	Area%
13.483	0.80	260.72	12.13	6.00
19.302	2.73	4086.02	99.30	94.00
	**Sum**	**4346.74**		

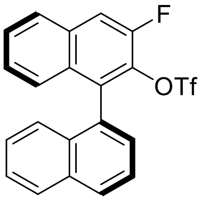

(*R*)-**1e**

rac-**1e**

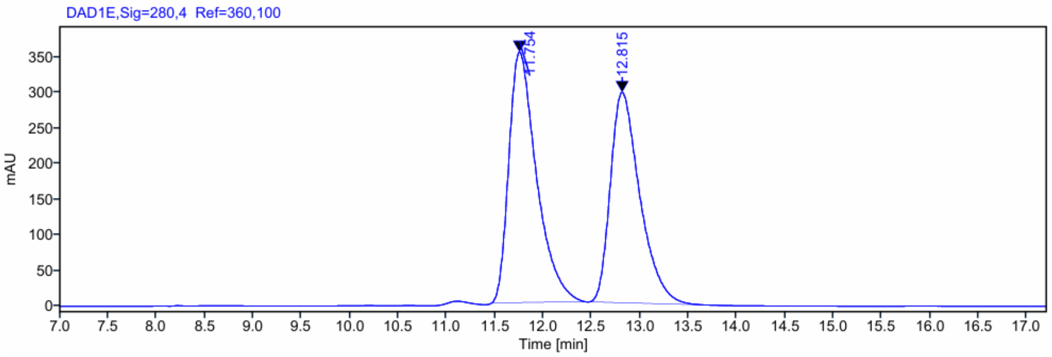

Signal:	DAD1E,Sig=280,4 Ref=360,100			
**RT [min]**	**Width [min]**	**Area**	**Height**	**Area%**
11.754	0.96	6989.82	351.20	52.78
12.815	1.26	6253.21	295.11	47.22
	**Sum**	**13243.04**		

(*R*)-**1e**

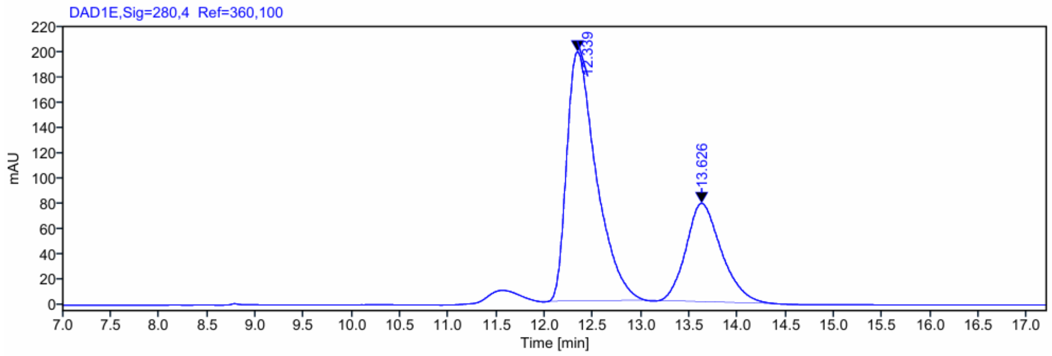

Signal:	DAD1E,Sig=280,4 Ref=360,100			
**RT [min]**	**Width [min]**	**Area**	**Height**	**Area%**
12.339	1.11	4192.15	197.00	67.99
13.626	1.34	1973.68	77.70	32.01
	**Sum**	**6165.83**		

(*S*)-1-(1-(phenanthren-9-yl)naphthalen-2-yl)-2-(p-tolyl)diazene [(*S*)-3fe] and (*R*)-1-(phenanthren-9-yl)naphthalen-2-yl trifluoromethanesulfonate [(*R*)-1f]

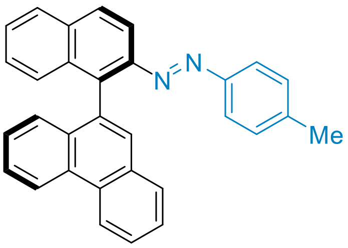

(*S*)-**3fe**

rac-**3fe**

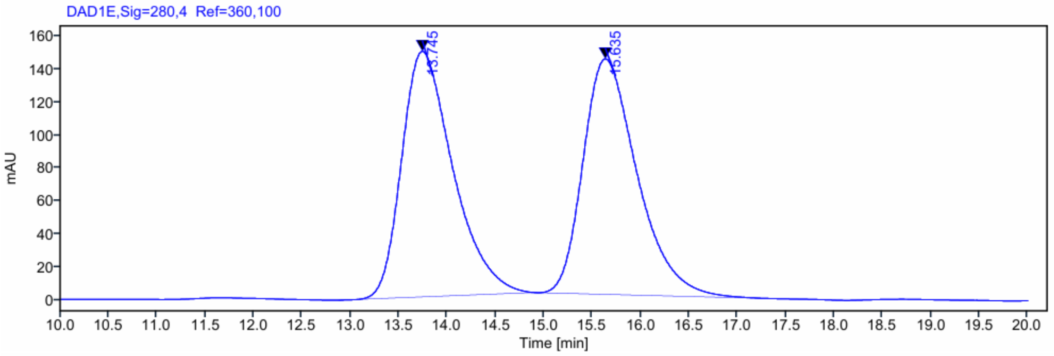

**Signal:**	DAD1E,Sig=280,4 Ref=360,100			
**RT [min]**	**Width [min]**	**Area**	**Height**	**Area%**
13.745	2.01	5483.80	148.94	49.68
15.635	2.62	5554.50	142.82	50.32
	**Sum**	**11038.30**		

(*S*)-**3fe**

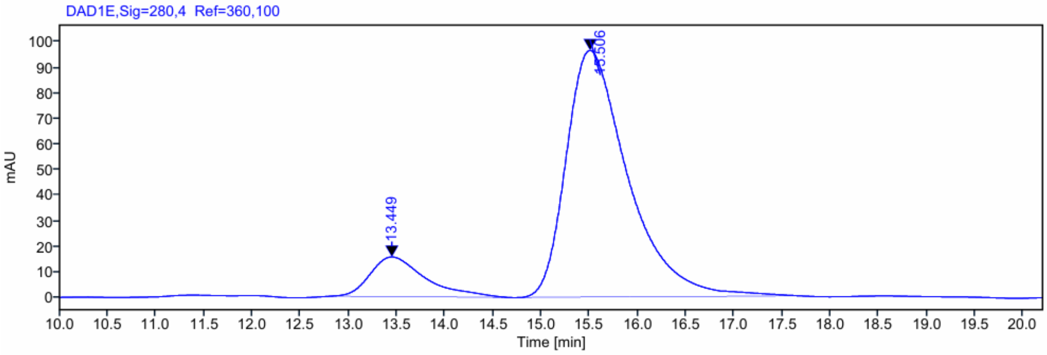

**Signal:**	DAD1E,Sig=280,4 Ref=360,100			
**RT [min]**	**Width [min]**	**Area**	**Height**	**Area%**
13.449	1.85	617.66	15.54	12.55
15.506	3.02	4302.34	96.61	87.45
	**Sum**	**4920.01**		

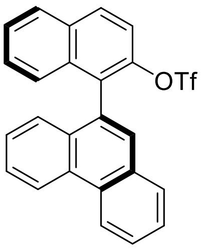

(*R*)-**1f**

rac-**1f**

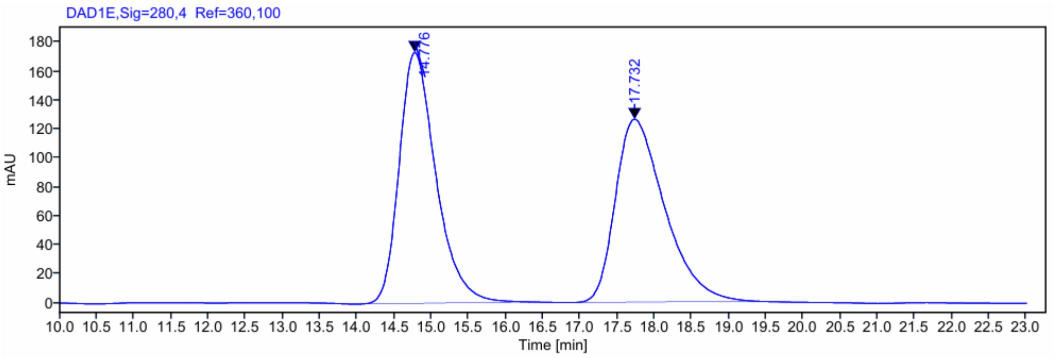

Signal:	DAD1E,Sig=280,4 Ref=360,100			
**RT [min]**	**Width [min]**	**Area**	**Height**	**Area%**
14.776	2.25	5957.01	173.51	50.56
17.732	2.47	5826.04	126.41	49.44
	**Sum**	**11783.05**		

(*R*)-**1f**

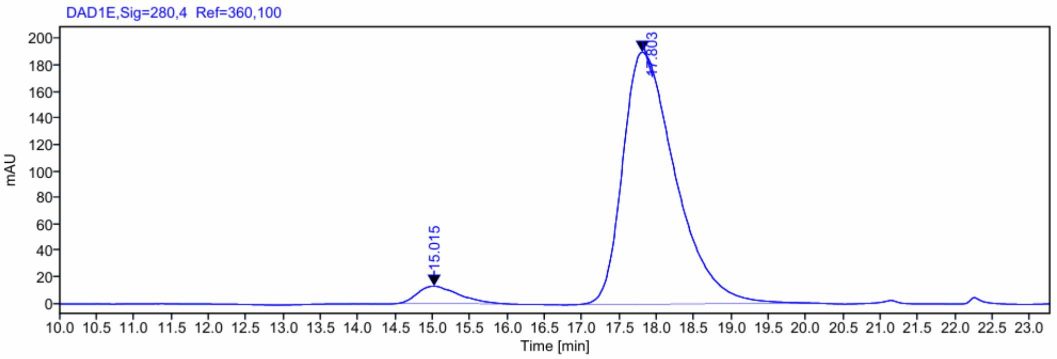

Signal:	DAD1E,Sig=280,4 Ref=360,100			
**RT [min]**	**Width [min]**	**Area**	**Height**	**Area%**
15.015	1.49	509.43	13.28	5.18
17.803	3.23	9324.18	190.55	94.82
	**Sum**	**9833.62**		

(*S*)-1-(1-(2-methoxyphenyl)naphthalen-2-yl)-2-phenyldiazene [(*S*)-3ga] and (*R*)-1-(2-methoxyphenyl)naphthalen-2-yl trifluoromethanesulfonate [(*R*)-1g]

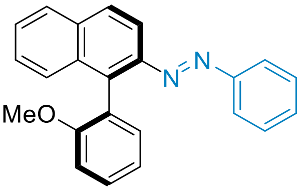

(*S*)-**3ga**

rac-**3ga**

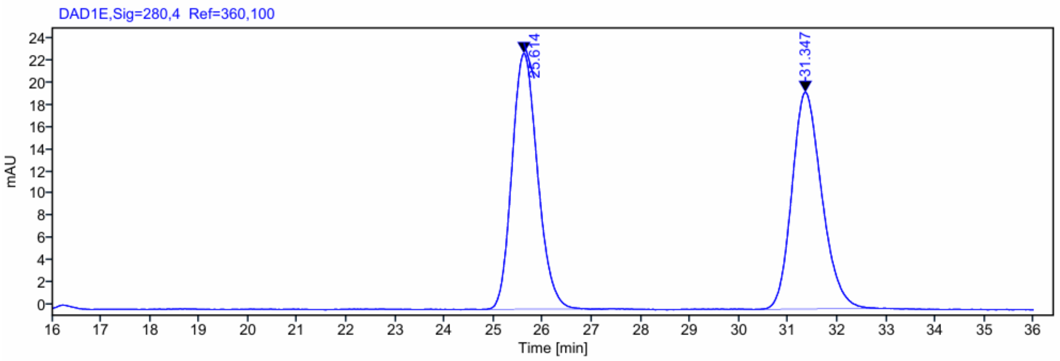

Signal:	DAD1E,Sig=280,4 Ref=360,100			
**RT [min]**	**Width [min]**	**Area**	**Height**	**Area%**
25.614	2.62	834.23	23.00	50.28
31.347	2.71	824.92	19.49	49.72
	**Sum**	**1659.16**		

(*S*)-**3ga**

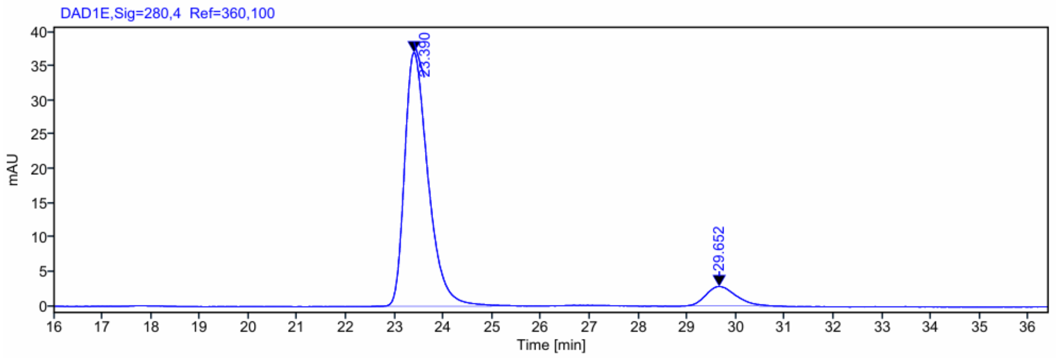

Signal:	DAD1E,Sig=280,4 Ref=360,100			
**RT [min]**	**Width [min]**	**Area**	**Height**	**Area%**
23.390	3.69	1243.01	37.01	91.02
29.652	1.97	122.58	2.83	8.98
	**Sum**	**1365.59**		

(*S*)-**3ga** (after recrystallization)

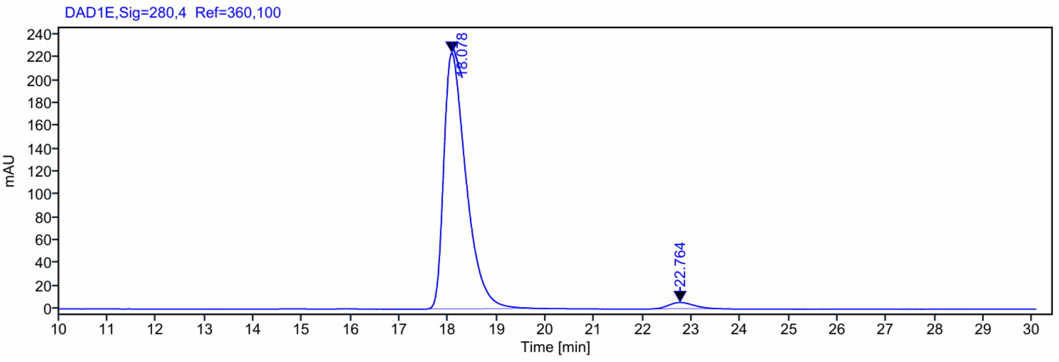

Signal:	DAD1E,Sig=280,4 Ref=360,100			
RT [min]	Width [min]	Area	Height	Area%
18.078	2.28	7112.21	223.90	97.43
22.764	1.25	187.94	5.42	2.57
	Sum	7300.15		

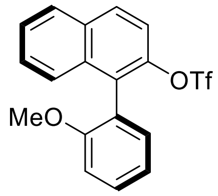

(*R*)-**1g**

rac-**1g**

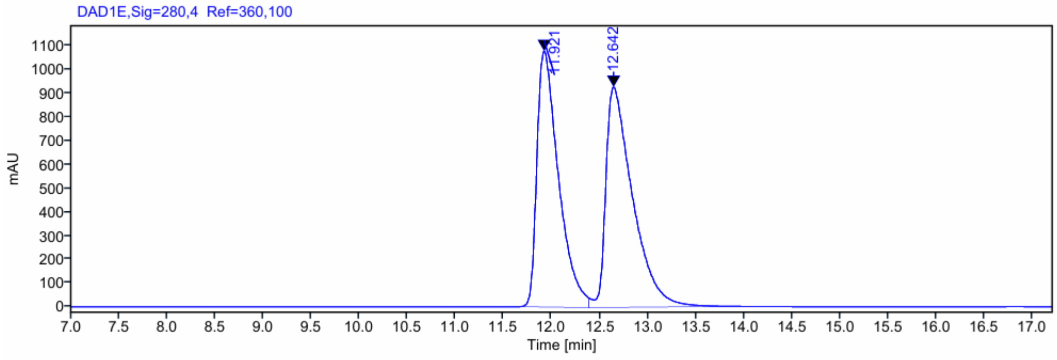

Signal:	DAD1E,Sig=280,4 Ref=360,100			
RT [min]	Width [min]	Area	Height	Area%
11.921	0.73	16863.29	1080.14	48.26
12.642	1.61	18081.85	929.92	51.74
	Sum	34945.13		

(*R*)-**1g**

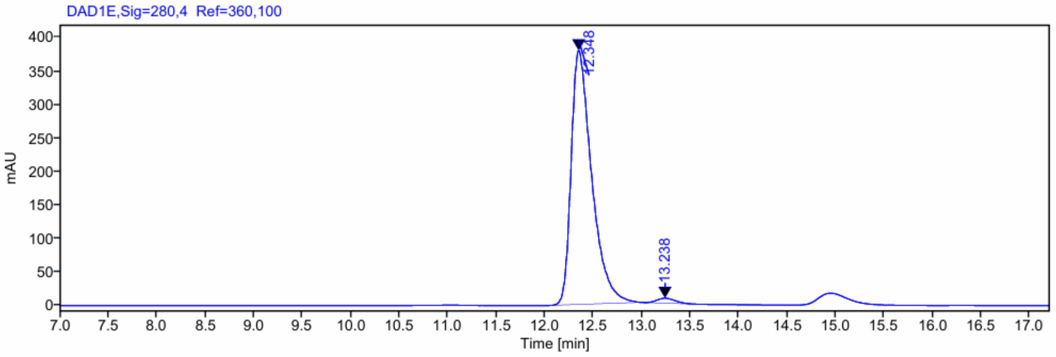

Signal: DAD1E,Sig=280,4 Ref=360,100

RT [min]	Width [min]	Area	Height	Area%
12.348	0.99	5589.24	378.90	98.34
13.238	0.61	94.42	7.34	1.66
	**Sum**	**5683.66**		

(*S*)-1-(4-fluorophenyl)-2-(1-(2-methoxyphenyl)naphthalen-2-yl)diazene [(*S*)-3gb] and (*R*)-1-(2-methoxyphenyl)naphthalen-2-yl trifluoromethanesulfonate [(*R*)-1g]

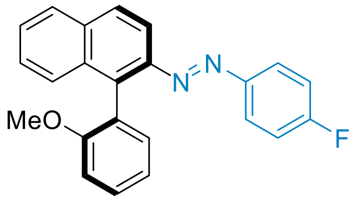

(*S*)-**3gb**

rac-**3gb**

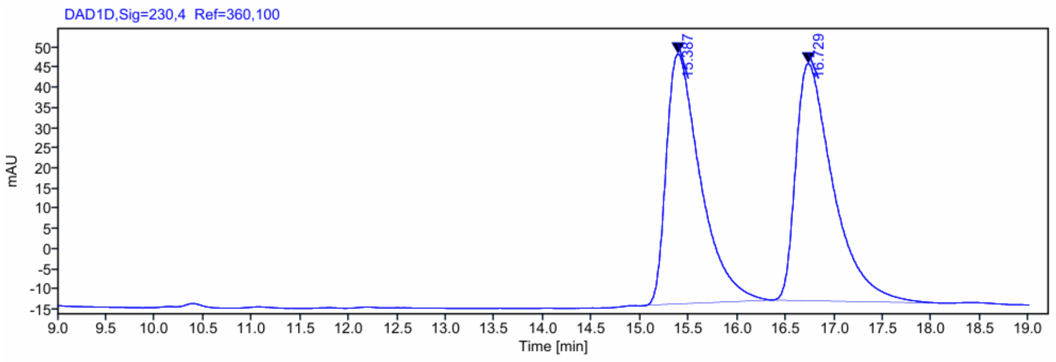

Signal:	DAD1D,Sig=230,4 Ref=360,100			
**RT [min]**	**Width [min]**	**Area**	**Height**	**Area%**
15.387	1.43	1512.45	62.05	48.99
16.729	1.79	1574.87	58.81	51.01
	**Sum**	**3087.32**		

(*S*)-**3gb**

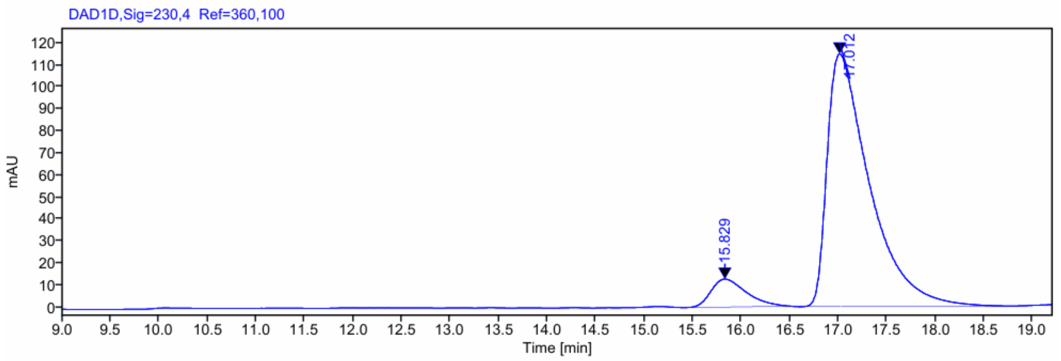

Signal:	DAD1D,Sig=230,4 Ref=360,100			
**RT [min]**	**Width [min]**	**Area**	**Height**	**Area%**
15.829	1.13	319.50	12.70	8.46
17.012	2.20	3458.67	114.32	91.54
	**Sum**	**3778.17**		

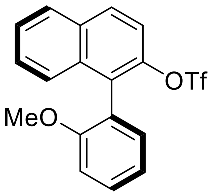

(*R*)-**1g**

rac-**1g**

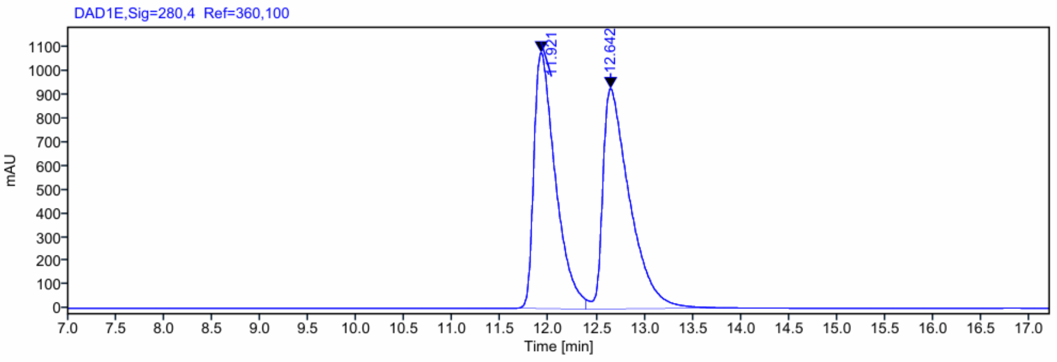

**Signal:**	DAD1E,Sig=280,4 Ref=360,100			
**RT [min]**	**Width [min]**	**Area**	**Height**	**Area%**
11.921	0.73	16863.29	1080.14	48.26
12.642	1.61	18081.85	929.92	51.74
	**Sum**	**34945.13**		

(*R*)-**1g**

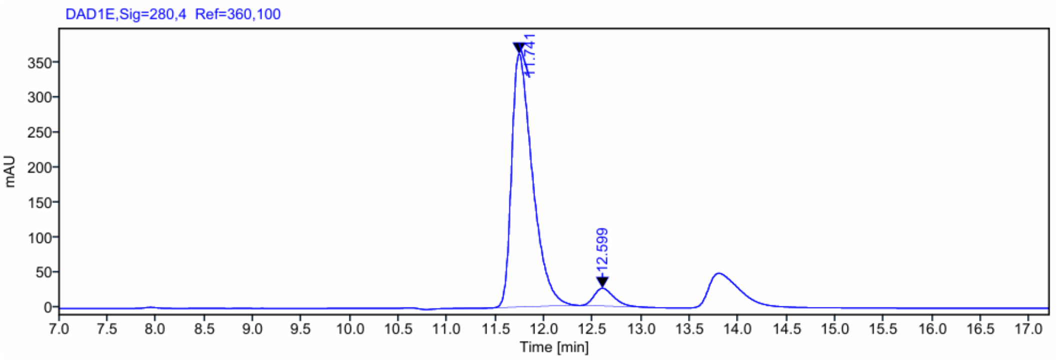

**Signal:**	DAD1E,Sig=280,4 Ref=360,100			
**RT [min]**	**Width [min]**	**Area**	**Height**	**Area%**
11.741	0.95	5401.03	360.98	93.84
12.599	0.71	354.54	25.23	6.16
	**Sum**	**5755.57**		

(*S*)-1-(1-(2-ethylphenyl)naphthalen-2-yl)-2-phenyldiazene [(*S*)-3ha] and (*R*)-1-(2-ethylphenyl)naphthalen-2-yl trifluoromethanesulfonate [(*R*)-1h]

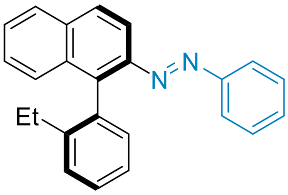

(*S*)-**3ha**

rac-**3ha**

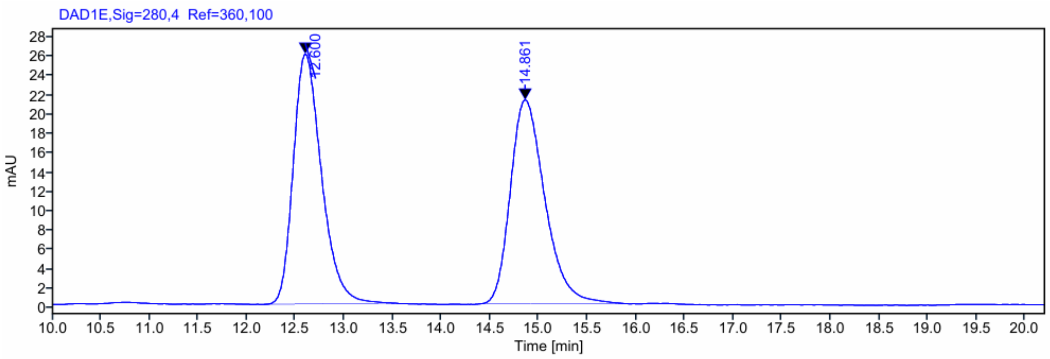

Signal:	DAD1E,Sig=280,4 Ref=360,100			
**RT [min]**	**Width [min]**	**Area**	**Height**	**Area%**
12.600	1.33	521.51	25.85	50.06
14.861	1.48	520.24	21.08	49.94
	**Sum**	**1041.75**		

(*S*)-**3ha**

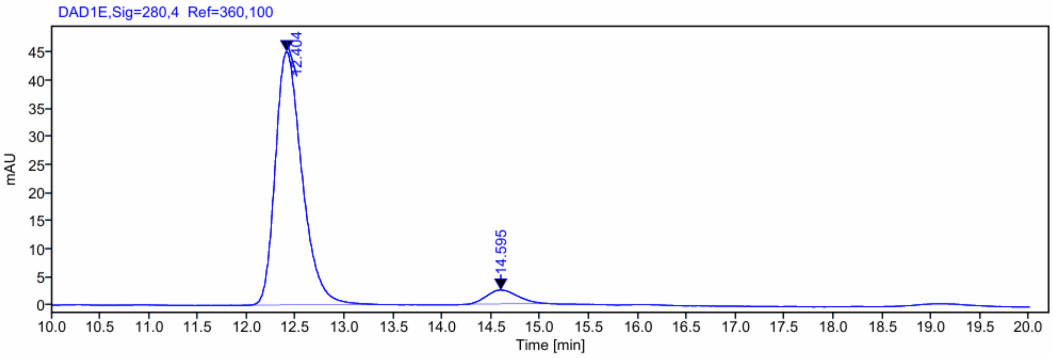

Signal:	DAD1E,Sig=280,4 Ref=360,100			
**RT [min]**	**Width [min]**	**Area**	**Height**	**Area%**
12.404	1.39	854.84	45.07	93.96
14.595	0.83	54.99	2.47	6.04
	**Sum**	**909.83**		

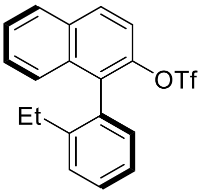

(*R*)-**1h**

rac-**1h**

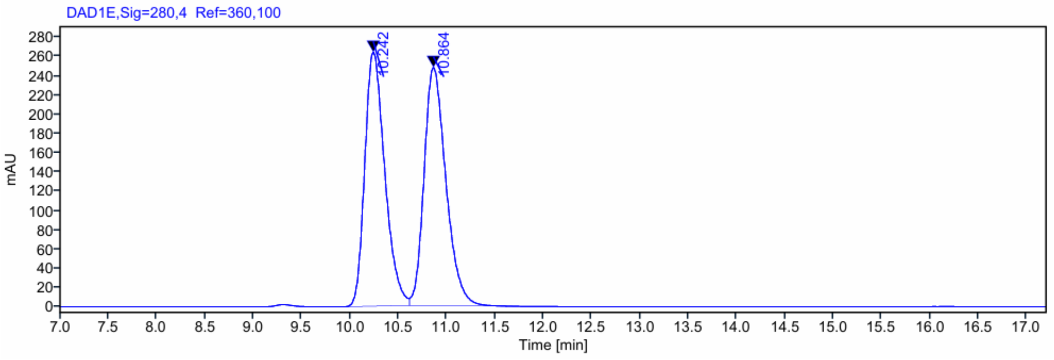

**Signal:**	DAD1E,Sig=280,4 Ref=360,100			
**RT [min]**	**Width [min]**	**Area**	**Height**	**Area%**
10.242	0.75	3800.71	263.72	49.43
10.864	1.01	3889.03	247.31	50.57
	**Sum**	**7689.74**		

(*R*)-**1h**

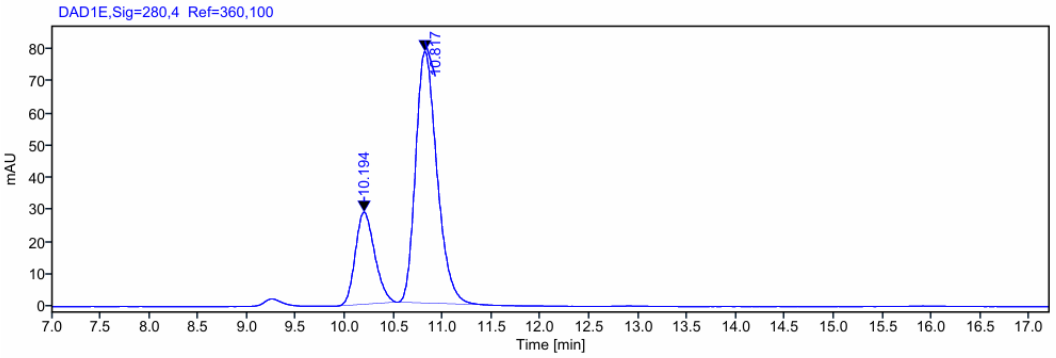

**Signal:**	DAD1E,Sig=280,4 Ref=360,100			
**RT [min]**	**Width [min]**	**Area**	**Height**	**Area%**
10.194	0.59	393.42	28.66	24.87
10.817	0.89	1188.19	78.22	75.13
	**Sum**	**1581.60**		

(*S*)-1-phenyl-2-(1-(o-tolyl)naphthalen-2-yl)diazene [(*S*)-3ia] and (*R*)-1-(o-tolyl)naphthalen-2-yl trifluoromethanesulfonate [(*R*)-1i]

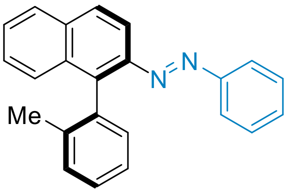

(*S*)-**3ia**

rac-**3ia**

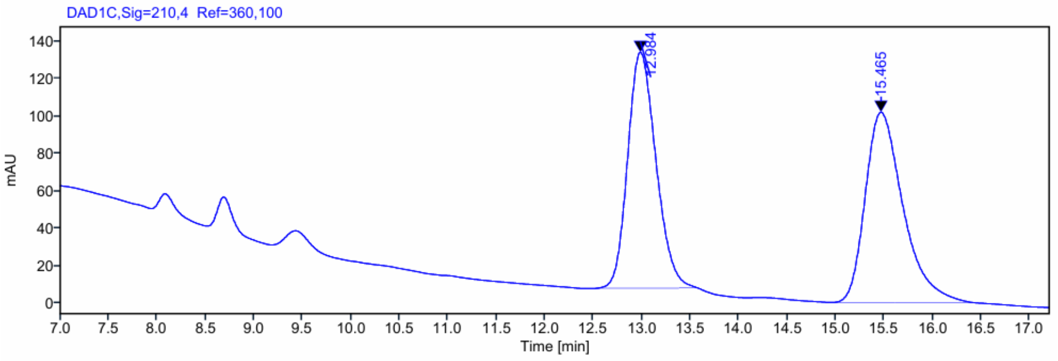

Signal:	DAD1C,Sig=210,4 Ref=360,100			
**RT [min]**	**Width [min]**	**Area**	**Height**	**Area%**
12.984	1.06	2559.01	126.07	48.11
15.465	1.53	2760.44	101.71	51.89
	**Sum**	**5319.45**		

(*S*)-**3ia**

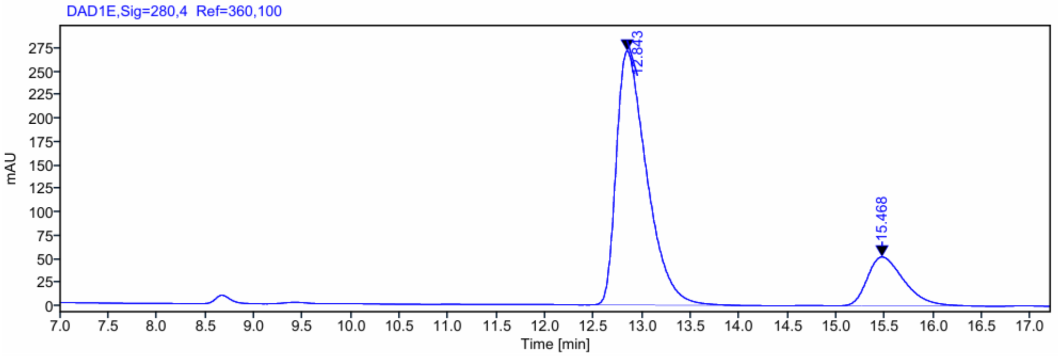

Signal:	DAD1E,Sig=280,4 Ref=360,100			
**RT [min]**	**Width [min]**	**Area**	**Height**	**Area%**
12.843	1.62	5910.72	270.75	81.35
15.468	1.34	1355.22	52.05	18.65
	**Sum**	**7265.94**		

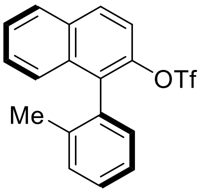

(*R*)-**1i**

rac-**1i**

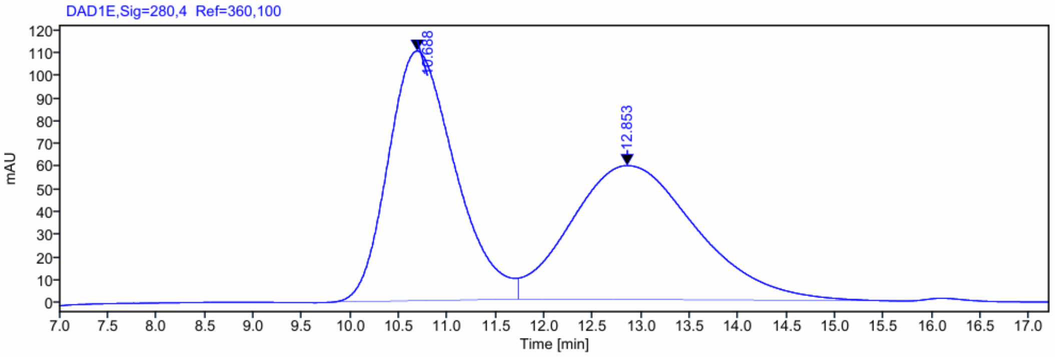

Signal:	DAD1E,Sig=280,4 Ref=360,100			
**RT [min]**	**Width [min]**	**Area**	**Height**	**Area%**
10.688	1.90	5286.15	110.13	49.86
12.853	3.69	5316.18	59.09	50.14
	**Sum**	**10602.33**		

(*R*)-**1i**

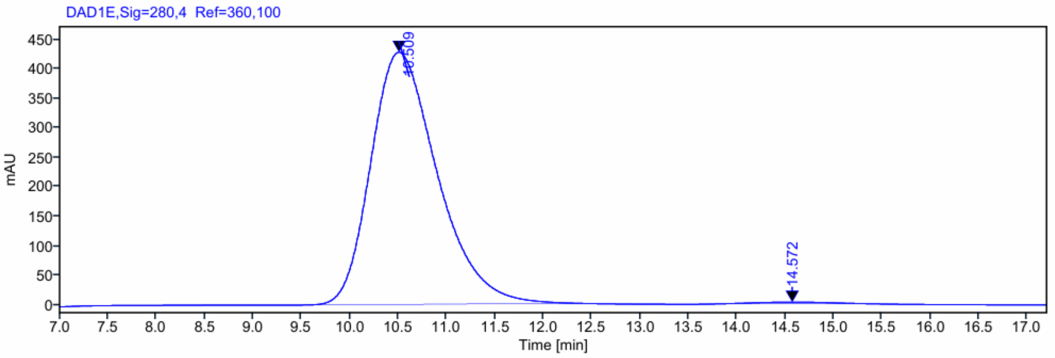

Signal:	DAD1E,Sig=280,4 Ref=360,100			
**RT [min]**	**Width [min]**	**Area**	**Height**	**Area%**
10.509	2.78	20404.69	427.26	99.37
14.572	1.41	129.91	2.56	0.63
	**Sum**	**20534.59**		

(*S*)-1-(1-(4-fluoro-2-methylphenyl)naphthalen-2-yl)-2-phenyldiazene [(*S*)-3ja] and (*R*)-1-(4-fluoro-2-methylphenyl)naphthalen-2-yl trifluoromethanesulfonate [(*R*)-1j]

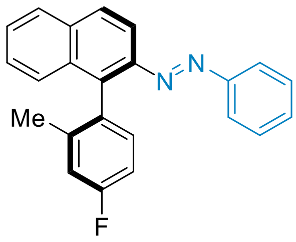

(*S*)-**3ja**

rac-**3ja**

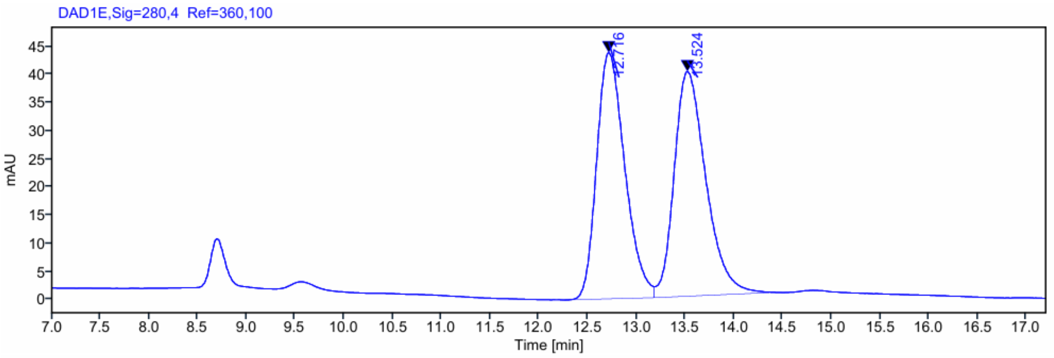

Signal:	DAD1E,Sig=280,4 Ref=360,100			
**RT [min]**	**Width [min]**	**Area**	**Height**	**Area%**
12.716	0.92	880.56	43.73	49.36
13.524	1.24	903.35	39.90	50.64
	**Sum**	**1783.92**		

(*S*)-**3ja**

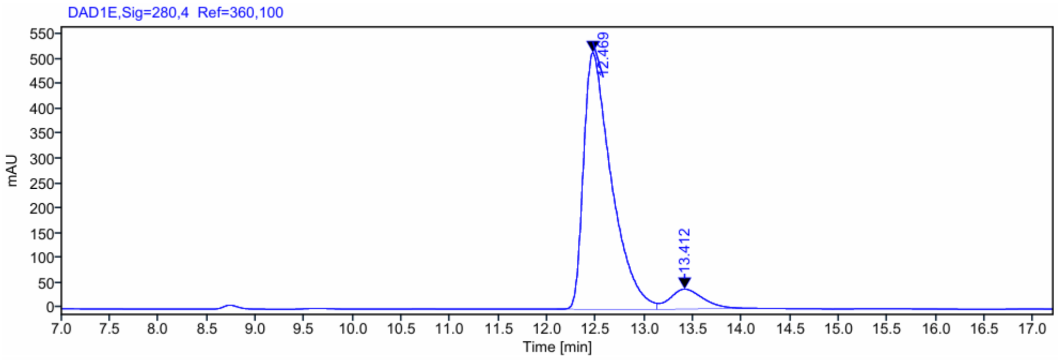

Signal:	DAD1E,Sig=280,4 Ref=360,100			
**RT [min]**	**Width [min]**	**Area**	**Height**	**Area%**
12.469	1.03	10315.93	516.37	91.51
13.412	0.96	956.93	40.06	8.49
	**Sum**	**11272.86**		

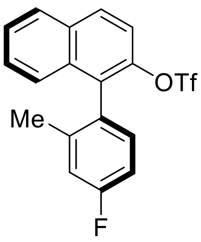

(*R*)-**1j**

rac-**1j**

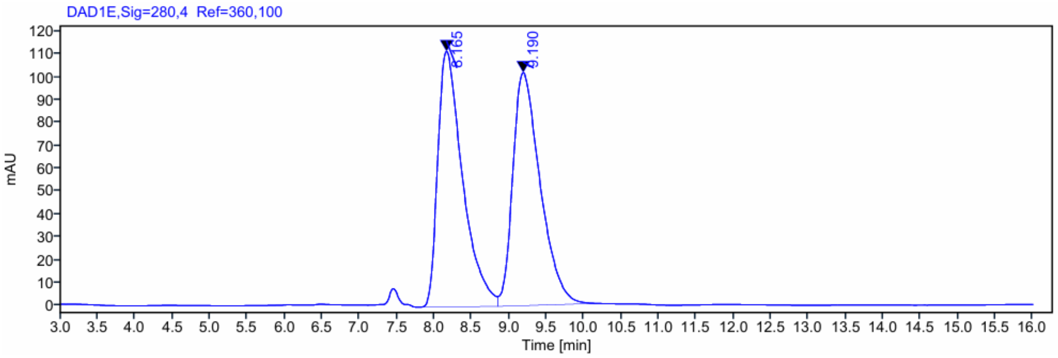

Signal: DAD1E,Sig=280,4 Ref=360,100

RT [min]	Width [min]	Area	Height	Area%
8.165	1.00	2577.71	111.73	49.78
9.190	1.33	2600.68	101.98	50.22
	**Sum**	**5178.39**		

(*R*)-**1j**

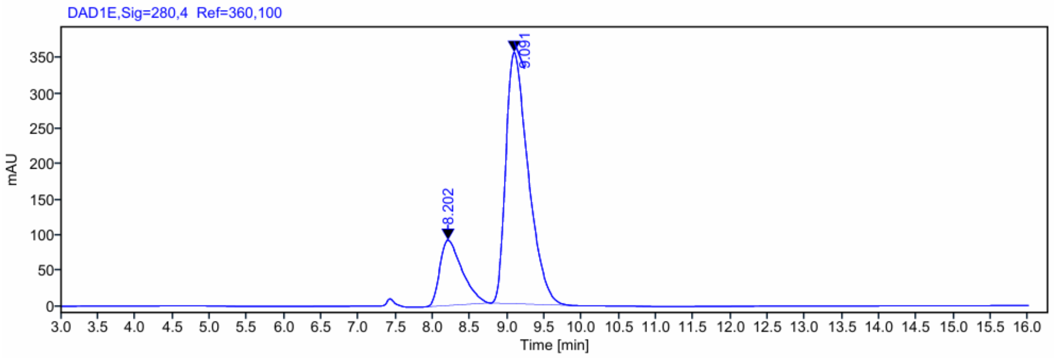

Signal: DAD1E,Sig=280,4 Ref=360,100

RT [min]	Width [min]	Area	Height	Area%
8.202	0.88	1913.53	92.22	20.45
9.091	1.23	7441.35	355.06	79.55
	**Sum**	**9354.88**		

(*S*)-1-(1-(2-chlorophenyl)naphthalen-2-yl)-2-phenyldiazene [(*S*)-3ka] and (*R*)-1-(2-chlorophenyl)naphthalen-2-yl trifluoromethanesulfonate [(*R*)-1k]

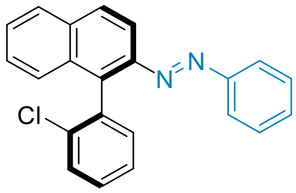

(*S*)-**3ka**

rac-**3ka**

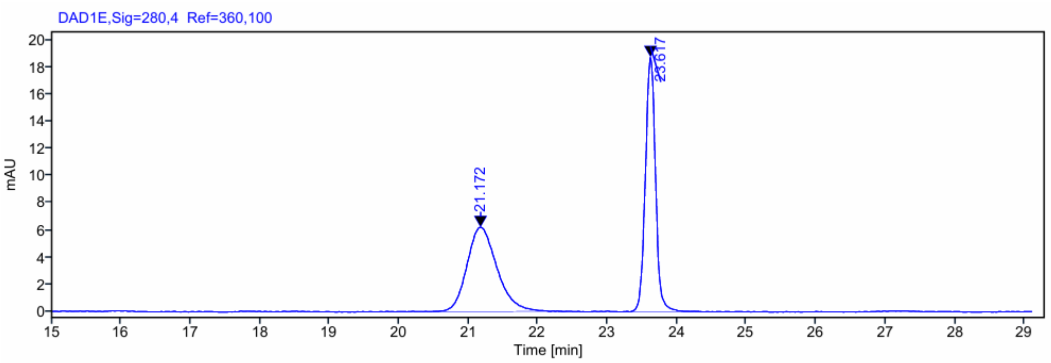

**Signal:**	DAD1E,Sig=280,4 Ref=360,100			
**RT [min]**	**Width [min]**	**Area**	**Height**	**Area%**
21.172	1.99	186.20	6.21	49.25
23.617	1.33	191.86	18.76	50.75
	**Sum**	**378.06**		

(*S*)-**3ka**

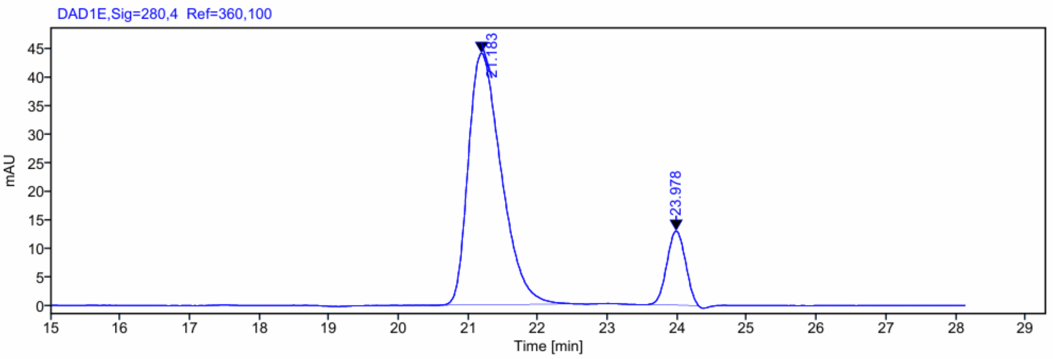

**Signal:**	DAD1E,Sig=280,4 Ref=360,100			
**RT [min]**	**Width [min]**	**Area**	**Height**	**Area%**
21.183	2.07	1444.65	44.01	85.76
23.978	0.71	239.78	12.95	14.24
	**Sum**	**1684.44**		

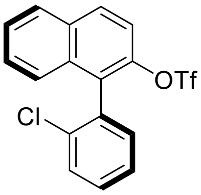

(*R*)-**1k**

rac-**1k**

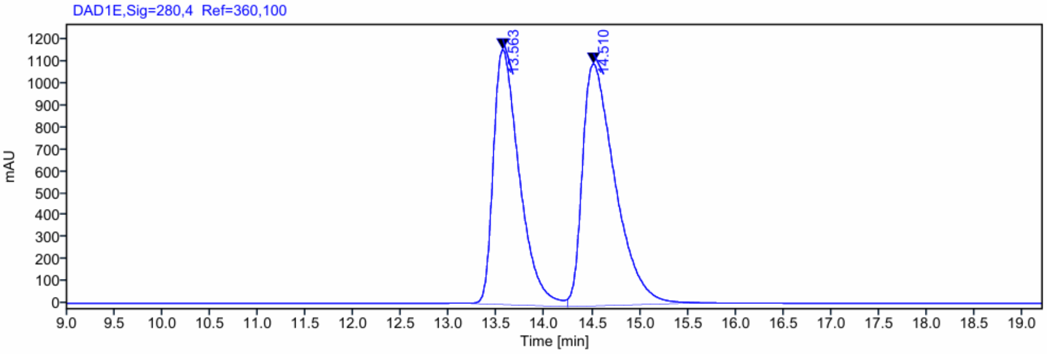

**Signal:**	DAD1E,Sig=280,4 Ref=360,100			
**RT [min]**	**Width [min]**	**Area**	**Height**	**Area%**
13.563	1.15	21943.74	1156.91	45.70
14.510	1.42	26072.18	1098.83	54.30
	**Sum**	**48015.93**		

(*R*)-**1k**

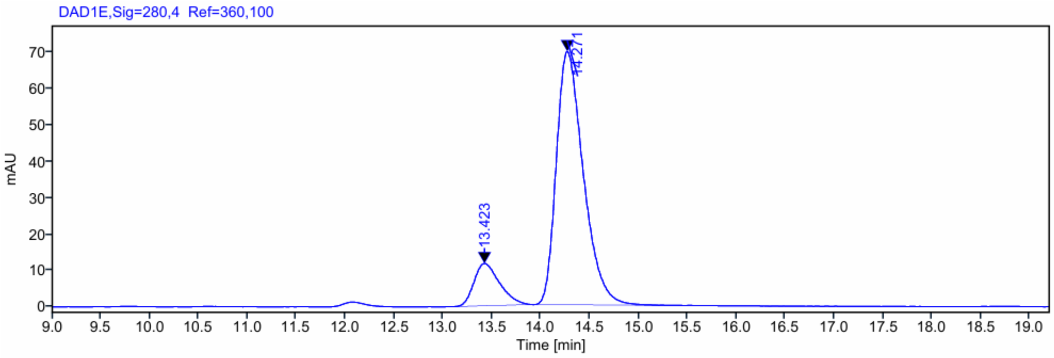

**Signal:**	DAD1E,Sig=280,4 Ref=360,100			
**RT [min]**	**Width [min]**	**Area**	**Height**	**Area%**
13.423	0.79	212.34	11.65	13.66
14.271	1.56	1342.44	69.74	86.34
	**Sum**	**1554.78**		

(*S*)-1-(1-([1,1'-biphenyl]-2-yl)naphthalen-2-yl)-2-phenyldiazene [(*S*)-3la] and (*R*)-1-([1,1'-biphenyl]-2-yl)naphthalen-2-yl trifluoromethanesulfonate [(*R*)-1l]

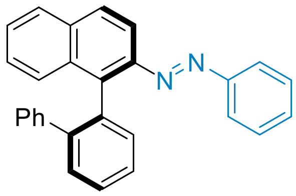

(*S*)-**3la**

rac-**3la**

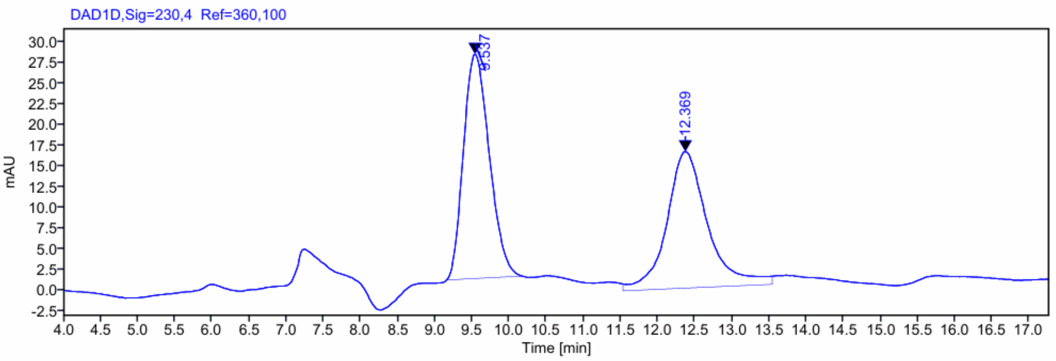

**Signal:**	DAD1D,Sig=230,4 Ref=360,100			
**RT [min]**	**Width [min]**	**Area**	**Height**	**Area%**
9.537	1.01	654.35	27.00	50.99
12.369	2.01	628.83	16.42	49.01
	**Sum**	**1283.18**		

(*S*)-**3la**

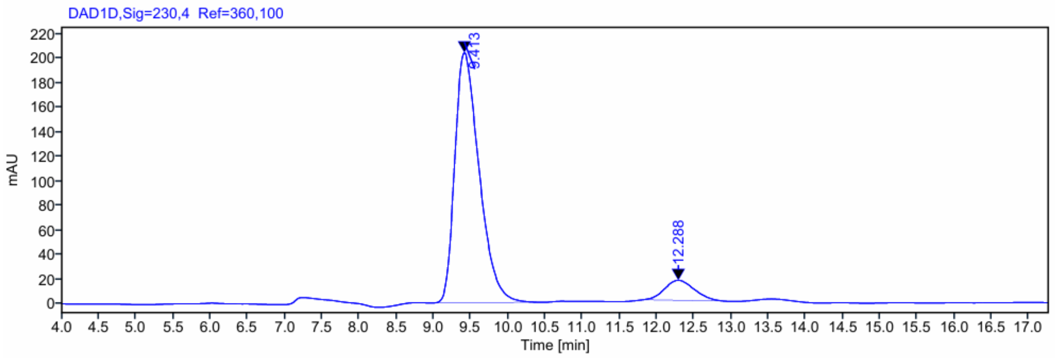

**Signal:**	DAD1D,Sig=230,4 Ref=360,100			
**RT [min]**	**Width [min]**	**Area**	**Height**	**Area%**
9.413	1.45	4638.75	203.53	91.15
12.288	1.06	450.60	16.26	8.85
	**Sum**	**5089.35**		

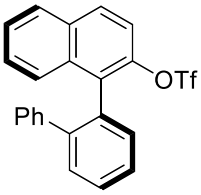

(*R*)-**1l**

rac-**1l**

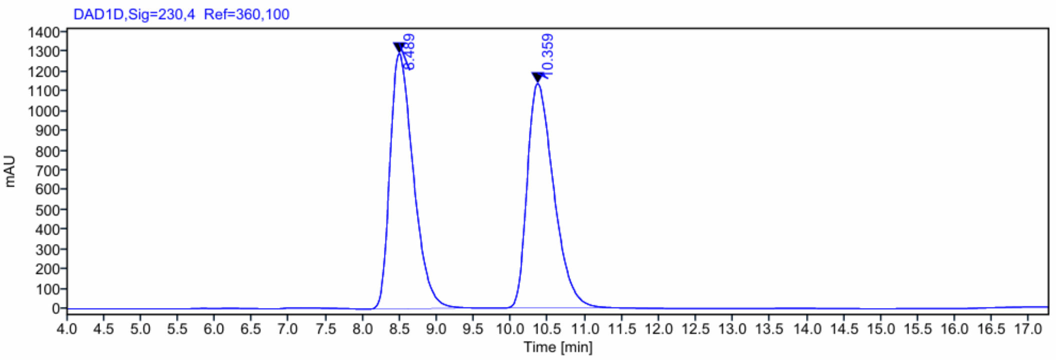

**Signal:**	DAD1D,Sig=230,4 Ref=360,100			
**RT [min]**	**Width [min]**	**Area**	**Height**	**Area%**
8.489	1.48	28385.29	1291.10	50.07
10.359	1.61	28308.95	1135.81	49.93
	**Sum**	**56694.24**		

(*R*)-**1l**

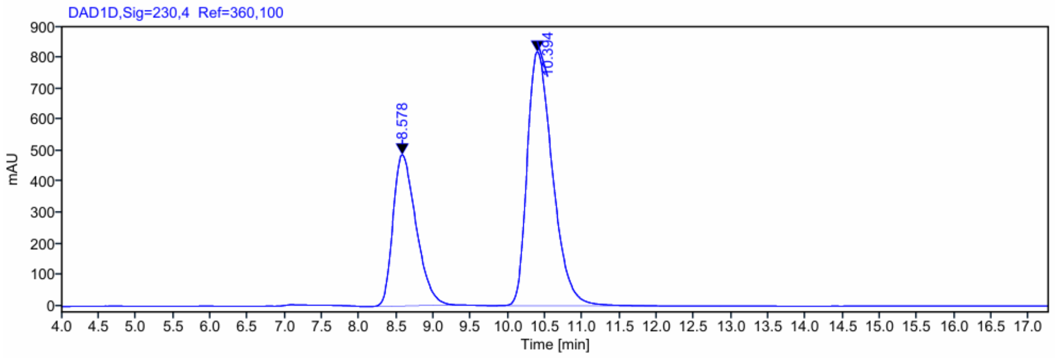

**Signal:**	DAD1D,Sig=230,4 Ref=360,100			
**RT [min]**	**Width [min]**	**Area**	**Height**	**Area%**
8.578	1.22	10535.07	487.89	35.20
10.394	1.82	19392.63	818.78	64.80
	**Sum**	**29927.70**		

***cis*-(*S*)-1-([1,1'-binaphthalen]-2-yl)-2-phenyldiazene [*cis*-(*S*)-3aa]**

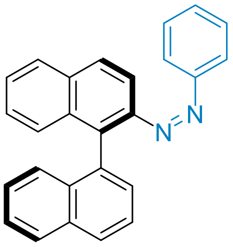

cis-(*S*)-**3aa,** 89% ee

cis-*rac*-**3aa**

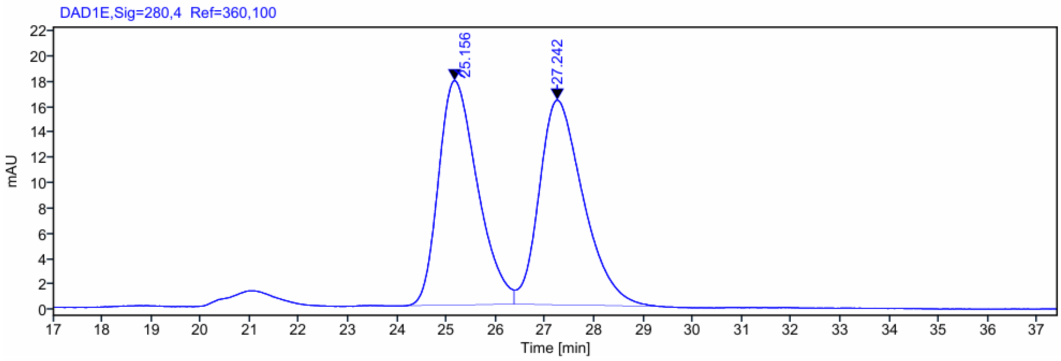

**Signal:**	DAD1E,Sig=280,4 Ref=360,100			
**RT [min]**	**Width [min]**	**Area**	**Height**	**Area%**
25.156	2.41	974.83	17.73	49.13
27.242	2.97	1009.55	16.18	50.87
	**Sum**	**1984.38**		

cis-(*S*)-**3aa**

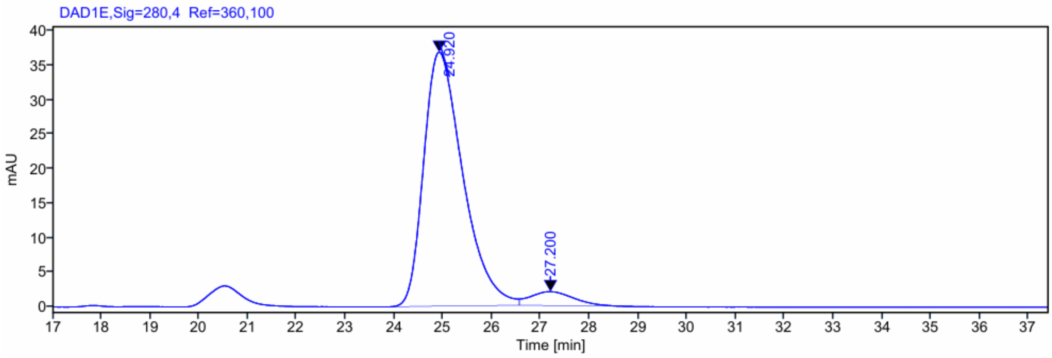

**Signal:**	DAD1E,Sig=280,4 Ref=360,100			
**RT [min]**	**Width [min]**	**Area**	**Height**	**Area%**
24.920	2.71	2058.10	36.91	94.25
27.200	2.32	125.62	2.03	5.75
	**Sum**	**2183.72**		

9 NMR Spectra

^{1}H NMR spectrum (500 MHz, $CDCl_3$, 298 K) of [1,1'-binaphthalen]-2-yl trifluoromethanesulfonate (*rac*-**1a**)

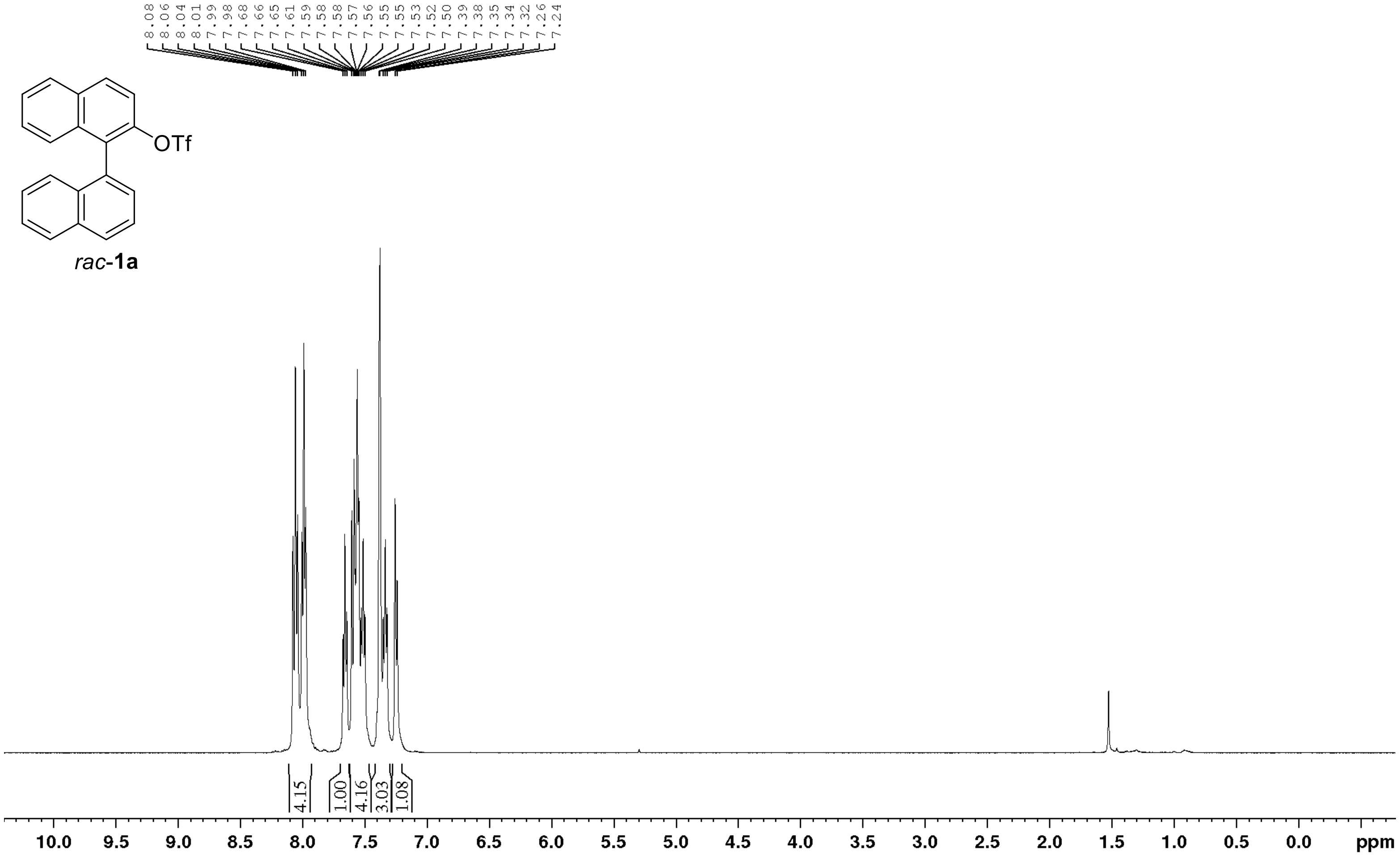

$^{13}C\{^1H\}$ NMR spectrum (126 MHz, $CDCl_3$, 298 K) of [1,1'-binaphthalen]-2-yl trifluoromethanesulfonate (*rac*-**1a**)

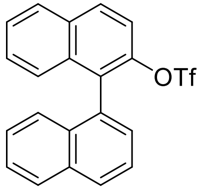

rac-**1a**

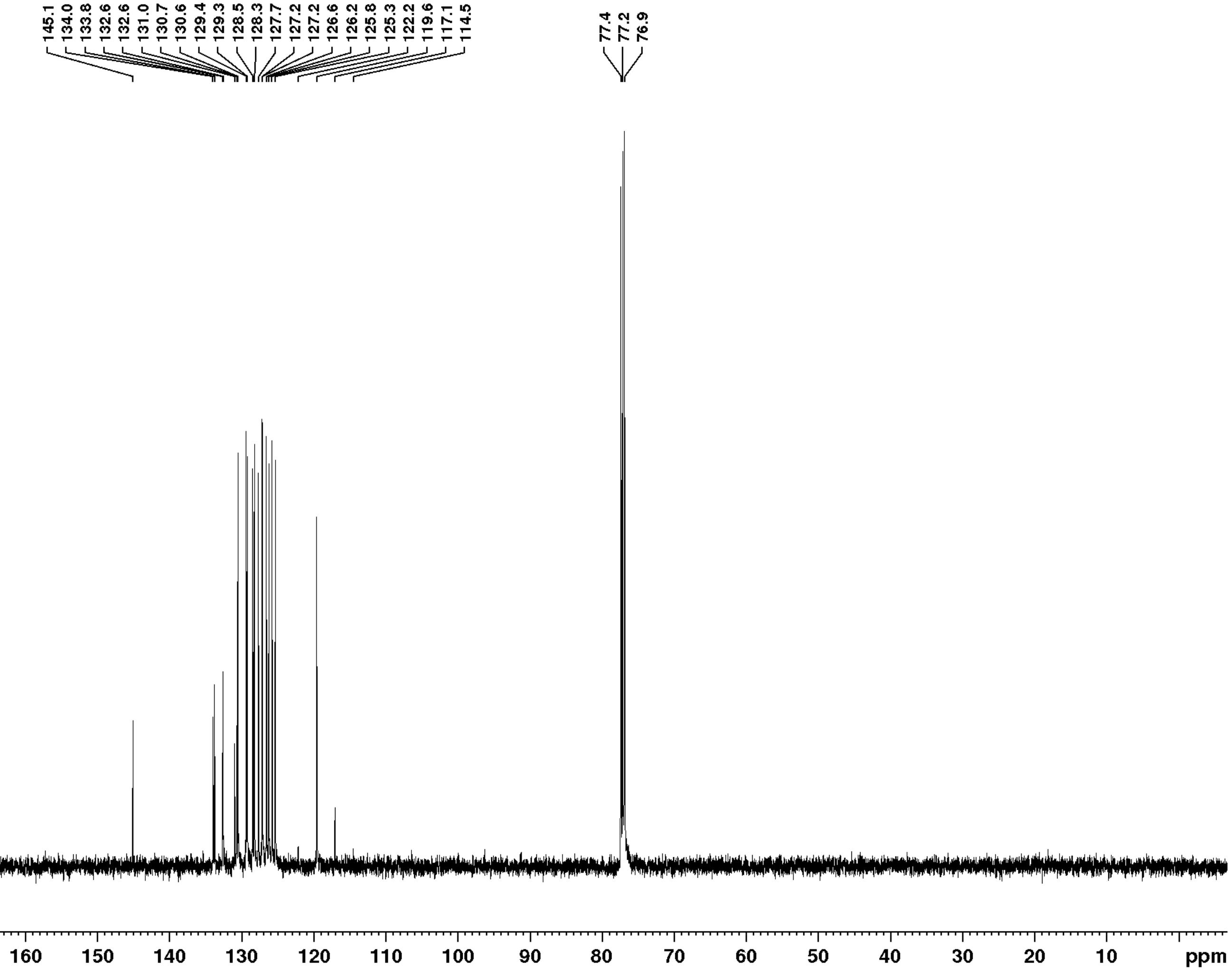

^{19}F NMR spectrum (471 MHz, $CDCl_3$, 298 K) of [1,1'-binaphthalen]-2-yl trifluoromethanesulfonate (*rac*-**1a**)

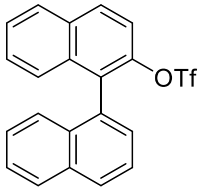

rac-**1a**

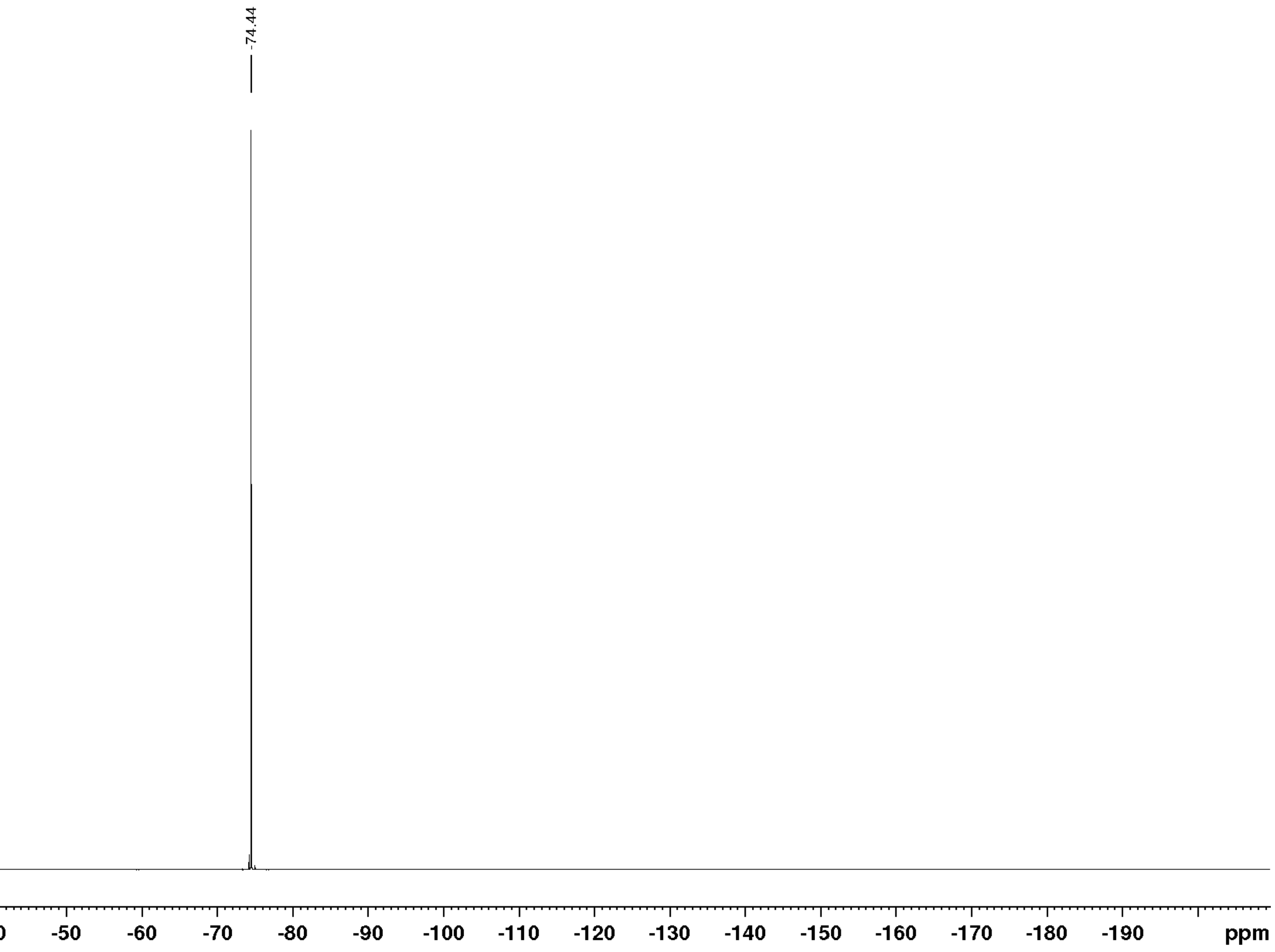

^{1}H NMR spectrum (500 MHz, $CDCl_3$, 298 K) of 6-methoxy-[1,1'-binaphthalen]-2-yl trifluoromethanesulfonate (*rac*-**1b**)

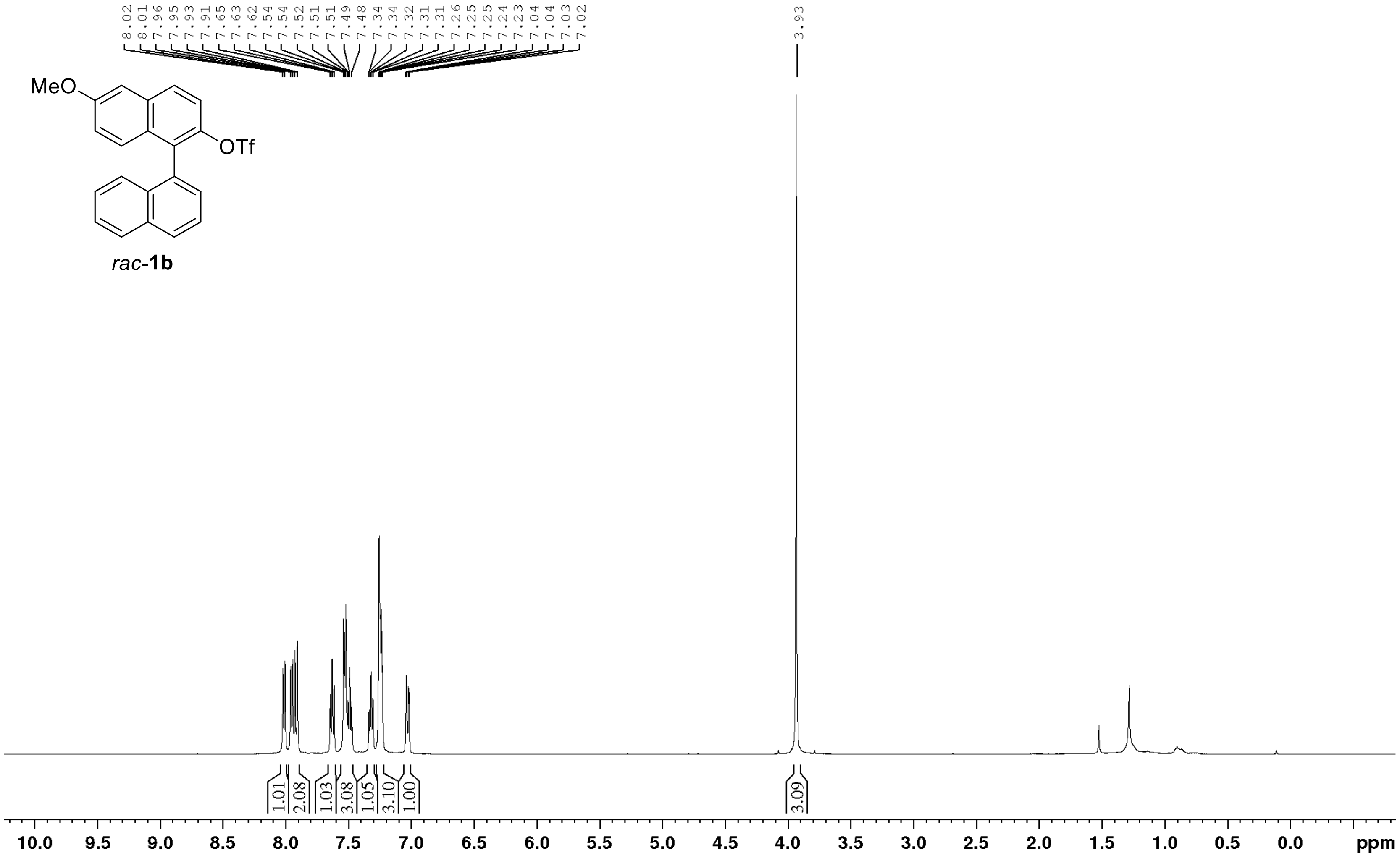

$^{13}C\{^{1}H\}$ NMR spectrum (126 MHz, $CDCl_3$, 298 K) of 6-methoxy-[1,1'-binaphthalen]-2-yl trifluoromethanesulfonate (*rac*-**1b**)

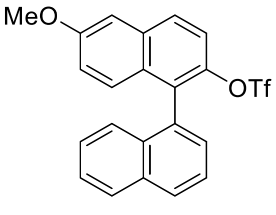

rac-**1b**

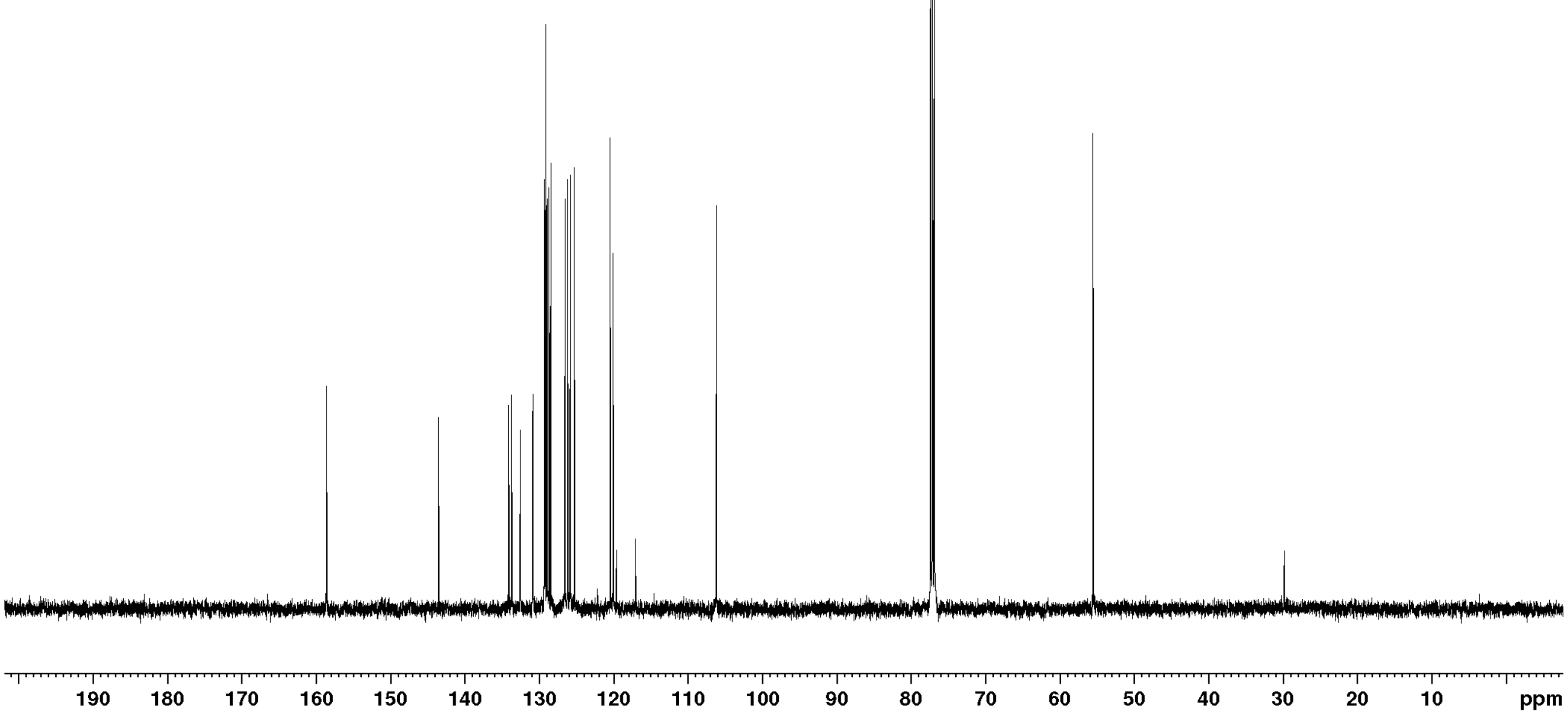

^{19}F NMR spectrum (471 MHz, $CDCl_3$, 298 K) of 6-methoxy-[1,1'-binaphthalen]-2-yl trifluoromethanesulfonate (*rac*-**1b**)

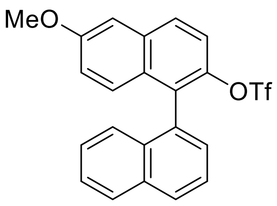

rac-**1b**

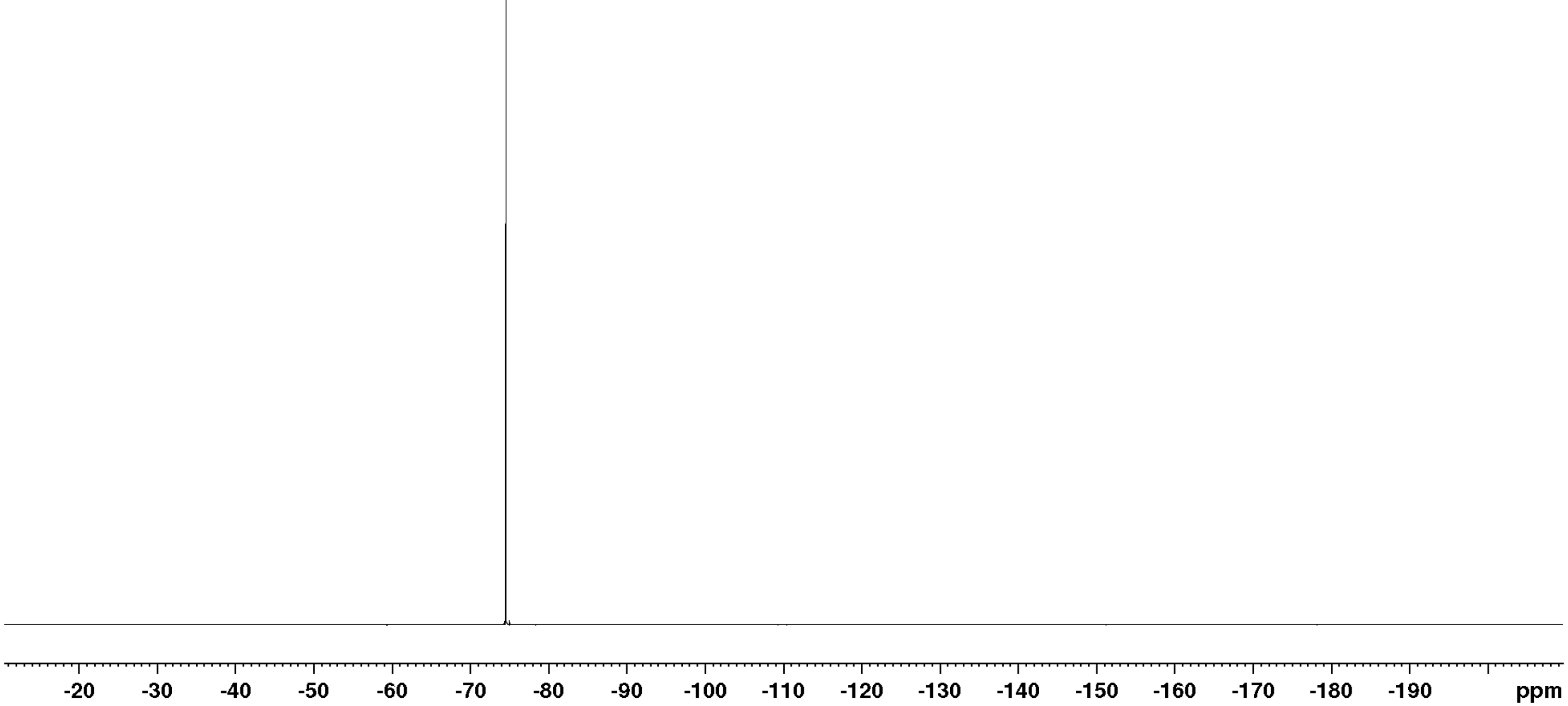

^{1}H NMR spectrum (400 MHz, $CDCl_3$, 298 K) of 6-(trimethylsilyl)-[1,1'-binaphthalen]-2-yl trifluoromethanesulfonate (*rac*-**1c**)

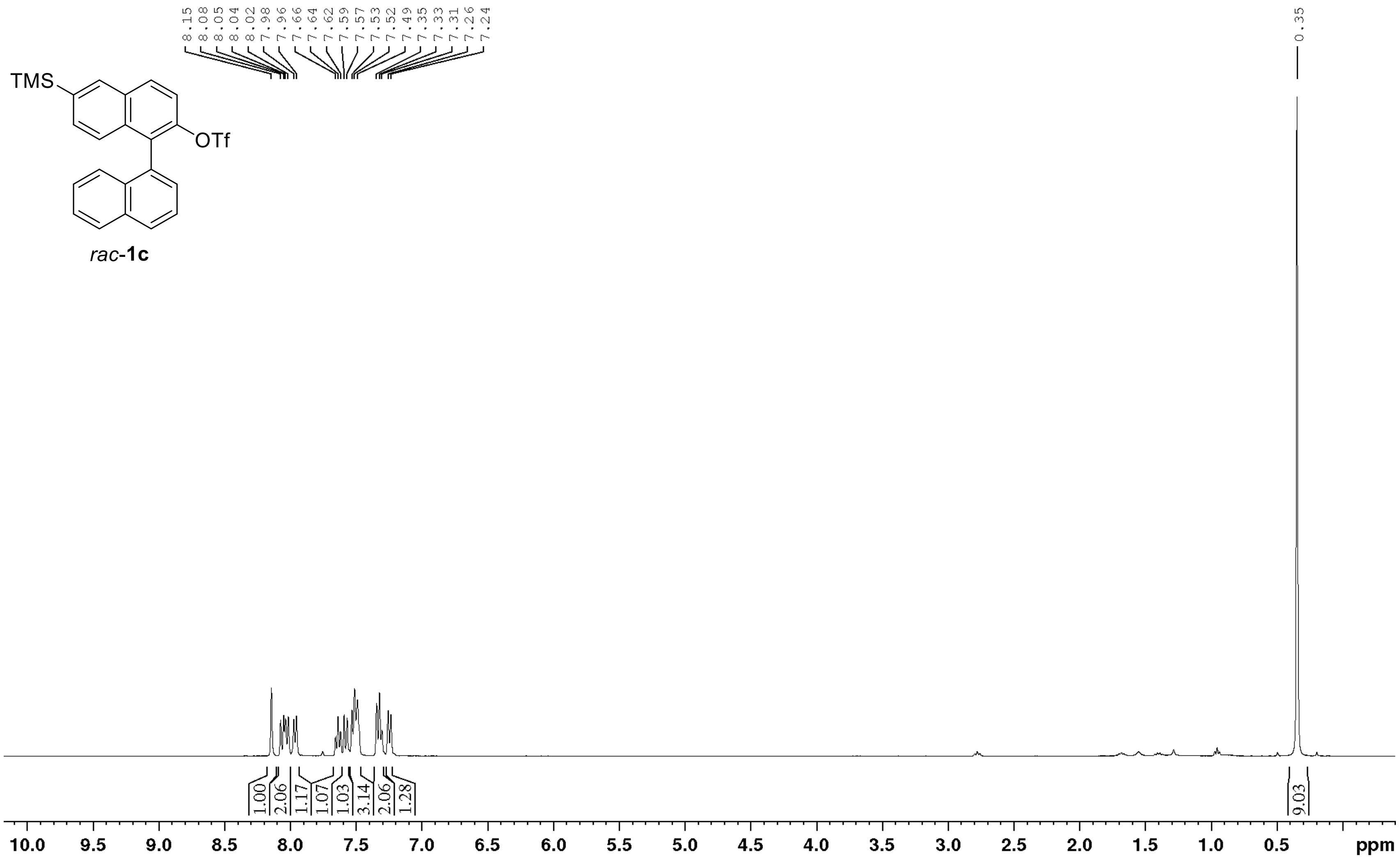

$^{13}C\{^{1}H\}$ NMR spectrum (101 MHz, $CDCl_3$, 298 K) of 6-(trimethylsilyl)-[1,1'-binaphthalen]-2-yl trifluoromethanesulfonate (*rac*-**1c**)

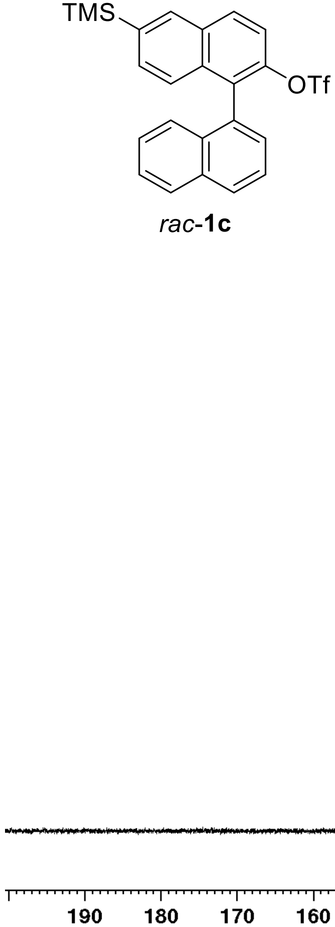

rac-**1c**

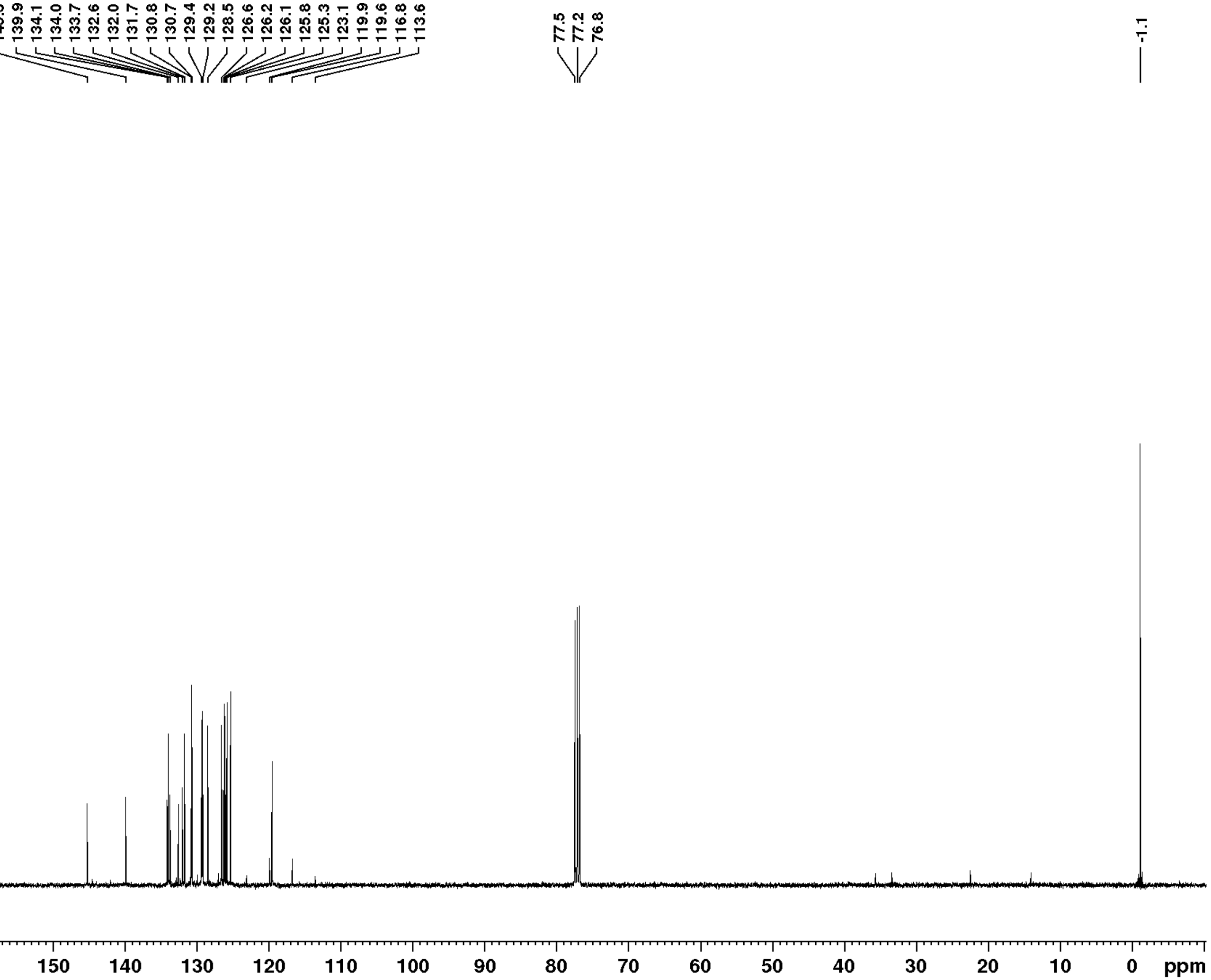

^{19}F NMR spectrum (471 MHz, $CDCl_3$, 298 K) of 6-(trimethylsilyl)-[1,1'-binaphthalen]-2-yl trifluoromethanesulfonate (*rac*-**1c**)

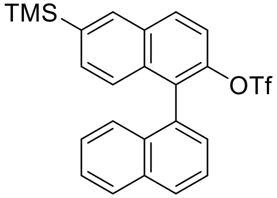

rac-**1c**

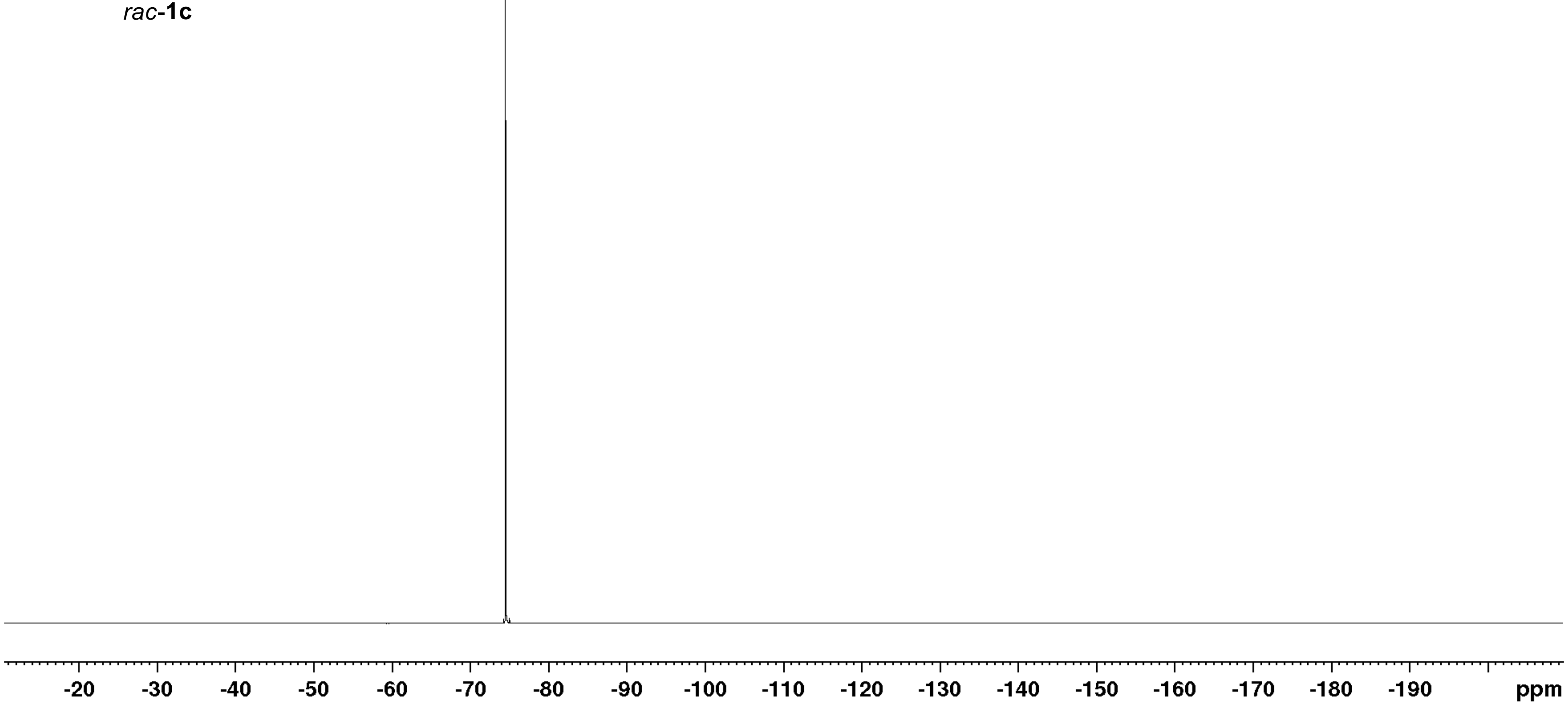

^{1}H NMR spectrum (500 MHz, $CDCl_3$, 298 K) of 3-methyl-[1,1'-binaphthalen]-2-yl trifluoromethanesulfonate (*rac*-**1d**)

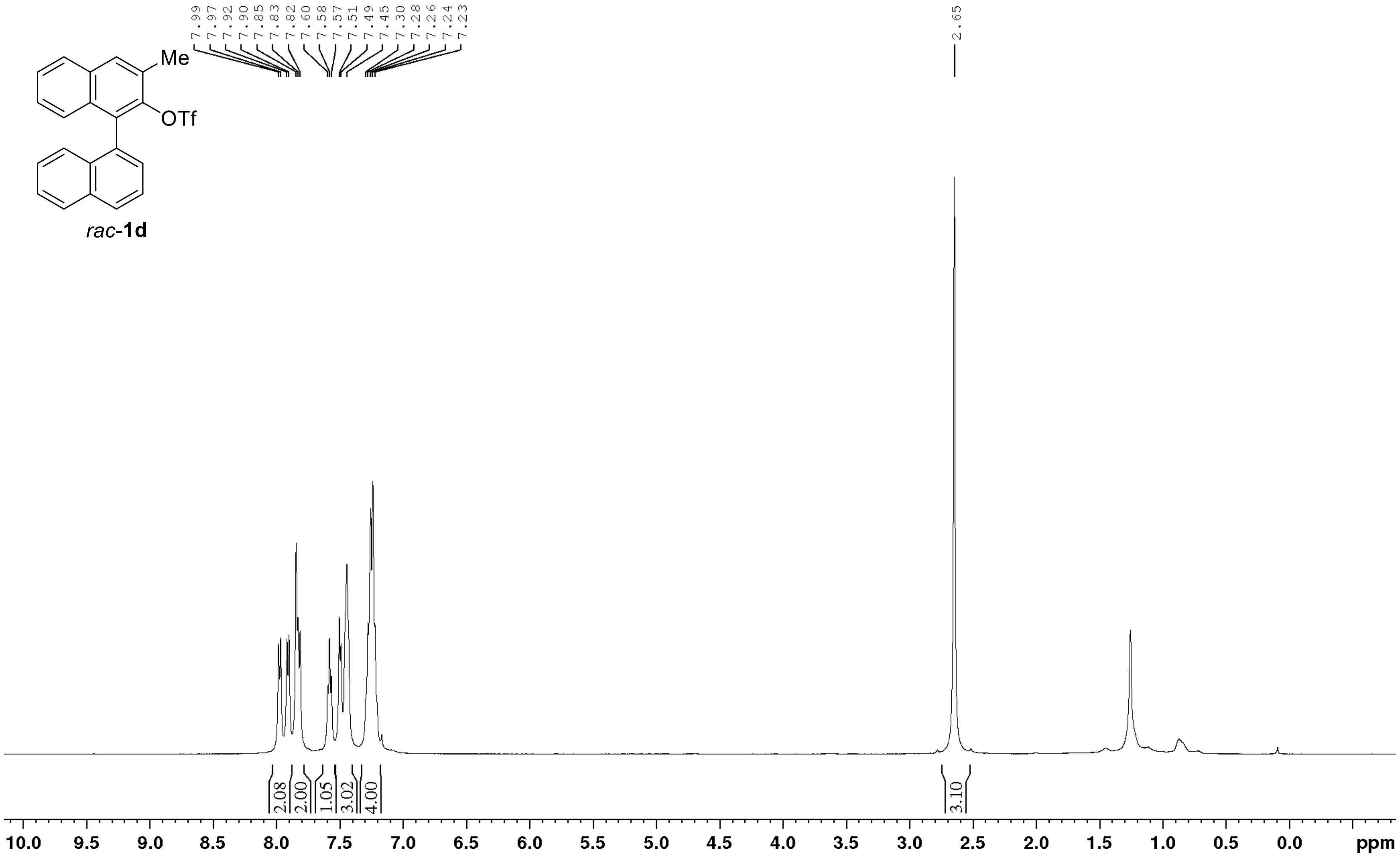

$^{13}C\{^1H\}$ NMR spectrum (126 MHz, $CDCl_3$, 298 K) of 3-methyl-[1,1'-binaphthalen]-2-yl trifluoromethanesulfonate (*rac*-**1d**)

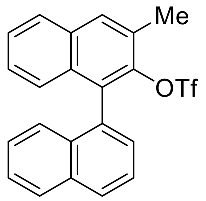

rac-**1d**

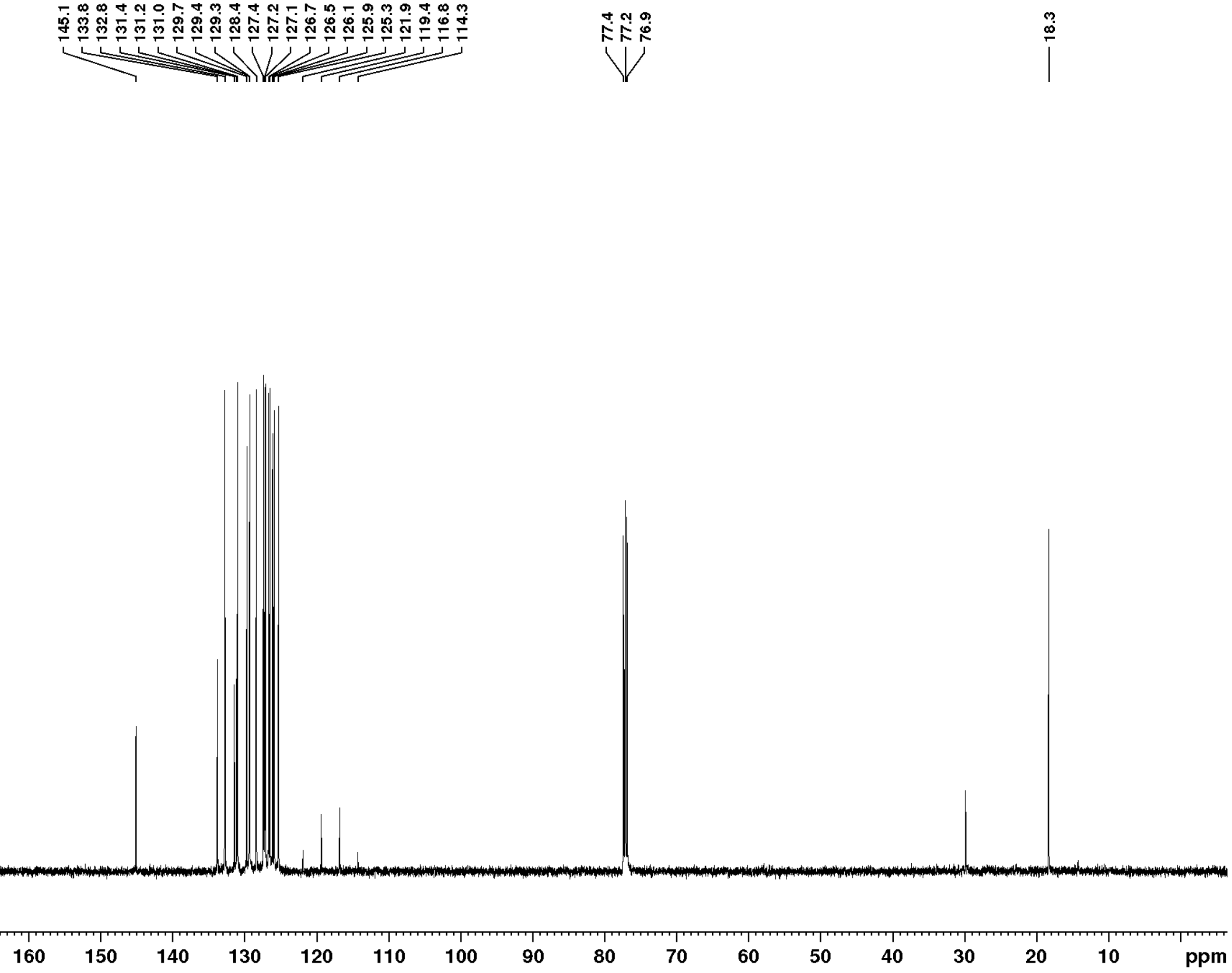

^{19}F NMR spectrum (471 MHz, $CDCl_3$, 298 K) of 3-methyl-[1,1'-binaphthalen]-2-yl trifluoromethanesulfonate (*rac*-**1d**)

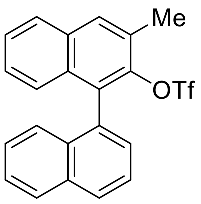

rac-**1d**

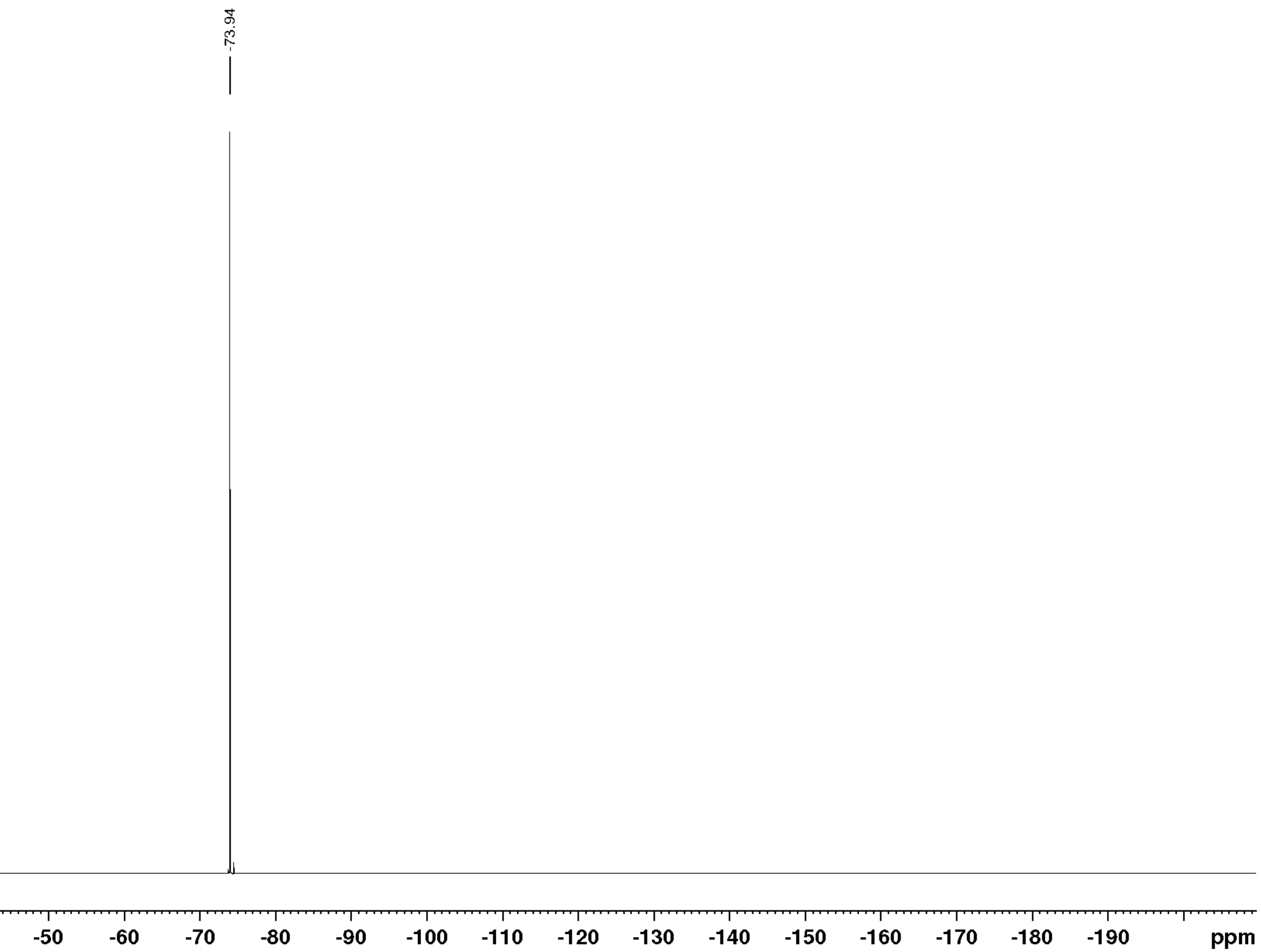

^{1}H NMR spectrum (500 MHz, $CDCl_3$, 298 K) of 3-fluoro-[1,1'-binaphthalen]-2-yl trifluoromethanesulfonate (*rac*-**1e**)

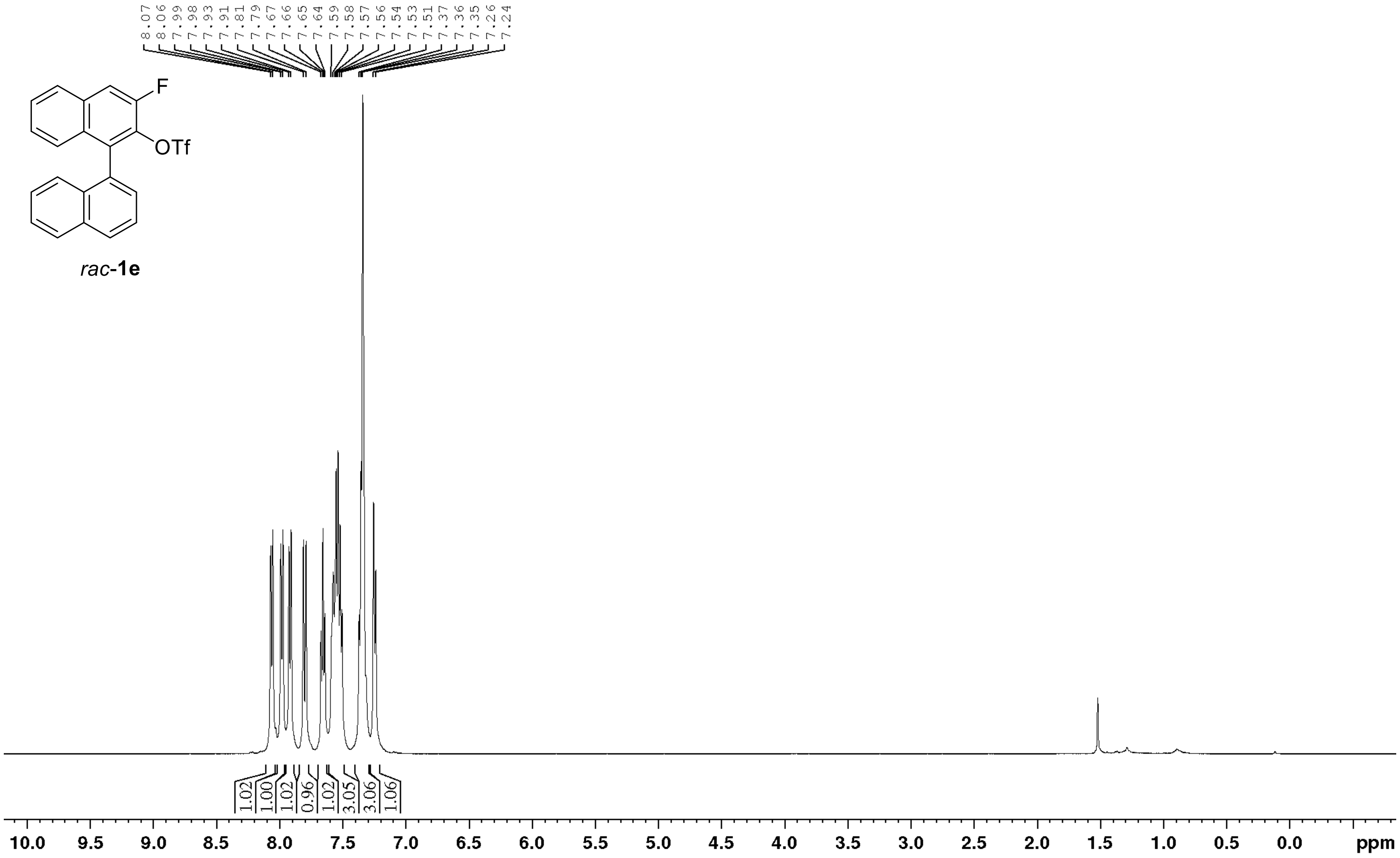

$^{13}C\{^1H\}$ NMR spectrum (126 MHz, $CDCl_3$, 298 K) of 3-fluoro-[1,1'-binaphthalen]-2-yl trifluoromethanesulfonate (*rac*-**1e**)

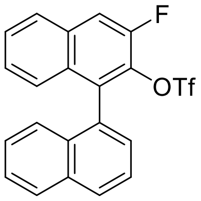

rac-**1e**

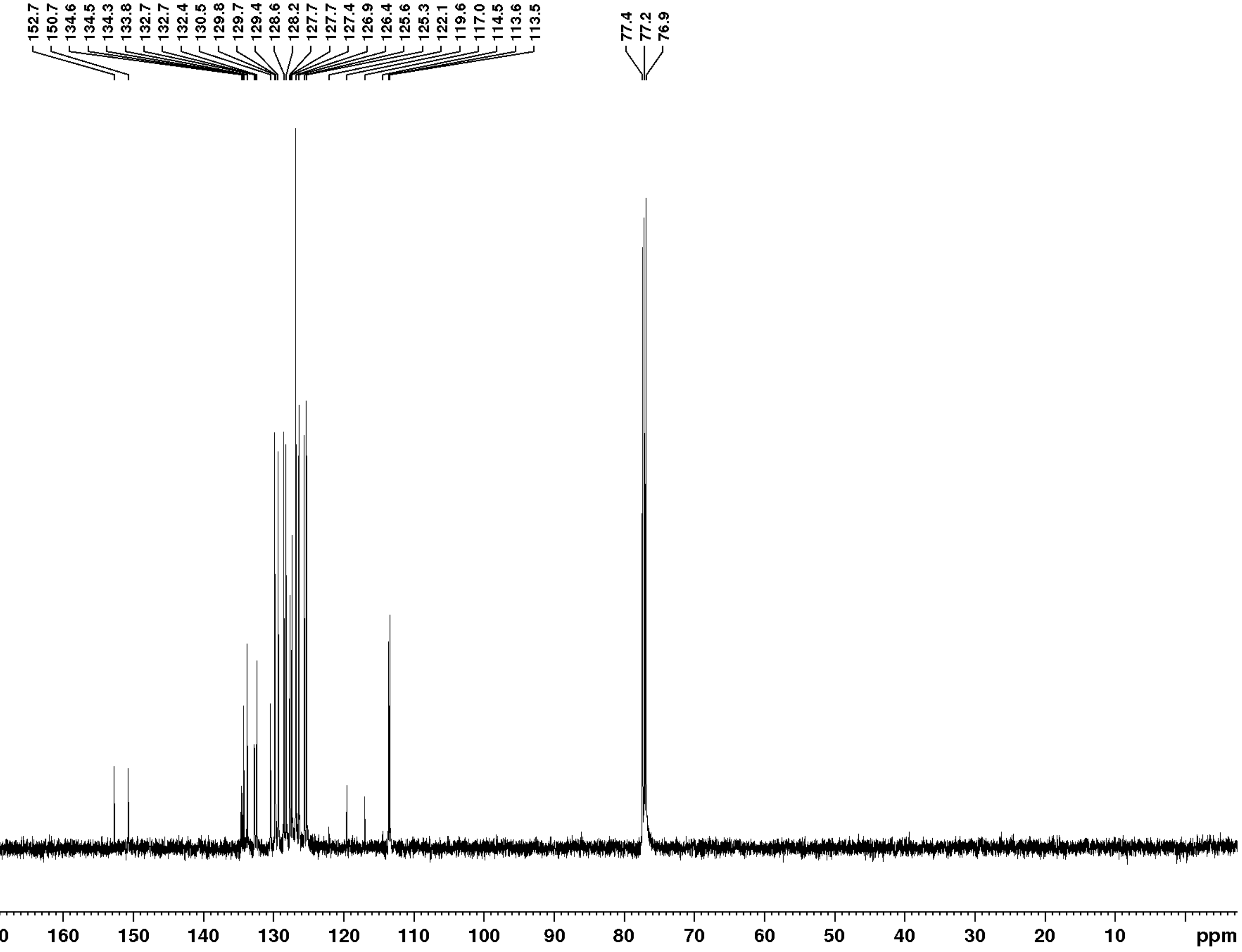

^{19}F NMR spectrum (471 MHz, $CDCl_3$, 298 K) of 3-fluoro-[1,1'-binaphthalen]-2-yl trifluoromethanesulfonate (*rac*-**1e**)

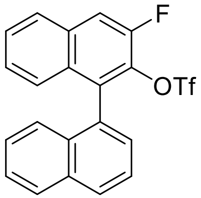

rac-**1e**

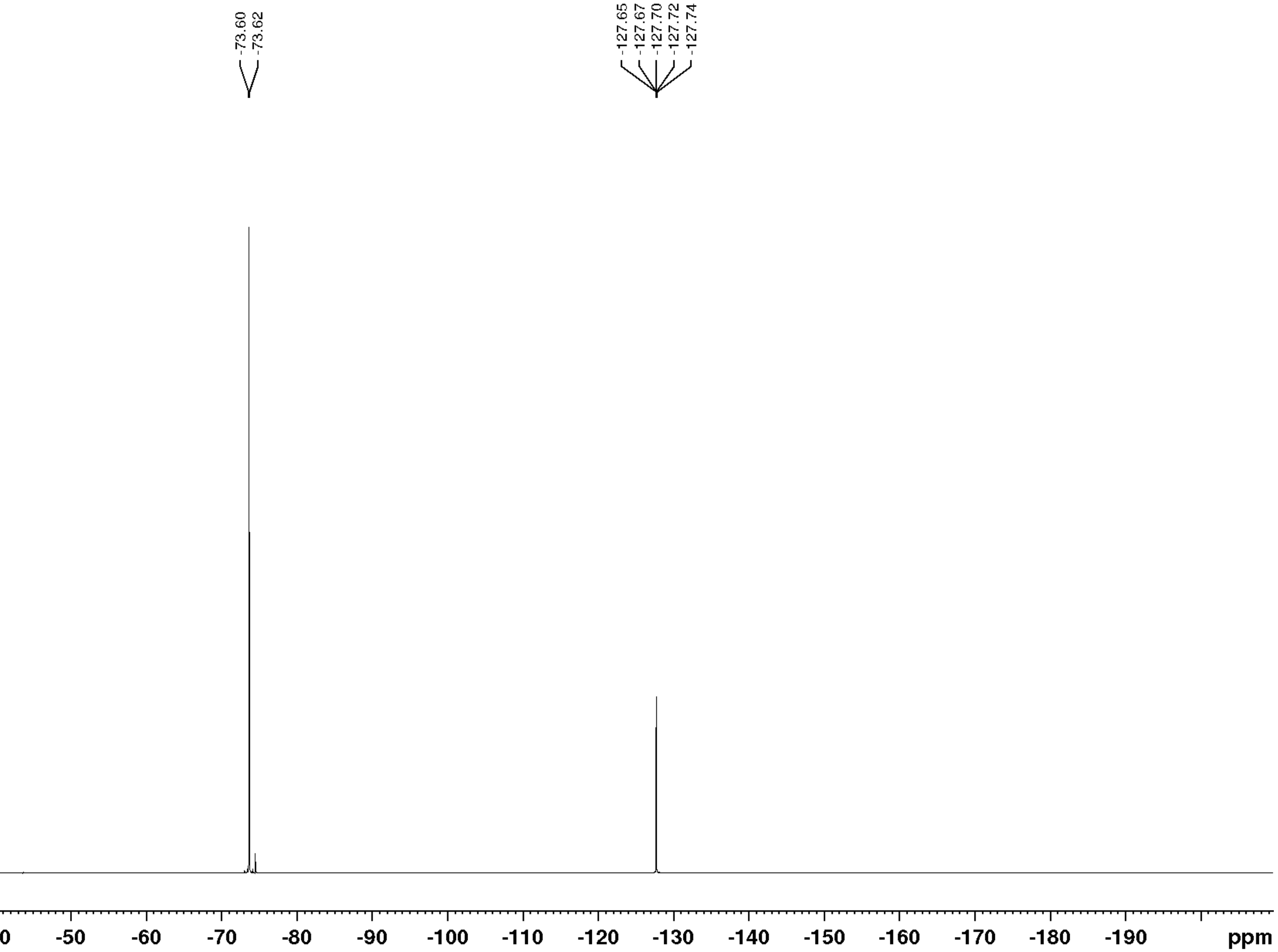

^{1}H NMR spectrum (500 MHz, $CDCl_3$, 298 K) of 1-(phenanthren-9-yl)naphthalen-2-yl trifluoromethanesulfonate (*rac*-**1f**)

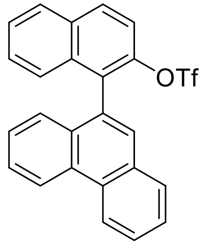

rac-**1f**

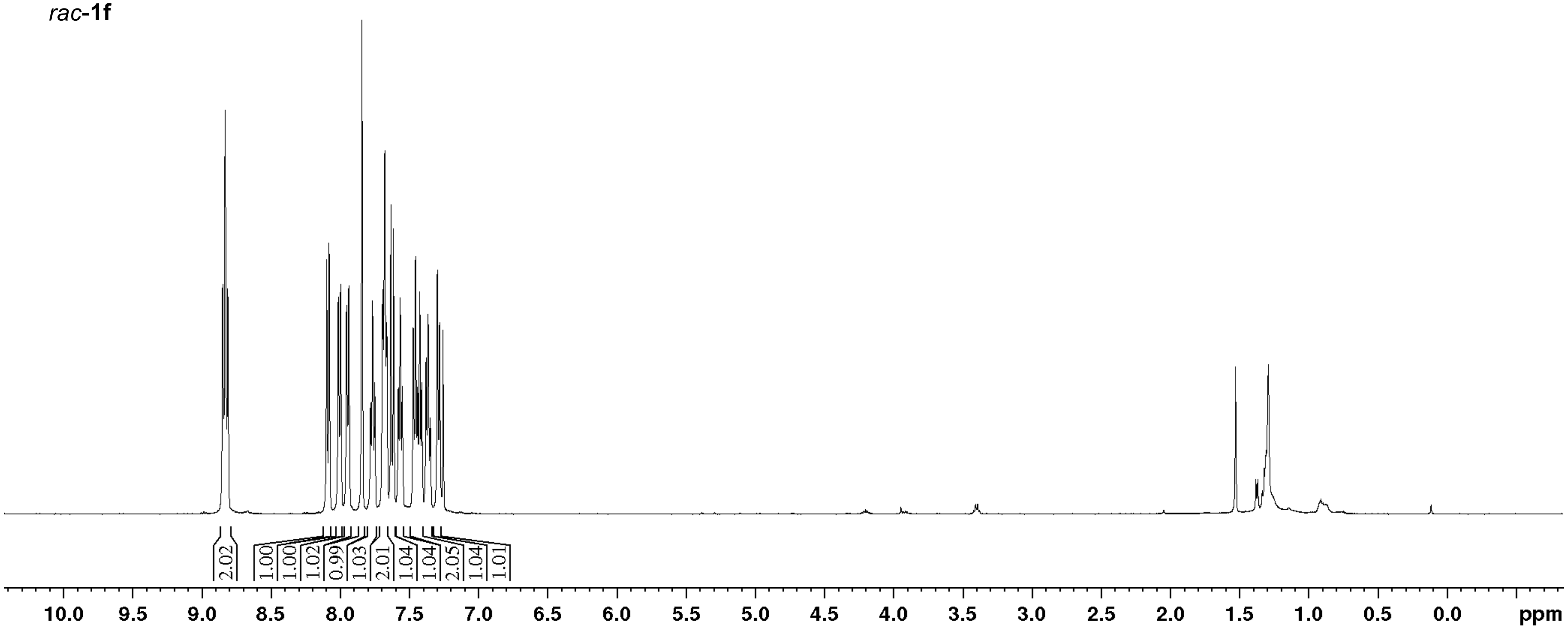

$^{13}C\{^{1}H\}$ NMR spectrum (126 MHz, $CDCl_3$, 298 K) of 1-(phenanthren-9-yl)naphthalen-2-yl trifluoromethanesulfonate (*rac*-**1f**)

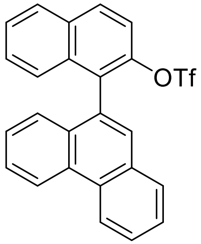

rac-**1f**

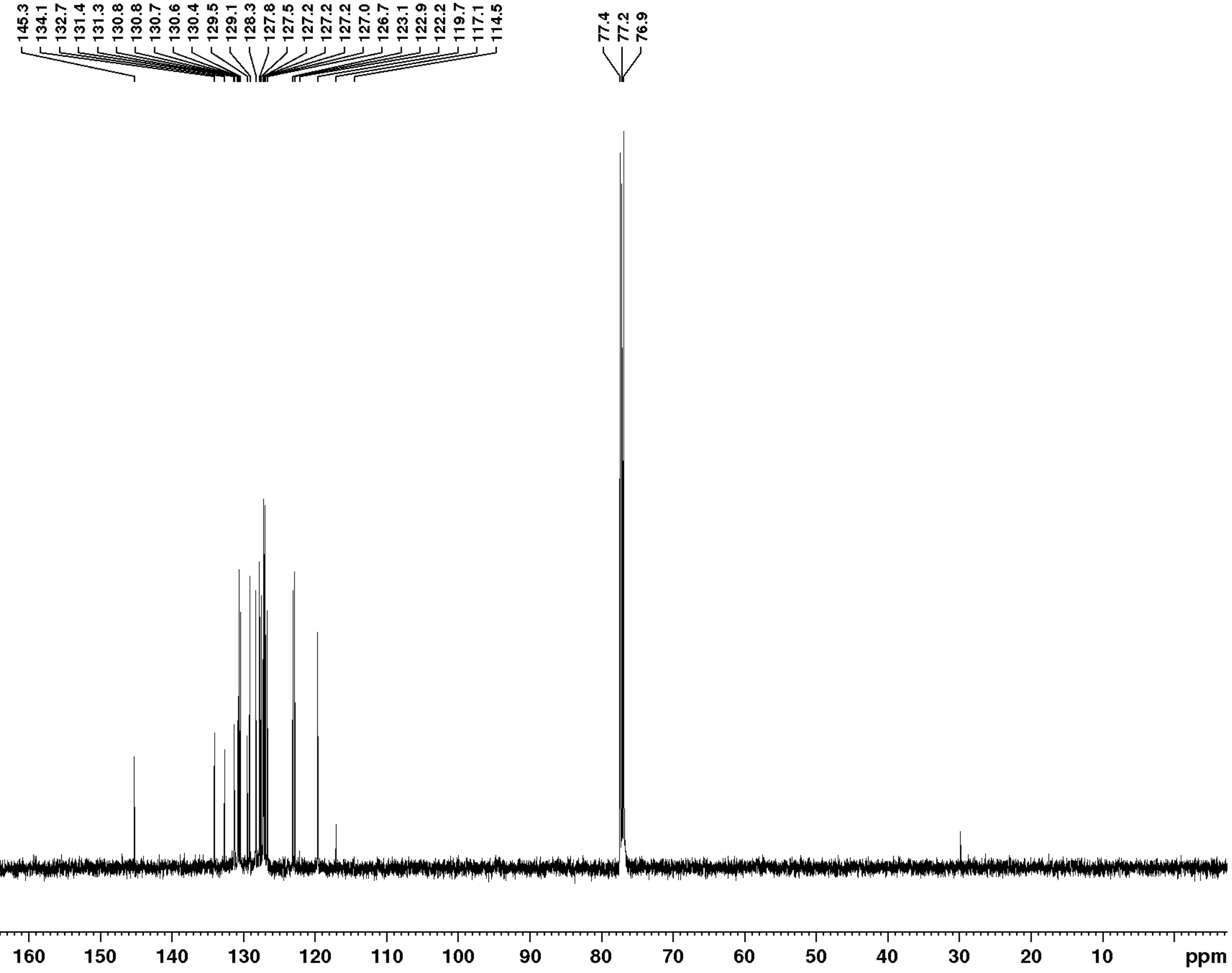

^{19}F NMR spectrum (471 MHz, $CDCl_3$, 298 K) of 1-(phenanthren-9-yl)naphthalen-2-yl trifluoromethanesulfonate (*rac*-**1f**)

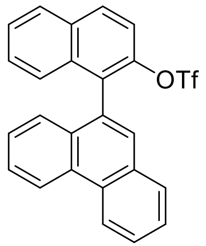

rac-**1f**

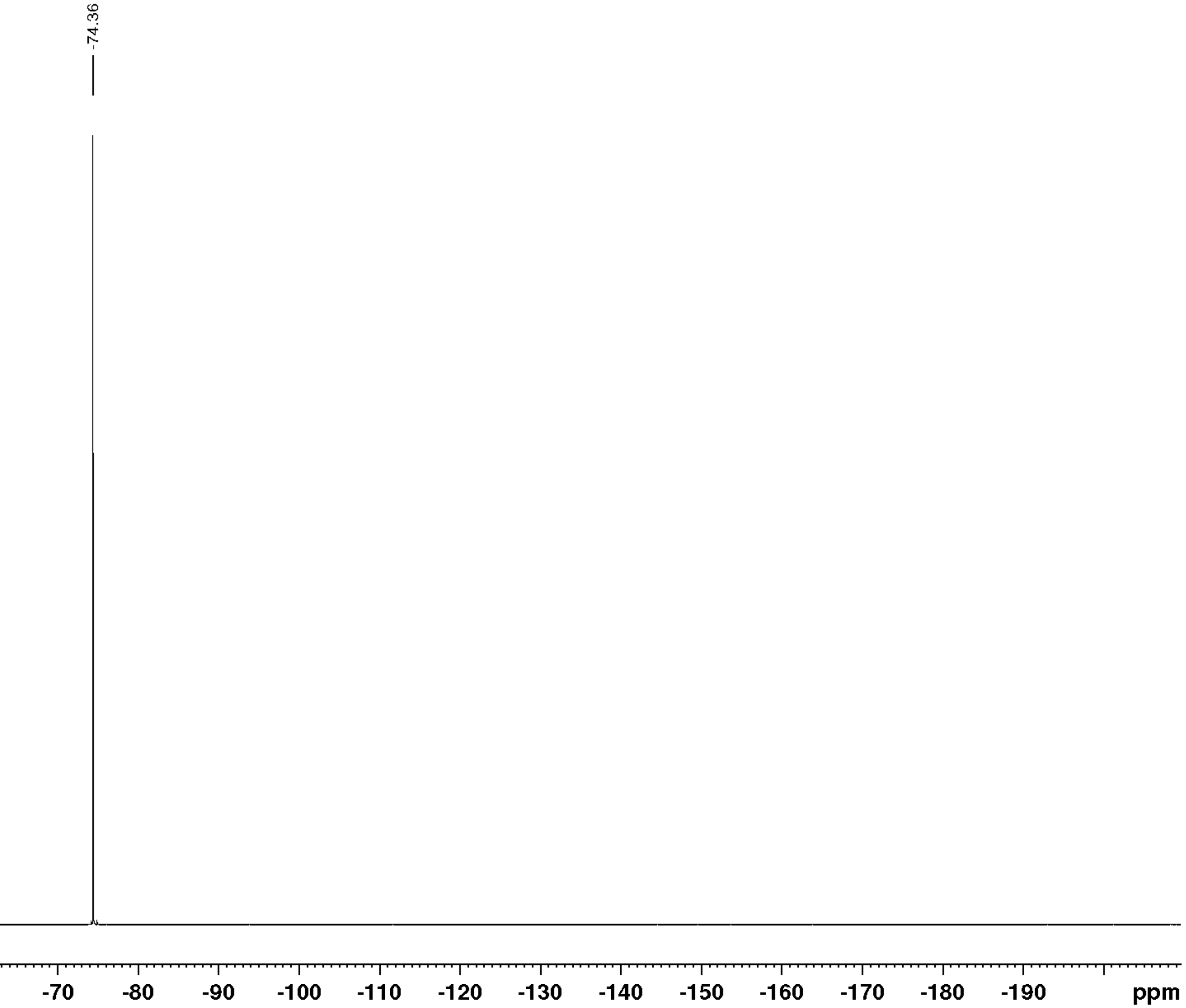

^{1}H NMR spectrum (500 MHz, $CDCl_3$, 298 K) of 1-(2-methoxyphenyl)naphthalen-2-yl trifluoromethanesulfonate (*rac*-**1g**)

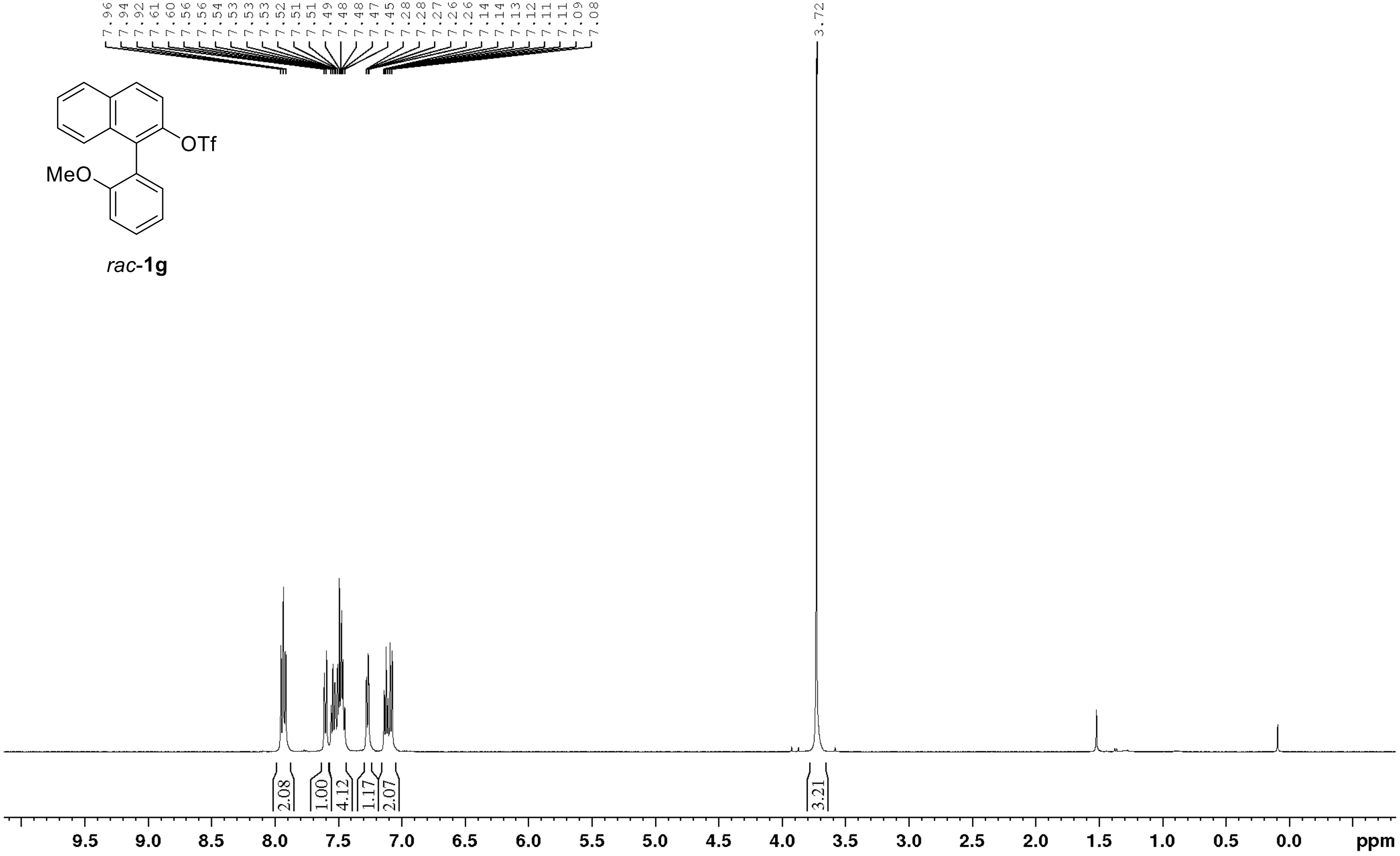

rac-**1g**

$^{13}C\{^1H\}$ NMR spectrum (126 MHz, $CDCl_3$, 298 K) of 1-(2-methoxyphenyl)naphthalen-2-yl trifluoromethanesulfonate (*rac*-**1g**)

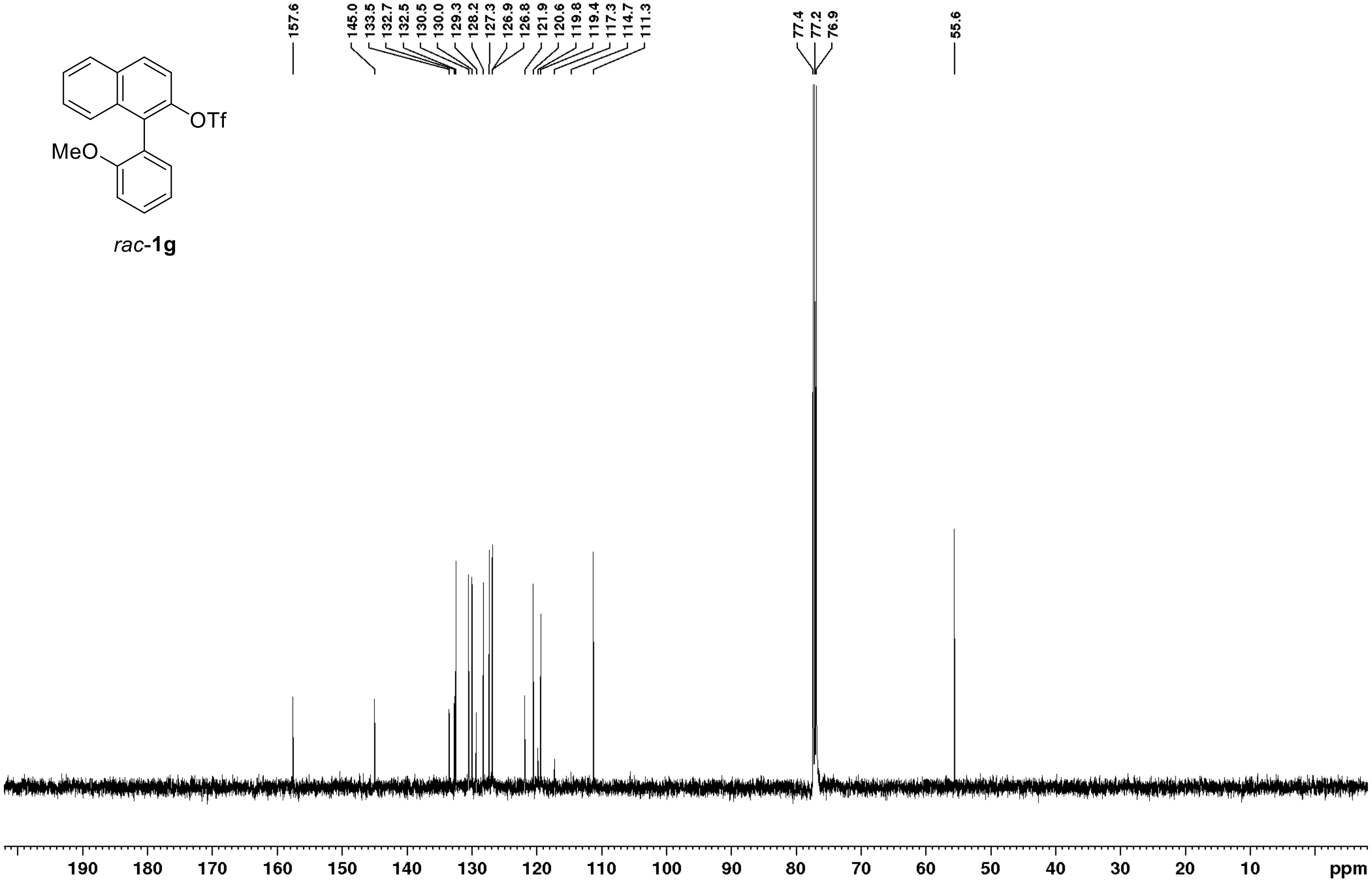

^{19}F NMR spectrum (471 MHz, $CDCl_3$, 298 K) of 1-(2-methoxyphenyl)naphthalen-2-yl trifluoromethanesulfonate (*rac*-**1g**)

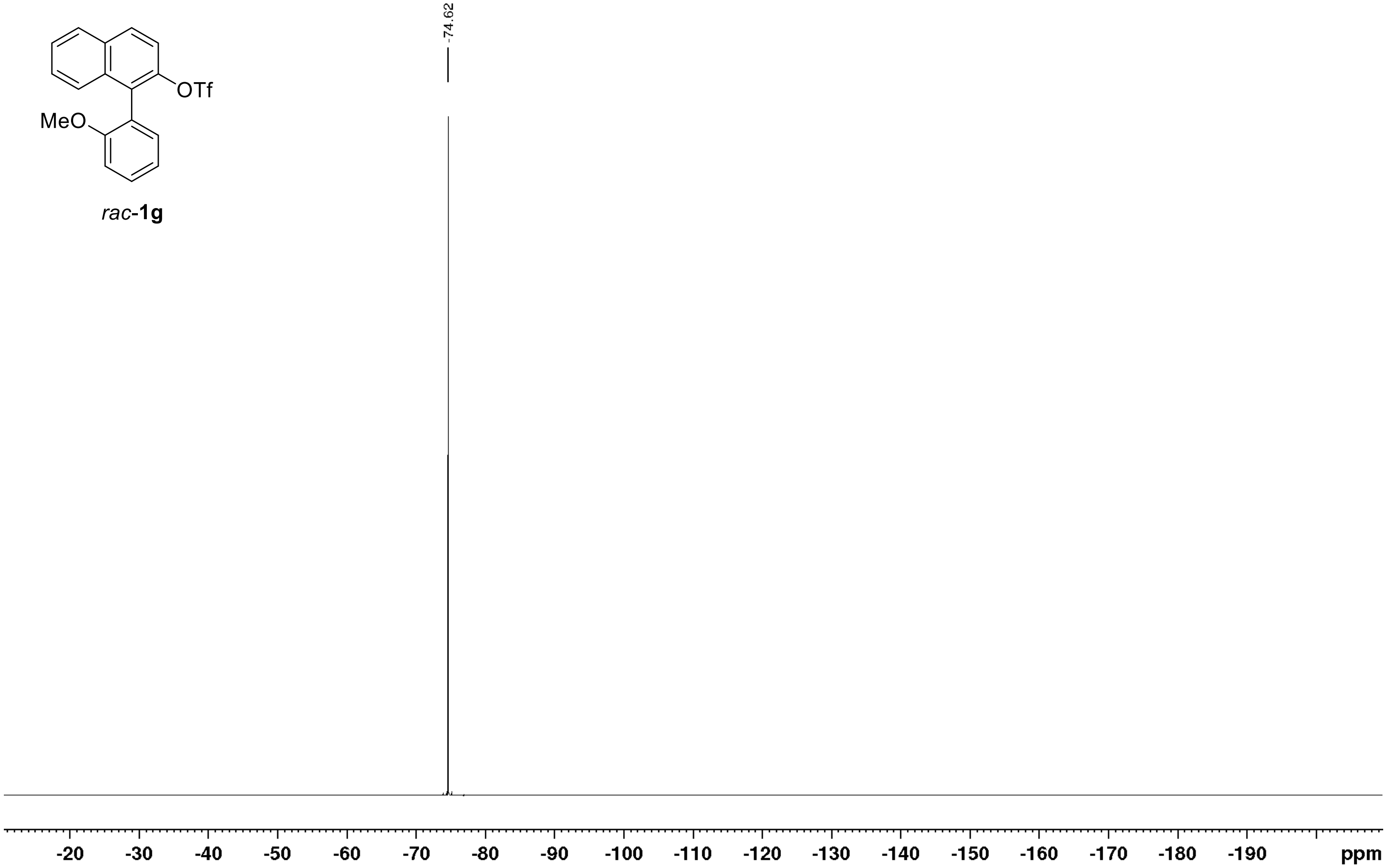

^1H NMR spectrum (500 MHz, $CDCl_3$, 298 K) of 1-(2-ethylphenyl)naphthalen-2-yl trifluoromethanesulfonate (*rac*-**1h**)

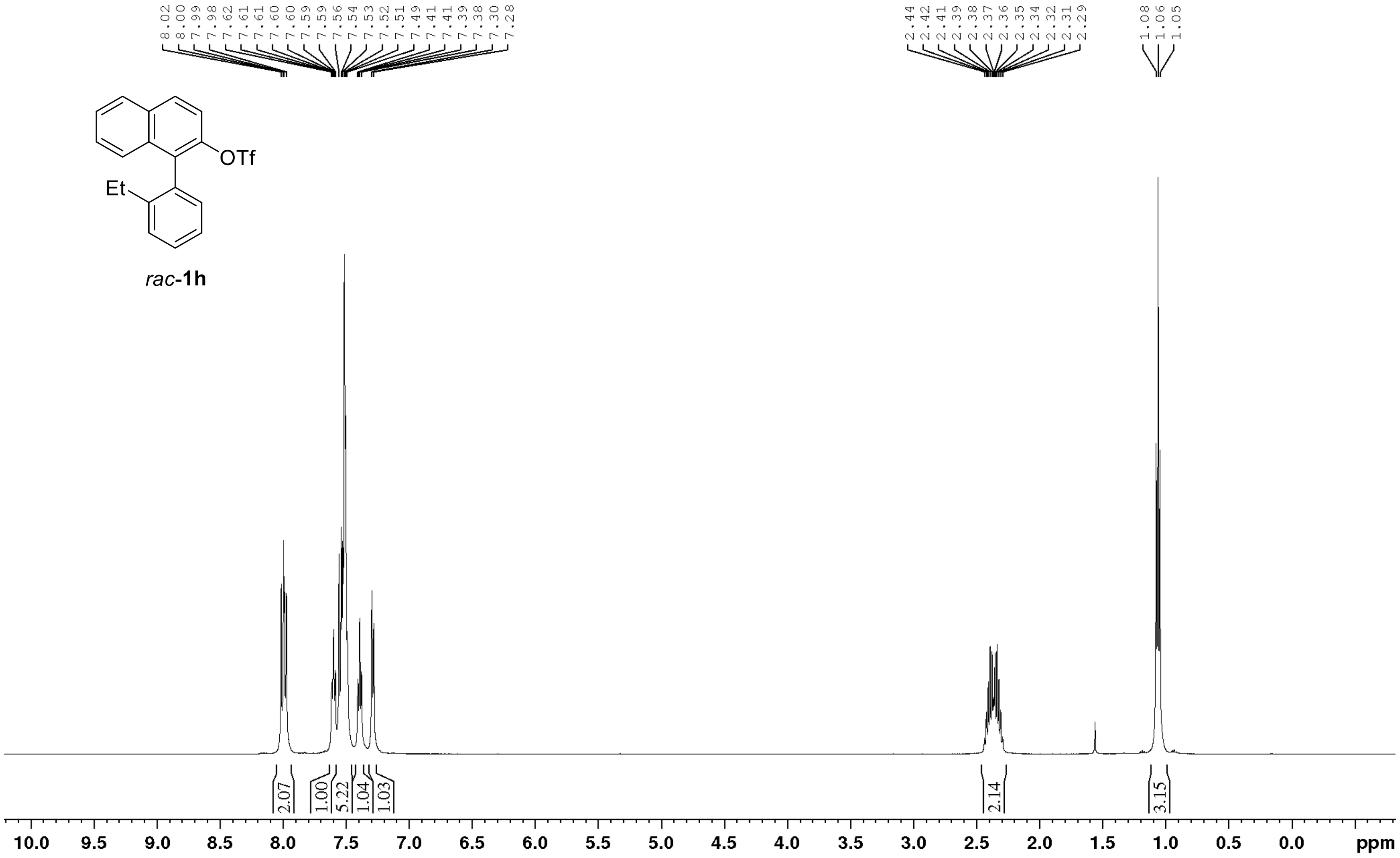

$^{13}C\{^1H\}$ NMR spectrum (126 MHz, $CDCl_3$, 298 K) of 1-(2-ethylphenyl)naphthalen-2-yl trifluoromethanesulfonate (*rac*-**1h**)

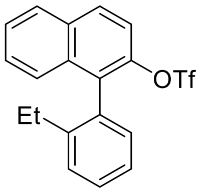

rac-**1h**

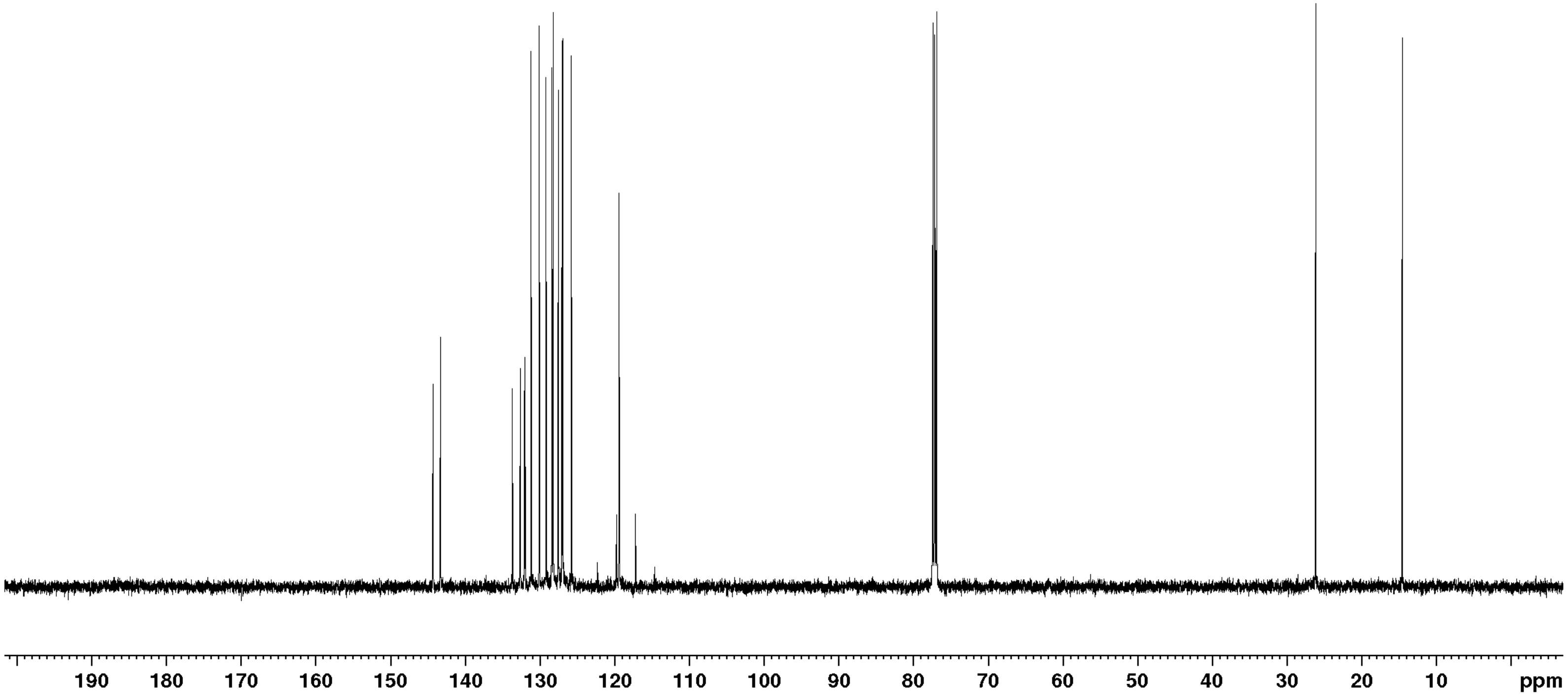

^{19}F NMR spectrum (471 MHz, $CDCl_3$, 298 K) of 1-(2-ethylphenyl)naphthalen-2-yl trifluoromethanesulfonate (*rac*-**1h**)

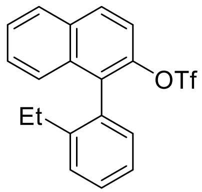

rac-**1h**

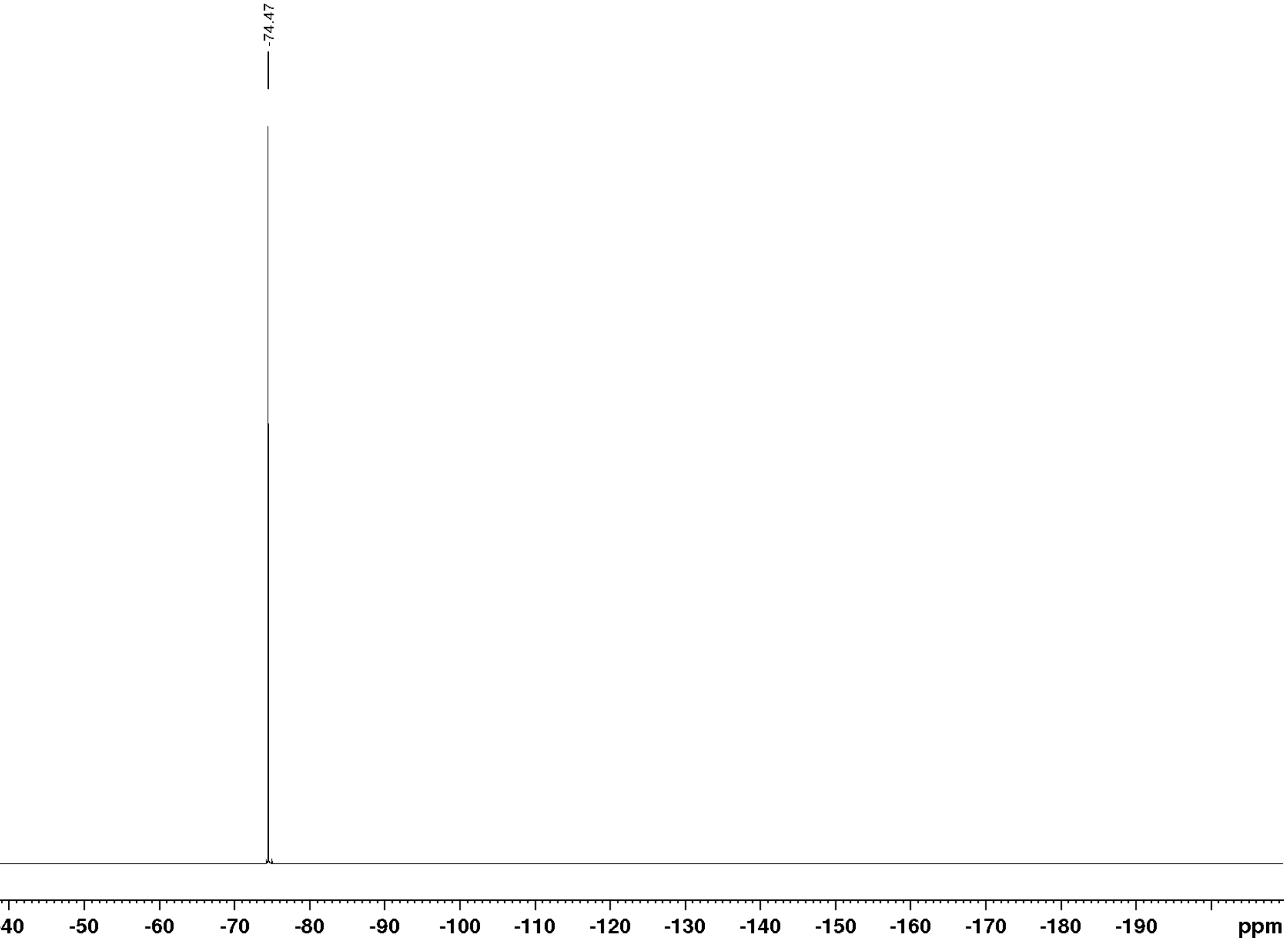

^{1}H NMR spectrum (500 MHz, $CDCl_3$, 298 K) of 1-(o-tolyl)naphthalen-2-yl trifluoromethanesulfonate (*rac*-**1i**)

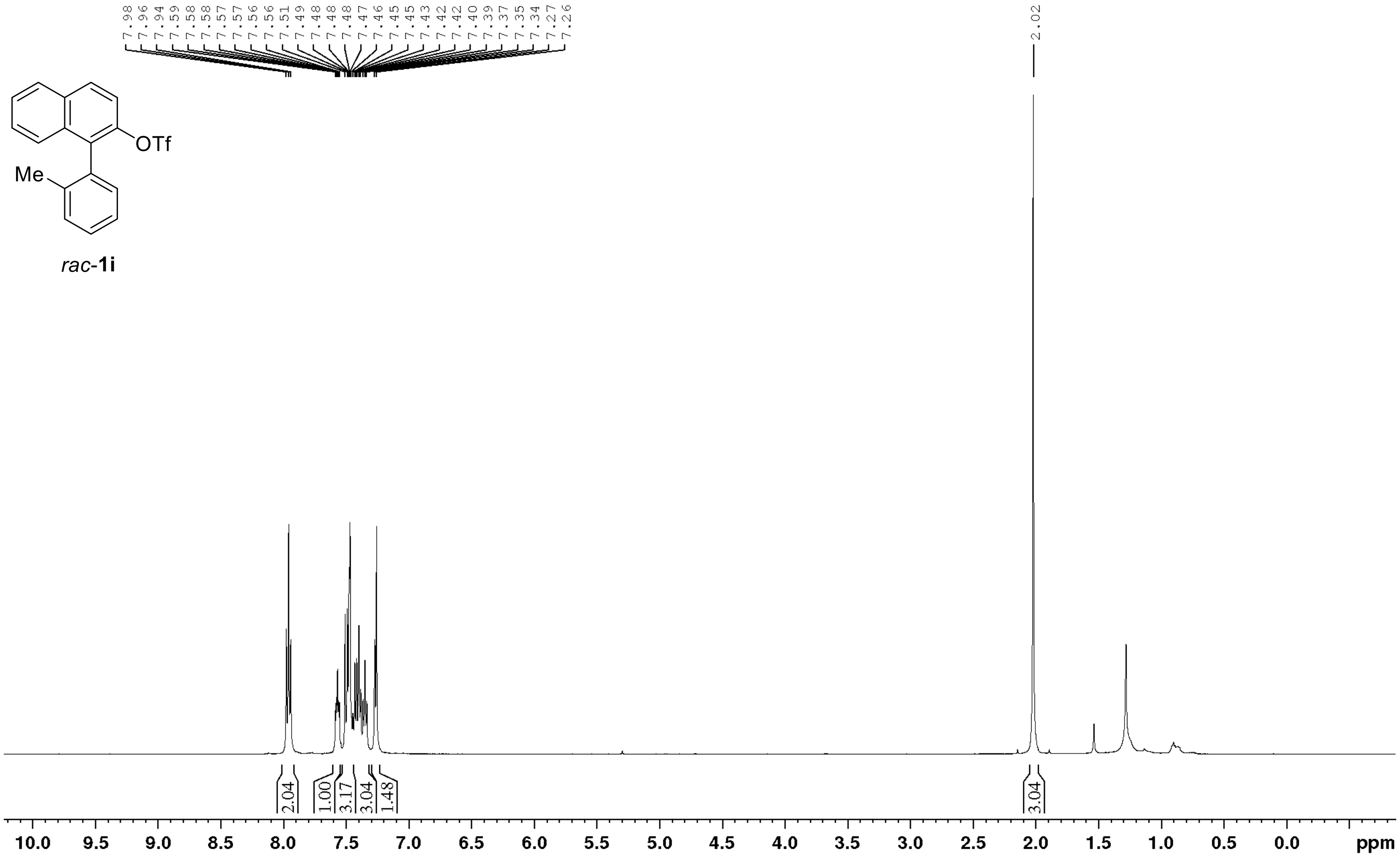

$^{13}C\{^{1}H\}$ NMR spectrum (126 MHz, $CDCl_3$, 298 K) of 1-(o-tolyl)naphthalen-2-yl trifluoromethanesulfonate (*rac*-**1i**)

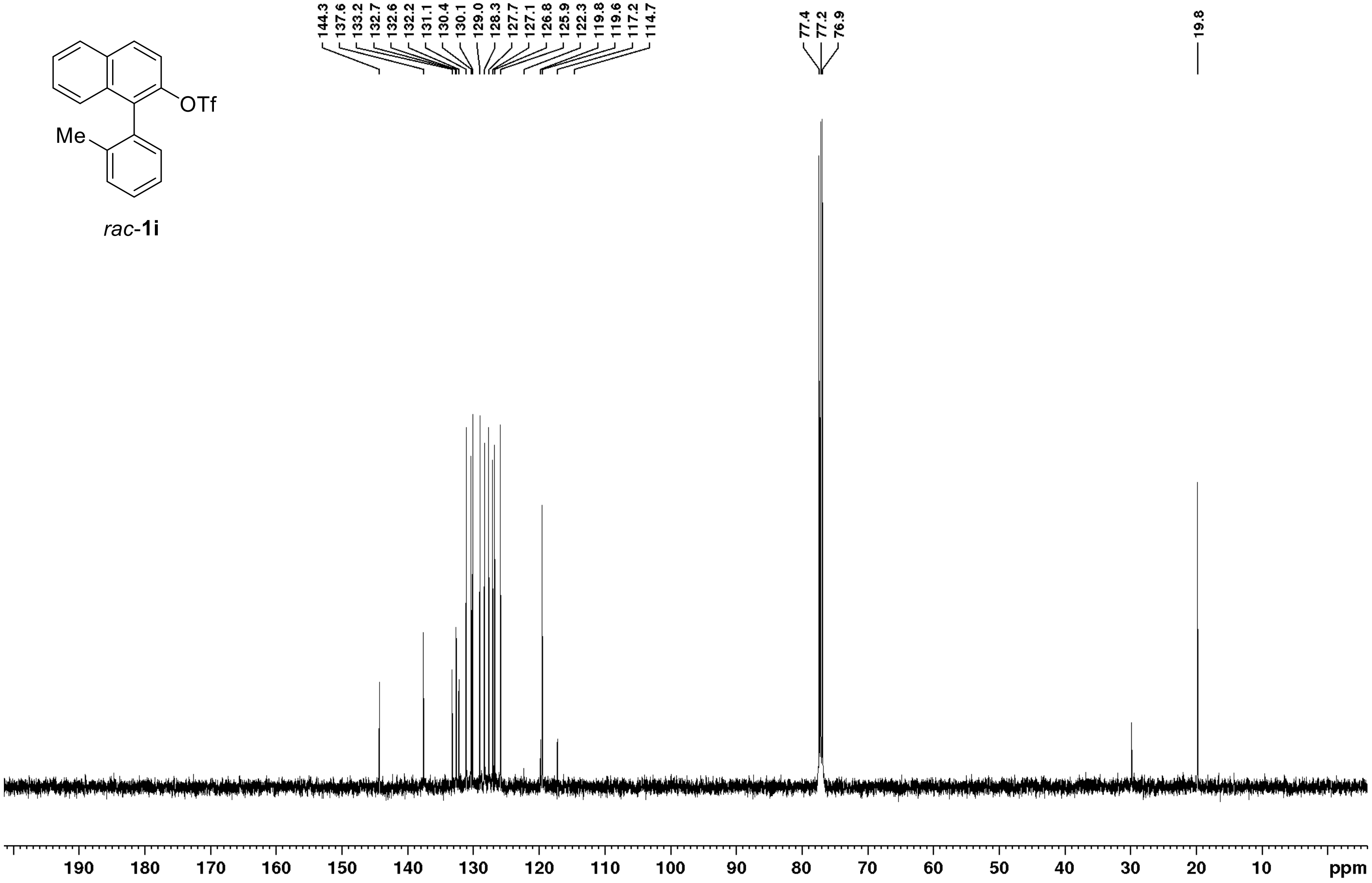

^{19}F NMR spectrum (471 MHz, $CDCl_3$, 298 K) of 1-(o-tolyl)naphthalen-2-yl trifluoromethanesulfonate (*rac*-**1i**)

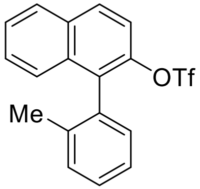

rac-**1i**

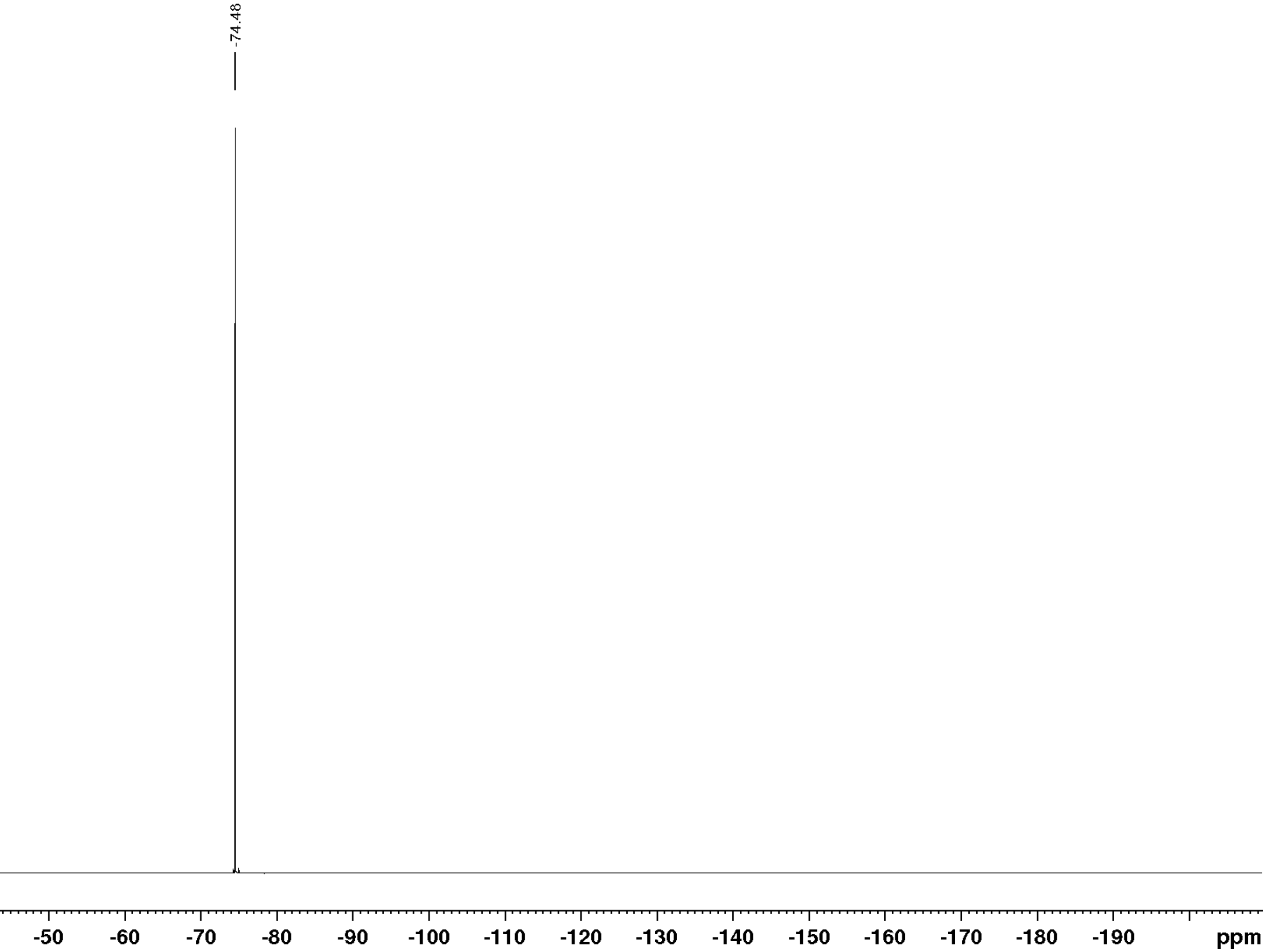

^{1}H NMR spectrum (500 MHz, $CDCl_3$, 298 K) of 1-(4-fluoro-2-methylphenyl)naphthalen-2-yl trifluoromethanesulfonate (*rac*-**1j**)

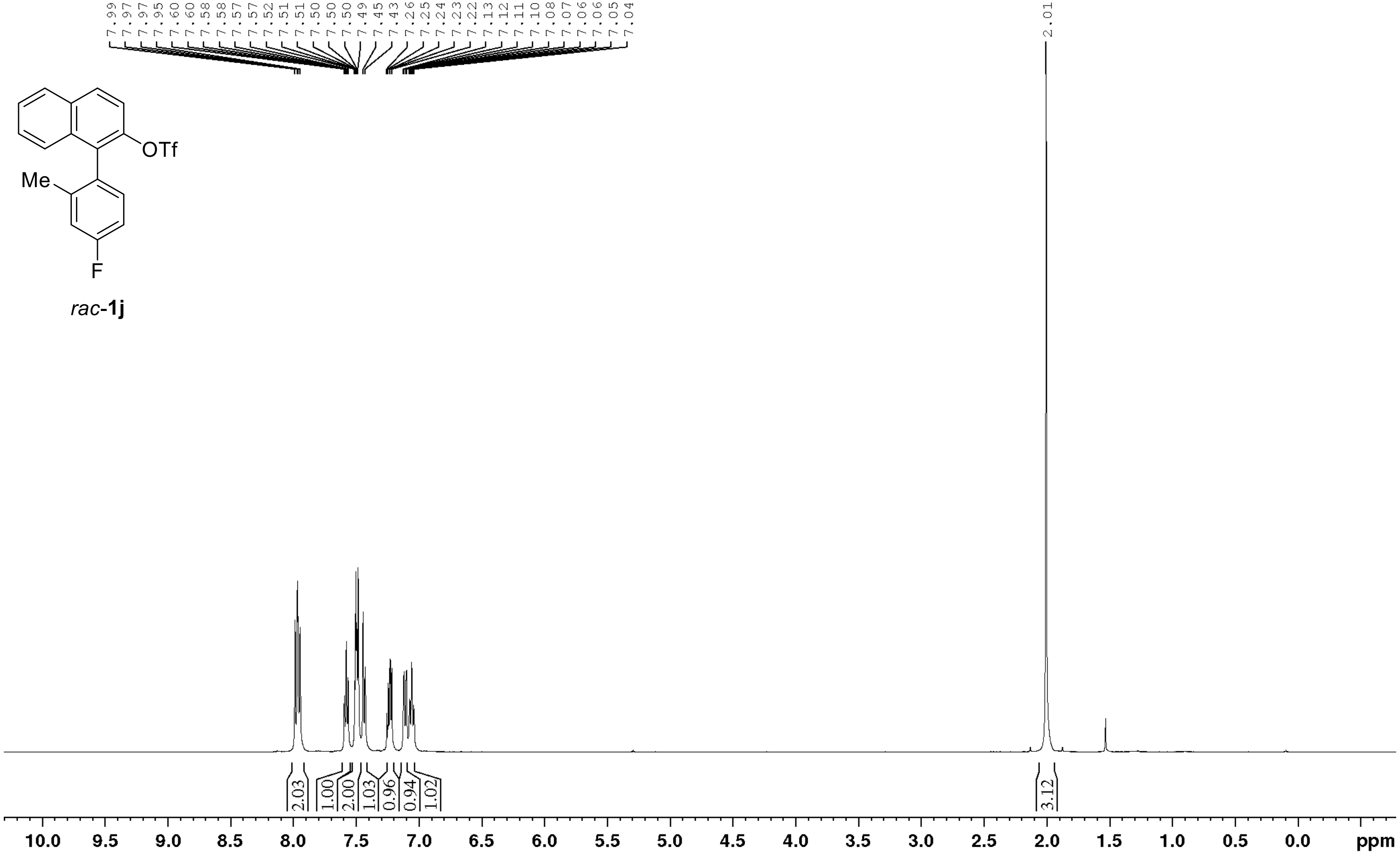

$^{13}C\{^{1}H\}$ NMR spectrum (126 MHz, $CDCl_3$, 298 K) of 1-(4-fluoro-2-methylphenyl)naphthalen-2-yl trifluoromethanesulfonate (*rac*-**1j**)

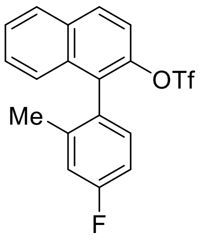

rac-**1j**

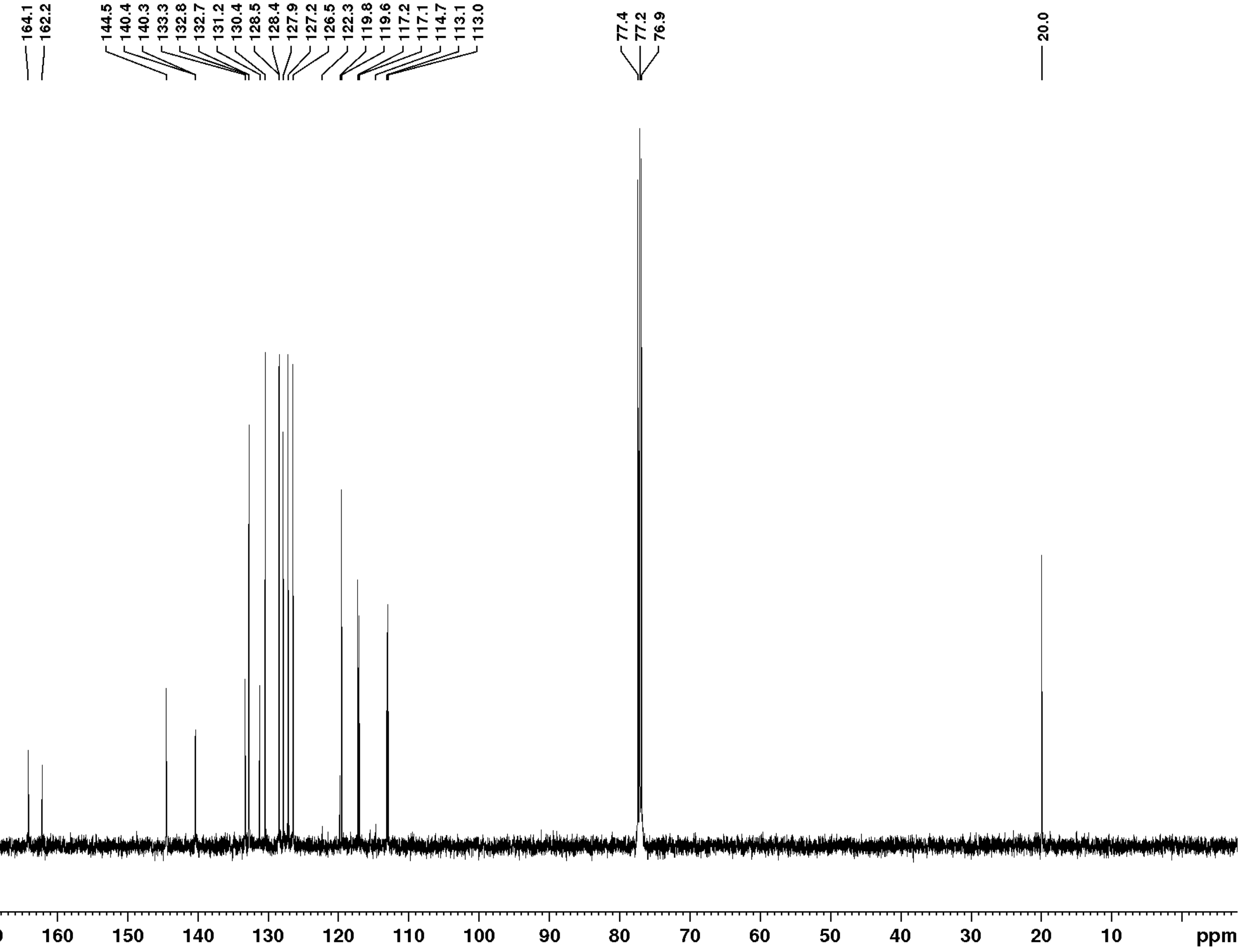

^{19}F NMR spectrum (471 MHz, $CDCl_3$, 298 K) of 1-(4-fluoro-2-methylphenyl)naphthalen-2-yl trifluoromethanesulfonate (*rac*-**1j**)

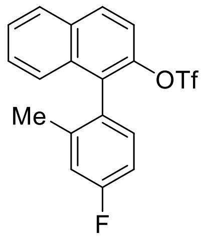

rac-**1j**

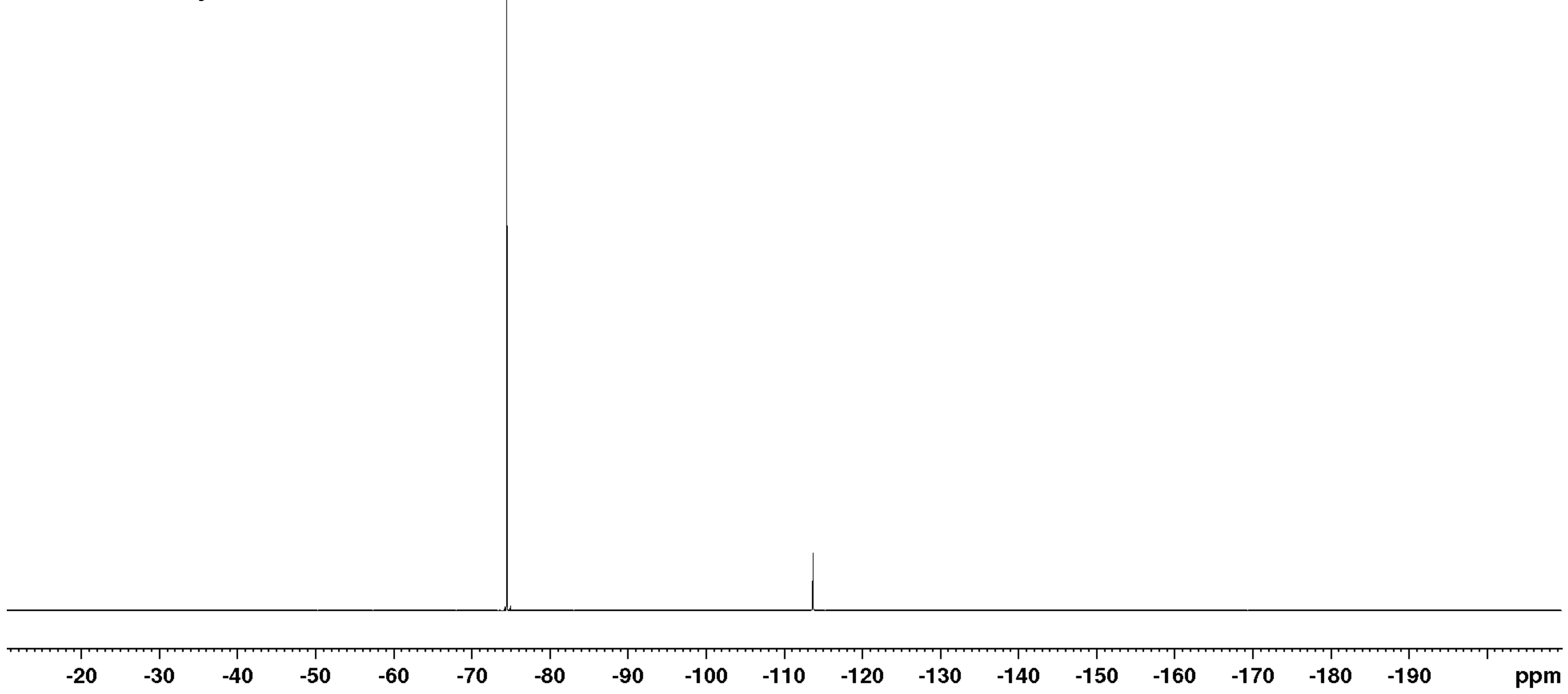

^{1}H NMR spectrum (500 MHz, $CDCl_3$, 298 K) of 1-(2-chlorophenyl)naphthalen-2-yl trifluoromethanesulfonate (*rac*-**1k**)

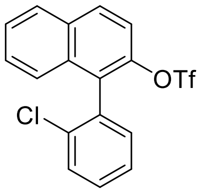

rac-**1k**

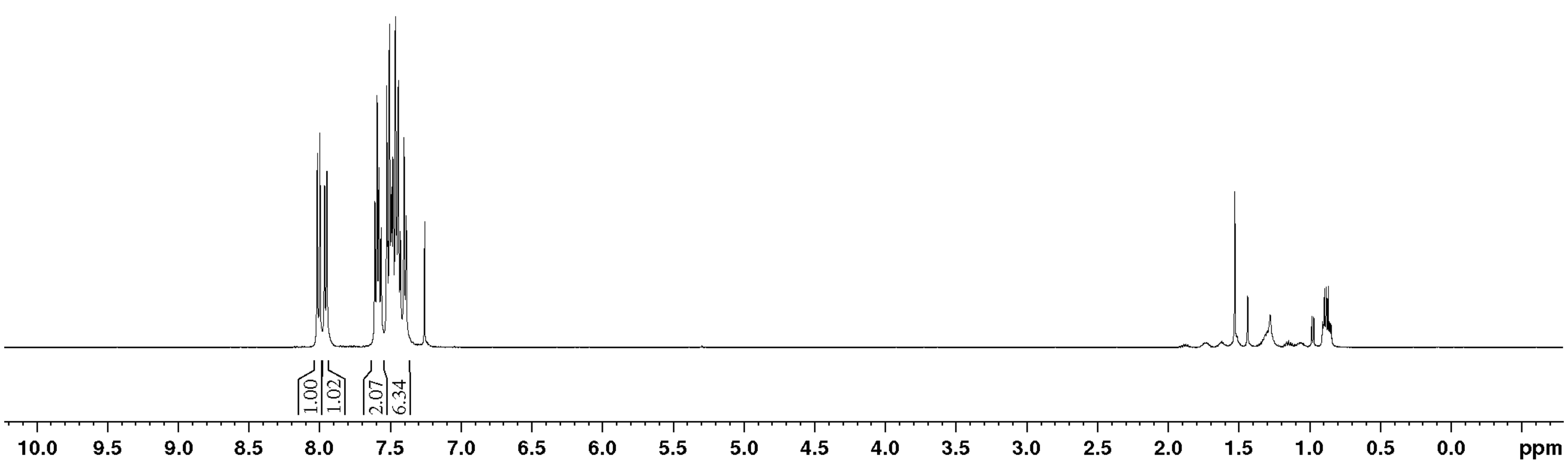

$^{13}C\{^1H\}$ NMR spectrum (126 MHz, $CDCl_3$, 298 K) of 1-(2-chlorophenyl)naphthalen-2-yl trifluoromethanesulfonate (*rac*-**1k**)

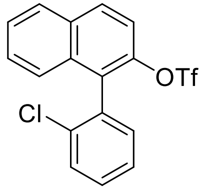

rac-**1k**

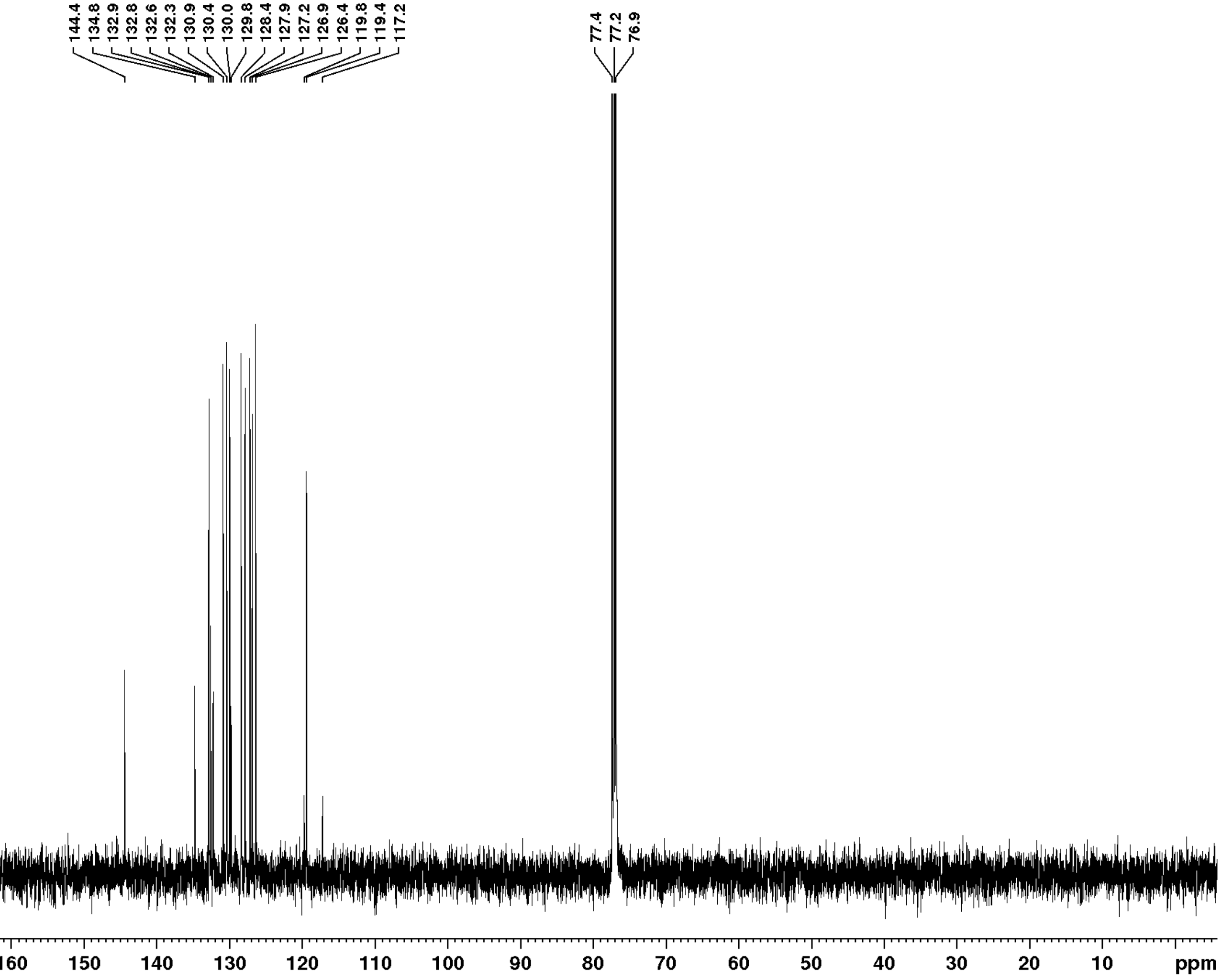

^{19}F NMR spectrum (471 MHz, $CDCl_3$, 298 K) of 1-(2-chlorophenyl)naphthalen-2-yl trifluoromethanesulfonate (*rac*-**1k**)

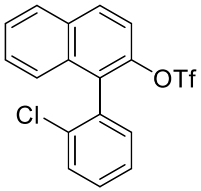

rac-**1k**

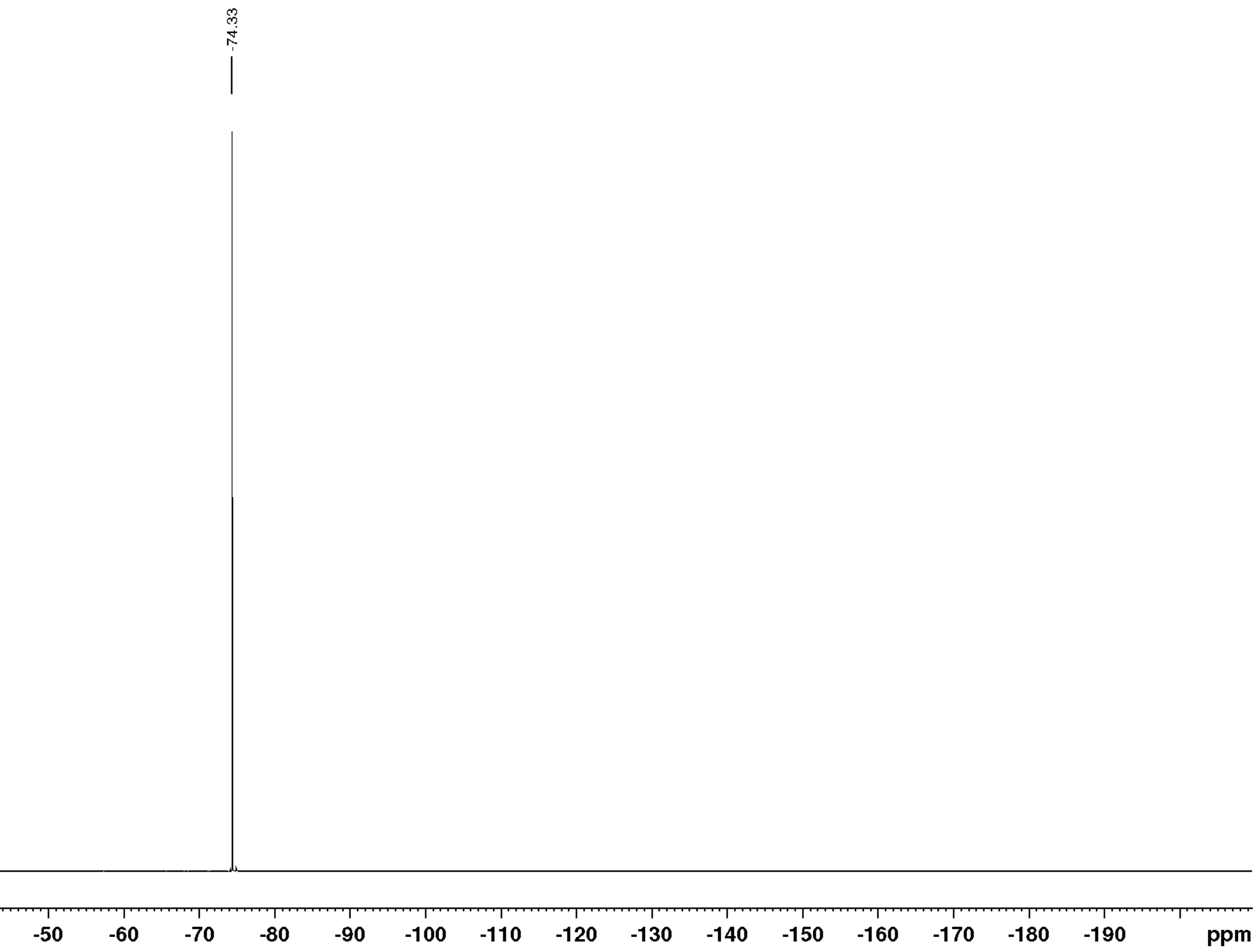

^1H NMR spectrum (500 MHz, $CDCl_3$, 298 K) of 1-([1,1'-biphenyl]-2-yl)naphthalen-2-yl trifluoromethanesulfonate (*rac*-**1l**)

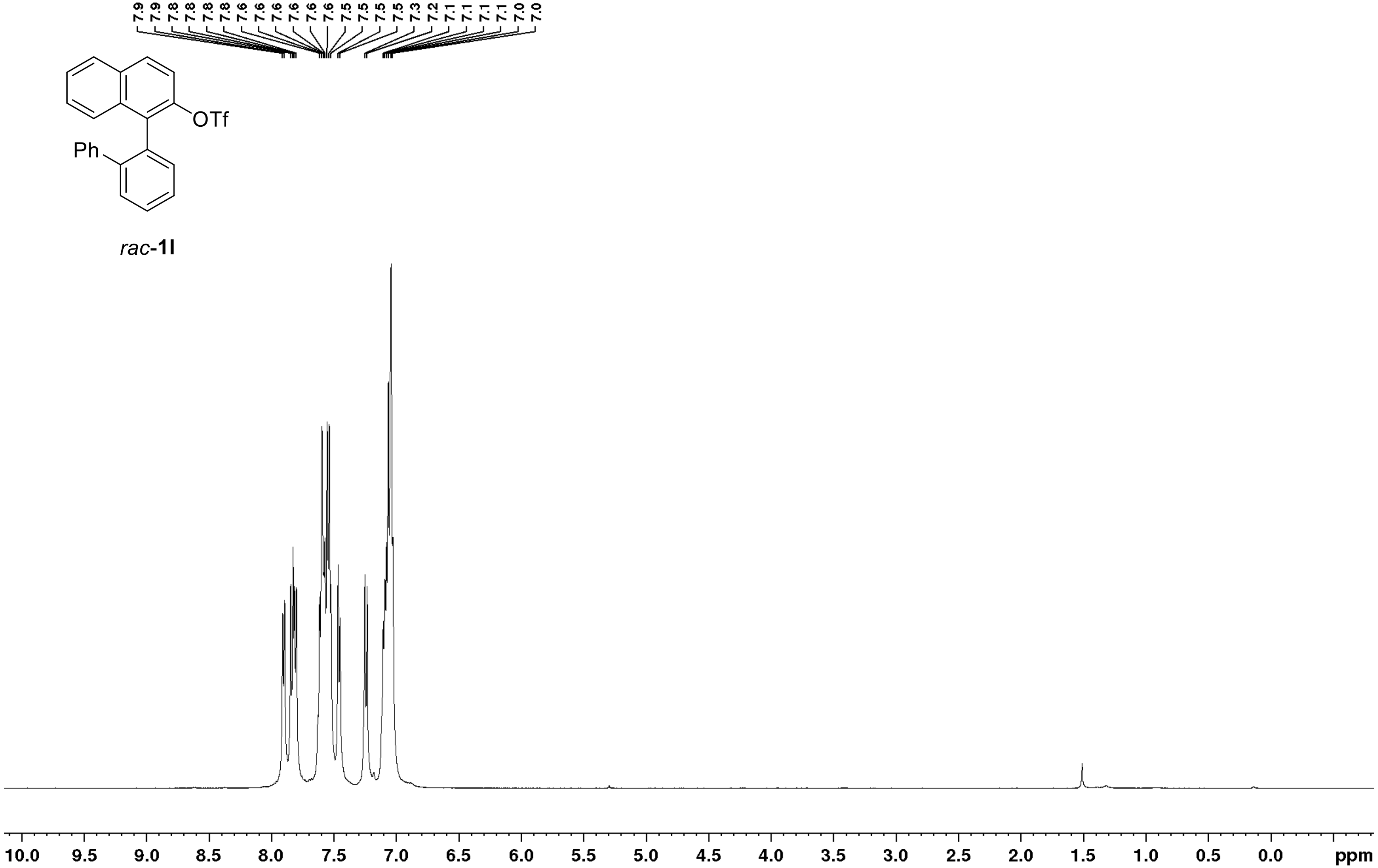

$^{13}C\{^1H\}$ NMR spectrum (126 MHz, $CDCl_3$, 298 K) of 1-([1,1'-biphenyl]-2-yl)naphthalen-2-yl trifluoromethanesulfonate (*rac*-**1l**)

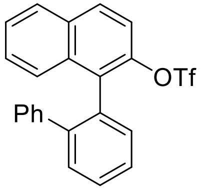

rac-**1l**

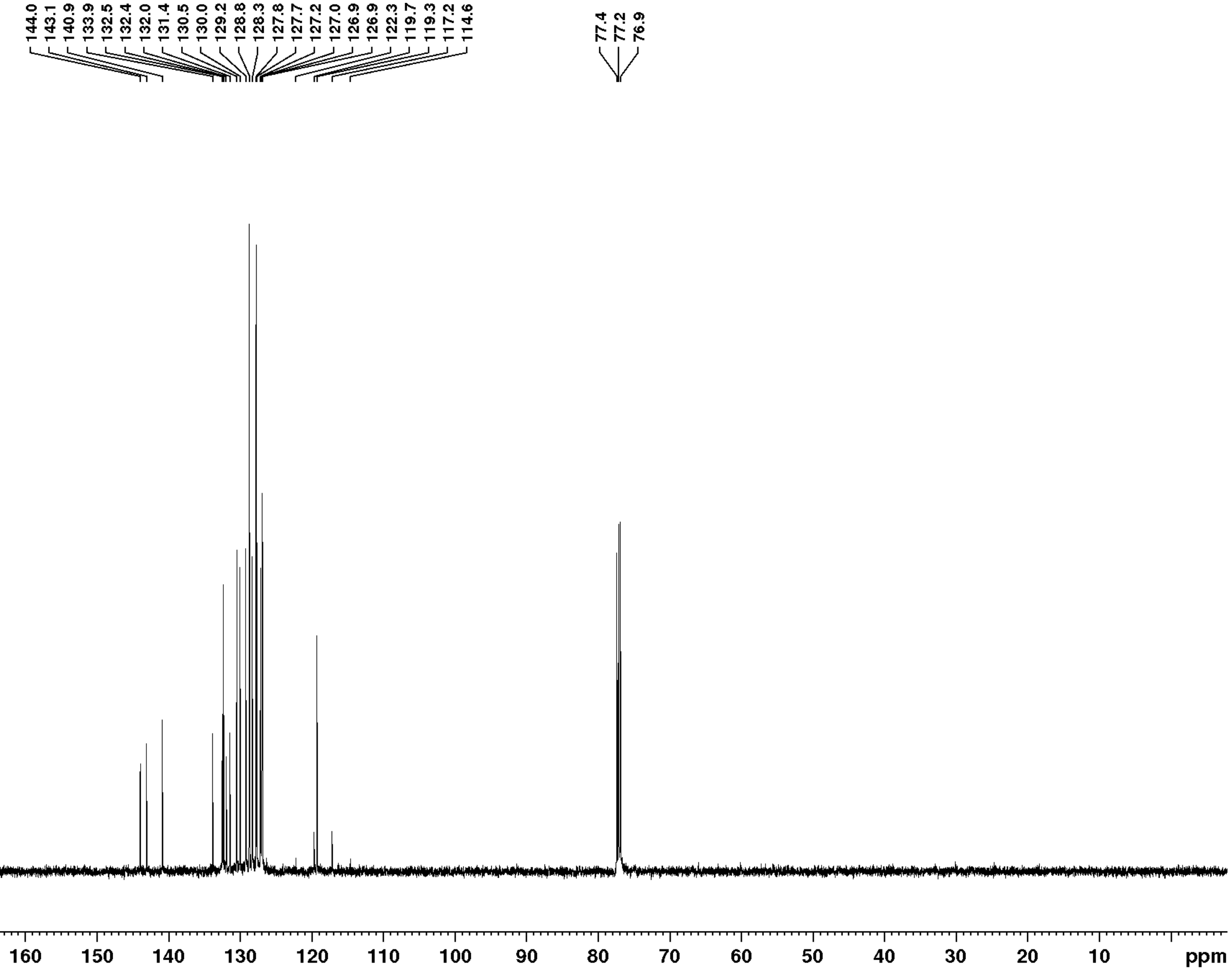

^{19}F NMR spectrum (471 MHz, $CDCl_3$, 298 K) of 1-([1,1'-biphenyl]-2-yl)naphthalen-2-yl trifluoromethanesulfonate (*rac*-**1l**)

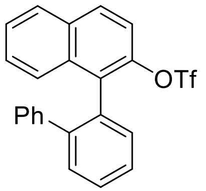

rac-**1l**

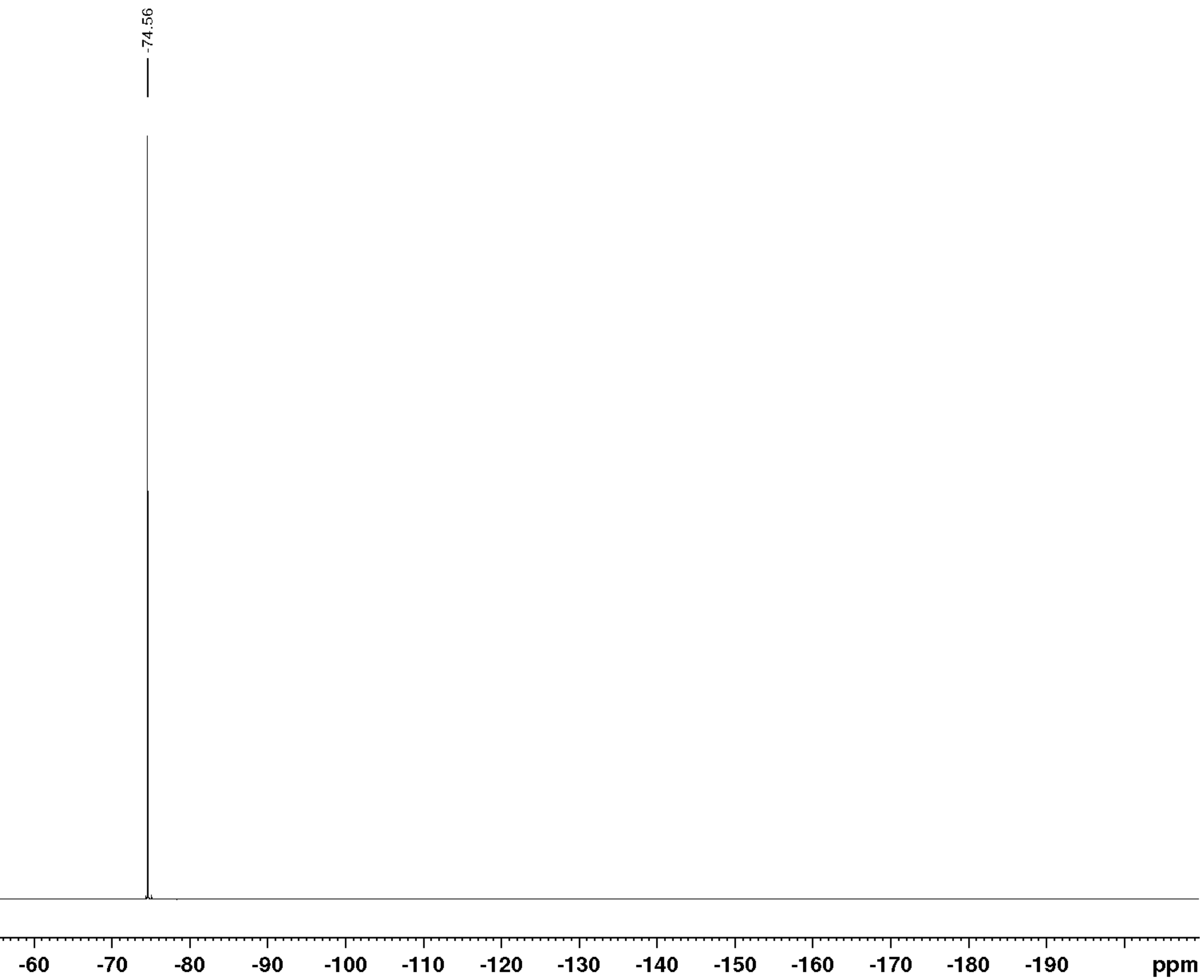

^{1}H NMR spectrum (500 MHz, $CDCl_3$, 298 K) of 3-fluoro-2-(naphthalen-1-yl)phenyl trifluoromethanesulfonate (*rac*-**1m**)

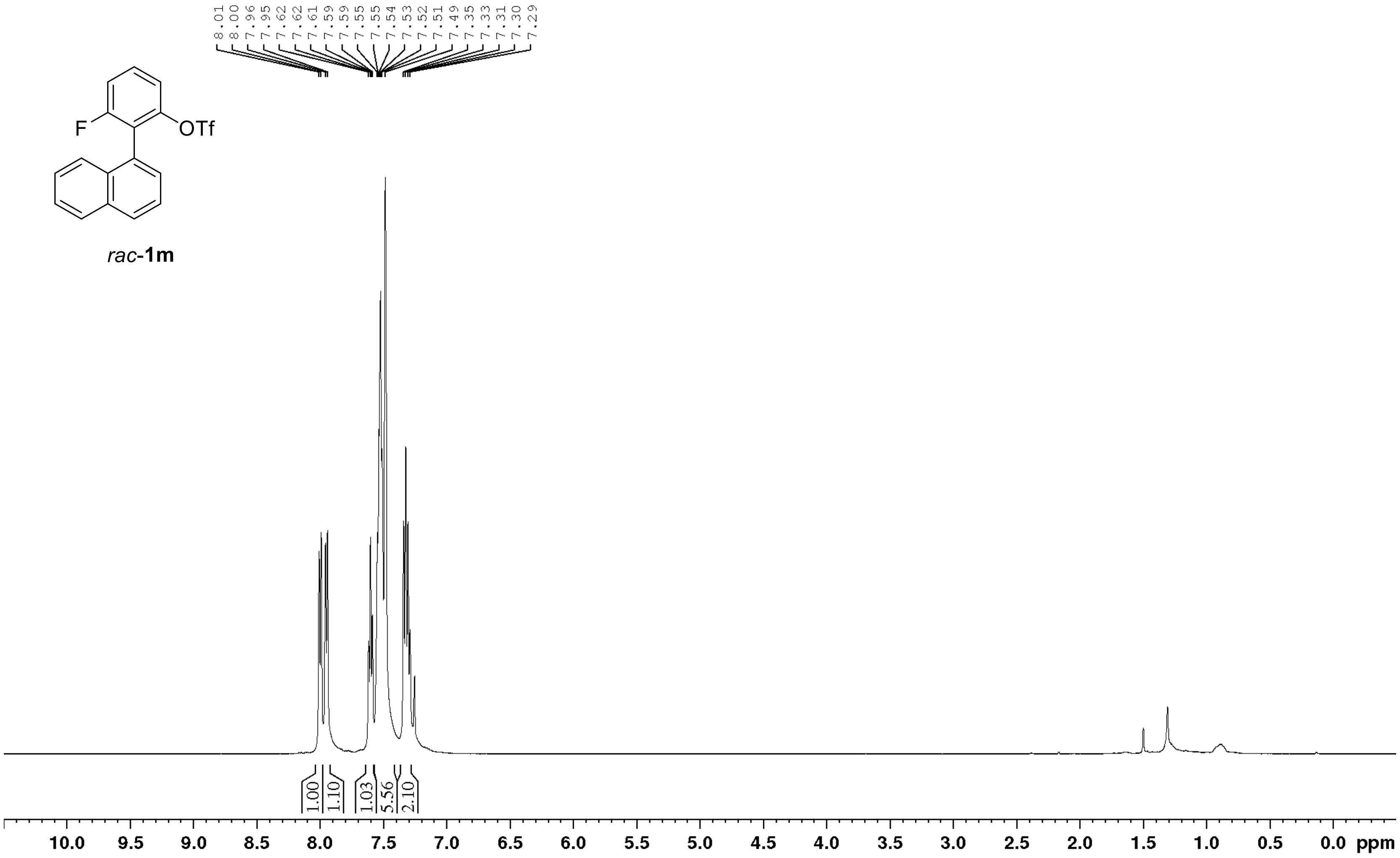

$^{13}C\{^1H\}$ NMR spectrum (126 MHz, $CDCl_3$, 298 K) of 3-fluoro-2-(naphthalen-1-yl)phenyl trifluoromethanesulfonate (*rac*-**1m**)

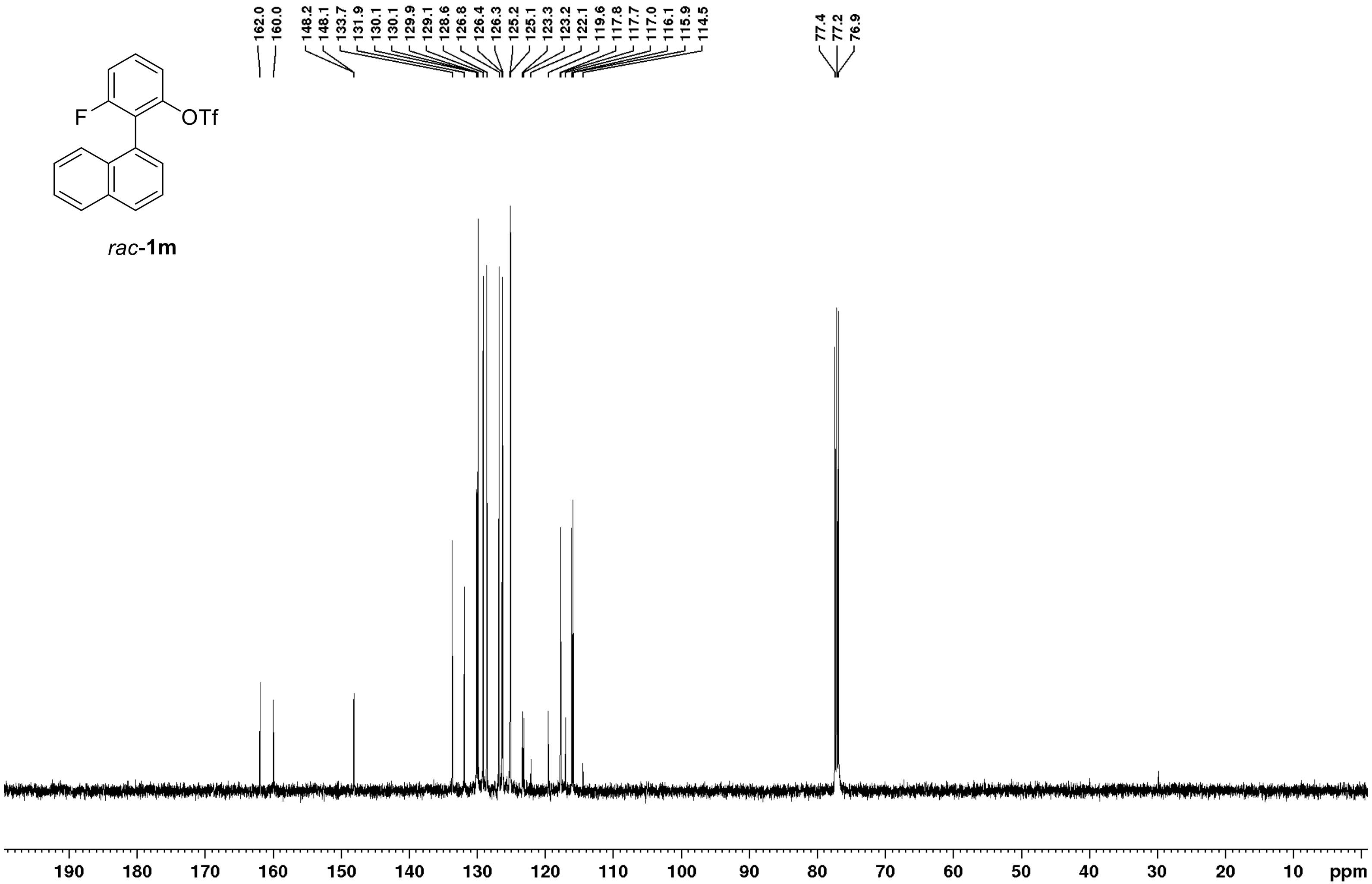

^{19}F NMR spectrum (471 MHz, $CDCl_3$, 298 K) of 3-fluoro-2-(naphthalen-1-yl)phenyl trifluoromethanesulfonate (*rac*-**1m**)

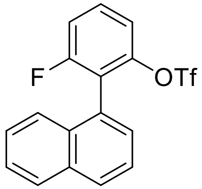

rac-**1m**

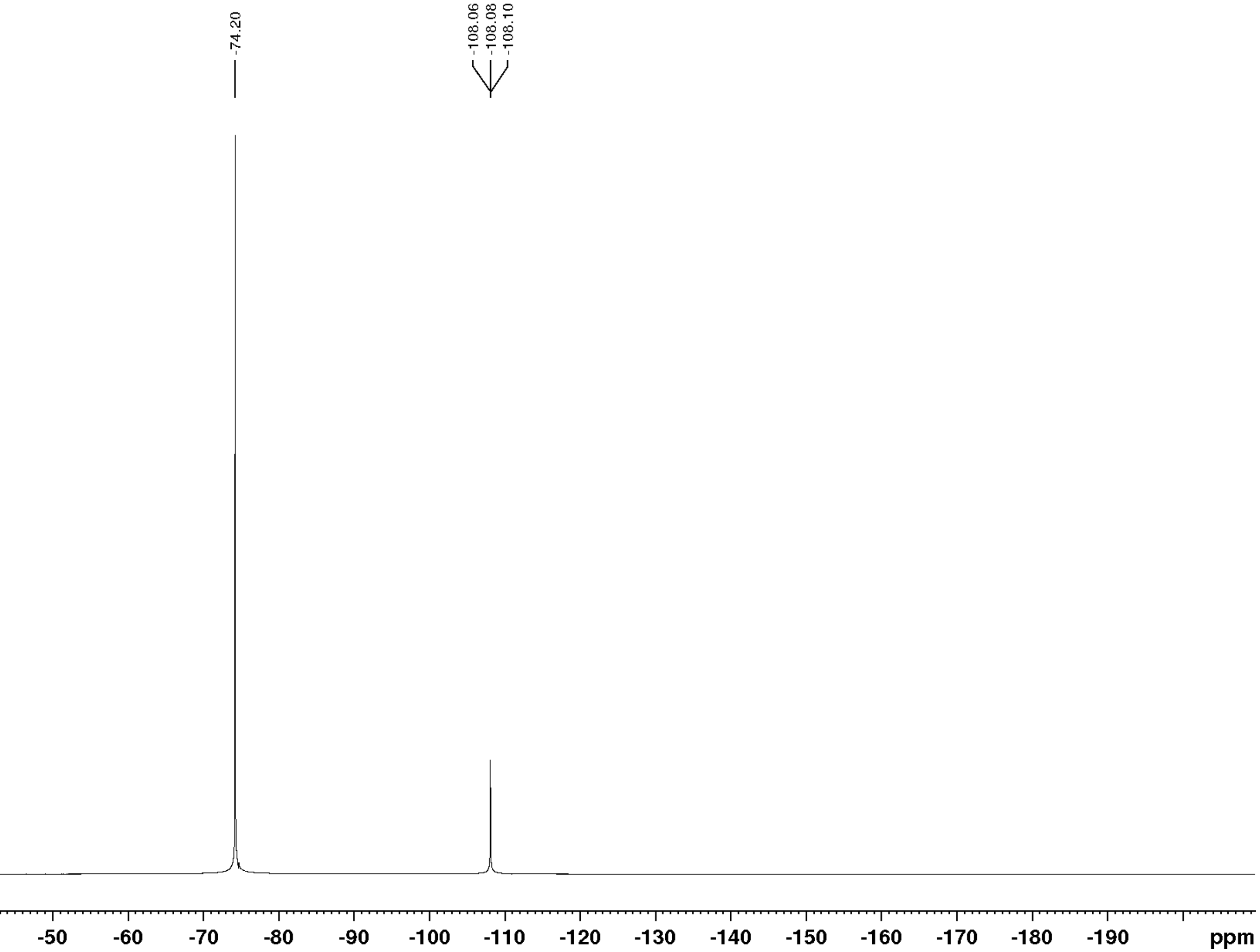

^{1}H NMR spectrum (500 MHz, $CDCl_3$, 298 K) of 3-methoxy-2-(naphthalen-1-yl)phenyl trifluoromethanesulfonate (*rac*-**1n**)

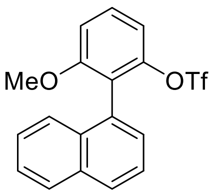

rac-**1n**

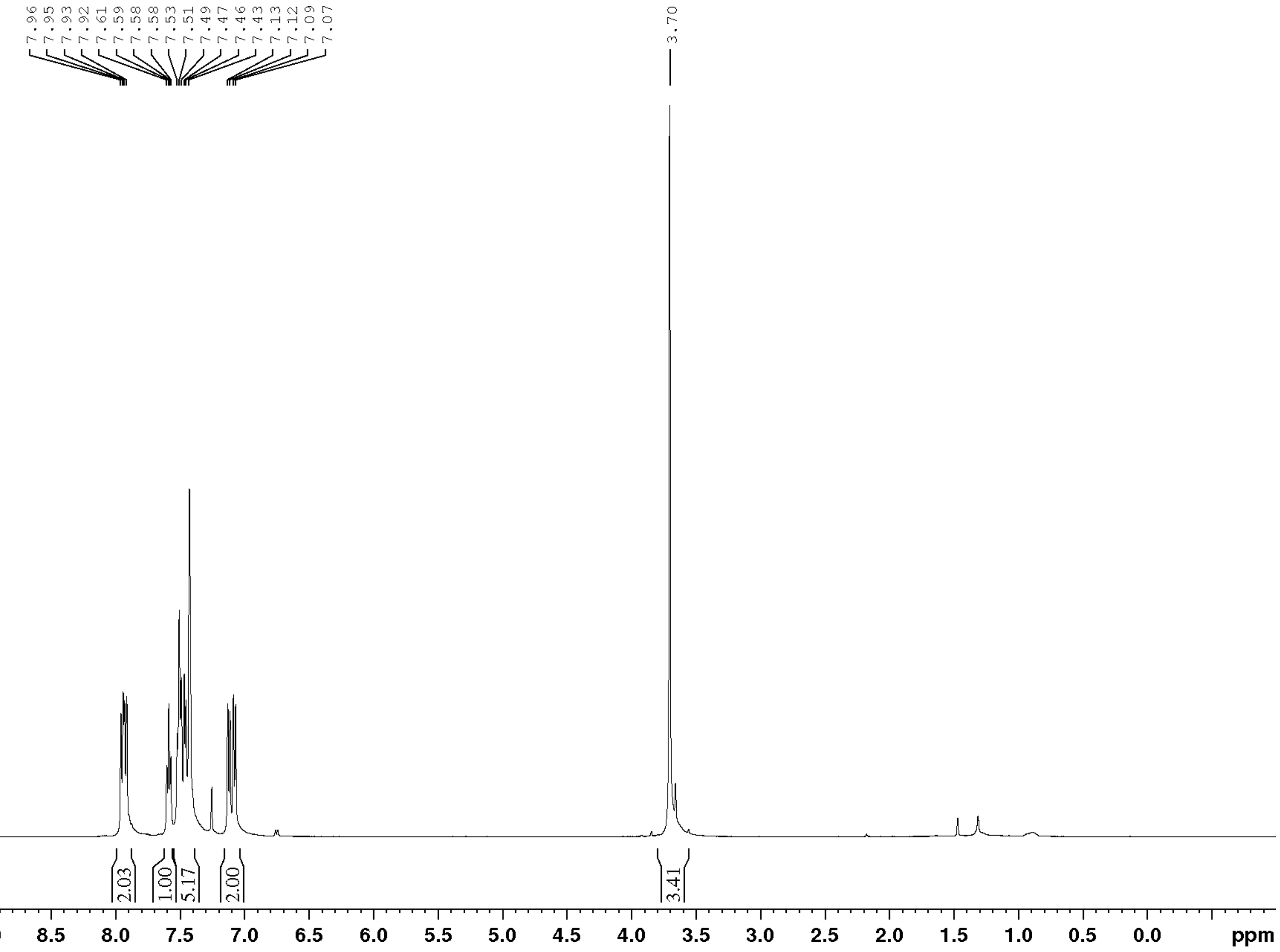

$^{13}C\{^1H\}$ NMR spectrum (126 MHz, $CDCl_3$, 298 K) of 3-methoxy-2-(naphthalen-1-yl)phenyl trifluoromethanesulfonate (*rac*-**1n**)

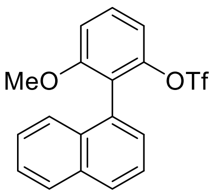

rac-**1n**

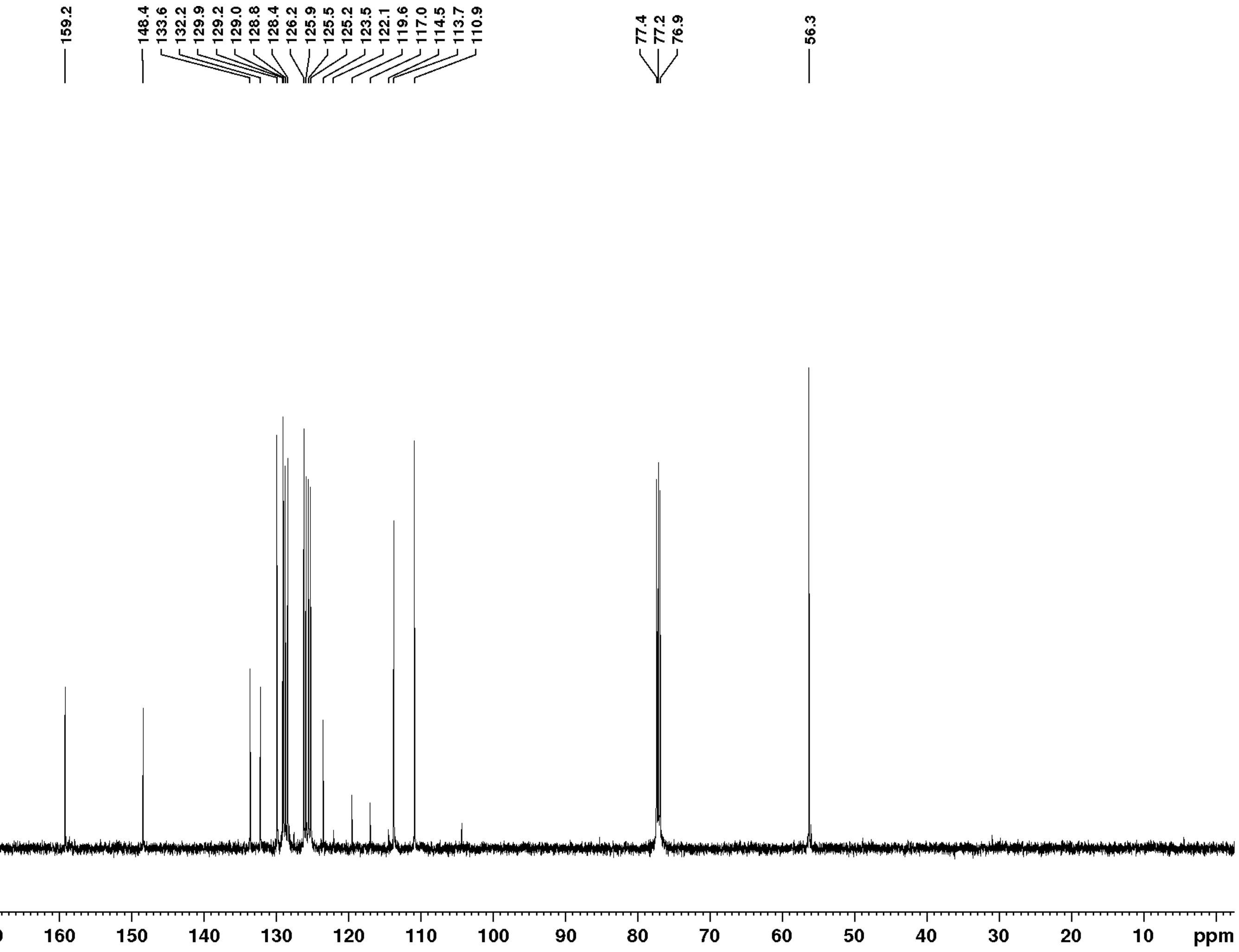

^{19}F NMR spectrum (471 MHz, $CDCl_3$, 298 K) of 3-methoxy-2-(naphthalen-1-yl)phenyl trifluoromethanesulfonate (*rac*-**1n**)

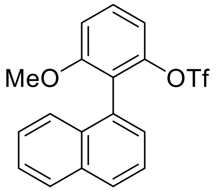

rac-**1n**

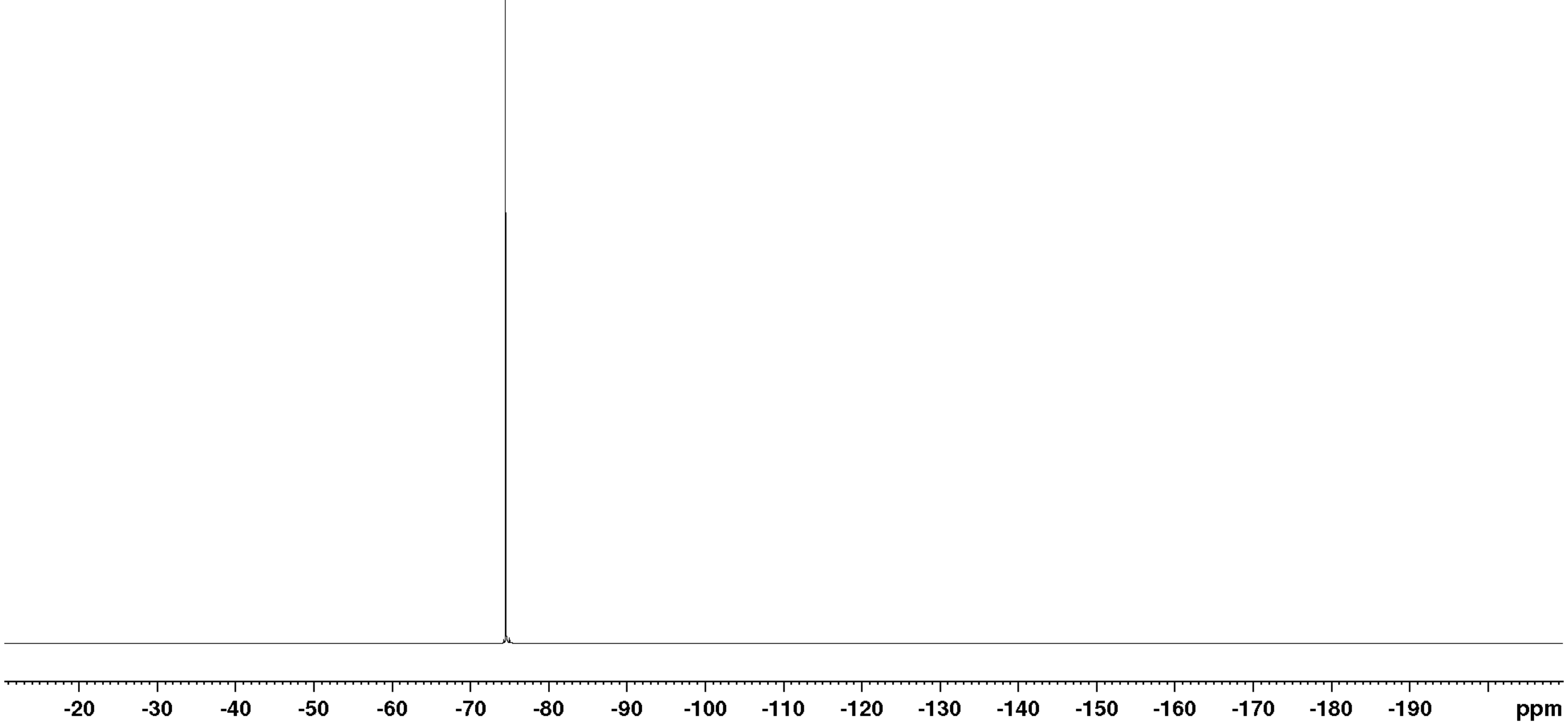

^{1}H NMR spectrum (500 MHz, $CDCl_3$, 298 K) of (*S*)-1-([1,1'-binaphthalen]-2-yl)-2-phenyldiazene ((*S*)-**3aa**)

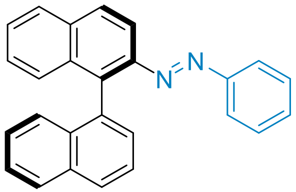

(*S*)-**3aa**

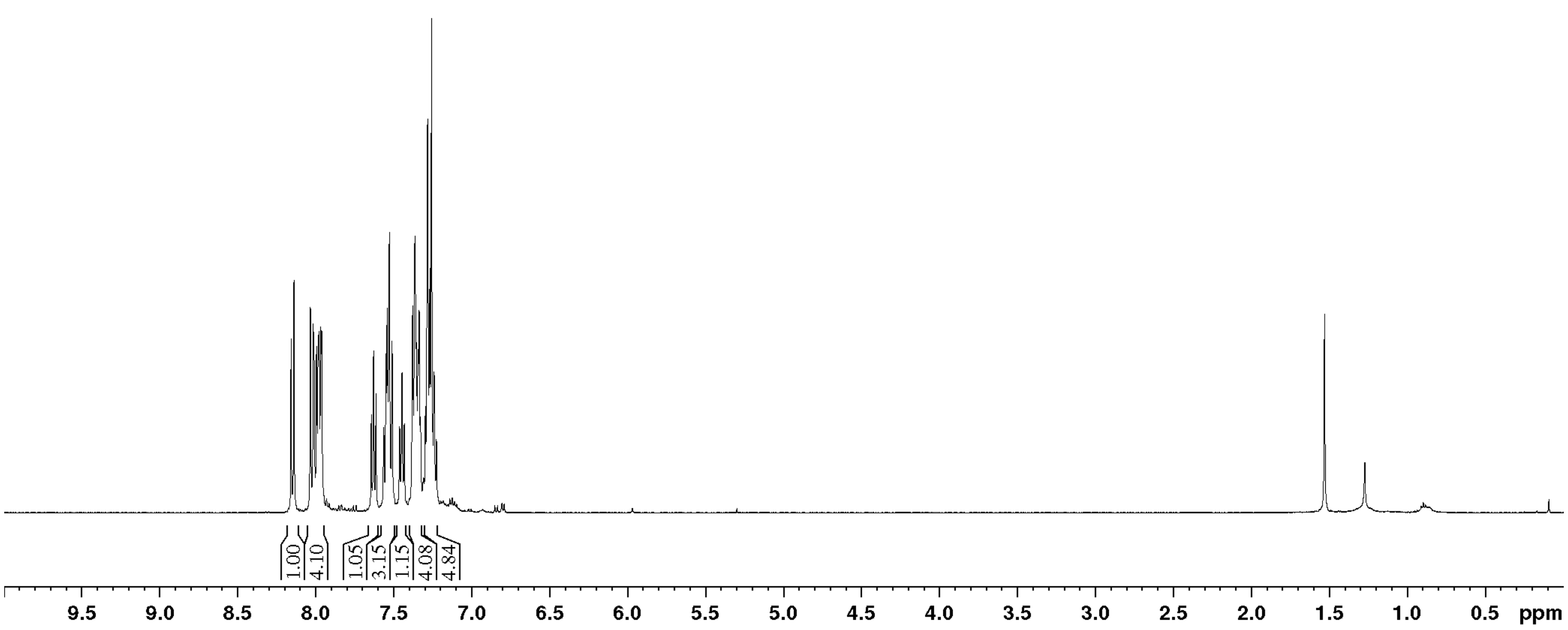

$^{13}C\{^{1}H\}$ NMR spectrum (126 MHz, $CDCl_3$, 298 K) of (*S*)-1-([1,1'-binaphthalen]-2-yl)-2-phenyldiazene ((*S*)-**3aa**)

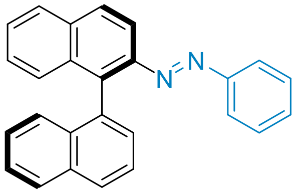

(*S*)-**3aa**

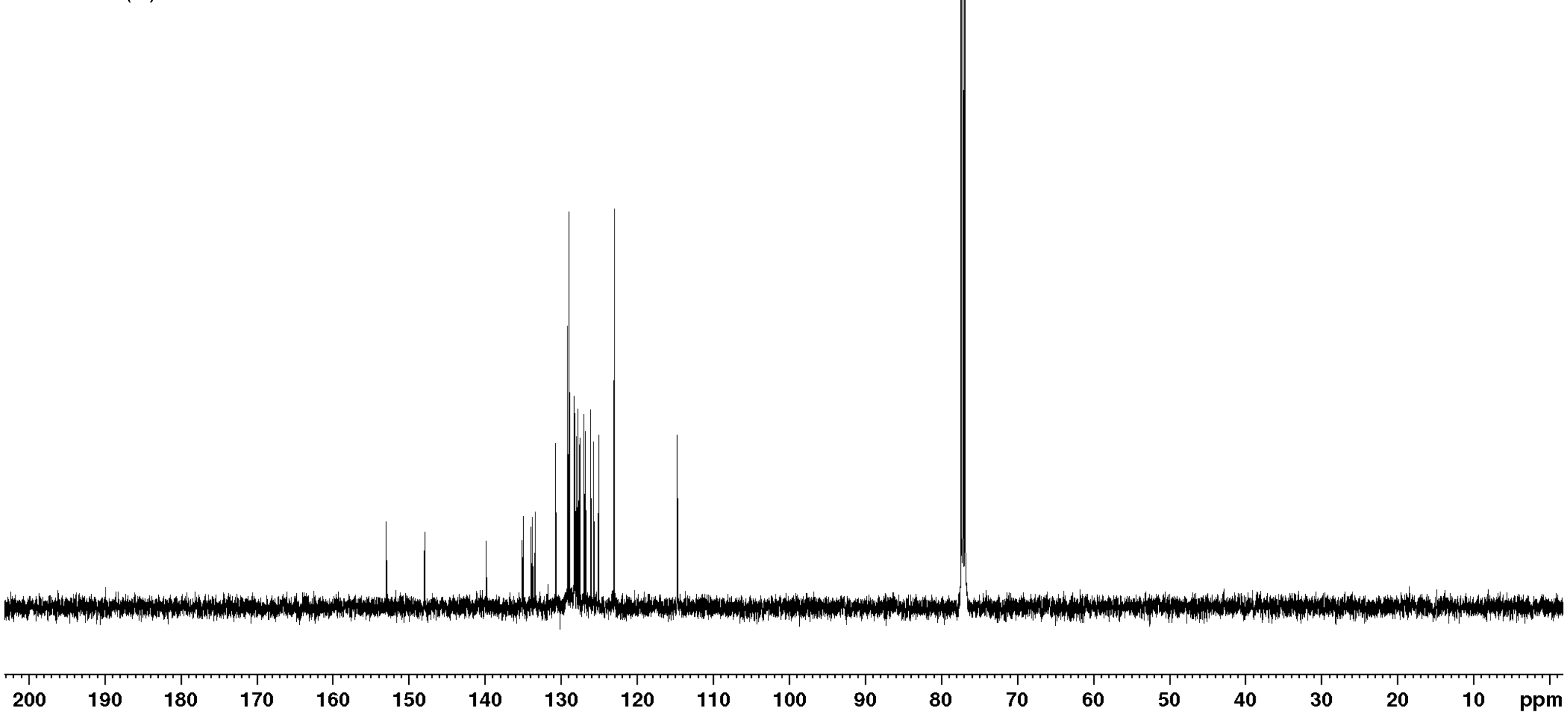

^{1}H NMR spectrum (500 MHz, $CDCl_3$, 298 K) of (*S*)-1-([1,1'-binaphthalen]-2-yl)-2-(4-fluorophenyl)diazene ((*S*)-**3ab**)

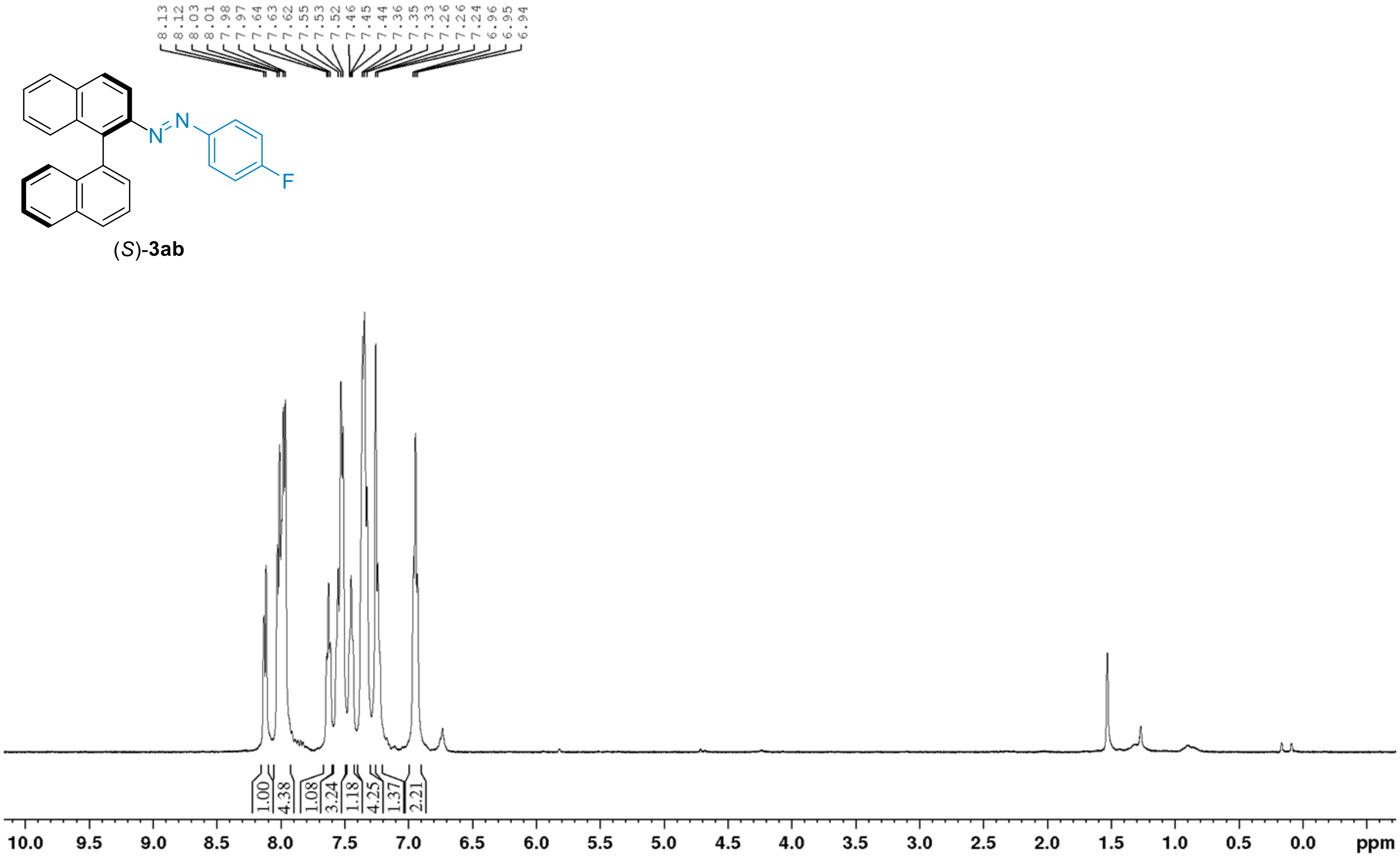

$^{13}C\{^1H\}$ NMR spectrum (126 MHz, $CDCl_3$, 298 K) of (*S*)-1-([1,1'-binaphthalen]-2-yl)-2-(4-fluorophenyl)diazene ((*S*)-**3ab**)

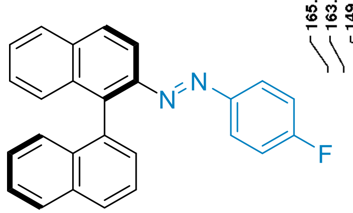

(*S*)-**3ab**

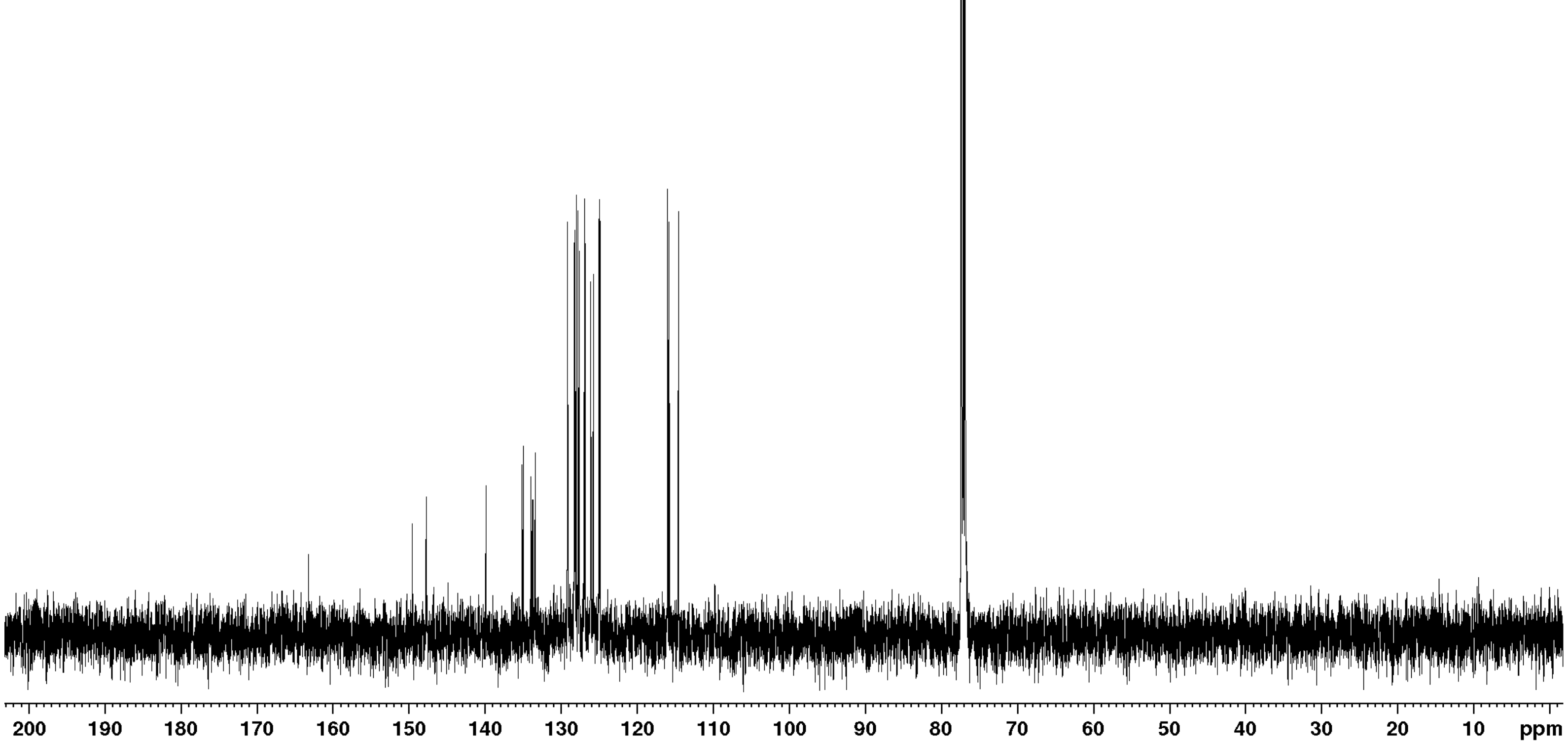

^{19}F NMR spectrum (471 MHz, $CDCl_3$, 298 K) of (*S*)-1-([1,1'-binaphthalen]-2-yl)-2-(4-fluorophenyl)diazene ((*S*)-**3ab**)

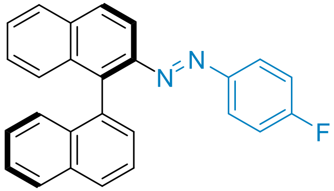

(*S*)-**3ab**

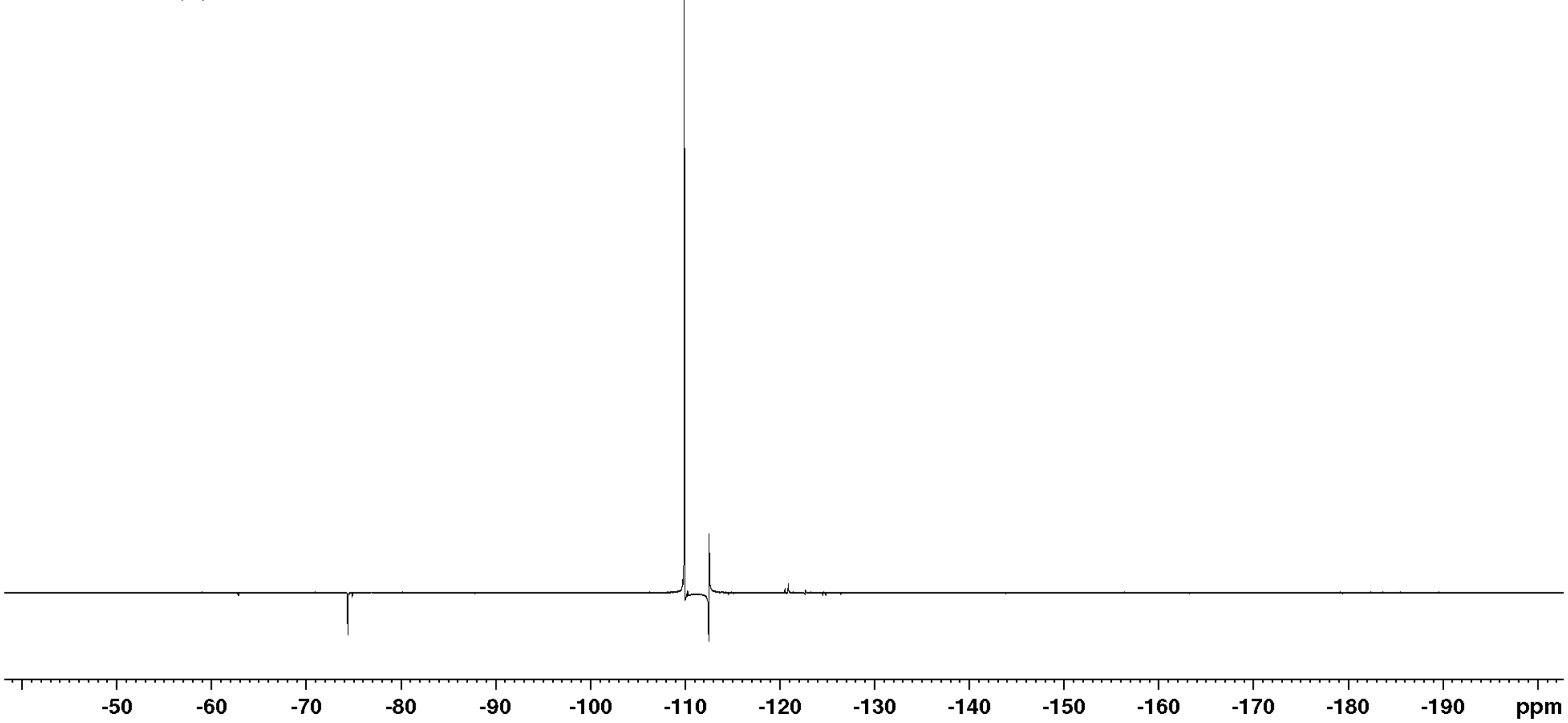

^{1}H NMR spectrum (500 MHz, $CDCl_3$, 298 K) of (*S*)-1-([1,1'-binaphthalen]-2-yl)-2-(4-chlorophenyl)diazene ((*S*)-**3ac**)

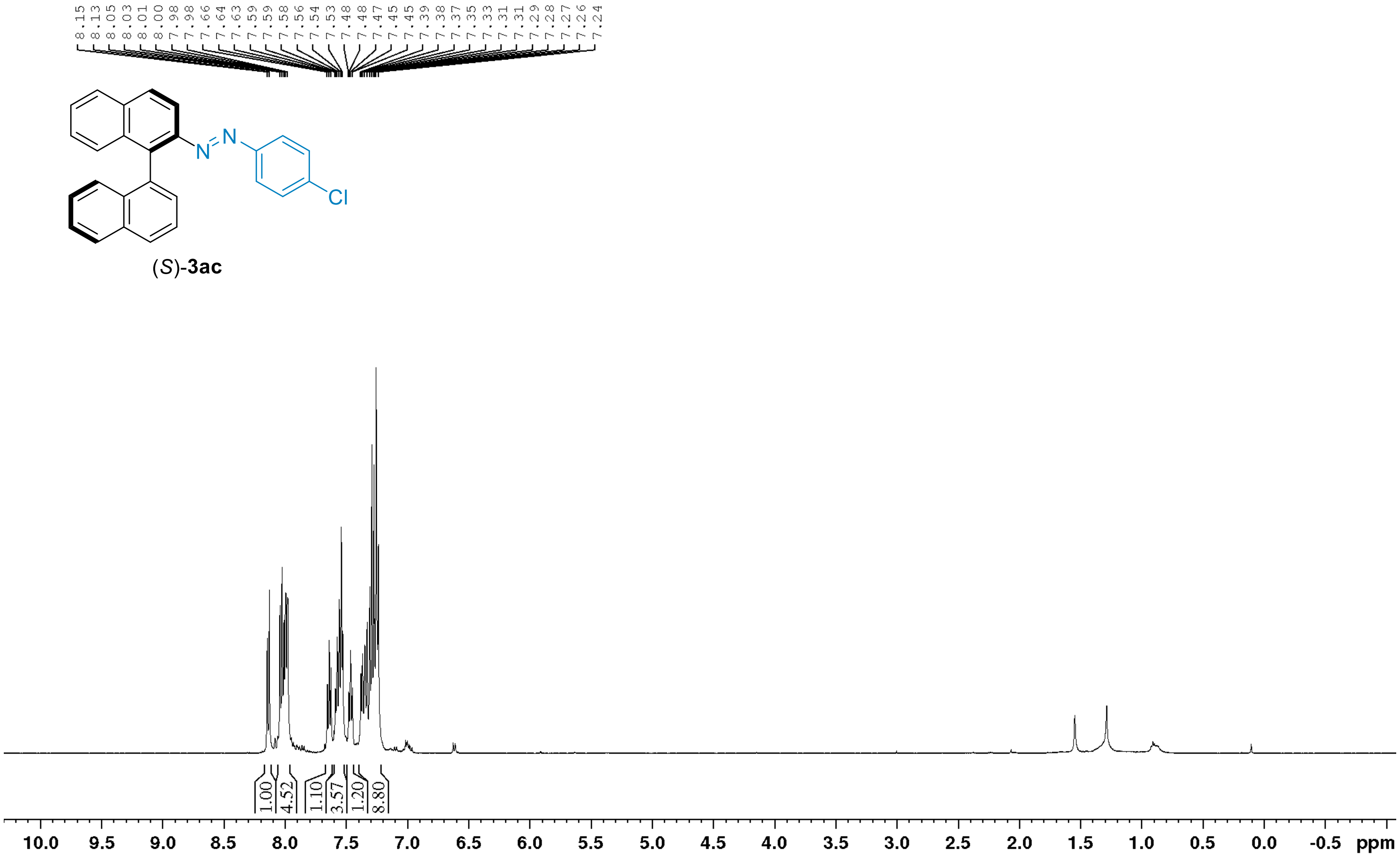

$^{13}C\{^1H\}$ NMR spectrum (126 MHz, $CDCl_3$, 298 K) of (*S*)-1-([1,1'-binaphthalen]-2-yl)-2-(4-chlorophenyl)diazene ((*S*)-**3ac**)

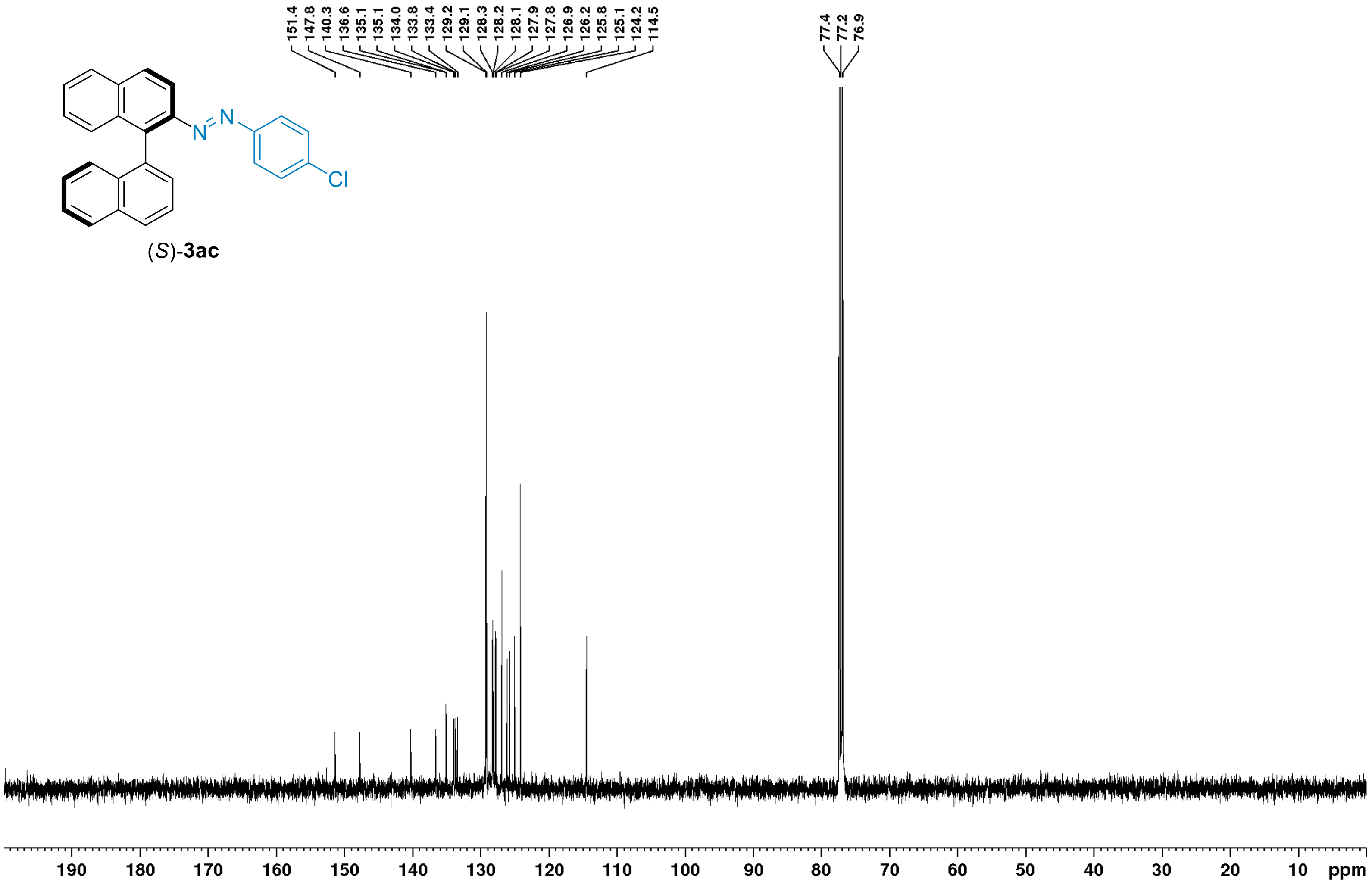

^{1}H NMR spectrum (500 MHz, $CDCl_3$, 298 K) of (*S*)-1-([1,1'-binaphthalen]-2-yl)-2-(3-fluorophenyl)diazene ((*S*)-**3ad**)

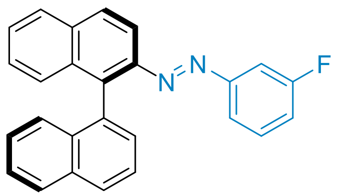

(*S*)-**3ad**

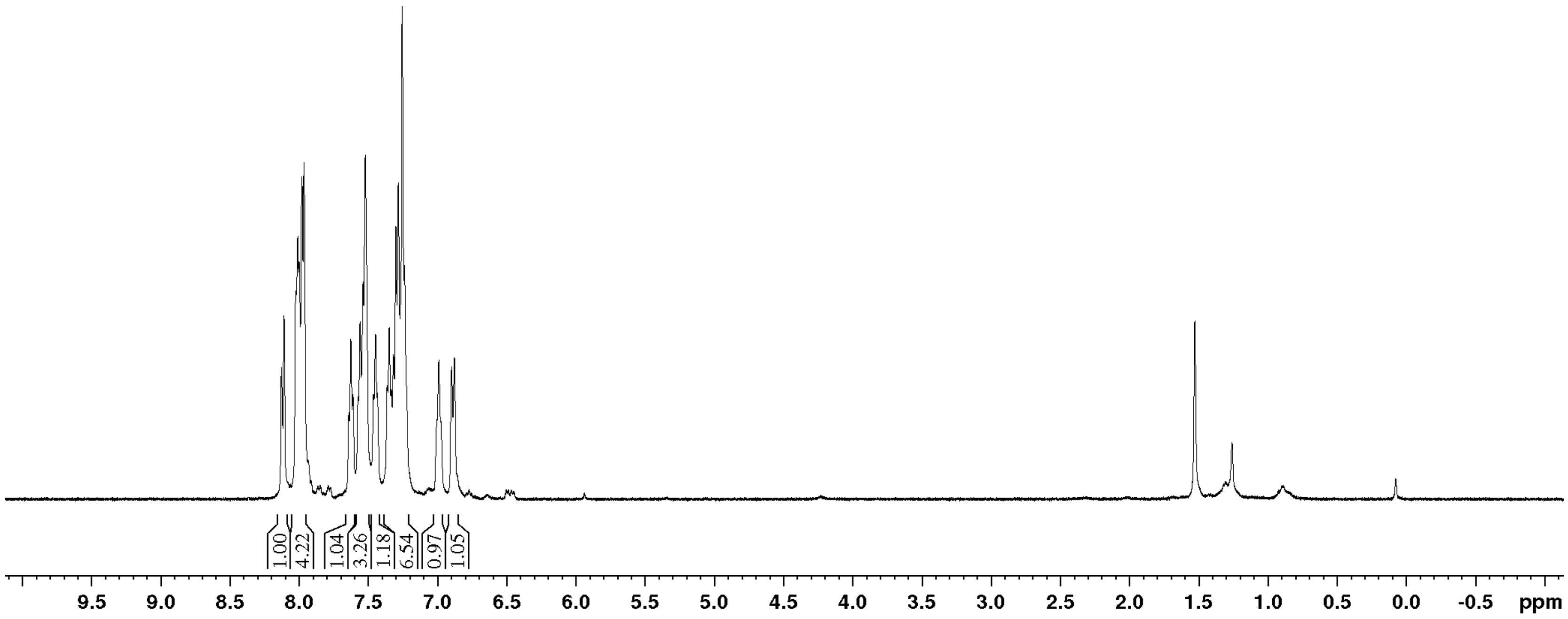

$^{13}C\{^1H\}$ NMR spectrum (101 MHz, $CDCl_3$, 298 K) of (*S*)-1-([1,1'-binaphthalen]-2-yl)-2-(3-fluorophenyl)diazene ((*S*)-**3ad**)

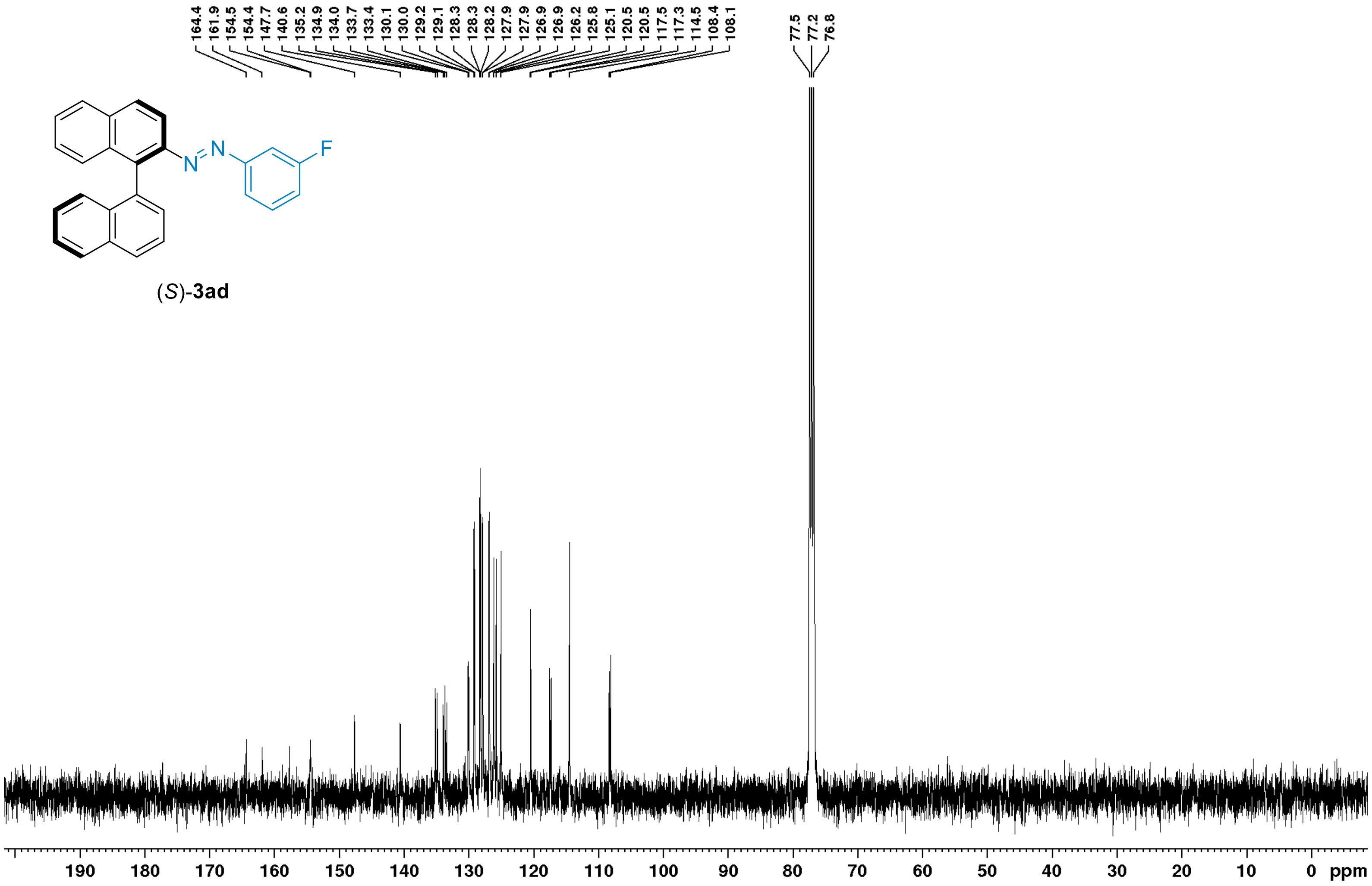

^{19}F NMR spectrum (471 MHz, $CDCl_3$, 298 K) of (*S*)-1-([1,1'-binaphthalen]-2-yl)-2-(3-fluorophenyl)diazene ((*S*)-**3ad**)

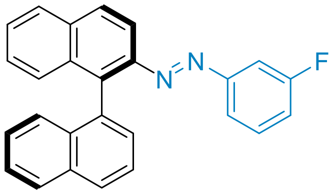

(*S*)-**3ad**

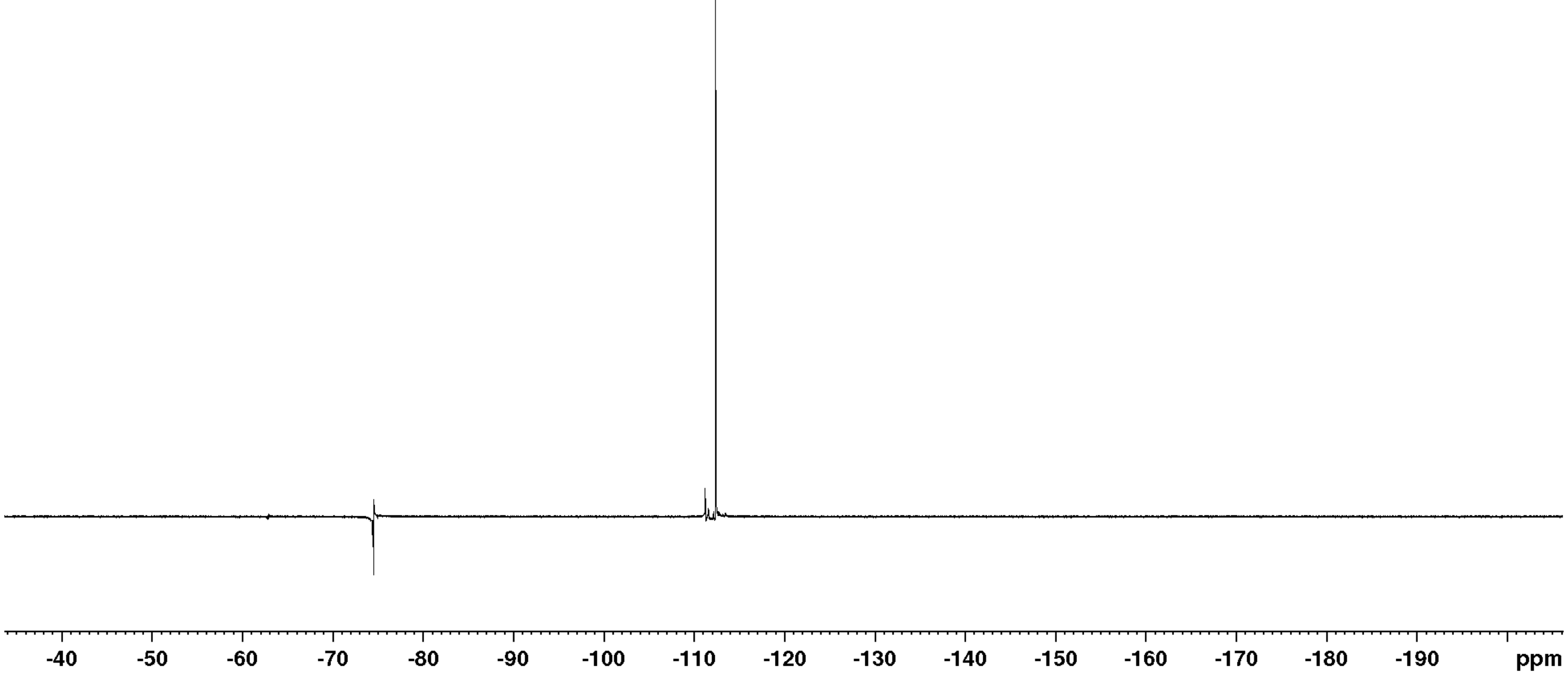

^{1}H NMR spectrum (500 MHz, $CDCl_3$, 298 K) of (*S*)-1-([1,1'-binaphthalen]-2-yl)-2-(p-tolyl)diazene ((*S*)-**3ae**)

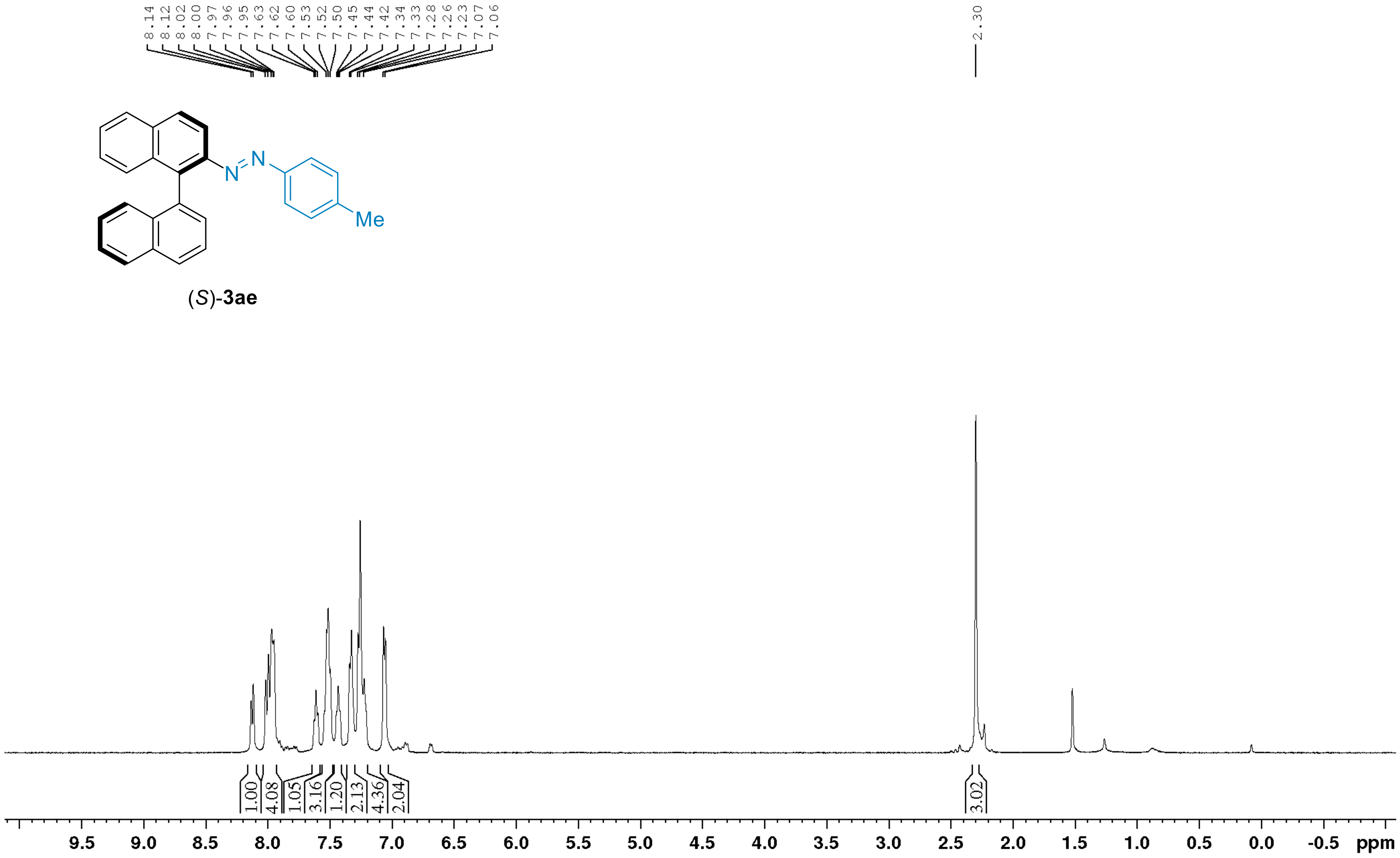

$^{13}C\{^1H\}$ NMR spectrum (126 MHz, $CDCl_3$, 298 K) of (*S*)-1-([1,1'-binaphthalen]-2-yl)-2-(p-tolyl)diazene ((*S*)-**3ae**)

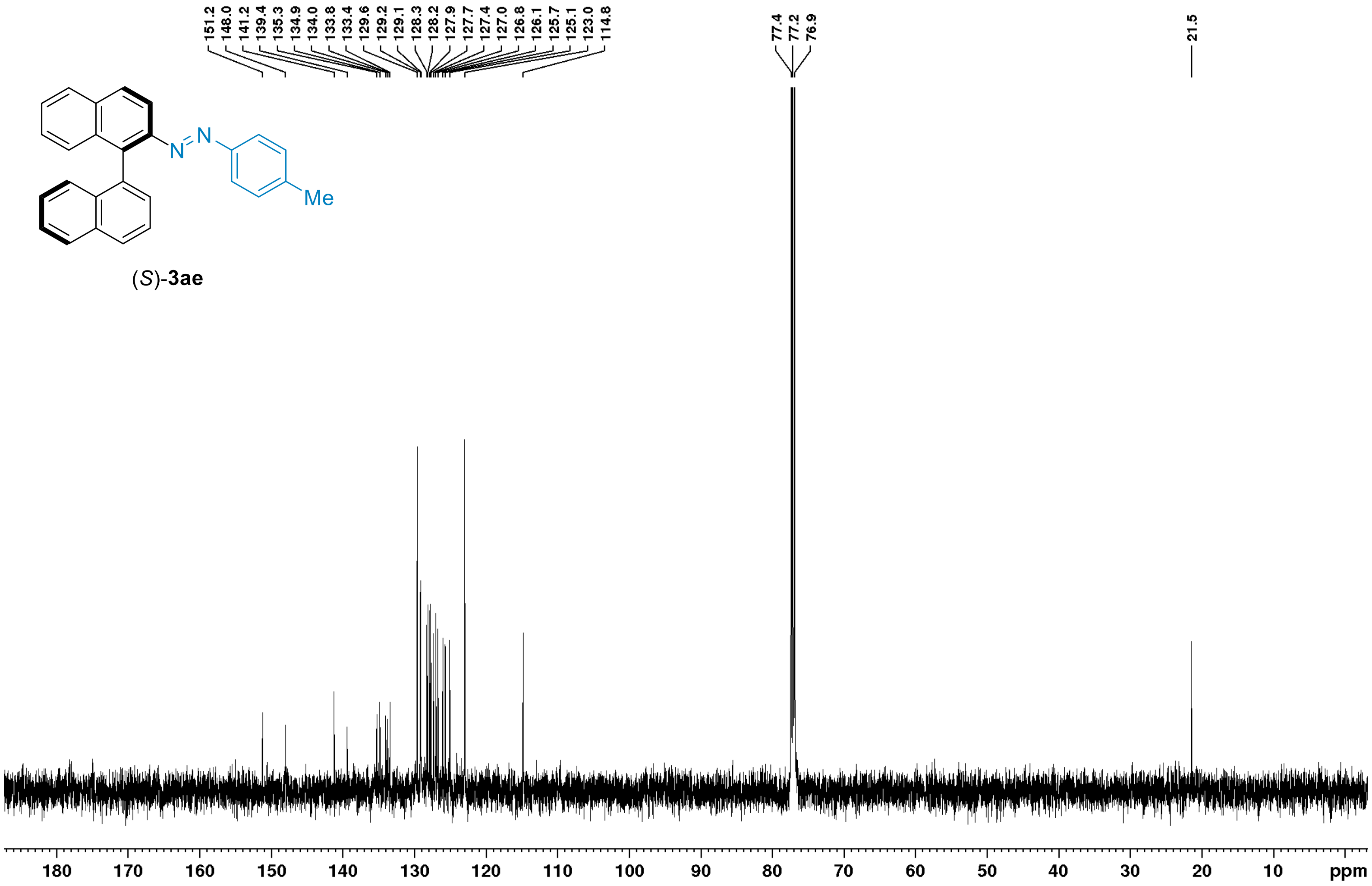

^{1}H NMR spectrum (500 MHz, $CDCl_3$, 298 K) of (*S*)-1-([1,1'-binaphthalen]-2-yl)-2-(4-methoxyphenyl)diazene ((*S*)-**3af**)

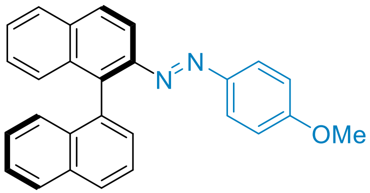

(*S*)-**3af**

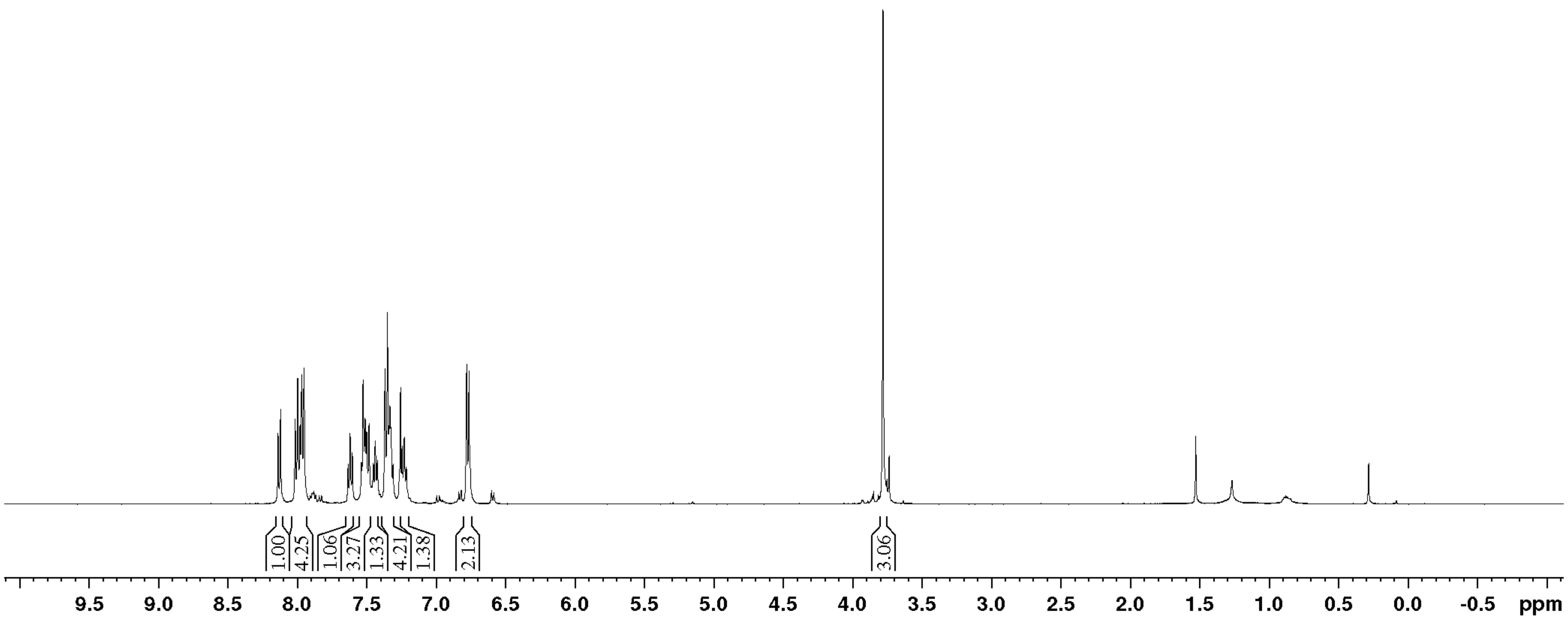

$^{13}C\{^{1}H\}$ NMR spectrum (126 MHz, $CDCl_3$, 298 K) of (*S*)-1-([1,1'-binaphthalen]-2-yl)-2-(4-methoxyphenyl)diazene ((*S*)-**3af**)

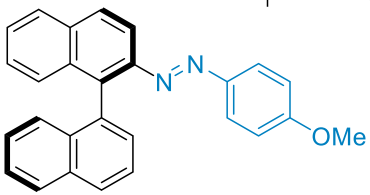

(*S*)-**3af**

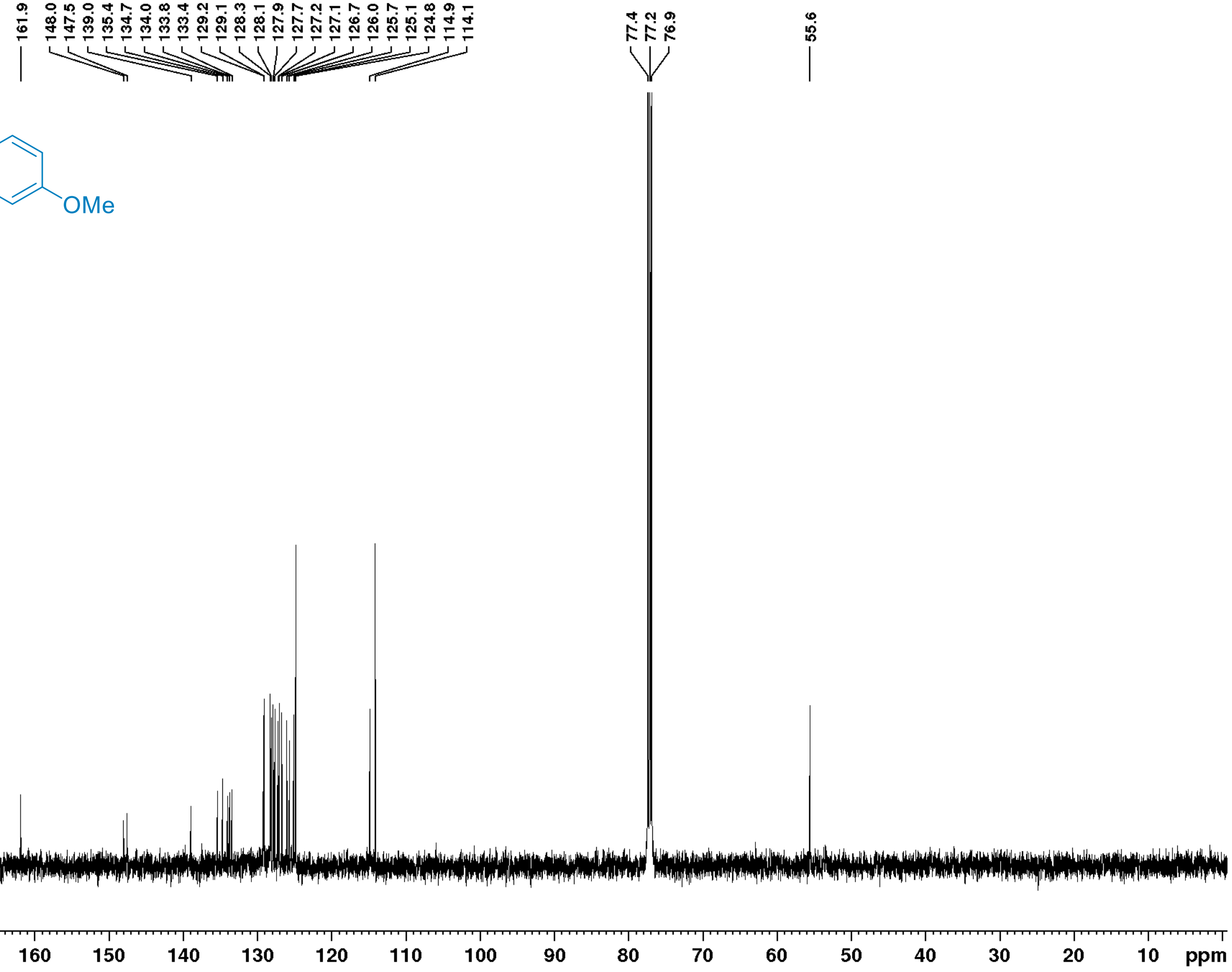

^{1}H NMR spectrum (500 MHz, $CDCl_3$, 298 K) of Methyl-(*S*)-4-([1,1'-binaphthalen]-2-yldiazenyl)benzoate ((*S*)-**3ag**)

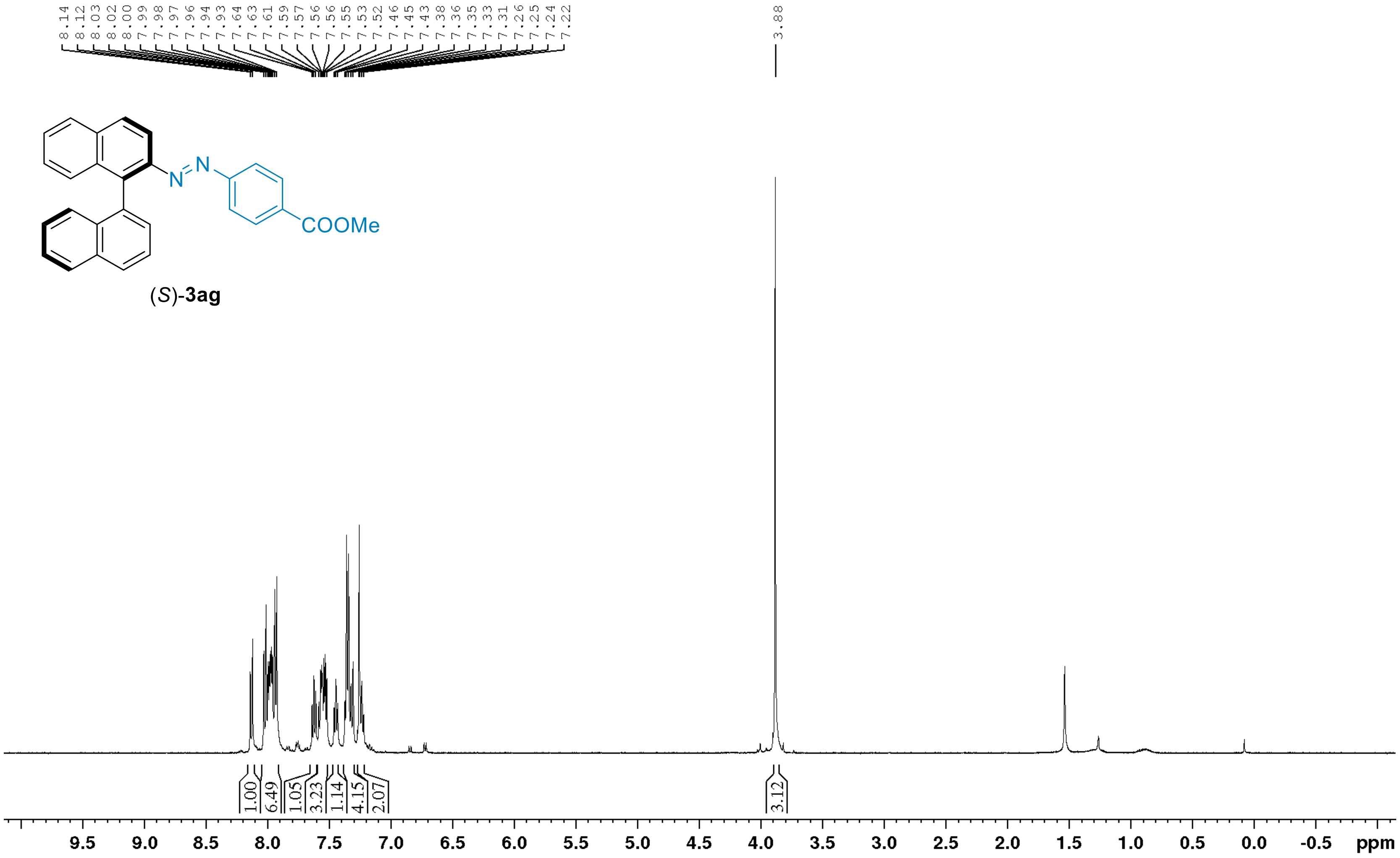

$^{13}C\{^{1}H\}$ NMR spectrum (126 MHz, $CDCl_3$, 298 K) of Methyl-(*S*)-4-([1,1'-binaphthalen]-2-yldiazenyl)benzoate ((*S*)-**3ag**)

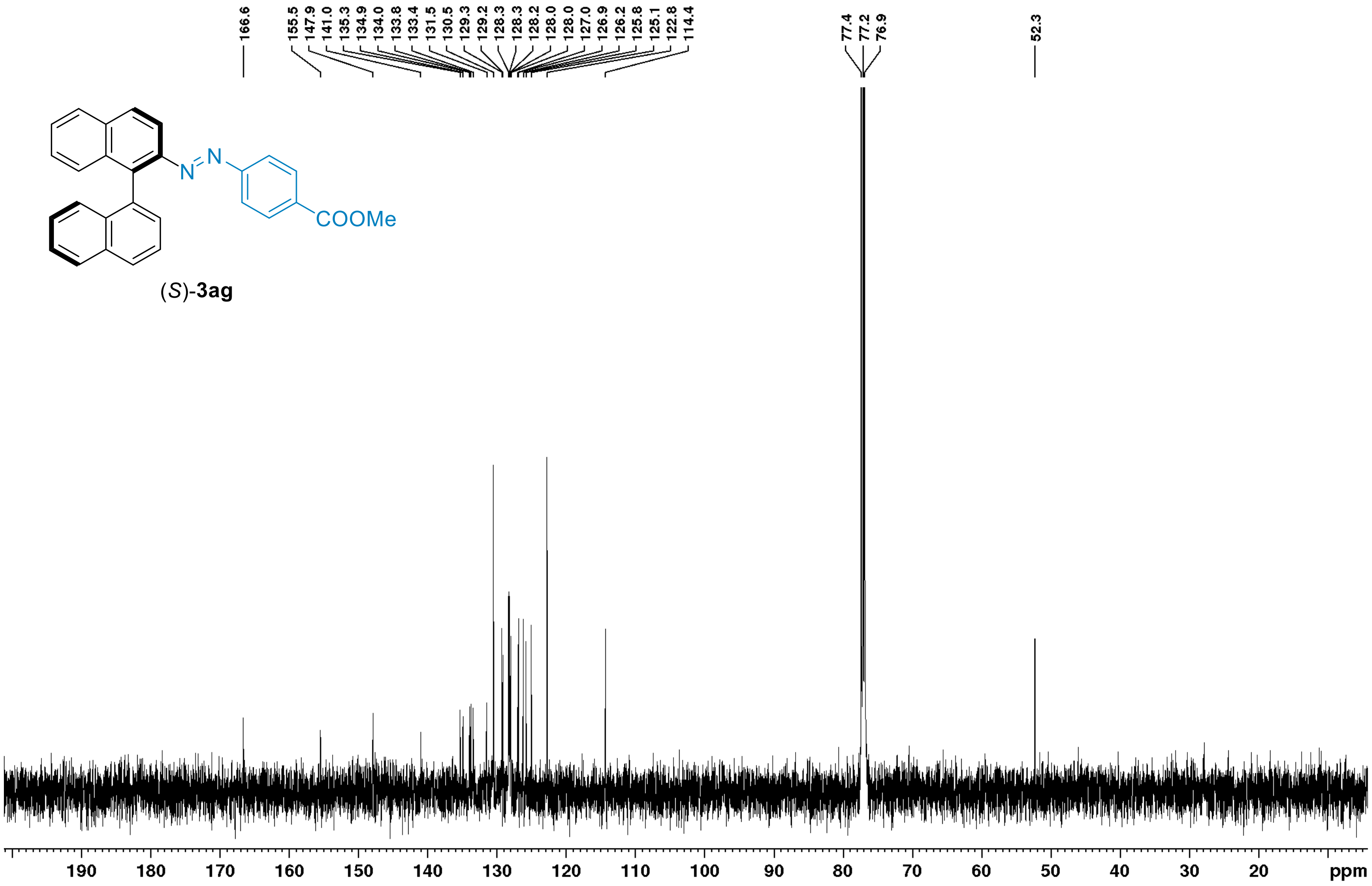

^{1}H NMR spectrum (500 MHz, $CDCl_3$, 298 K) of (*S*)-1-(4-fluorophenyl)-2-(6-methoxy-[1,1'-binaphthalen]-2-yl)diazene ((*S*)-**3bb**)

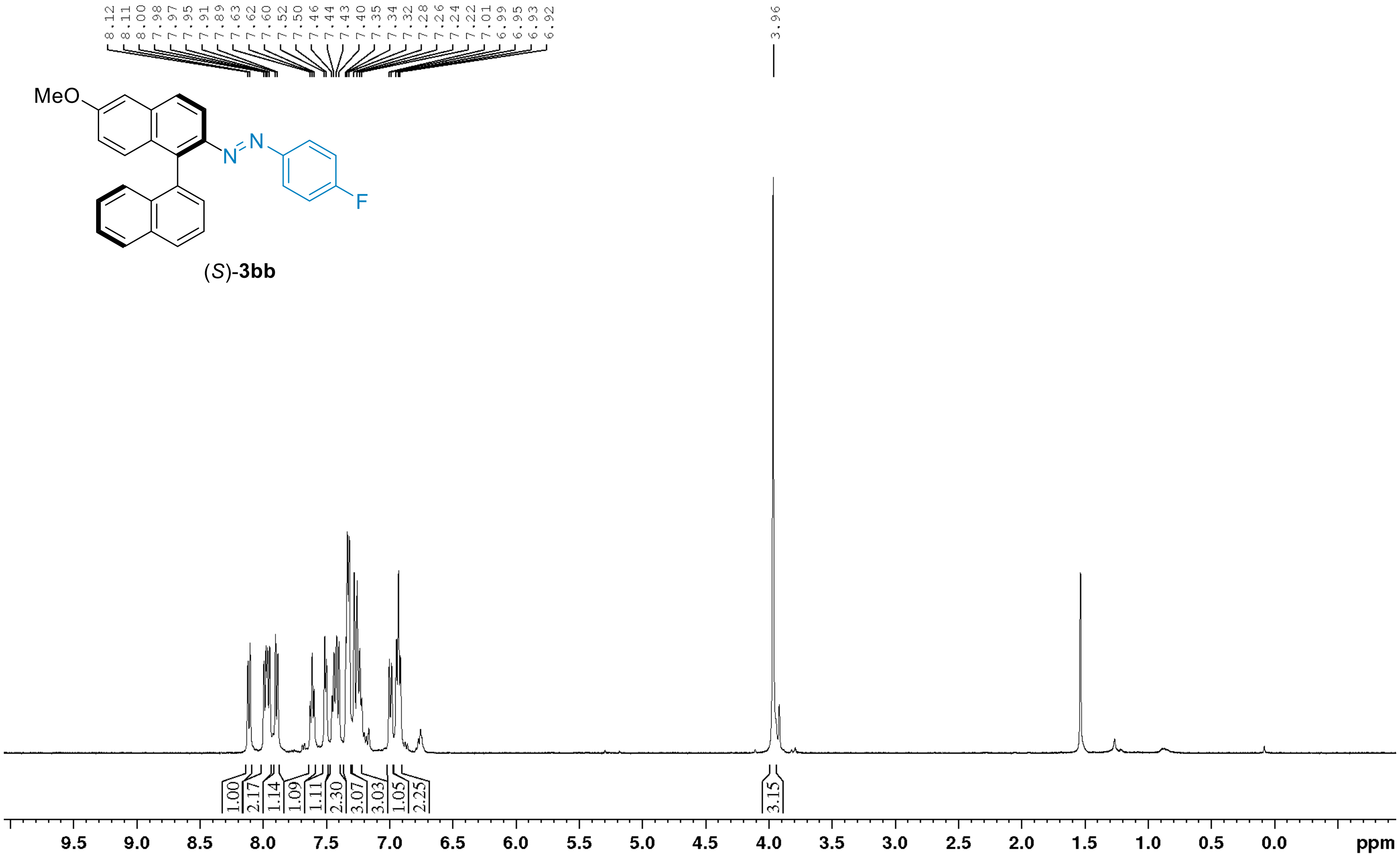

$^{13}C\{^{1}H\}$ NMR spectrum (126 MHz, $CDCl_3$, 298 K) of (*S*)-1-(4-fluorophenyl)-2-(6-methoxy-[1,1'-binaphthalen]-2-yl)diazene ((*S*)-**3bb**)

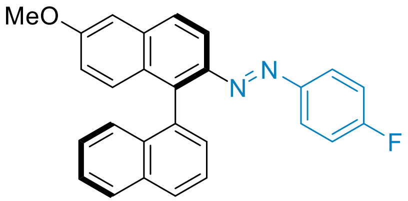

(*S*)-**3bb**

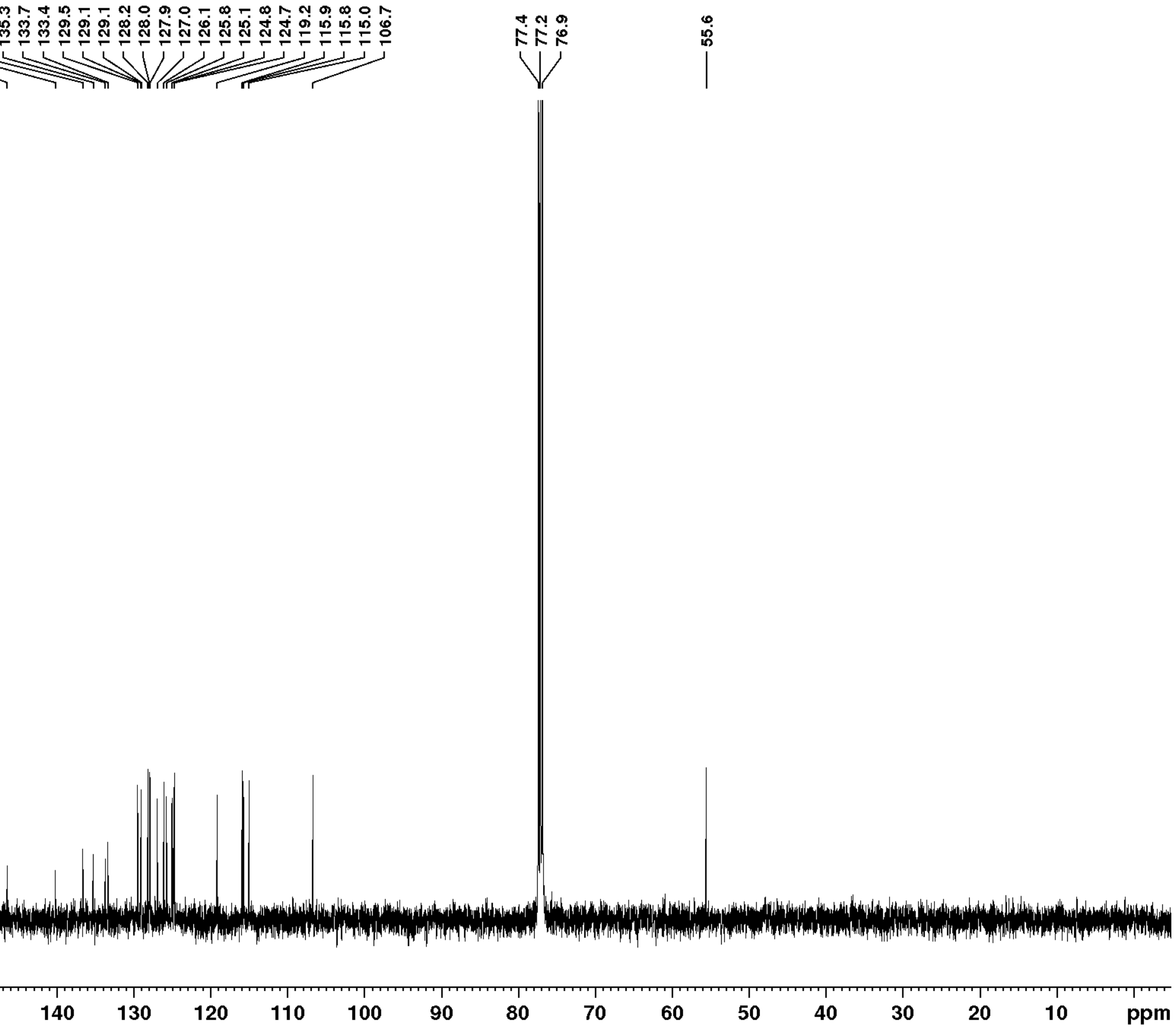

^{19}F NMR spectrum (500 MHz, $CDCl_3$, 298 K) of (*S*)-1-(4-fluorophenyl)-2-(6-methoxy-[1,1'-binaphthalen]-2-yl)diazene ((*S*)-**3bb**)

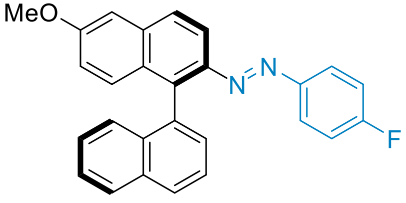

(*S*)-**3bb**

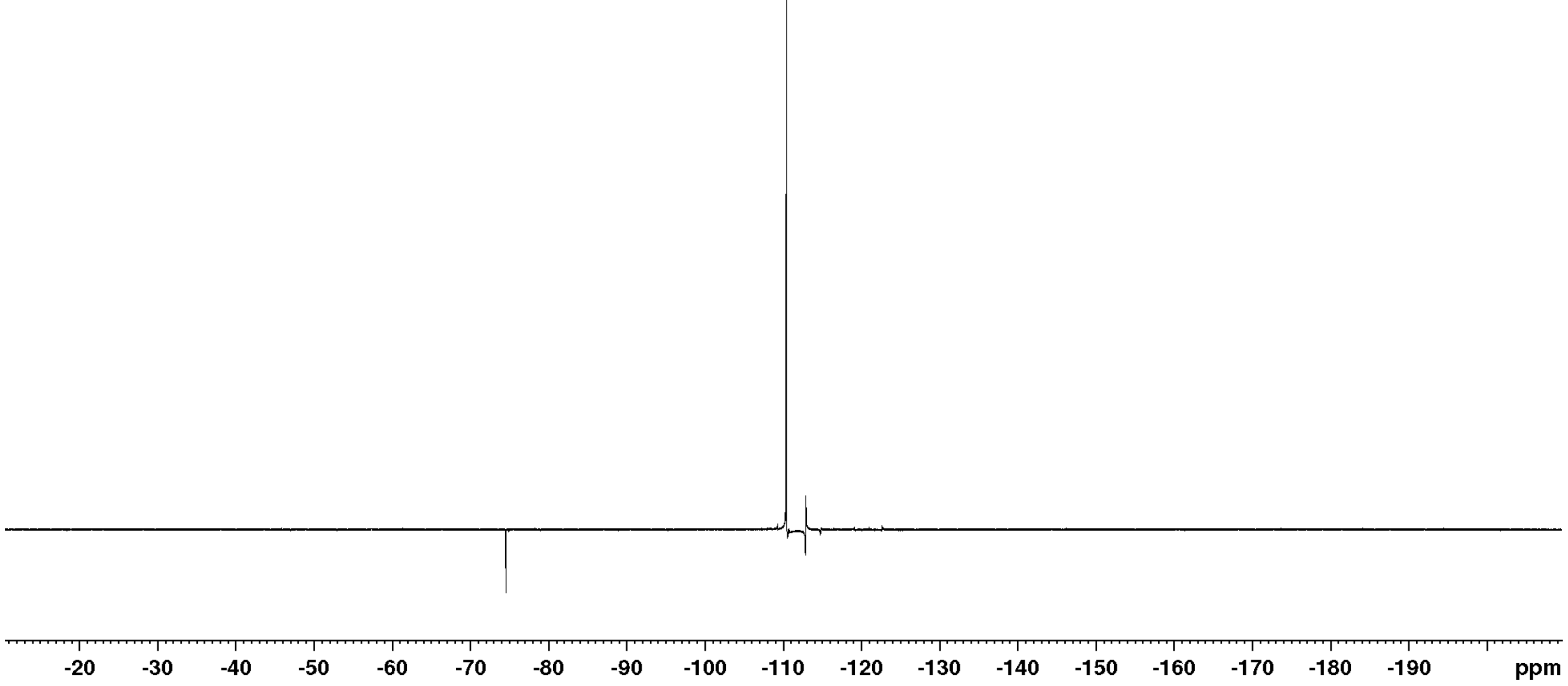

^{1}H NMR spectrum (500 MHz, $CDCl_3$, 298 K) of (*S*)-1-phenyl-2-(6-(trimethylsilyl)-[1,1'-binaphthalen]-2-yl)diazene ((*S*)-**3ca**)

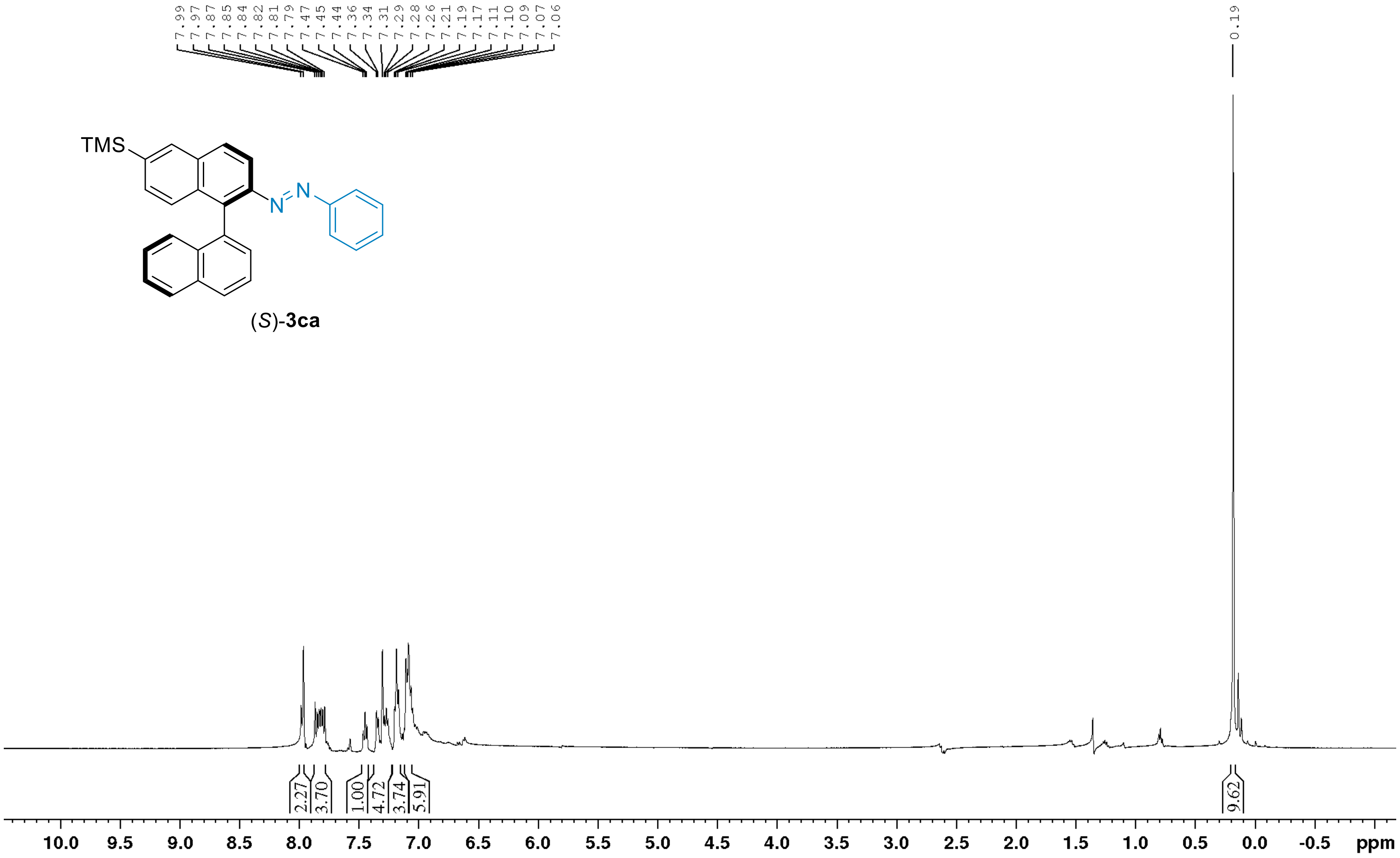

(*S*)-**3ca**

$^{13}C\{^{1}H\}$ NMR spectrum (126 MHz, $CDCl_3$, 298 K) of (*S*)-1-phenyl-2-(6-(trimethylsilyl)-[1,1'-binaphthalen]-2-yl)diazene

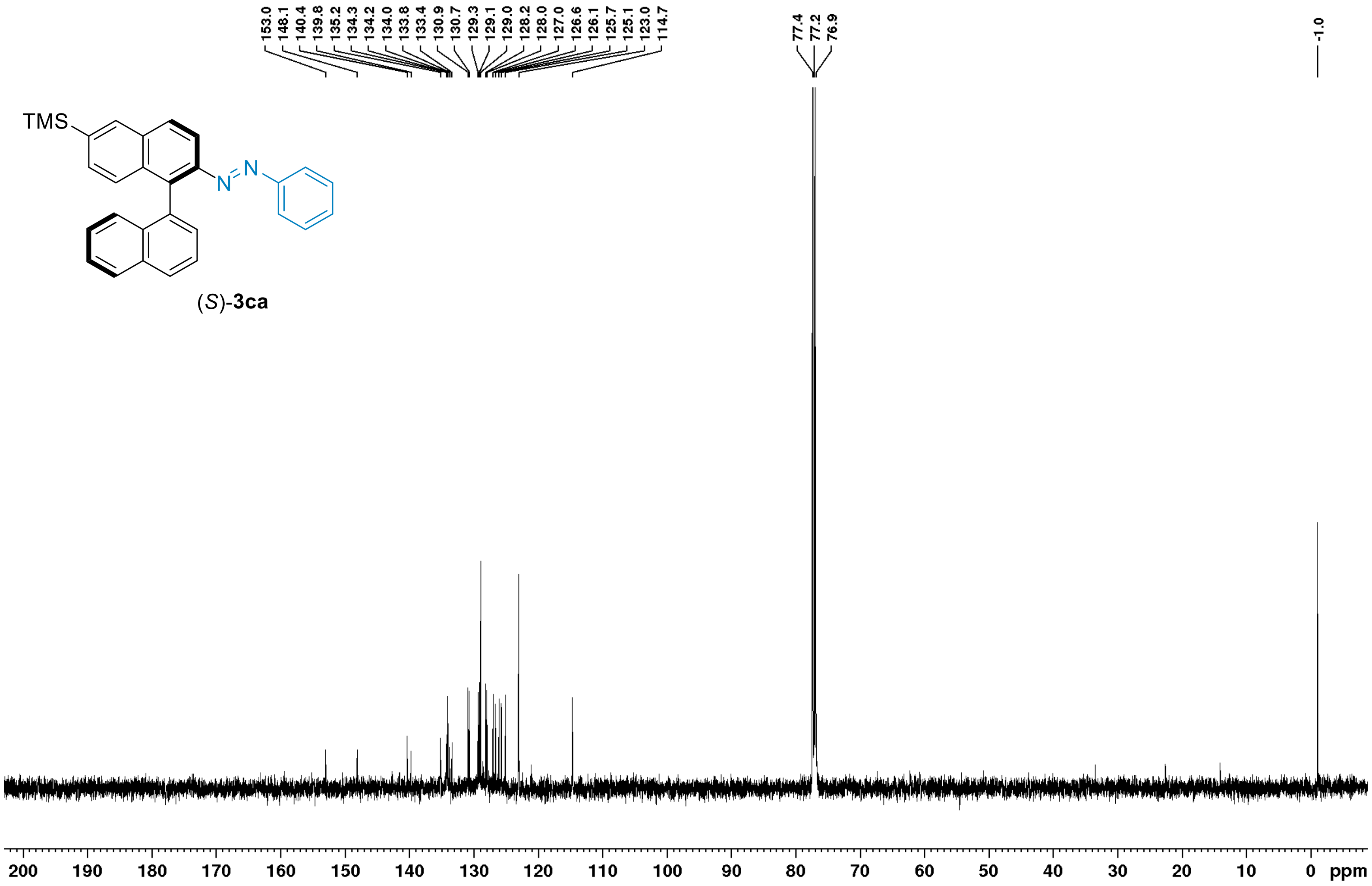

^{1}H NMR spectrum (500 MHz, $CDCl_3$, 298 K) of (*S*)-1-(3-methyl-[1,1'-binaphthalen]-2-yl)-2-phenyldiazene ((*S*)-**3da**)

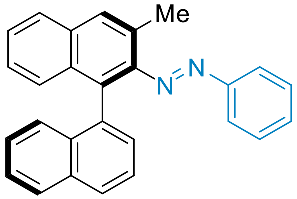

(*S*)-**3da**

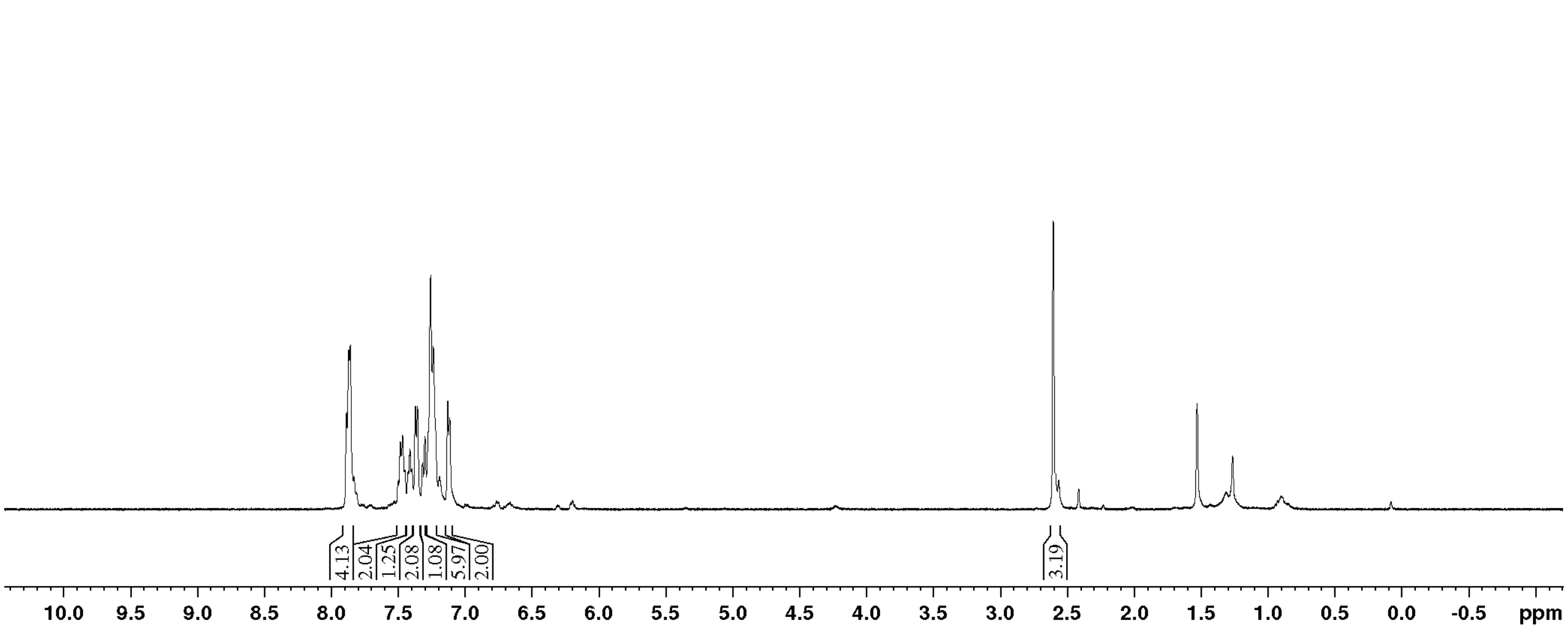

$^{13}C\{^{1}H\}$ NMR spectrum (101 MHz, $CDCl_3$, 298 K) of (*S*)-1-(3-methyl-[1,1'-binaphthalen]-2-yl)-2-phenyldiazene ((*S*)-**3da**)

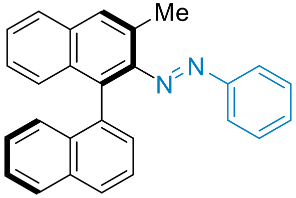

(*S*)-**3da**

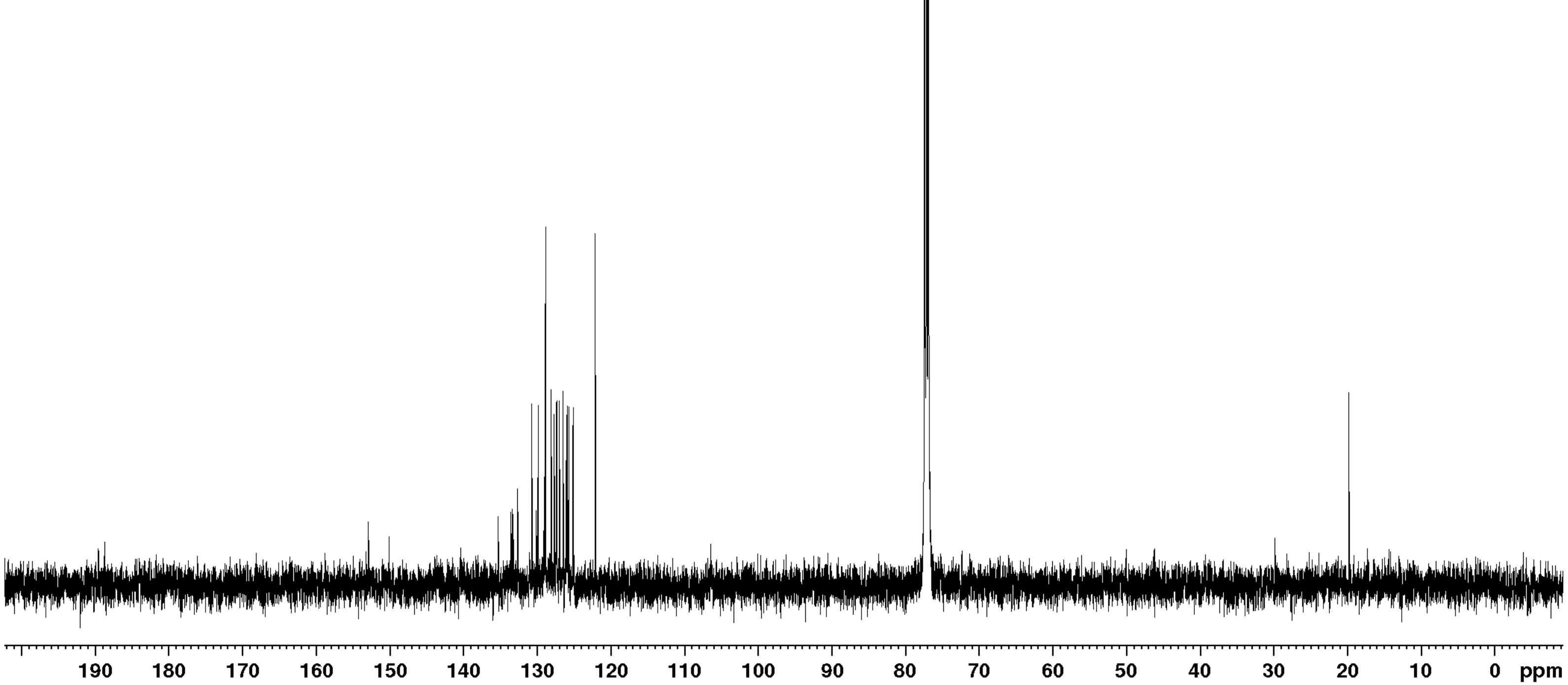

^{1}H NMR spectrum (400 MHz, $CDCl_3$, 298 K) of (*S*)-1-(3-methyl-[1,1'-binaphthalen]-2-yl)-2-phenyldiazene ((*S*)-**3db**)

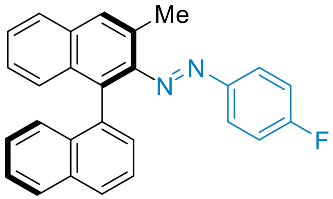

(*S*)-**3db**

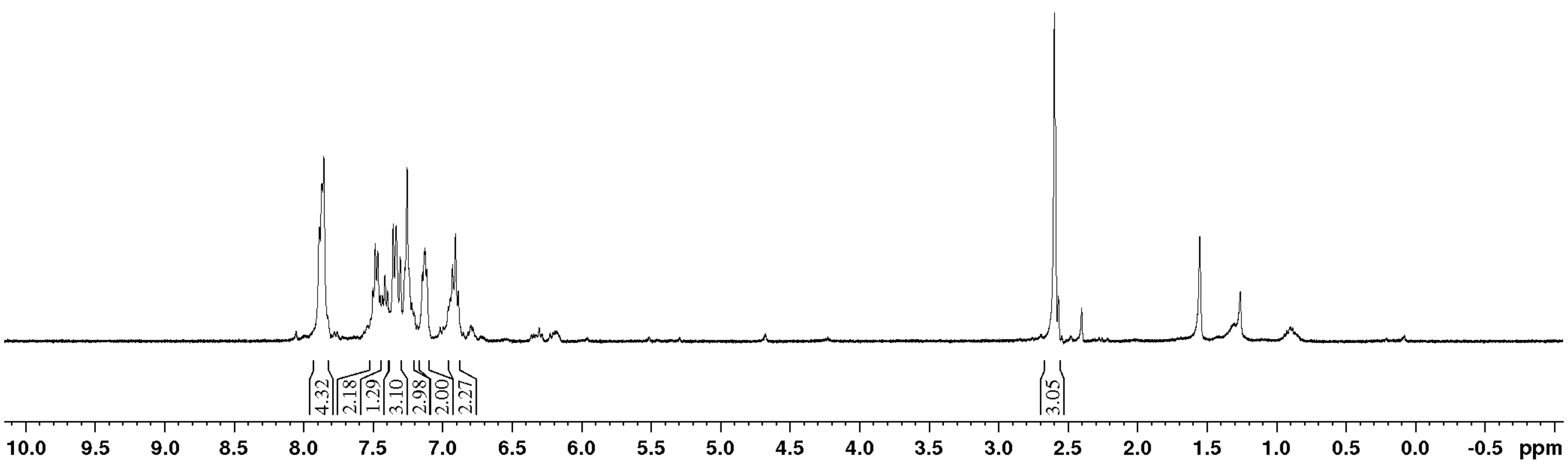

$^{13}C\{^{1}H\}$ NMR spectrum (101 MHz, $CDCl_3$, 298 K) of (*S*)-1-(3-methyl-[1,1'-binaphthalen]-2-yl)-2-phenyldiazene ((*S*)-**3db**)

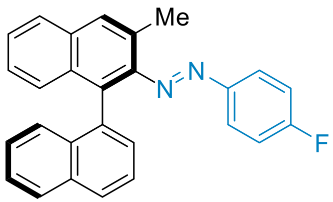

(*S*)-**3db**

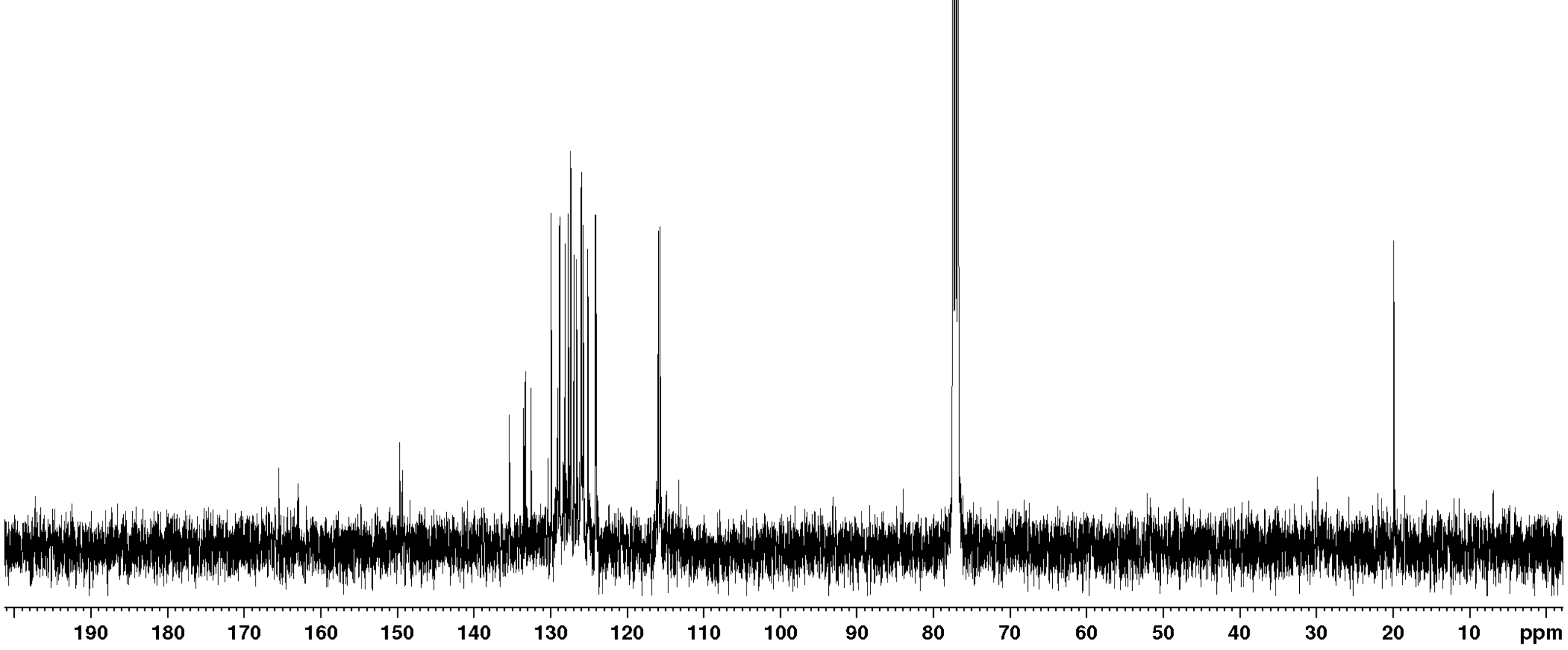

^{1}H NMR spectrum (400 MHz, $CDCl_3$, 298 K) of (*S*)-1-(3-fluoro-[1,1'-binaphthalen]-2-yl)-2-phenyldiazene ((*S*)-**3ea**)

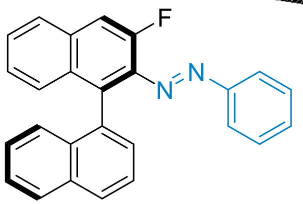

(*S*)-**3ea**

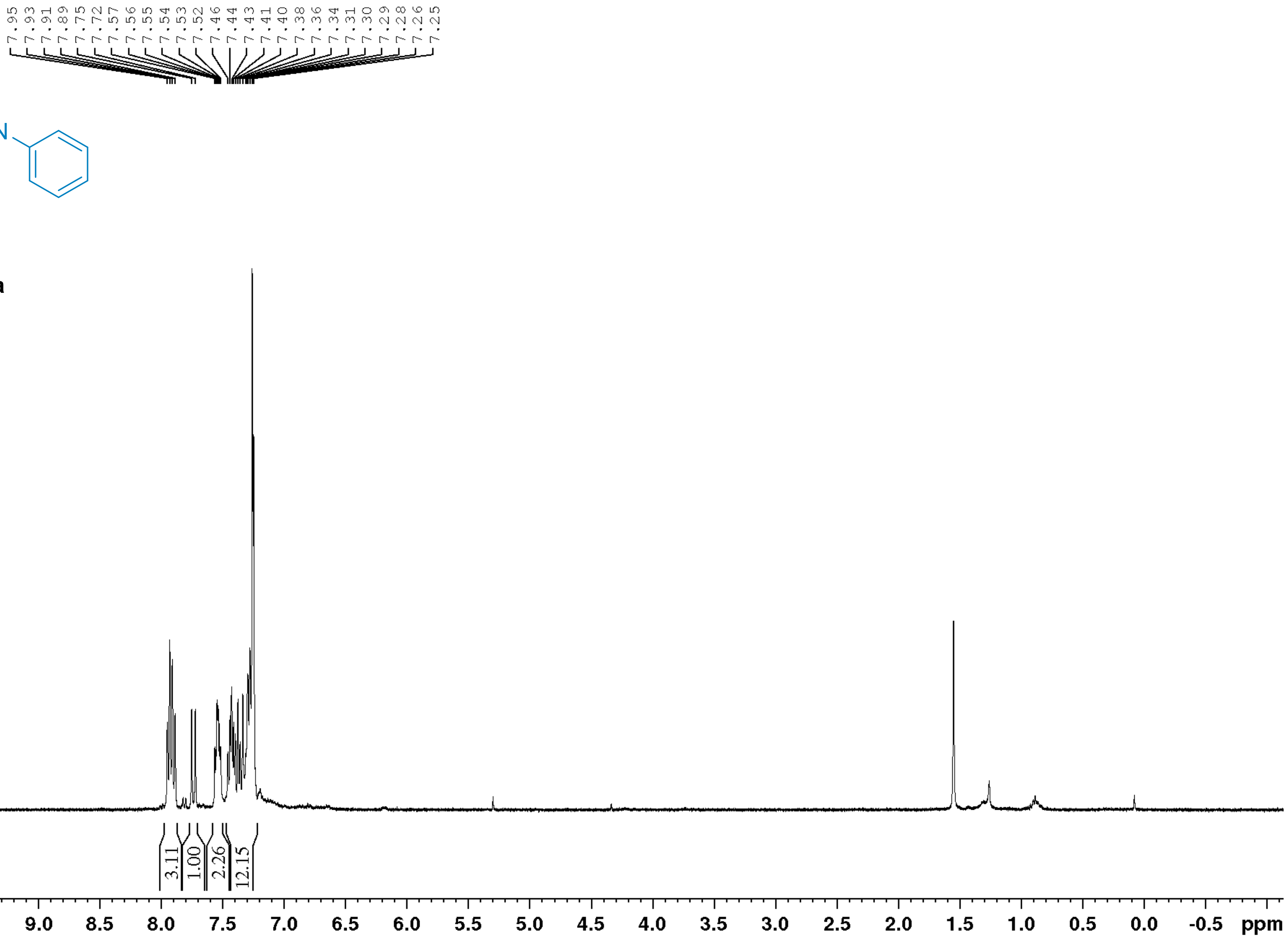

$^{13}C\{^{1}H\}$ NMR spectrum (101 MHz, $CDCl_3$, 298 K) of (*S*)-1-(3-fluoro-[1,1'-binaphthalen]-2-yl)-2-phenyldiazene ((*S*)-**3ea**)

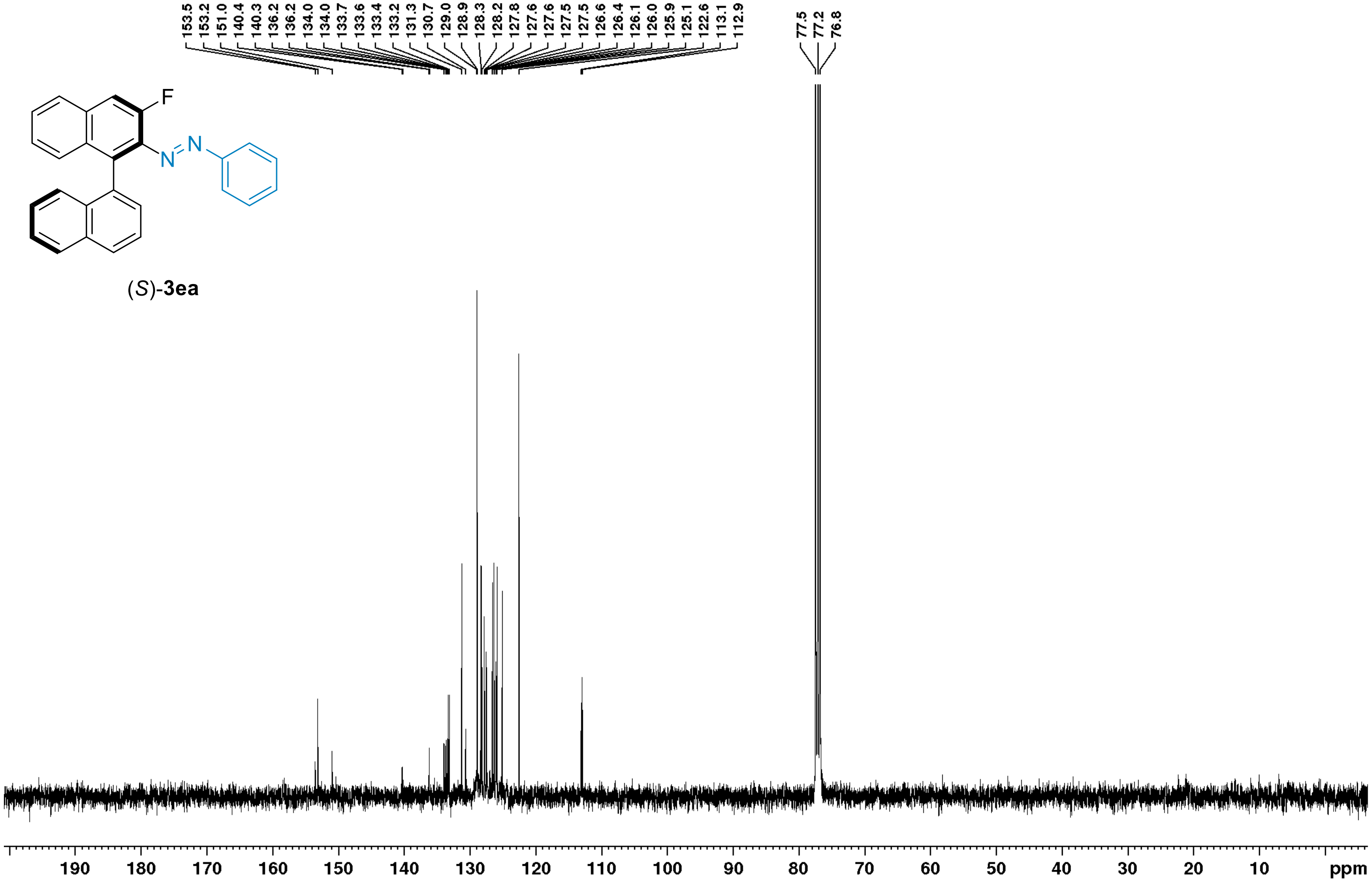

^{19}F NMR spectrum (471 MHz, $CDCl_3$, 298 K) of (*S*)-1-(3-fluoro-[1,1'-binaphthalen]-2-yl)-2-phenyldiazene ((*S*)-**3ea**)

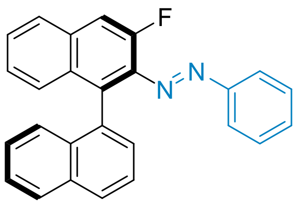

(*S*)-**3ea**

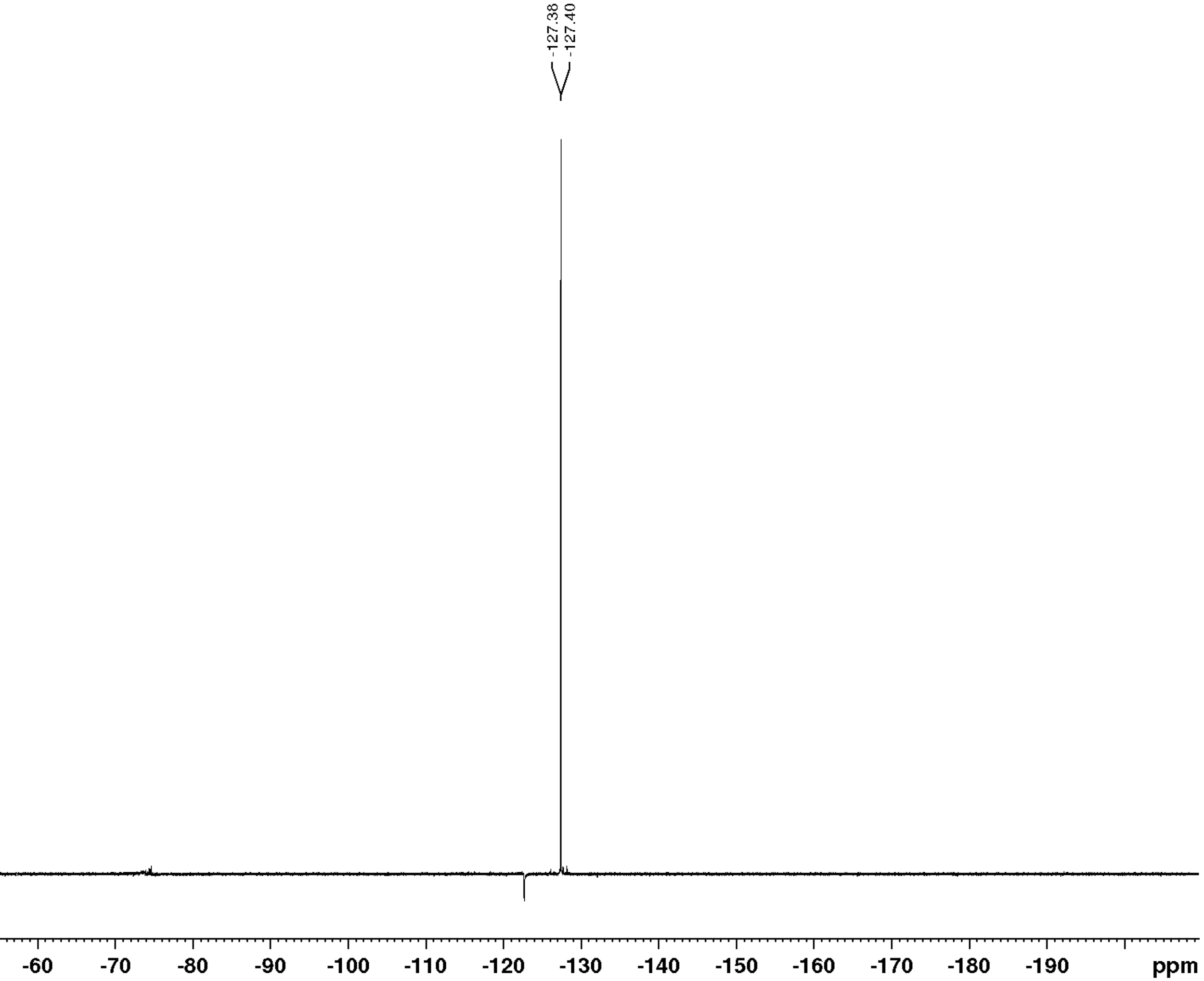

^{1}H NMR spectrum (500 MHz, $CDCl_3$, 298 K) of (*S*)-1-(1-(phenanthren-9-yl)naphthalen-2-yl)-2-(p-tolyl)diazene ((*S*)-**3fe**)

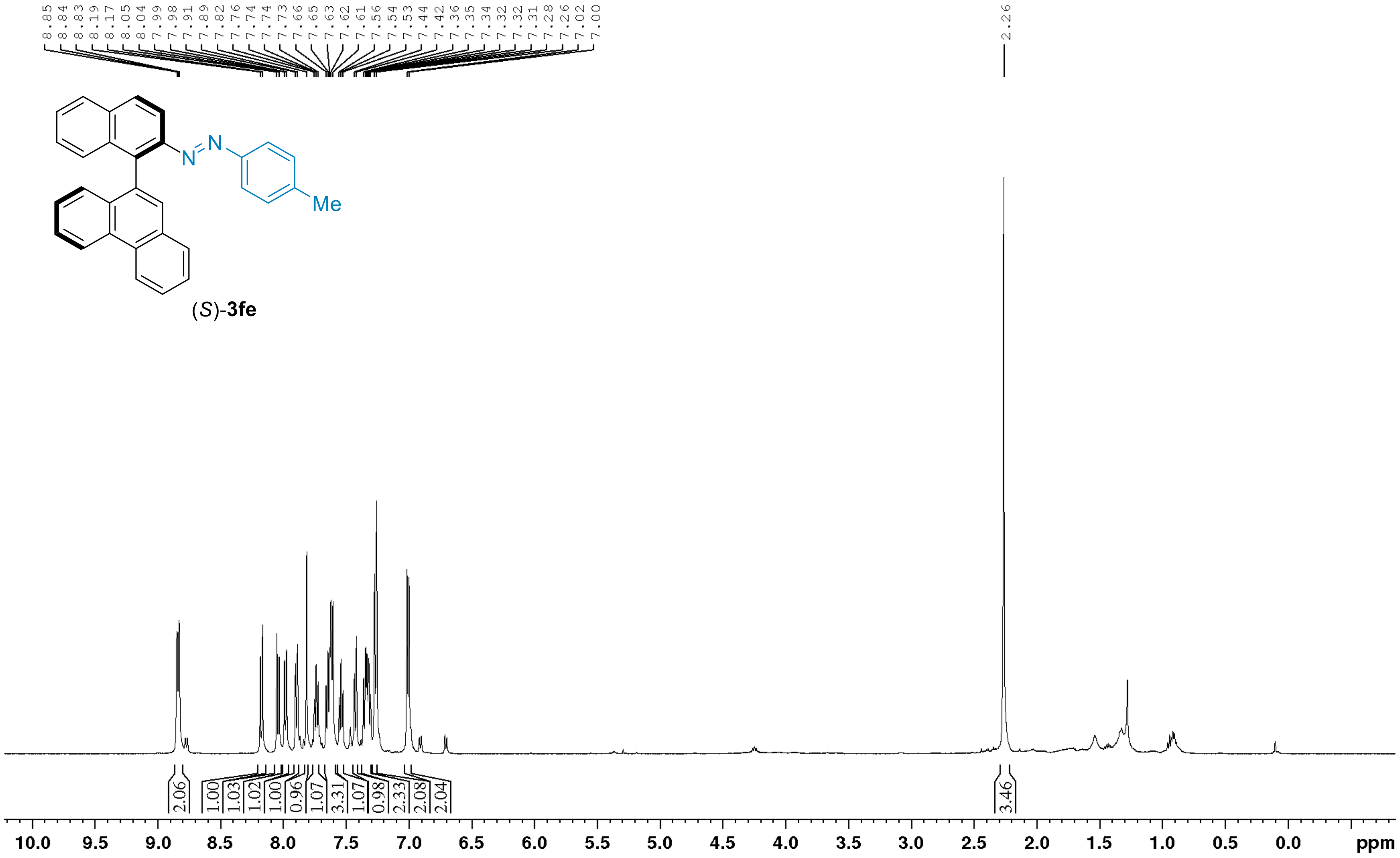

$^{13}C\{^1H\}$ NMR spectrum (126 MHz, $CDCl_3$, 298 K) of (*S*)-1-(1-(phenanthren-9-yl)naphthalen-2-yl)-2-(p-tolyl)diazene ((*S*)-**3fe**)

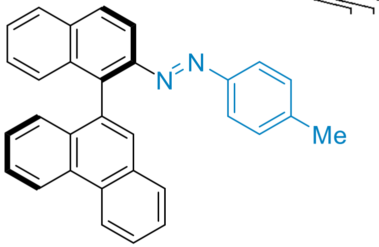

(*S*)-**3fe**

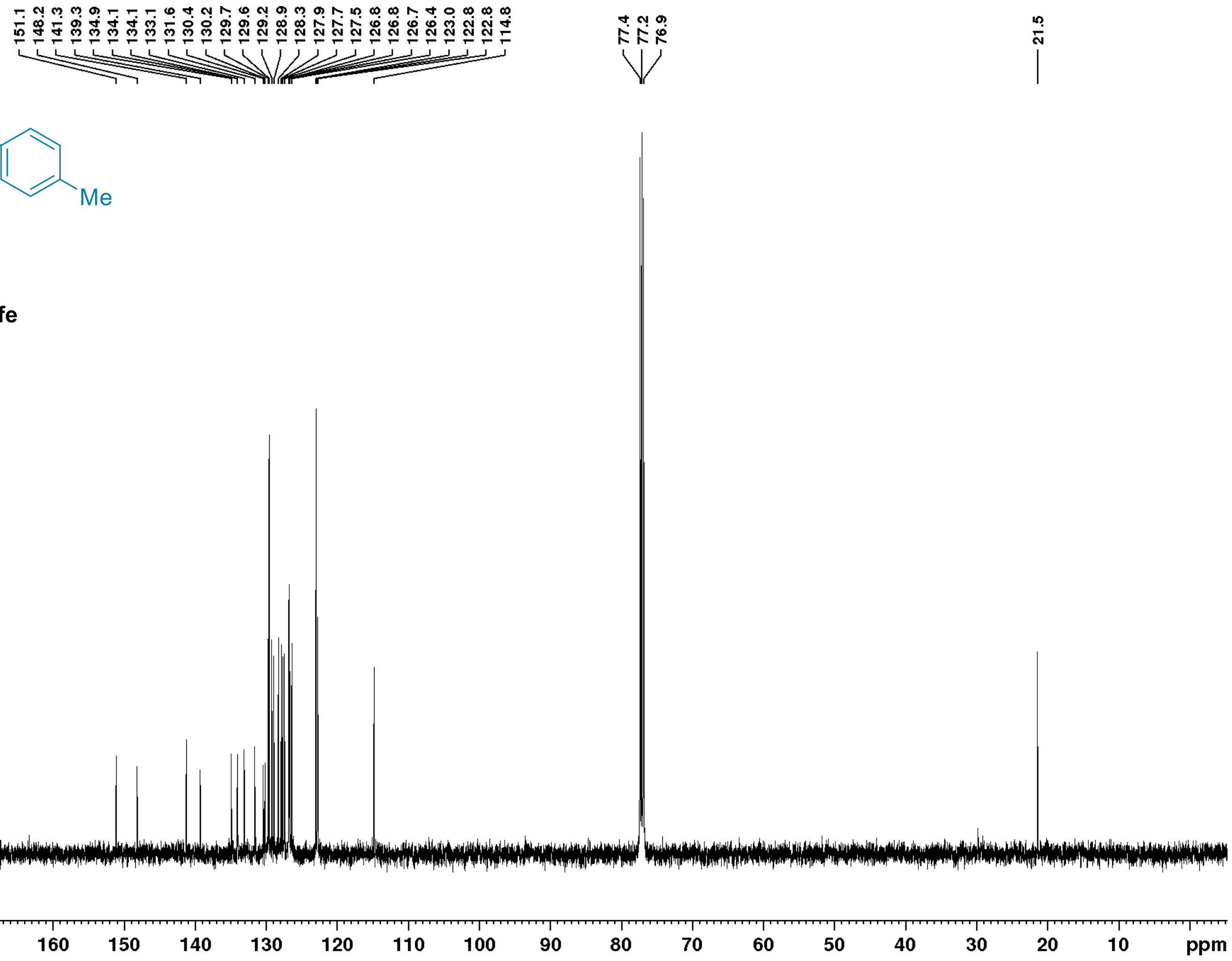

^{1}H NMR spectrum (500 MHz, $CDCl_3$, 298 K) of (*S*)-1-(1-(2-methoxyphenyl)naphthalen-2-yl)-2-phenyldiazene ((*S*)-**3ga**)

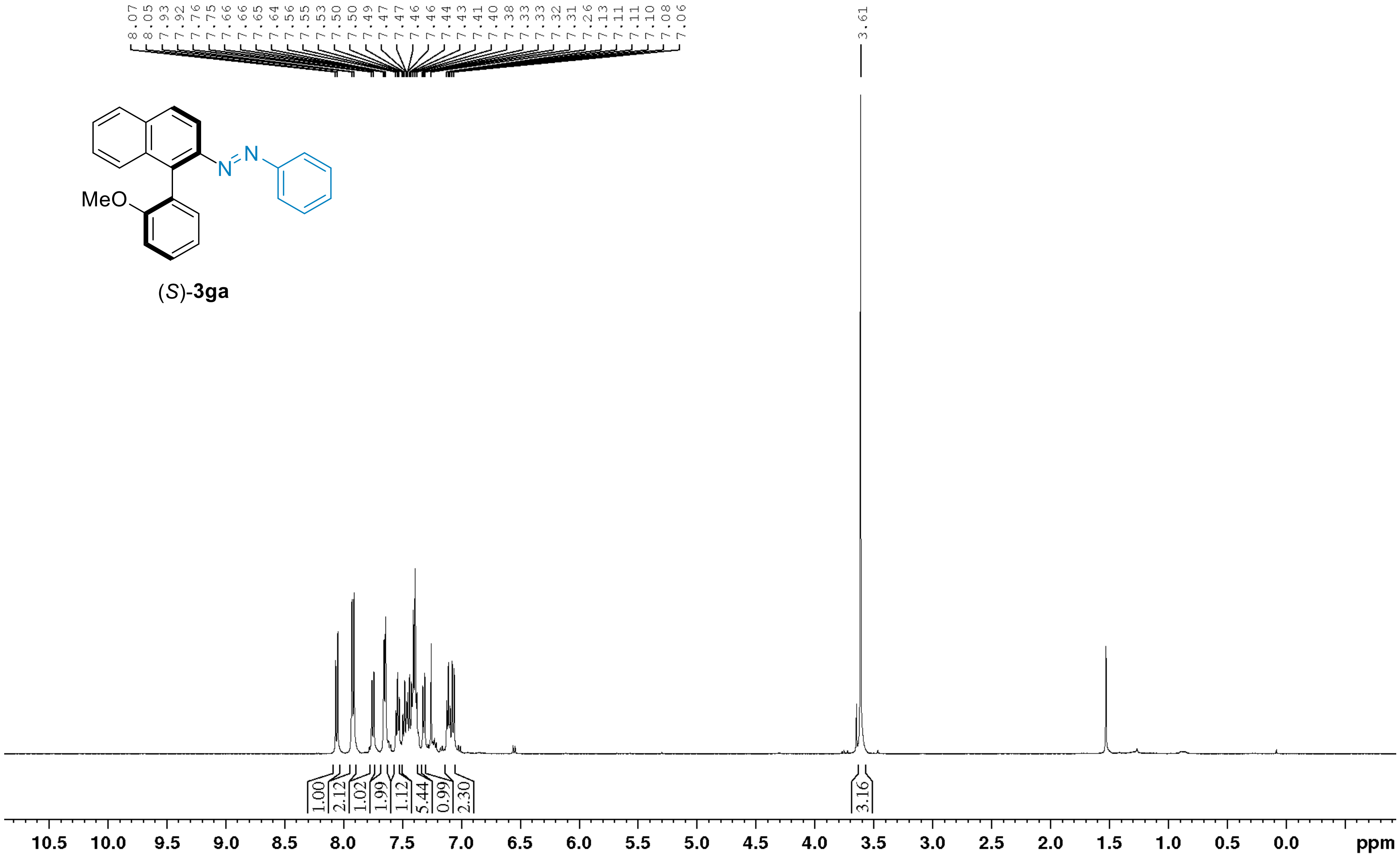

$^{13}C\{^{1}H\}$ NMR spectrum (126 MHz, $CDCl_3$, 298 K) of (*S*)-1-(1-(2-methoxyphenyl)naphthalen-2-yl)-2-phenyldiazene ((*S*)-**3ga**)

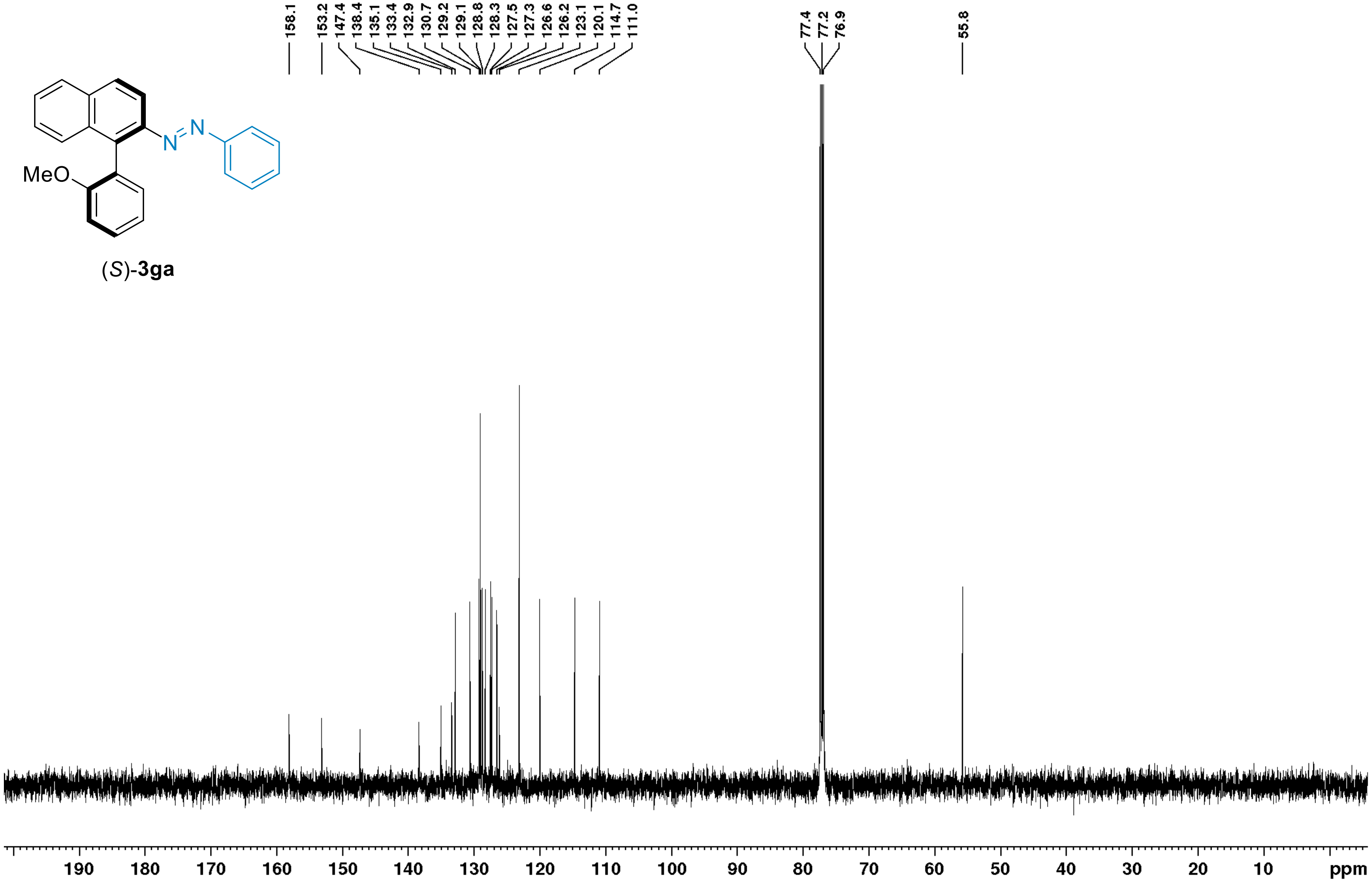

^{1}H NMR spectrum (500 MHz, $CDCl_3$, 298 K) of (*S*)-1-(4-fluorophenyl)-2-(1-(2-methoxyphenyl)naphthalen-2-yl)diazene ((*S*)-**3gb**)

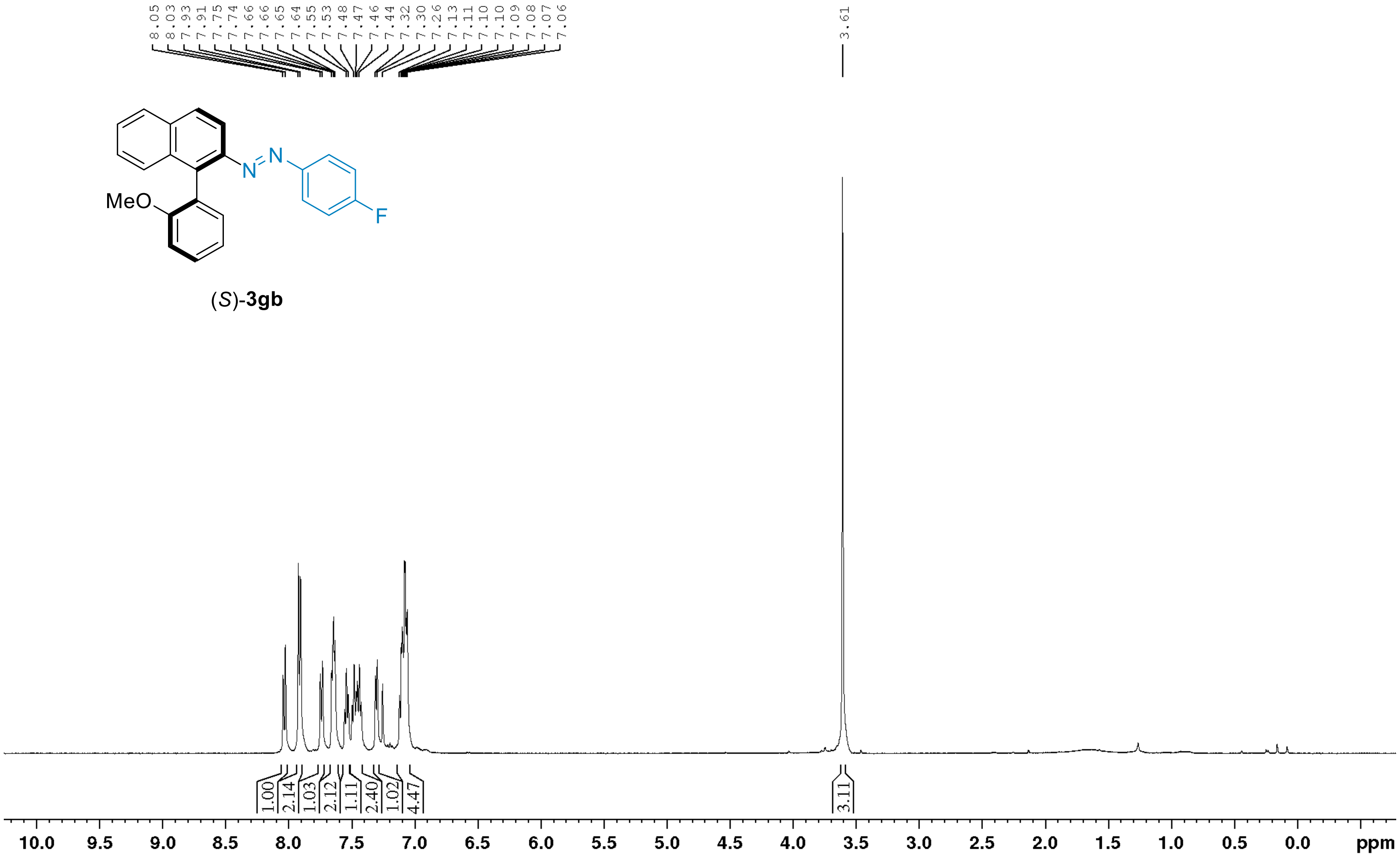

$^{13}C\{^{1}H\}$ NMR spectrum (126 MHz, $CDCl_3$, 298 K) of (*S*)-1-(4-fluorophenyl)-2-(1-(2-methoxyphenyl)naphthalen-2-yl)diazene ((*S*)-**3gb**)

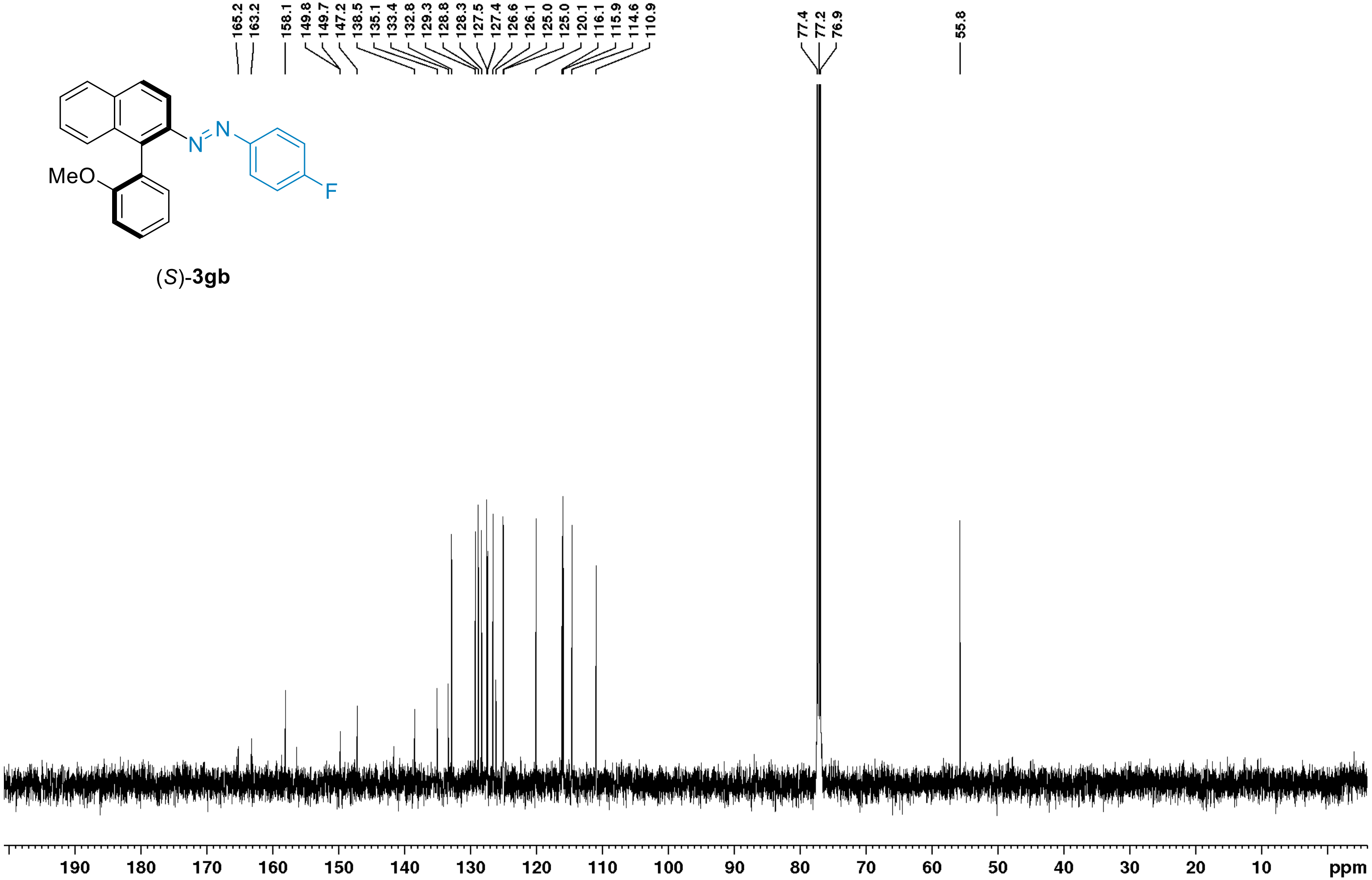

^{19}F NMR spectrum (500 MHz, $CDCl_3$, 298 K) of (*S*)-1-(4-fluorophenyl)-2-(1-(2-methoxyphenyl)naphthalen-2-yl)diazene ((*S*)-**3gb**)

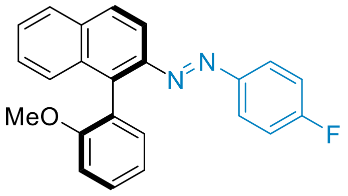

(*S*)-**3gb**

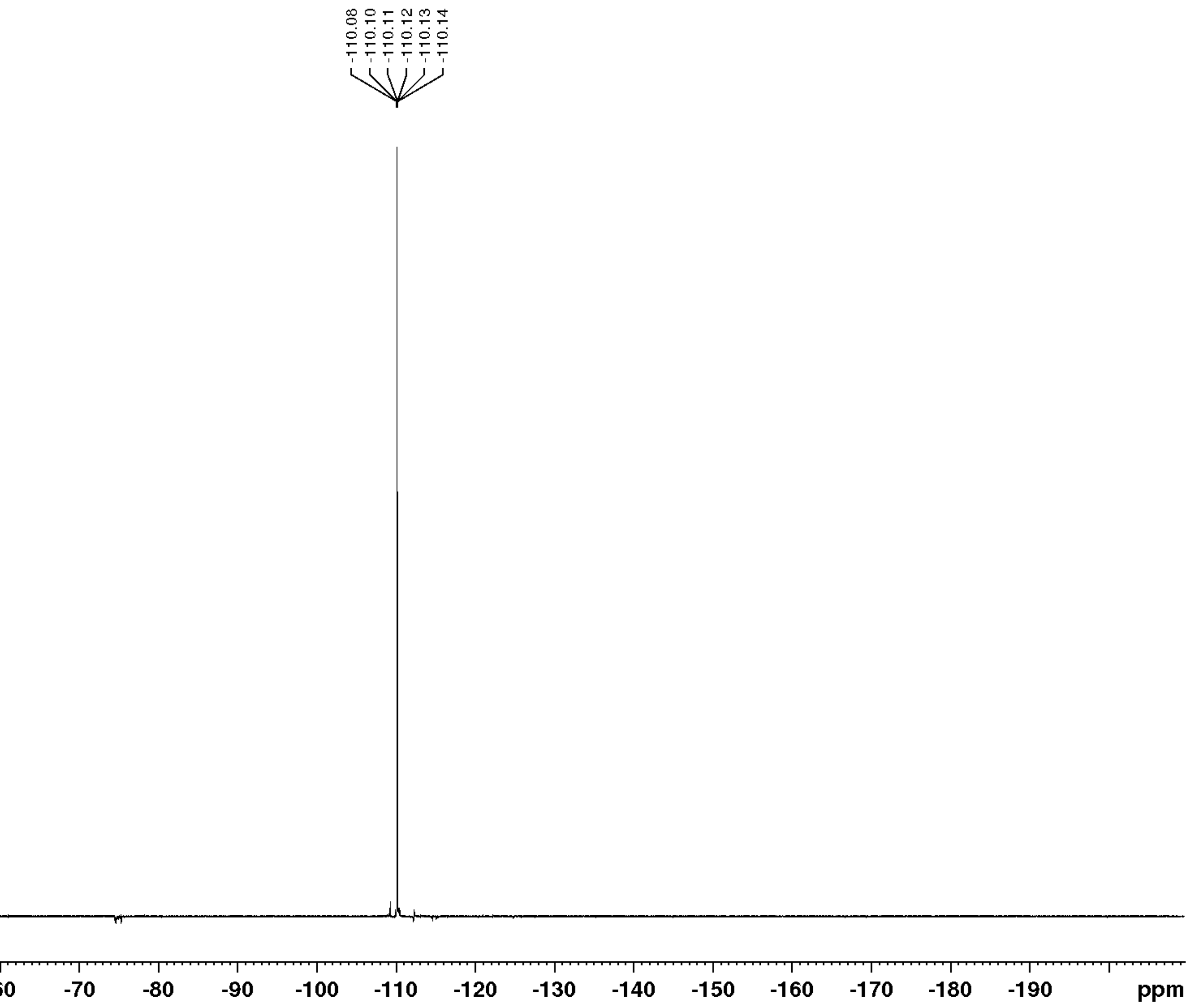

^{1}H NMR spectrum (500 MHz, $CDCl_3$, 298 K) of (*S*)-1-(1-(2-ethylphenyl)naphthalen-2-yl)-2-phenyldiazene ((*S*)-**3ha**)

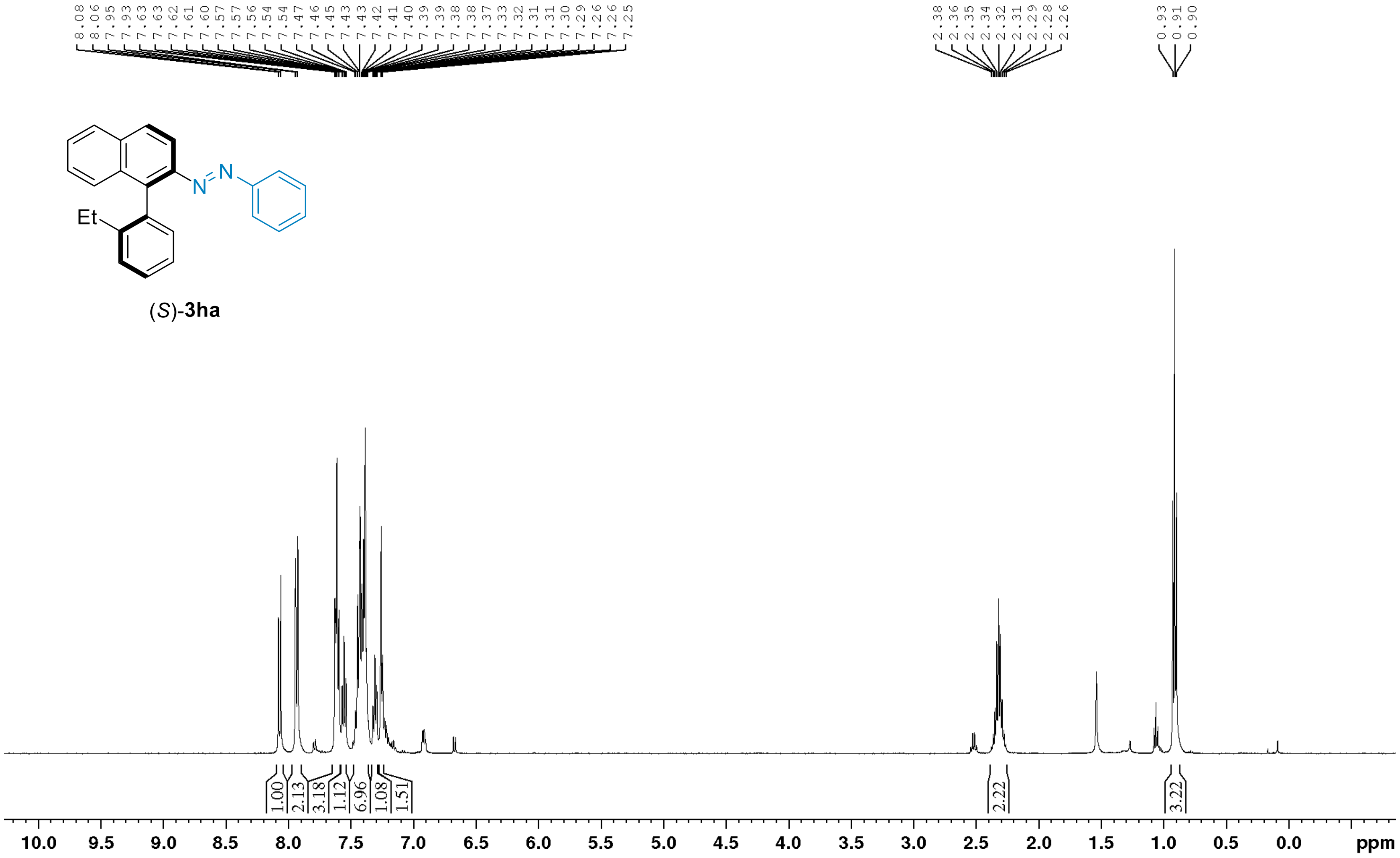

$^{13}C\{^{1}H\}$ NMR spectrum (126 MHz, $CDCl_3$, 298 K) of (*S*)-1-(1-(2-ethylphenyl)naphthalen-2-yl)-2-phenyldiazene ((*S*)-**3ha**)

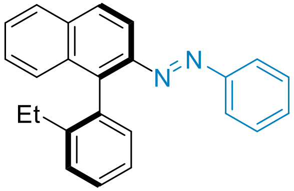

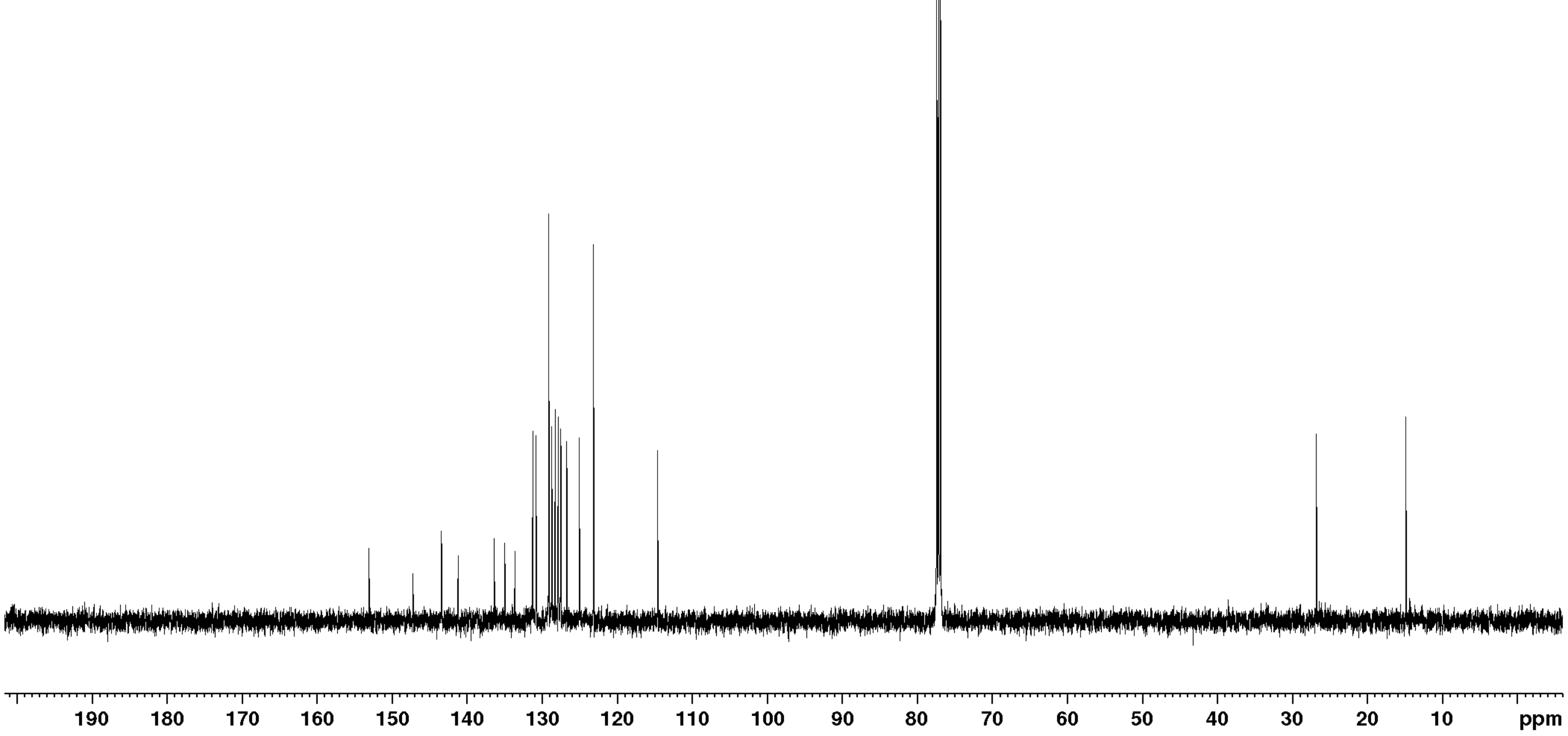

^{1}H NMR spectrum (500 MHz, $CDCl_3$, 298 K) of (*S*)-1-phenyl-2-(1-(o-tolyl)naphthalen-2-yl)diazene ((*S*)-**3ia**)

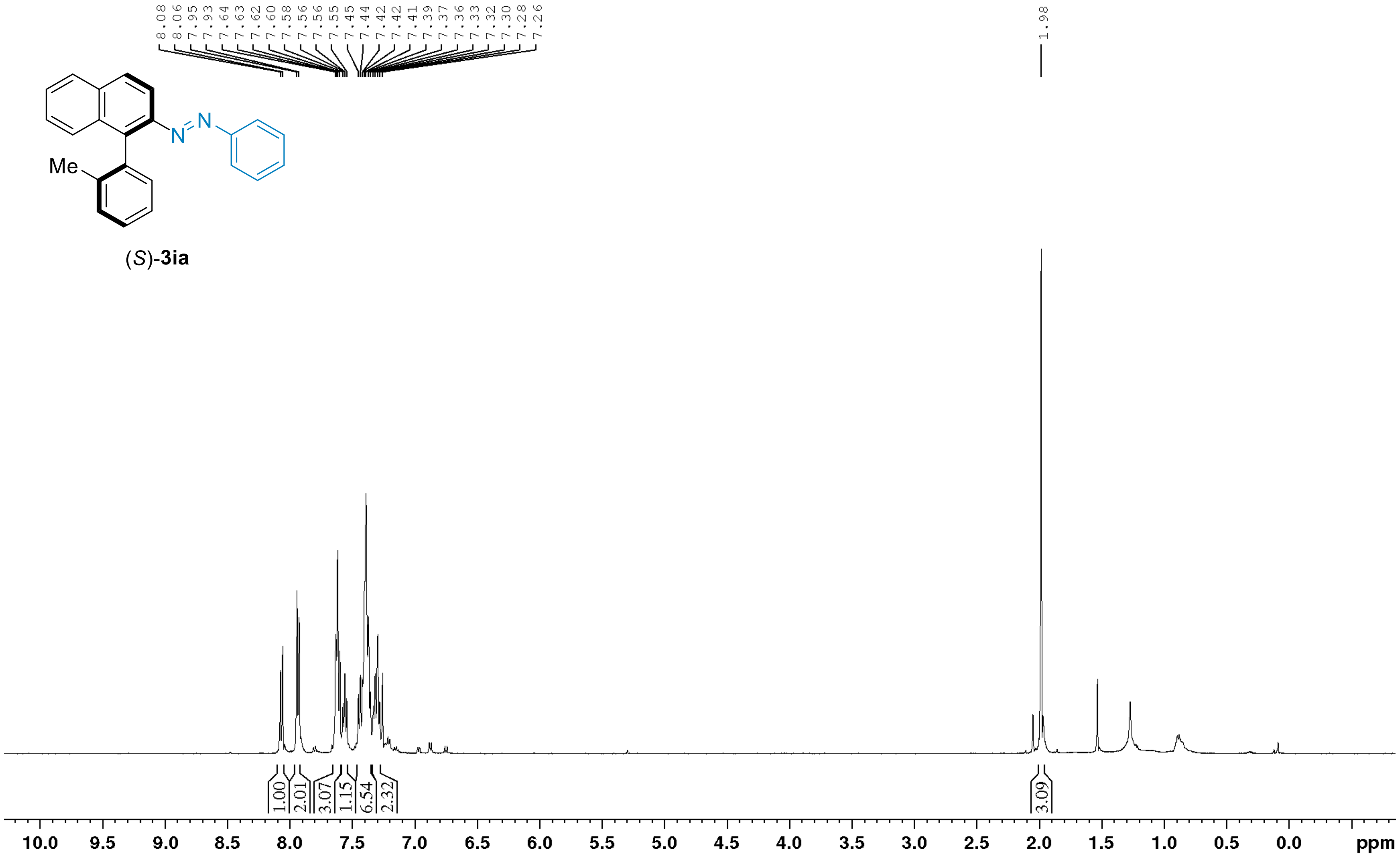

$^{13}C\{^{1}H\}$ NMR spectrum (500 MHz, $CDCl_3$, 298 K) of (*S*)-1-phenyl-2-(1-(o-tolyl)naphthalen-2-yl)diazene ((*S*)-**3ia**)

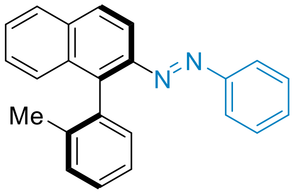

(*S*)-**3ia**

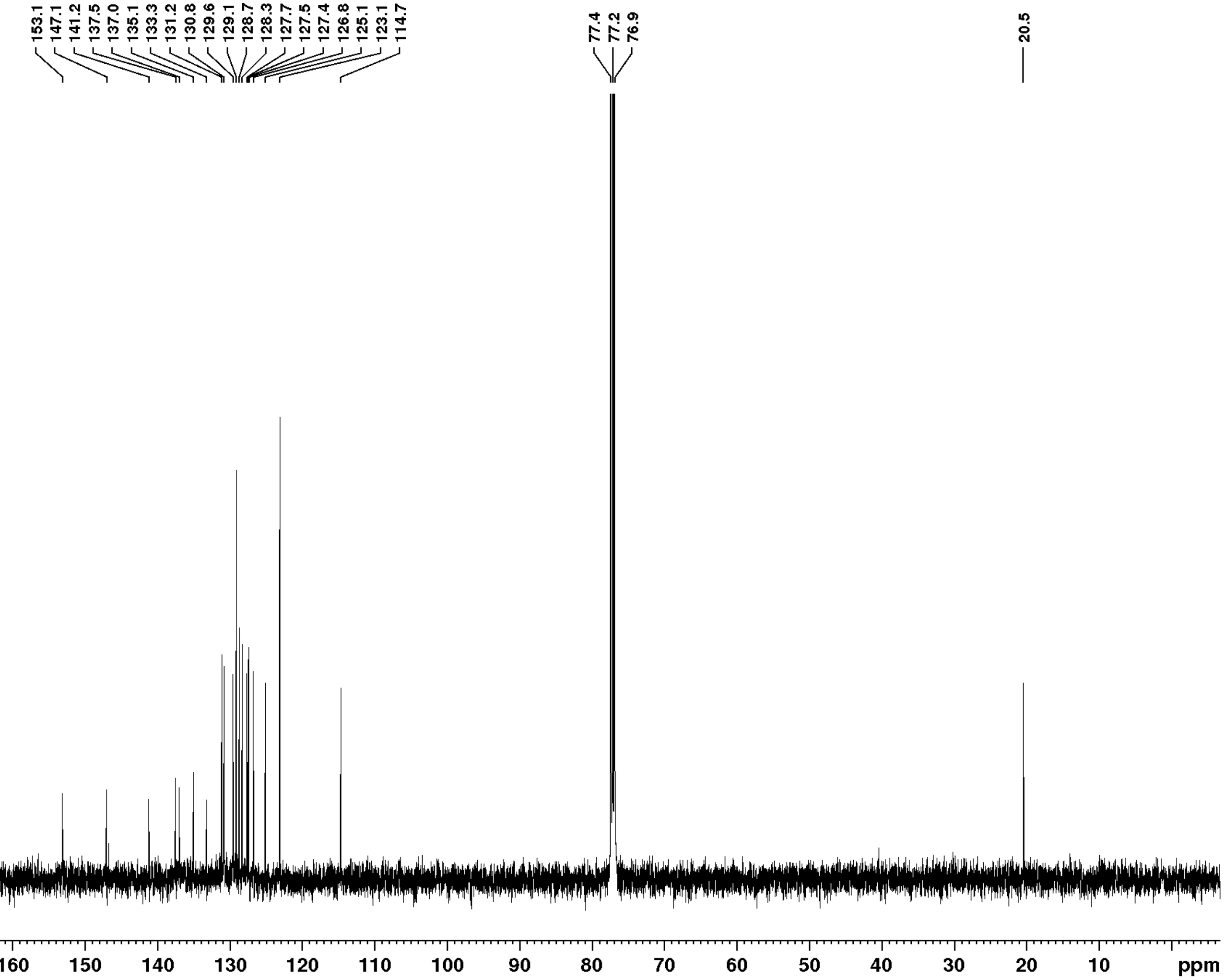

^{1}H NMR spectrum (500 MHz, $CDCl_3$, 298 K) of (*S*)-1-(1-(4-fluoro-2-methylphenyl)naphthalen-2-yl)-2-phenyldiazene ((*S*)-**3ja**)

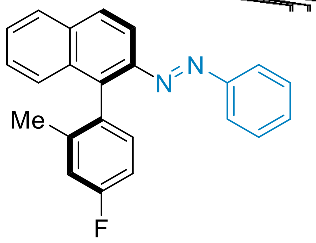

(*S*)-**3ja**

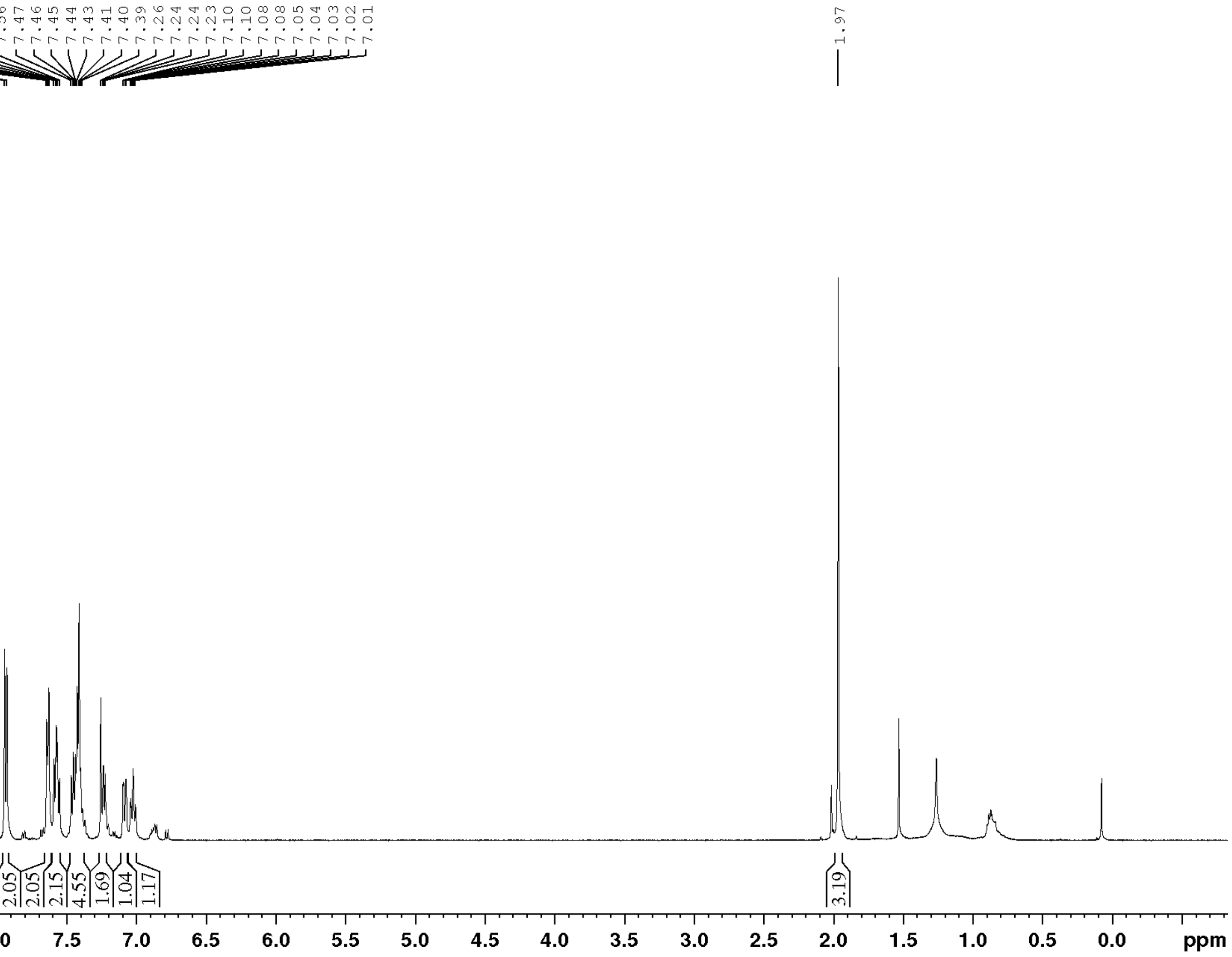

$^{13}C\{^{1}H\}$ NMR spectrum (101 MHz, $CDCl_3$, 298 K) of (*S*)-1-(1-(4-fluoro-2-methylphenyl)naphthalen-2-yl)-2-phenyldiazene ((*S*)-**3ja**)

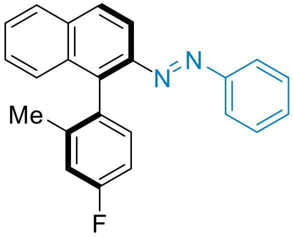

(*S*)-**3ja**

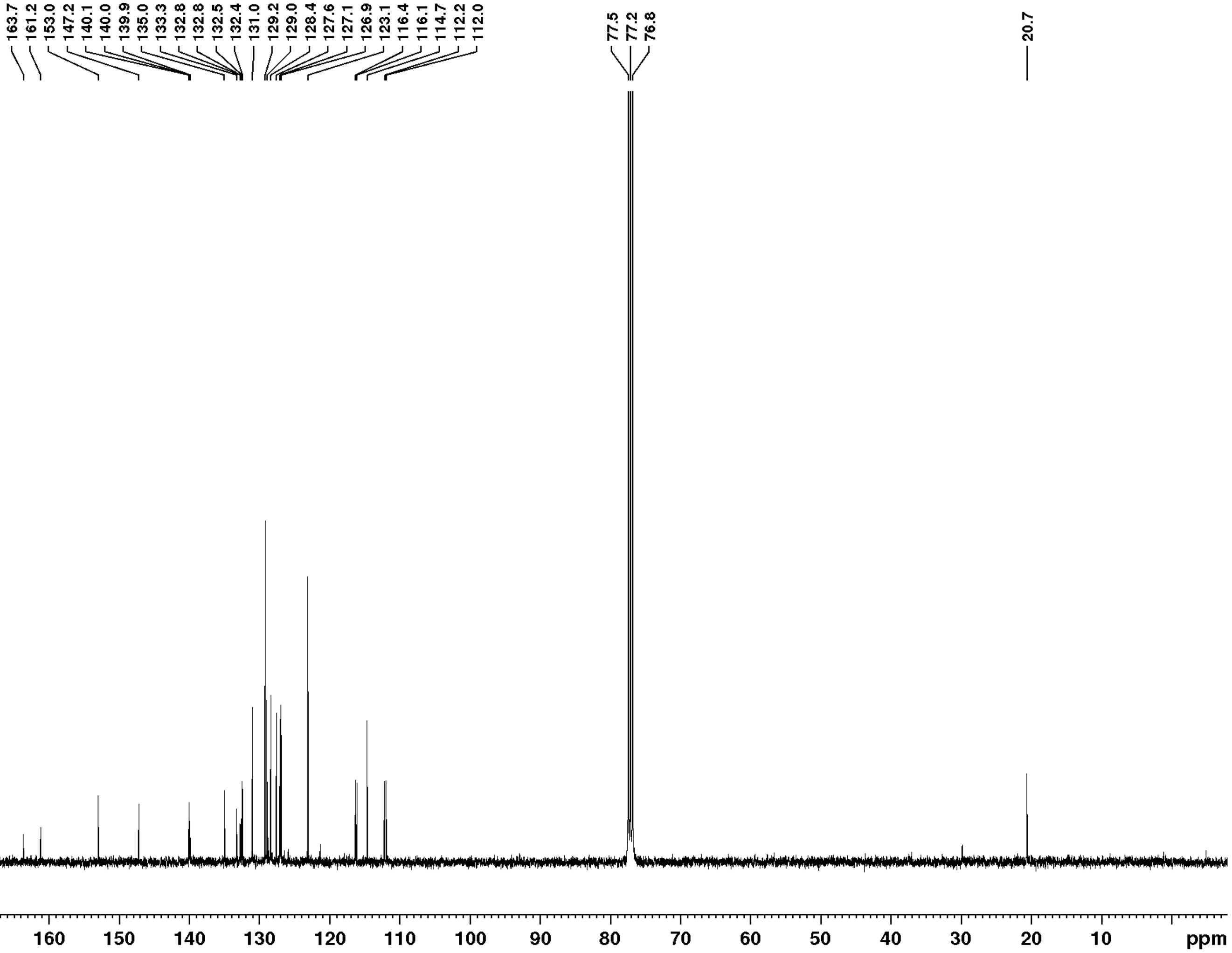

^{19}F NMR spectrum (471 MHz, $CDCl_3$, 298 K) of (*S*)-1-(1-(4-fluoro-2-methylphenyl)naphthalen-2-yl)-2-phenyldiazene ((*S*)-**3ja**)

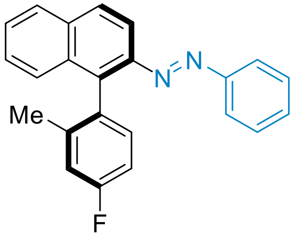

(*S*)-**3ja**

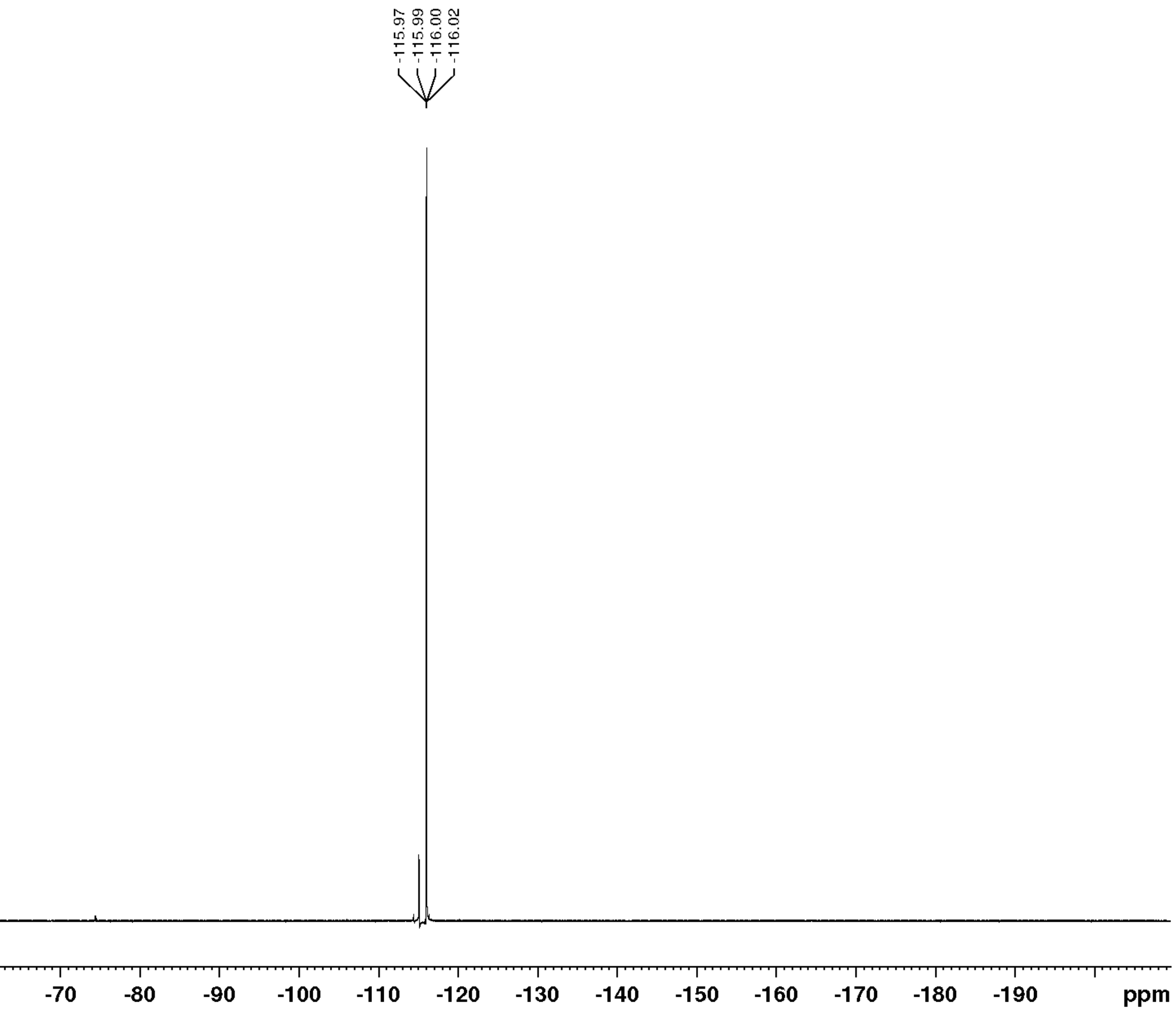

^{1}H NMR spectrum (500 MHz, $CDCl_3$, 298 K) of (*S*)-1-(1-(2-chlorophenyl)naphthalen-2-yl)-2-phenyldiazene ((*S*)-**3ka**)

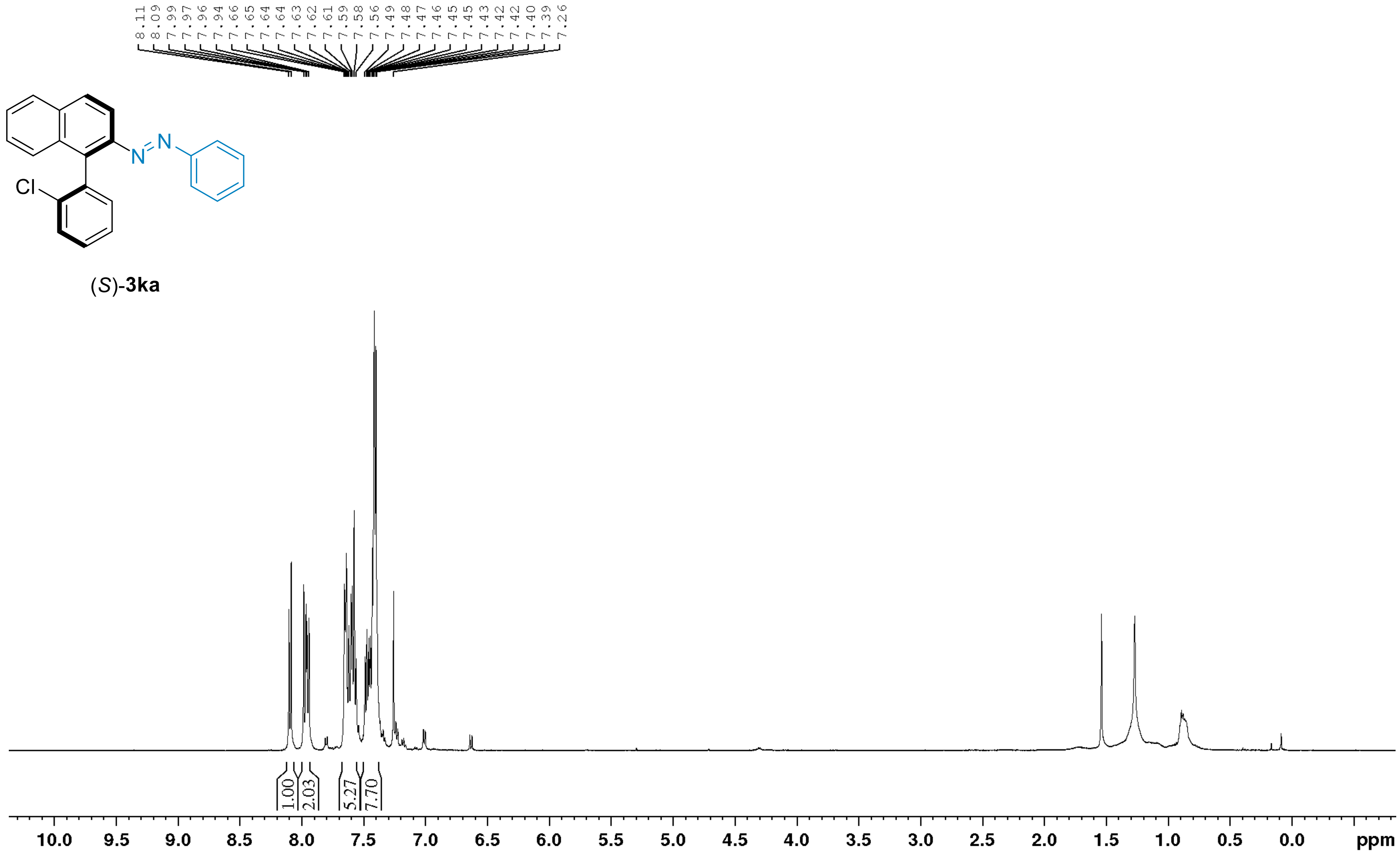

$^{13}C\{^1H\}$ NMR spectrum (126 MHz, $CDCl_3$, 298 K) of (*S*)-1-(1-(2-chlorophenyl)naphthalen-2-yl)-2-phenyldiazene ((*S*)-**3ka**)

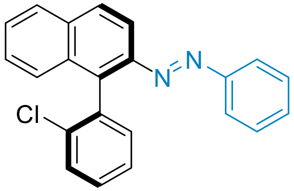

(*S*)-**3ka**

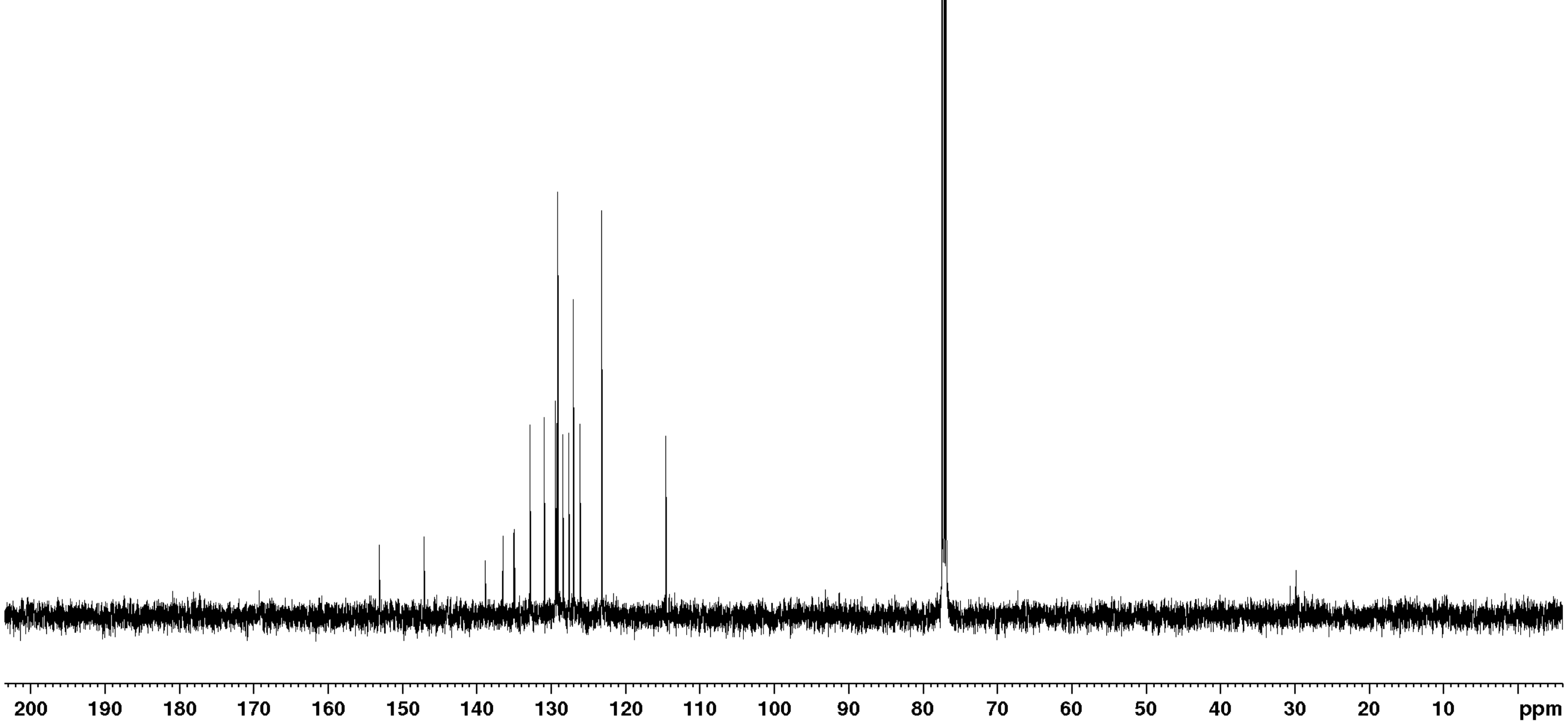

^{1}H NMR spectrum (500 MHz, $CDCl_3$, 298 K) of *(S)*-1-(1-([1,1'-biphenyl]-2-yl)naphthalen-2-yl)-2-phenyldiazene ((*S*)-**3la**)

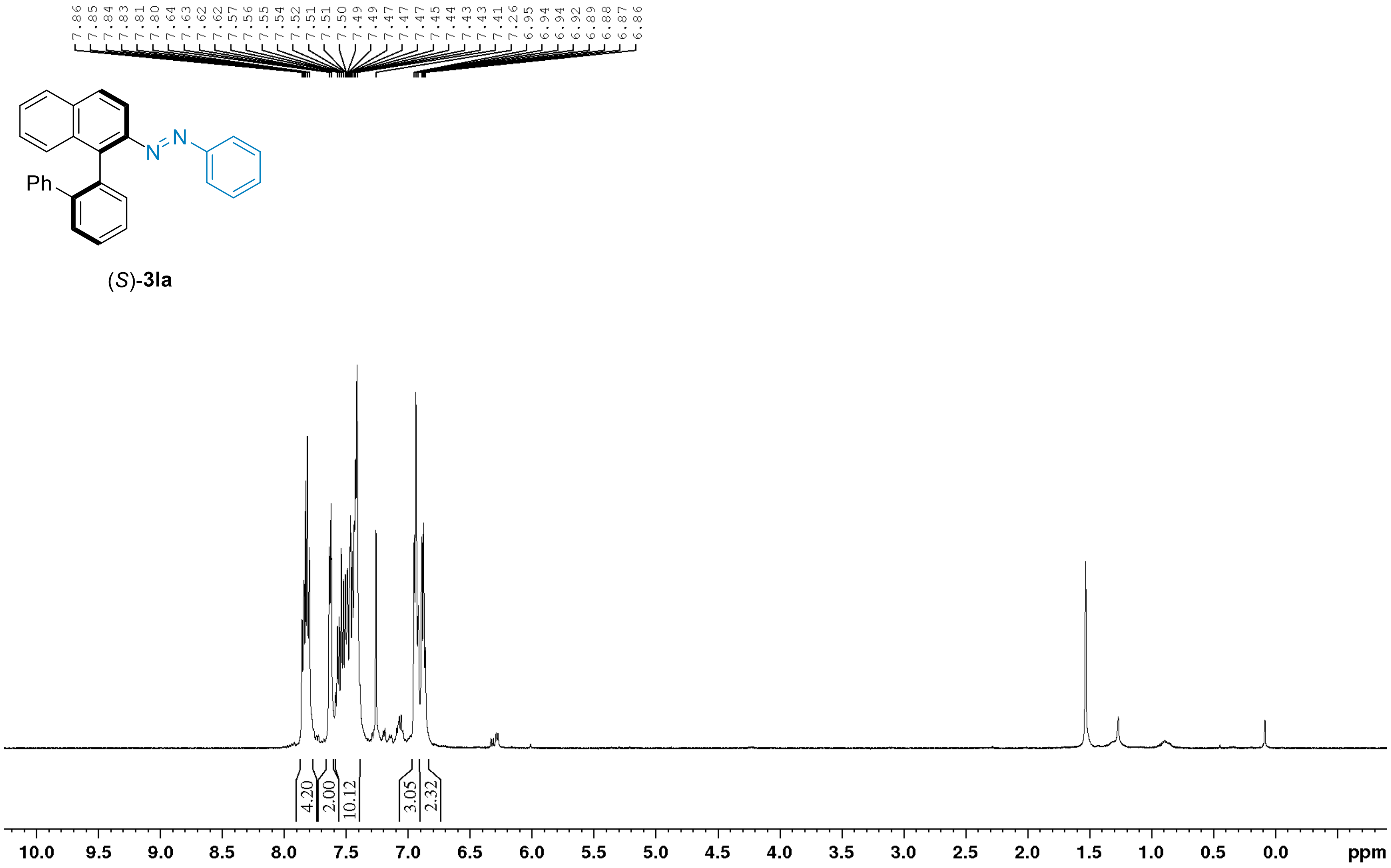

$^{13}C\{^1H\}$ NMR spectrum (126 MHz, $CDCl_3$, 298 K) of (*S*)-1-(1-([1,1'-biphenyl]-2-yl)naphthalen-2-yl)-2-phenyldiazene ((*S*)-**3la**)

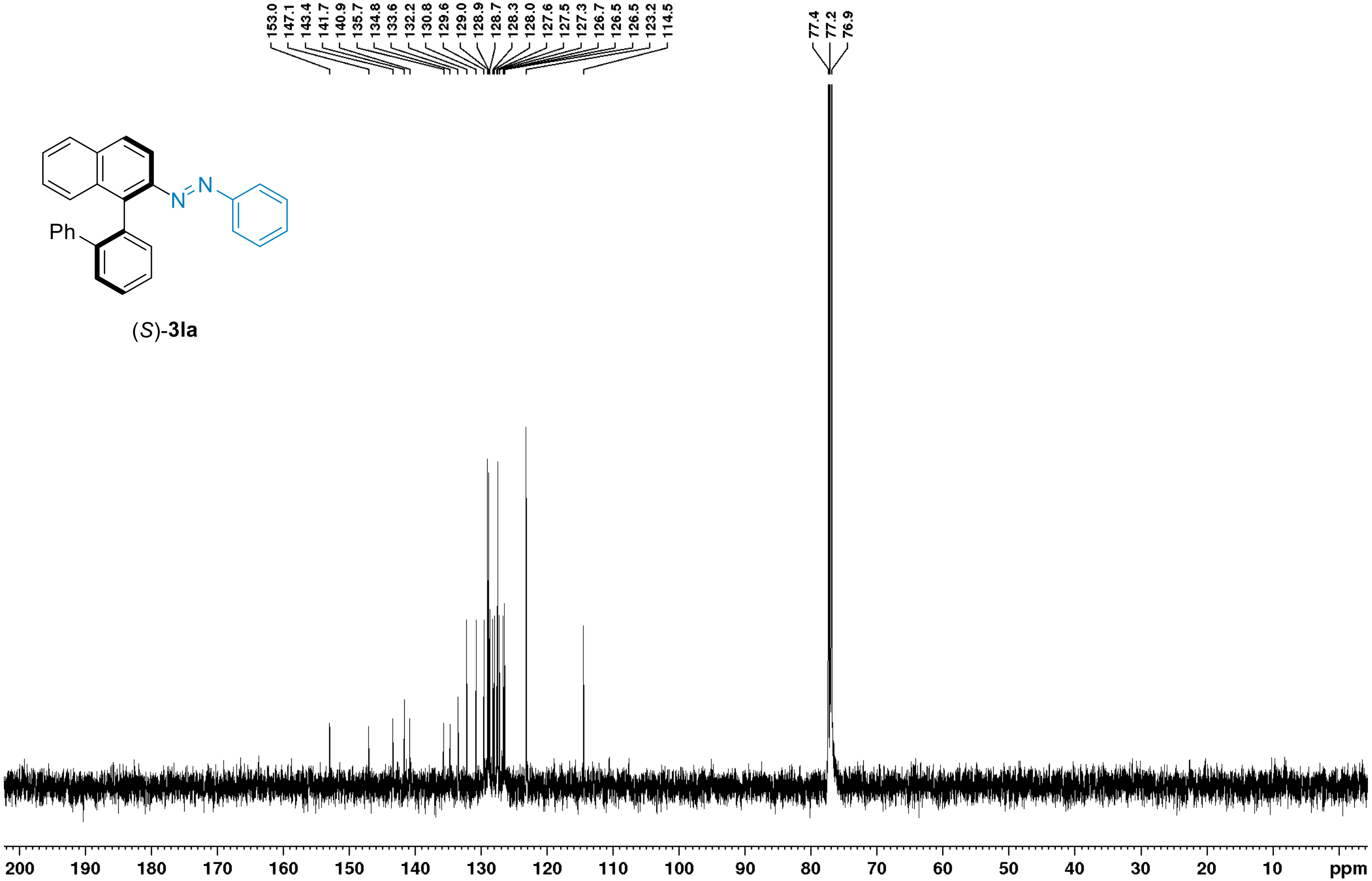

^{1}H NMR spectrum (400 MHz, C_6D_6, 298 K) of *cis*-(*S*)-1-([1,1'-binaphthalen]-2-yl)-2-phenyldiazene (*cis*-(*S*)-**3aa**)

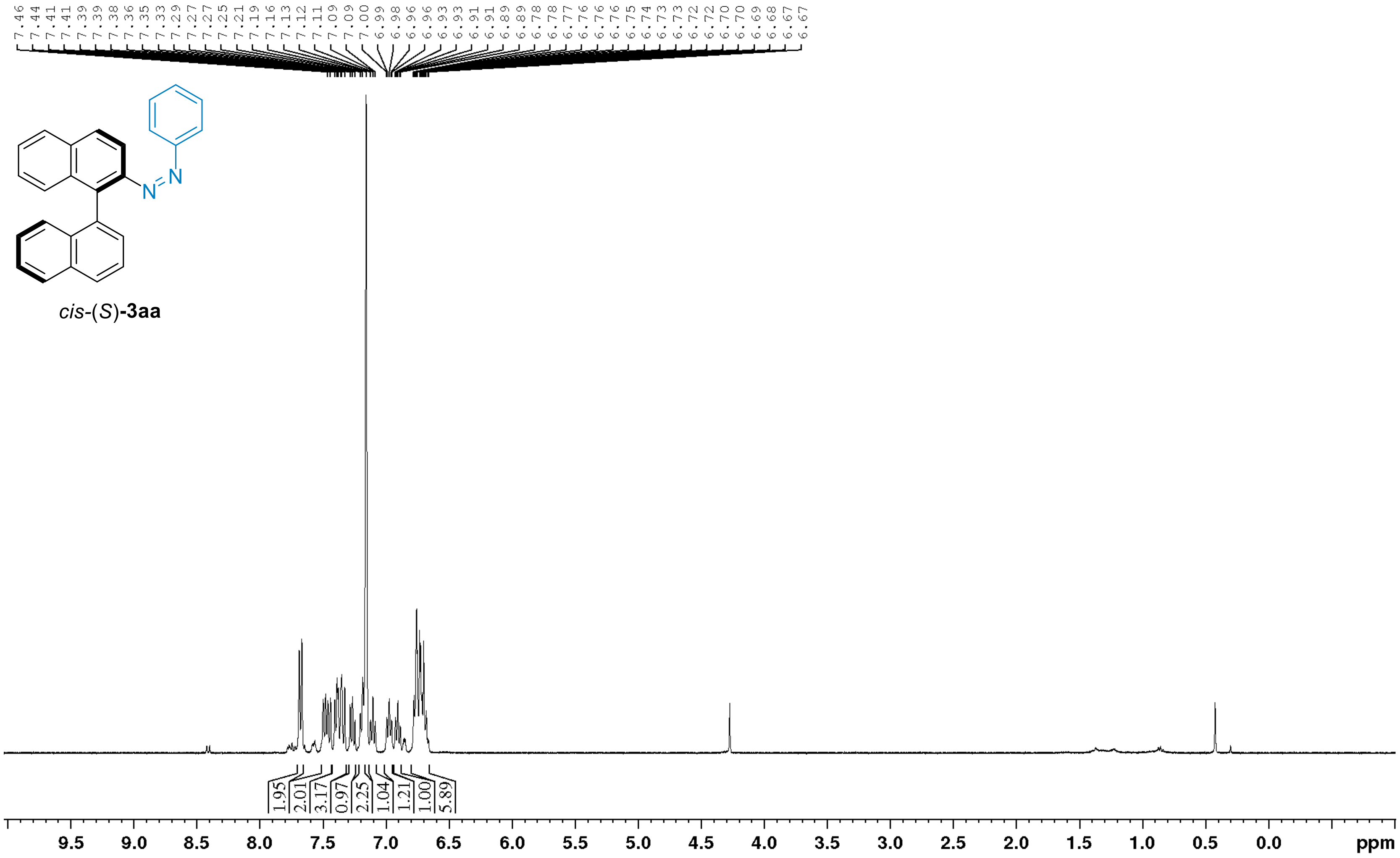

$^{13}C\{^1H\}$ NMR spectrum (101 MHz, $CDCl_3$, 298 K) of (*S,cis*)-1-([1,1'-binaphthalen]-2-yl)-2-phenyldiazene ((*S,cis*)-**3aa**)

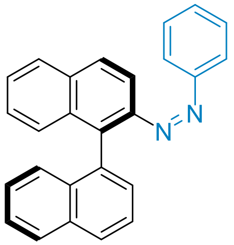

cis-(*S*)-**3aa**

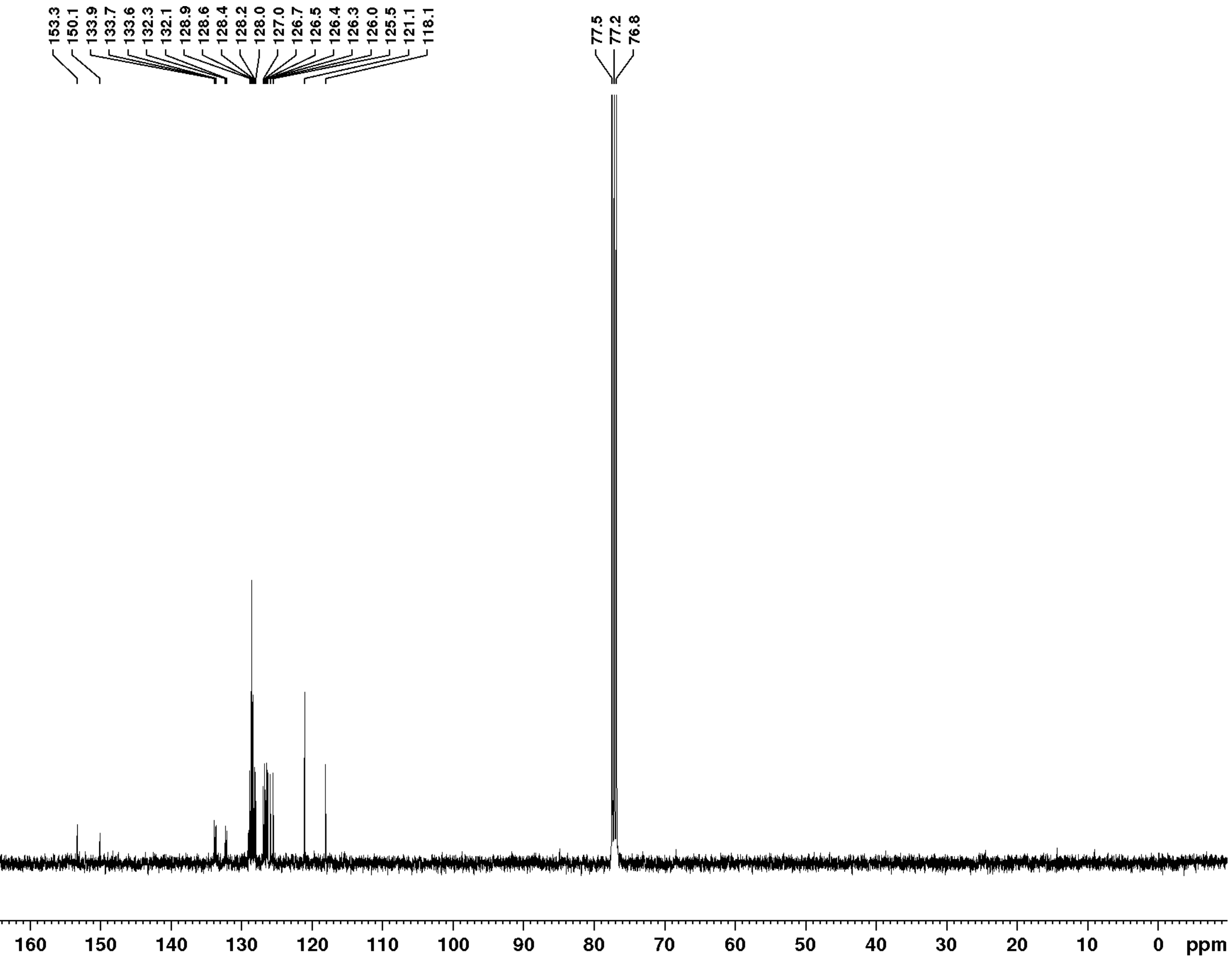

^{1}H NMR spectrum (400 MHz, $CDCl_3$, 298 K) of *N*-phenyl-7*H*-dibenzo[c,g]carbazol-7-amine (**4aa**)

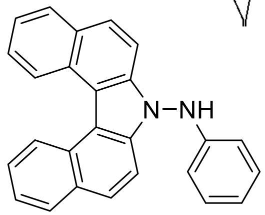

4aa

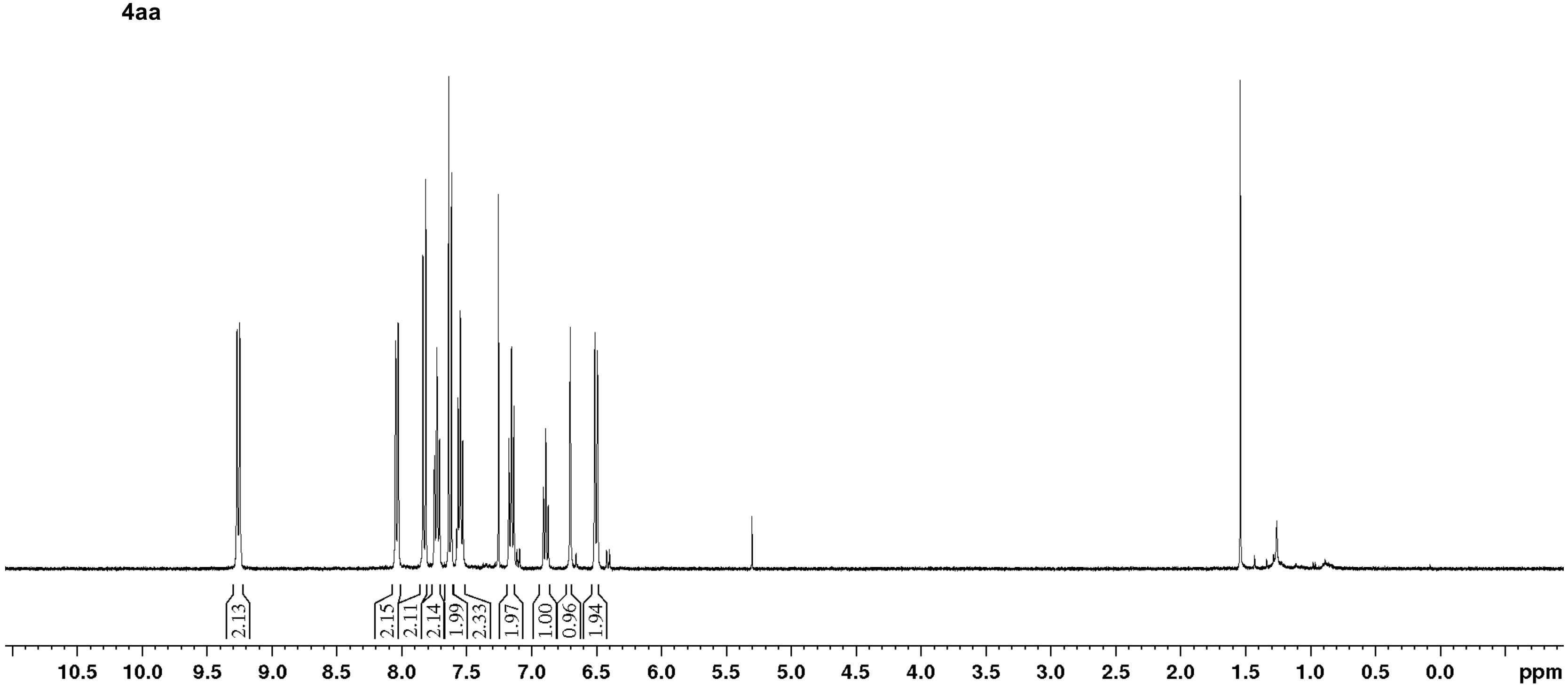

$^{13}C\{^{1}H\}$ NMR spectrum (101 MHz, $CDCl_3$, 298 K) of *N*-phenyl-7*H*-dibenzo[c,g]carbazol-7-amine (**4aa**)

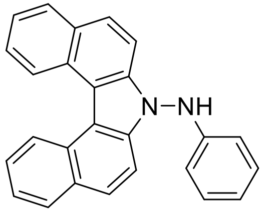

4aa

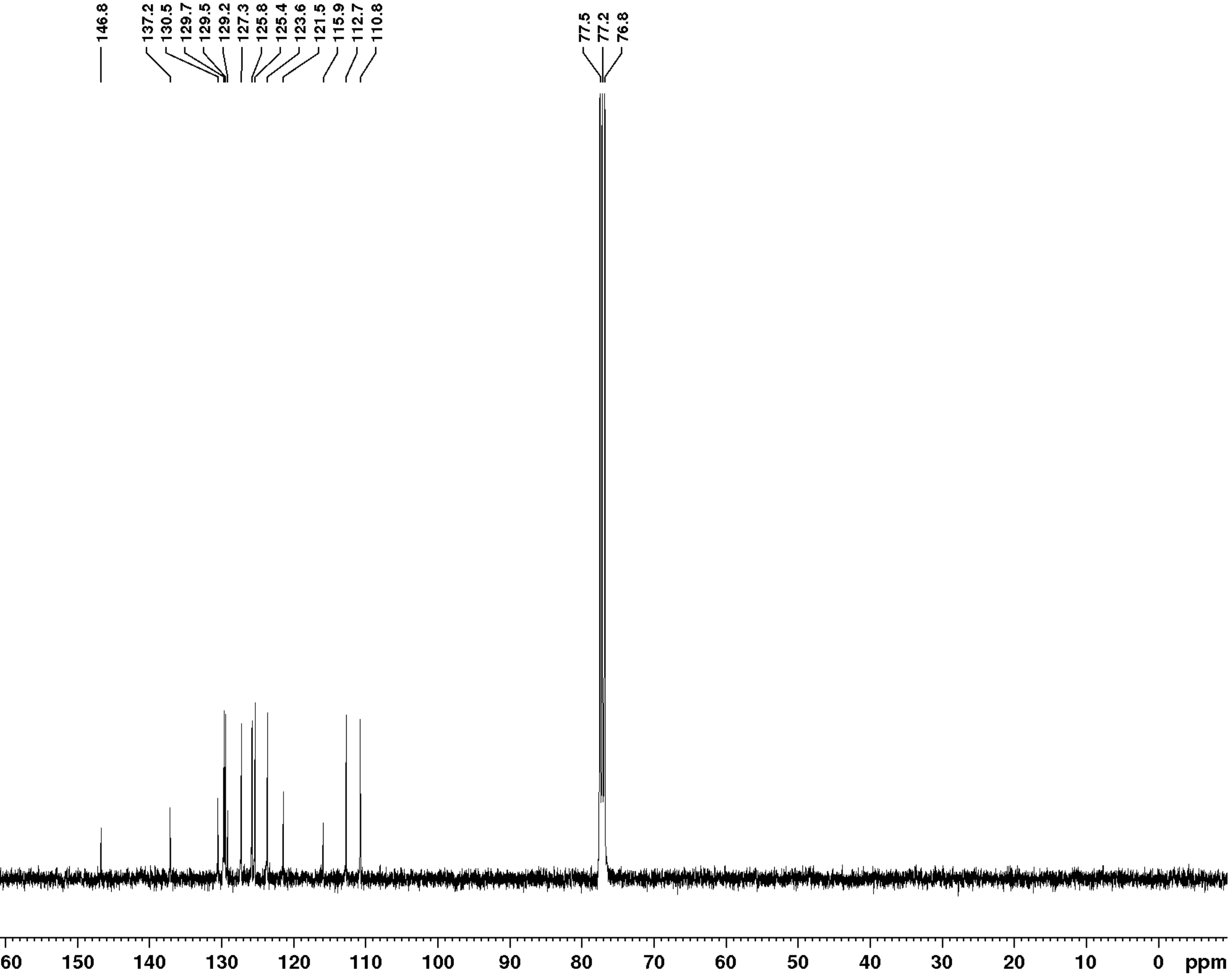

10 References

[S1] Losada, P.; Goicoechea, L.; Mascarenas, J. L.; Gulias, M. Axially Chiral 2-Hydroxybiaryls by Palladium-Catalyzed Enantioselective C-H Activation. *ACS Catal.* **2023**, *13*, 13994–13999.

[S2] Han, L.; Wang, H.; Luan, X. Pd(II)-Catalyzed [3 + 2] spiroannulation of *α*-aryl-*β*-naphthols with alkynes via a C–H activation/dearomatization approach. *Org. Chem. Front.* **2018**, *5*, 2453–2457.

[S3] Chauvier, C.; Finck, L.; Hecht, S.; Oestreich, M. General Synthesis and Optical Properties of *N*-Aryl-*N′*-Silyldiazenes. *Organometallics* **2019**, *38*, 4679–4686.

[S4] Ma, Y. N.; Zhang, H. Y.; Yang, S. D. Pd(II)-catalyzed P(O)R^1R^2-directed asymmetric C-H activation and dynamic kinetic resolution for the synthesis of chiral biaryl phosphates. *Org. Lett.* **2015**, *17*, 2034–2037.
